# Discovery of the First Highly Selective 1,4-dihydropyrido[3,4‑*b*]pyrazin-3(2H)-one MKK4 Inhibitor

**DOI:** 10.1021/acs.jmedchem.5c00919

**Published:** 2025-07-11

**Authors:** Leon Katzengruber, Pascal Sander, Stefan Zwirner, Alexander Rasch, Eric Eberlein, Roland Selig, Wolfgang Albrecht, Lars Zender, Stefan A. Laufer

**Affiliations:** † Department of Pharmaceutical/Medicinal Chemistry, 9188 Eberhard Karls University Tübingen, Auf der Morgenstelle 8, Tübingen 72076, Germany; ‡ Department of Chemical and Systems Biology, Stanford University, 290 Jane Stanford Way, Stanford, California 94305, United States; § Department of Medical Oncology and Pneumology (Internal Medicine VIII), University Hospital Tübingen,, Tübingen 72076, Germany; ∥ HepaRegenix GmbH, Eisenbahnstraße 63, Tübingen 72072, Germany; ⊥ IFIT Cluster of Excellence (EXC2180) “Image Guided and Functionally Instructed Tumor Therapies”, Eberhard Karls University of Tübingen, Tübingen 72076, Germany; # German Cancer Research Center (DKFZ), German Consortium for Translational Cancer Research (DKTK), Partner Site Tübingen, Heidelberg 69120, Germany; ¶ Tübingen Center for Academic Drug Discovery & Development (TüCAD_2_), Auf der Morgenstelle 8, Tübingen 72076, Germany

## Abstract

Due to limited treatment options, liver failure remains a major challenge in modern medicine. With the validation of mitogen-activated protein kinase kinase 4 (MKK4, also known as MEK4 or MAP2K4) as a regulator of hepatocyte regeneration, a promising target for curative treatment of degenerative liver diseases was recently identified via in vivo RNAi experiments. The field of small molecules targeting MKK4 is of growing interest. Several MKK4 inhibitors with differing scaffolds are known, but few have reasonable selectivity profiles and drug-like properties. To further explore the space of drug-like MKK4 scaffolds, we performed a broad screening campaign and identified BI-D1870 as a promising candidate. The dihydropteridinone BI-D1870 is an unselective ribosomal S6 kinase inhibitor with broad off-target activity. In the study presented herein, we report a successful off-to-on target strategy that led to the development of highly selective 1,4-dihydropyrido­[3,4-*b*]­pyrazin-3­(2H)-one inhibitors of MKK4.

## Graphical abstract

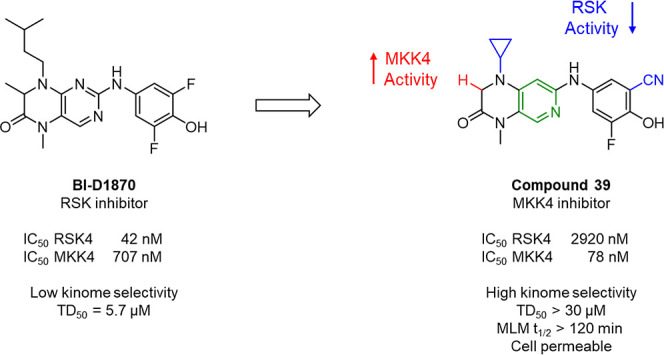

## Introduction

Liver diseases constitute a significant and escalating health concern, responsible for more than two million deaths annually on a global scale.^1^ The incidence of mortality attributable to liver diseases has escalated by 50% over the past three decades, and projections indicate that this figure is poised to double within the subsequent two decades.^2^ While acute liver failure is relatively rare, chronic liver disease—defined as a progressive, destructive process that lasts longer than six months and can lead to liver cirrhosis or liver cancer—accounts for 3.5% of the global total death rate with two million deaths annually (2015).^1^ Several reasons can lead to chronic liver disease. The major ones are alcohol abuse, viral diseases, and nonalcoholic fatty liver disease (NAFLD). For alcohol abuse and hepatitis viruses, treatments such as lifestyle changes, vaccinations, and antiviral drugs are available.^3,4^ However, nonalcoholic fatty liver disease (NAFLD) is a poorly understood condition and its significance for chronic liver disease is rising due to a lack of eligible therapies.^1^ NAFLD comprises two different conditions: Nonalcoholic fatty liver (NAFL), which is characterized by pathological fat enclosure in liver tissue, and nonalcoholic steatohepatitis (NASH), including fibrosis, cirrhosis, and hepatocellular carcinoma (HCC).^5^ Both are associated with genetic variations^6^ as well as obesity, diabetes and metabolic syndrome.^7^ NASH is one of the most important public health issues. The global prevalence not only tripled between 1975 and 2016 ^8^ but is even expected to increase. For example, in the US, obesity has been projected to reach a prevalence of 50% by 2030 ^9^ accompanied by an increase in NAFLD prevalence and mortality.^10^ This highlights the growing need to find appropriate therapies.

In 2013, Wuestefeld et al. identified mitogen-activated protein kinase kinase 4 (MKK4) in a genome-wide in vivo RNAi screen as a key enzyme for liver regeneration.^11^ A knockdown of MKK4 with short hairpin RNA (shRNA) resulted in reduced fibrosis development in damaged livers, a faster cell-cycle entry, and higher protection against Fas-induced apoptosis. According to the authors, MKK4 silencing leads to amplified activation of mitogen-activated protein kinase kinase 7 (MKK7), resulting in enhanced downstream phosphorylation of c-Jun N-terminal kinase 1 (JNK1). This, in turn, promotes phosphorylation of the ETS transcription factor ELK1 and activating transcription factor 2 (ATF2), thereby increasing both hepatocyte proliferation and robustness without inducing any signs of malignancy, as depicted in Figure 1. This study validated MKK4 as a promising target for small molecule drug design.

**Figure 1:**
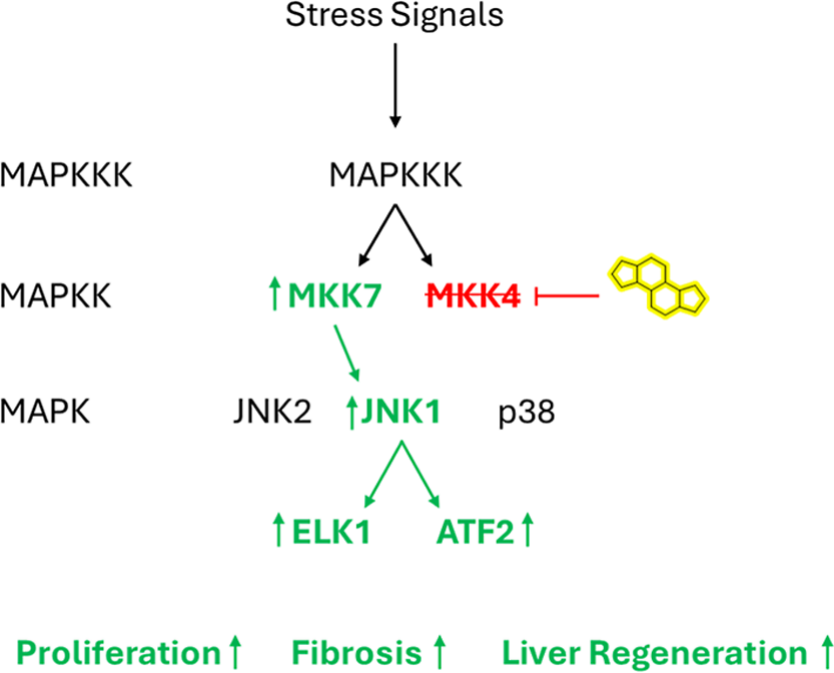
Overview of the MAPK/JNK signaling pathway after MKK4 knockdown. Adapted from Zwirner et al.^12^

Historically, only a few compounds with moderate MKK4 activity and poor selectivity were available (Figure 2). Bayers 4-phenyl-pyrimido­[4,5-*b*]­indole (**1**, IC_50_ < 1 μM),^13^ **Genistein** (**2**, IC_50_ = 400 nM)^14^ and **HWY336** (**3**, IC_50_ = 6 μM)^15^ are described as MKK4 inhibitors with moderate potency and low selectivity. However, in recent years, with an increasing understanding of the physiological and pathological roles of MKK4, there has been growing interest in developing highly active MKK4 inhibitors. **BSJ-04–122** (**4**, IC_50_ = 4 nM in LanthaScreen Eu binding assay) is a covalent dual MKK4/MKK7 inhibitor published by Jiang et al. that targets Cys246 in MKK4 and Cys261 in MKK7.^16^ In 2021, Kwong et al. reported a rational approach using 3-arylindazoles leading to compound **5** (IC_50_ = 83 nM) with modest selectivity.^17^ At the same time, Kloevekorn et al. published a target-hopping strategy from FDA-approved B-Raf^V600E^ Vemurafenib, resulting in various active and selective compounds based on the azaindole scaffold (e.g., **6**).^18^ Pfaffenrot et al. described pyrazolopyridine-based compound **7** (IC_50_ = 146 nM) with high kinome selectivity. In 2022, an updated study by Juchum et al. in which the series of Kloevekorn and Pfaffenrot et al. were further developed by the implementation of α-carboline as a scaffold (**8**), a new generation of these MKK4 inhibitors was presented. While keeping the potency of the inhibitors comparable to the azaindole and pyrazolopyridine scaffolds, the selectivity was markedly improved as the scaffold itself showed a strong selectivity-inducing effect.^19^ Darizmetinib (**9**, also referred to as HRX-215) recently entered clinical trials as a highly potent and selective MKK4 inhibitor aiming for liver regenerative therapy.^12,20^

**Figure 2:**
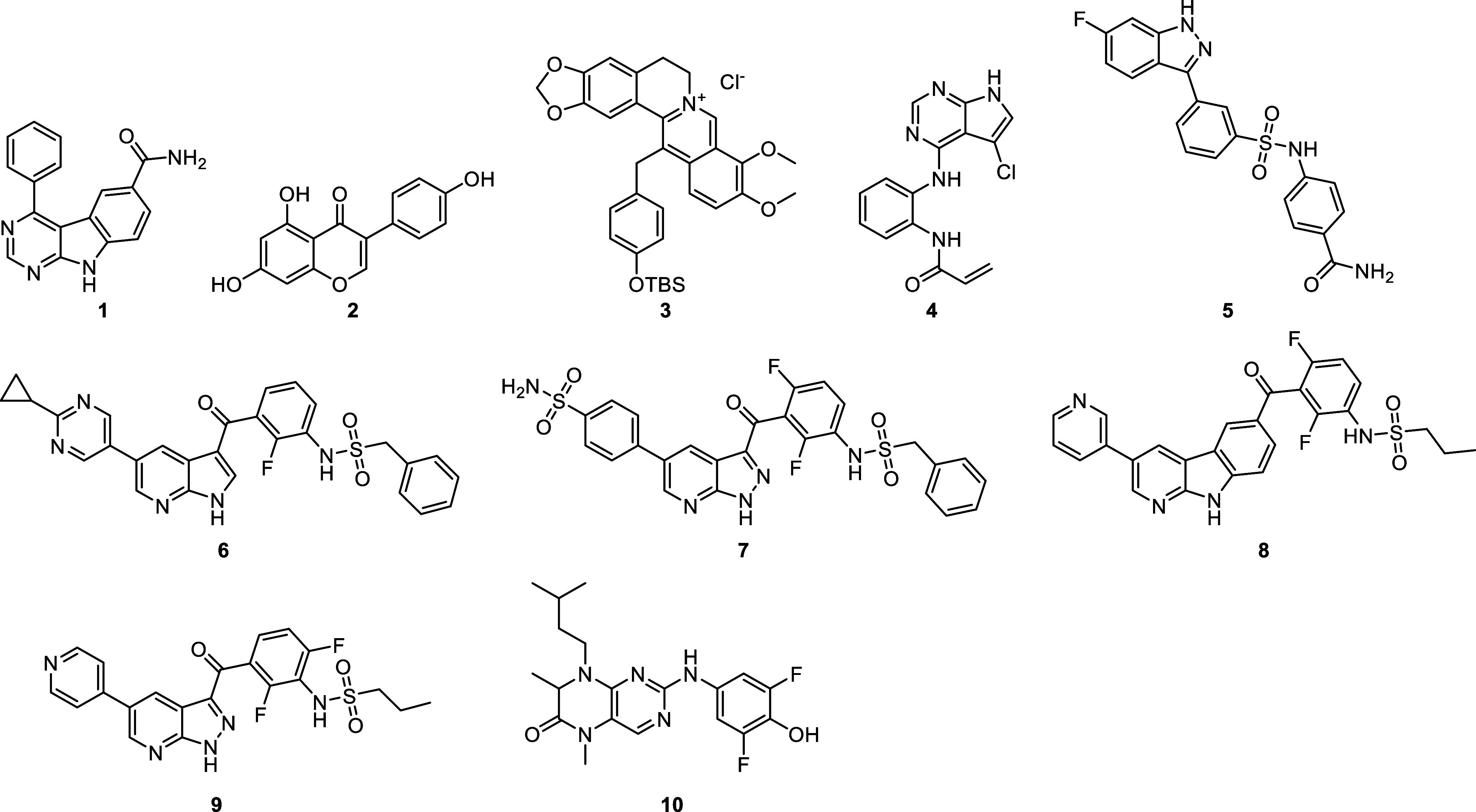
Recently reported MKK4 inhibitors.

In this study, a new class of MKK4-inhibitors was investigated. MKK7 and JNK1 were considered as the most relevant antitargets as the shRNA-mediated genetic suppression of either MKK7 or JNK1 abolished the pro-regenerative effect of genetic MKK4-suppression. In our previous studies,^18,19,21^ several scaffolds were identified as potent MKK4 inhibitors. Based on these scaffolds, a fluorescence polarization (FP) assay was developed.^22^ With this high throughput FP assay, **BI-D1870** (**10**), a reversible ribosomal S6 kinase (RSK) inhibitor^23^ with a broad activity profile,^24^ was identified as a moderate MKK4 inhibitor.^22^ Through chemical modifications, MKK4-activity and selectivity-driving moieties were identified. A strategic stepwise modification of **BI-D1870** resulted in a tremendous selectivity toward a multitude of MKK4 relevant off-targets, including the original target ribosomal protein S6 kinase A6 (RPS6KA6, also referred to as RSK4), mitogen-activated kinase kinase kinase 11 (MAP3K11), mitogen-activated kinase kinase kinase kinase 4 (MAP4K4), mitogen-activated kinase 6 (MKK6), JNK1 and MKK7.

## Results & Discussion

**BI-D1870** has been identified as a reversible ribosomal S6 kinase (RSK) inhibitor; however, its kinase selectivity is poor. Based on the moderate but promising inhibition potency against MKK4 (IC_50_ ∼ 200 nM), we considered this compound as an attractive screening hit for the development of selective MKK4 inhibitors. A stepwise strategic approach was followed to understand SAR and to design potent MKK4 inhibitors with a selectivity profile that allows their use as new therapeutics for liver regeneration.

First, we focused on chemical modifications that we believed would provide insights into the possible binding mode of our lead compound. Structure-based design was not feasible, as no reasonable docking approach could be established using available MKK4 crystal structures (PDBs: 3ALN, 3ALO, 3VUT), likely due to inconsistencies in the ATP-binding pocket or lack of cocrystallized ligands. Therefore, we pursued a medicinal chemistry strategy guided by systematic modification of the BI-D1870 scaffold.

Our approach was to explore whether potency against MKK4 could be improved while ultimately aiming to eliminate activity at relevant off-targets. To this end, we divided the molecule into five regions (Scheme 1), each selected for its potential to influence activity or selectivity:

**Scheme 1:**
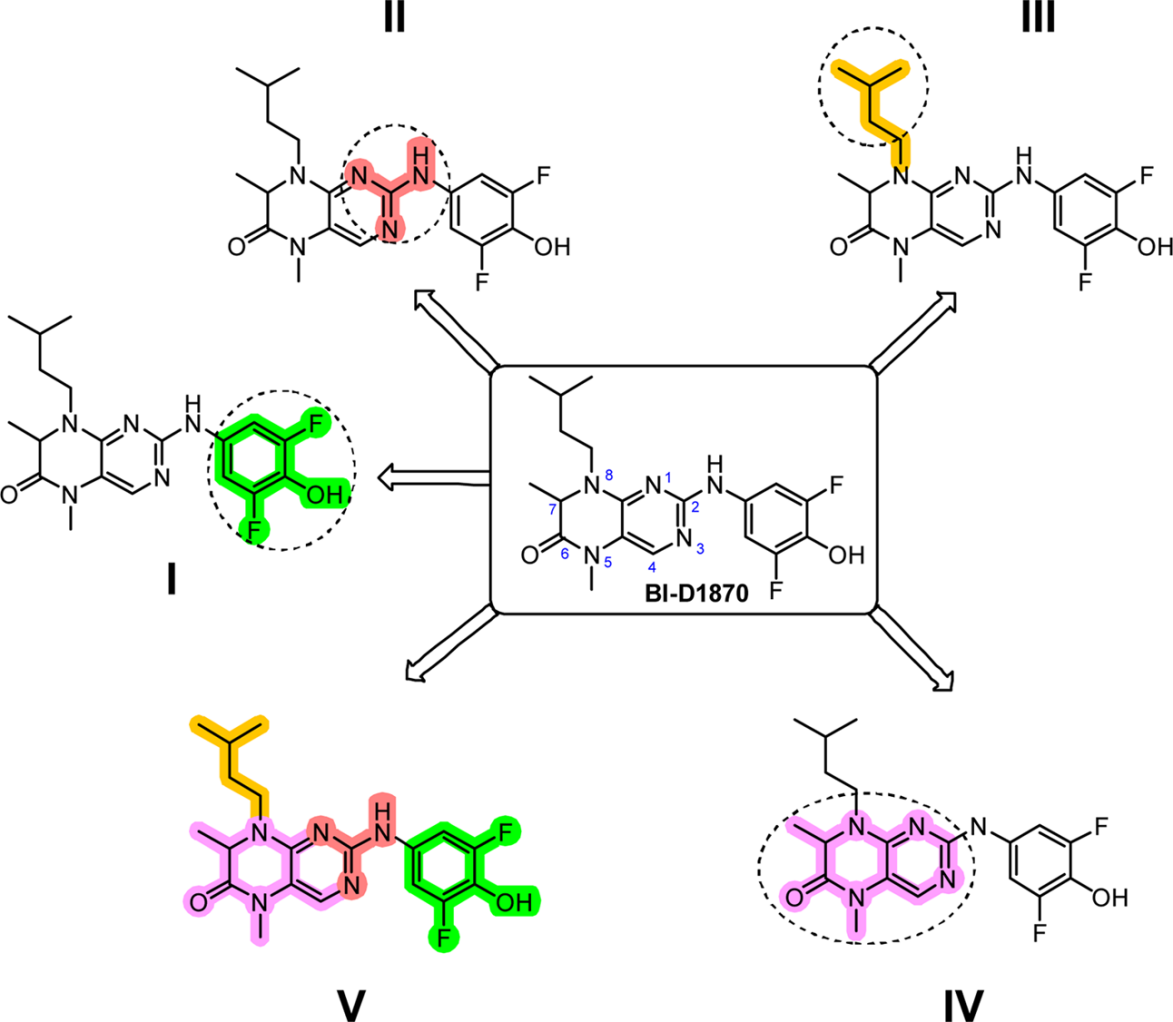
Intended Sites for Modification I to V of **BI-D1870** to Develop Selective and Potent MKK4 Inhibitors

**Region I:** Orientation within the binding pocket and identification of critical interactions;

**Region II:** The hinge-binding motif and conformational preferences associated with hinge engagement;

**Region III:** Lipophilic tuning via variation of aliphatic residues to enhance binding or reduce off-target effects;

**Region IV:** Modifications of the dihydropteridinone and pyrimidine cores to probe SAR and selectivity drivers;

**Region V:** Combination of beneficial features from Regions I–IV to achieve both potency and selectivity.

This iterative strategy aimed to deliver selective and potent MKK4 inhibitors, while also serving as an experimental foundation for deducing the compound’s binding mode through structure–activity insights.

**BI-D1870** (**10**) was discovered by Boehringer Ingelheim, inhibiting RSK1, RSK2, RSK3 and RSK4 in vitro with a half maximal inhibitory concentration (IC_50_) of 15–30 nM.^23^ In addition to the four isoforms of RSK, **BI-D1870** showed considerable off-target activity toward PLK1 (IC_50_ = 0.10 μM) which laid the foundation for Boehringer Ingelheim’s further development of dihydropteridinones as PLK1 inhibitors like **BI-2536** and **Volasertib**.^25,26^

To identify potential differences and similarities of RSK and PLK1 versus MKK4 early on, the corresponding available crystal structures of bound dihydropteridinones in RSK and PLK1 were reexamined to evaluate a possible binding mode for MKK4 which should be verified by experimental data. The binding mode of **BI-D1870** in RSK2 elucidated by Jain et al. and has the difluorophenol moiety oriented toward the DFG aspartate, as depicted in Figure 3A.^27^ However, it has been shown that dihydropteridinones are flipped in other kinases like PLK1 as shown in Figure 3B for Boehringer Ingelheim’s developed inhibitors BI-2536 ^28^ and Volasertib. **BI-D1870**’s wide activity profile was attributed to its ability to adapt to several conformations and orientations.^27^ In addition, the acidity of the phenol for **BI-D1870** was highlighted. In the available crystal structure, this shows up with an un-ionized hydroxyl group forming a hydrogen bond to the Asp211. The low p*K*_a_ (7.5)^29^ derived from the flanking fluorines, which also increase hydrophobic contacts in the partially hydrophobic pocket, are responsible for the high affinity to RSK2 due to the hydrogen bonding potential.^27^ In PLK1 the Asp does not seem to be relevant for the binding, due to the DFG conformation.

**Figure 3:**
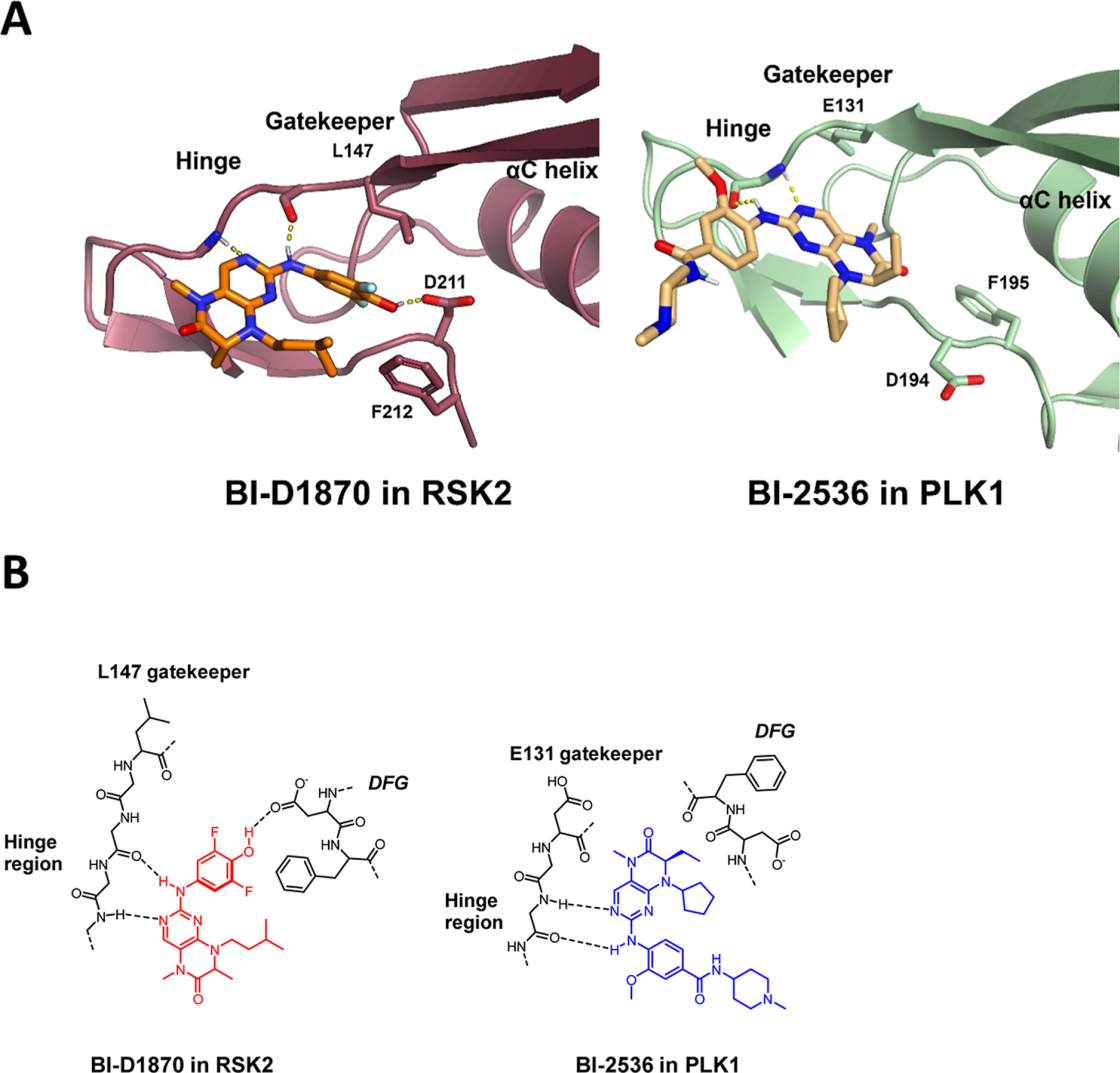
3D Binding modes of dihydropteridinone inhibitors BI-D1870 and BI-2536. (A) 3D Binding mode of BI-D1870 in RSK2 (left; PDB code5D9K) and BI-2536 in PLK1 (right; PDB code 2RKU). (B) 2D-binding mode of BI-D1870 in RSK2 (left) and BI-2536 in PLK1 (right) illustrating the kinase-dependent scaffold-flip.

### Initial SAR. Variation of the Difluorophenol Moiety

Initial structure–activity-relationship studies were focused on the difluorophenyl moiety as modifications thereof would give, besides possible interaction sites, first insights into the binding mode of **BI-D1870** in MKK4, whether a more PLK1 or RSK orientation is likely. A solvent-exposed orientation of the difluorophenyl moiety in MKK4, which would correspond to PLK1, would allow a broad variability of substitutions, whereas an orientation as seen in the RSK crystal structure would lead to steep SARs as was shown by Kloevekorn, Pfaffenrot, Kircher and Juchum et al. First, we investigated the difluorophenol moiety by systematic variation of the difluorophenyl pattern, omitting single and multiple substituents (**11**–**14**, see Table 1) and compared their potency to **BI-D1870** with a determined IC_50_ of 707 nM. A steep SAR was observed within this series, compound **11** showed no significant activity toward MKK4 with 9.38 μM, and the corresponding phenol (**12**) showed even less potency than compound **11** with an IC_50_ of 58.9 μM. Removal of the phenol moiety in the difluorophenyl derivative **13** resulted in a complete loss of activity above 100 μM, indicating the importance of this hydroxyl group. Omitting one fluorine led to a decreased MKK4 activity with compound **14**, showing an IC_50_ of 911 nM. The interplay of fluorine and the phenol moiety seems to play a crucial role in the SAR and according to the results, it was assumed that the p*K*_a_ of the phenol is the determining factor for the obtained SAR. Based on this assumption the phenol was substituted by benzoic acid (compound **15**), falsifying the hypothesis with an IC_50_ of 2792 nM, which is not close to **BI-D1870**. Another derivative was designed with the tetrafluoro analog of **BI-D1870**, in which the acidity of the phenol is increased by the additional fluorination. This derivative, compound **16**, finally showed an improved inhibitory effect on MKK4 with an IC_50_ of 73 nM. A strong decrease in MKK4 activity was also observed by the introduction of a methylene spacer between the core structure and the difluorophenyl moiety with compound **17**.

**Table 1:**
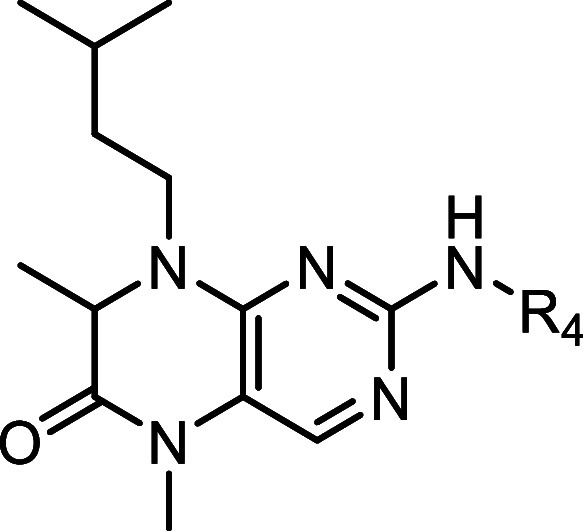

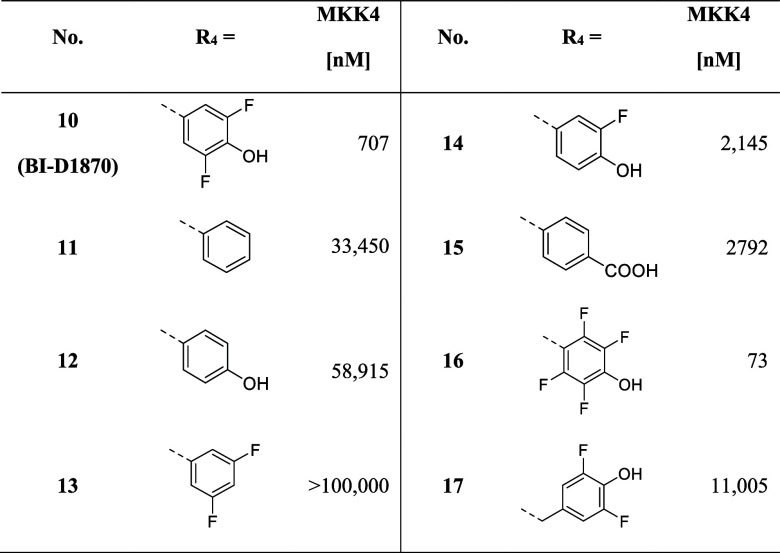
Potency of Dihydropteridinone Derivatives with Modified Difluorophenol Moiety^a^

a IC_50_ values were determined as the means of duplicates using commercial radiometric ^33^PanQinase assay by Reaction Biology.

The steep SAR strongly suggests that the phenyl group is not in a solvent-exposed orientation and in addition to the significance of the acidic phenol, an RSK-similar binding mode in MKK4 seems to be likely. To simulate such a binding mode a homology modeling of RSK2 (5D9K) and MKK4 (3ALO, being aware that sequence homology is low) was performed. Next, **BI-D1870** was docked into the homology model to evaluate possible binding interactions using Glide (Schroedinger). The resulting binding mode (Figure 4) was in accordance with our SAR findings. The phenol interacts with Asp247 and the hinge binding of NH/N-3 correlates with the typical hinge binding to Met181 and Glu179.

**Figure 4:**
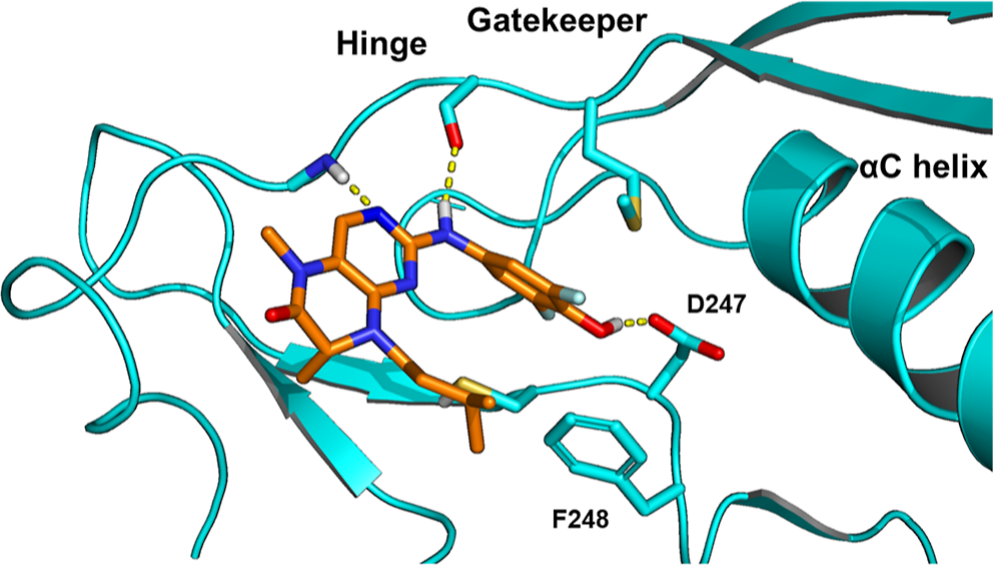
Homology model of surrogated **BI-D1870** binding mode in MKK4 (Uniprot: P45985) based on RSK2 (5D9K).

As previously described by Jain et al., an acidic phenol with p*K*_a_ around 7 is advantageous for binding to Asp211 in RSK because it is at least partially un-ionized and can form a hydrogen bond.^27^

### Identification of the Hinge Binding Motif

For both, **BI-D1870** in RSK2 and **BI-2536** in PLK1 the hinge binding motif is the typical aniline NH as donor and the pyrimidine N-3 as acceptor (Figure 3). For **BI-D1870** in MKK4, we wanted to suggest a binding mode, based on experimental results. The first step of this approach was to identify the relevant hinge binding motif, which is also important to suggest the conformation of the difluorophenyl moiety within MKK4. Within the structure, the 2-aminopyrimidine is the most conspicuous to be relevant for the hinge binding. To clarify which nitrogen acceptor is responsible for the hinge binding in MKK4 two derivatives were tested, compounds **18** and **19** (Table 2).

**Table 2:**
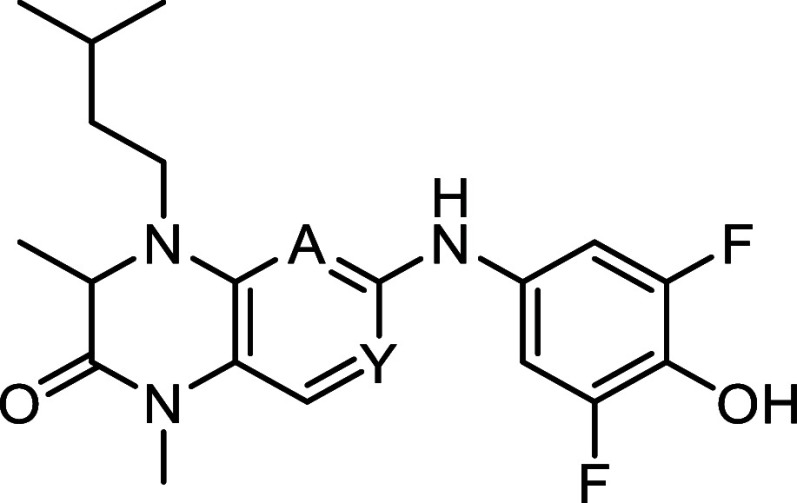
Variation of the Hinge-Binding Core Structure^a^

**No.**	**A =**	**Y =**	**R_5_ =**	**MKK4 [nM]**
**10**	N	N	H	707
**18**	CH	N	H	390
**19**	N	CH	H	>10^5^

a IC_50_ values were determined as the means of duplicates using commercial radiometric ^33^PanQinase assay by Reaction Biology.

The inactivity of derivative **19** and the potency of compound **18** (IC_50_ of 309 nM) suggest that the pyridine nitrogen of **18** (N-3) is the hinge binding functionality, just as observed in the RSK2 and PLK1 crystal structures.

### Variation of the Dihydropteridinone Core

#### Modifications of the Dihydropteridinone Core

The racemic α-methyl group in the C-7 position has been shown to be a selectivity factor for RSK with the (*S*)-enantiomer as the more potent enantiomer.^27^ We hypothesized that the methyl group was detrimental to binding to MKK4 due to its smaller binding cavity compared to RSK4 in this region of the active site. Compound **20** (Table 3) supports this idea, as it shows that removal of this methyl gives a compound with a 7-fold higher potency on MKK4 compared to **BI-D1870**, which contains a methyl group. To explore N-5 substitution SAR in the MKK4 binding site, we designed derivative **21**. A dramatic loss of activity was observed, indicating that increased substituent size in this position is poorly tolerated by MKK4.

**Table 3:**
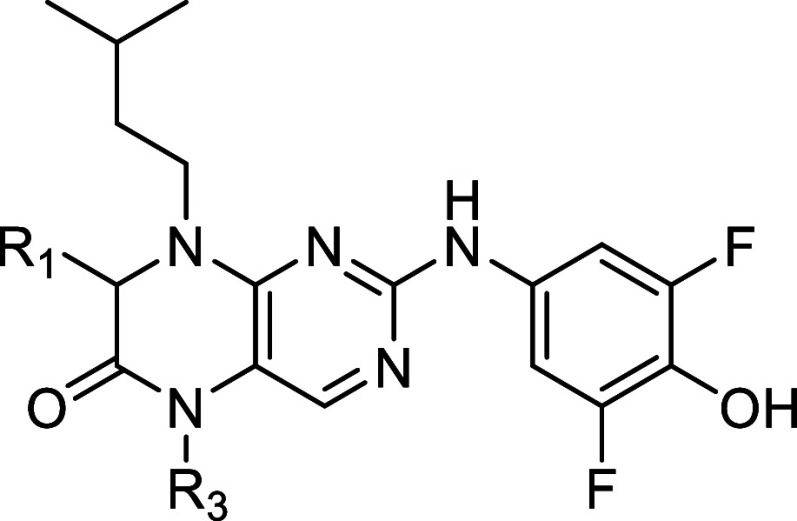
IC_50_ of Compounds with Modification on the Dihydropteridinone Ring^a^

**No.**	**R_1_ =**	**R_3_ =**	**MKK4 [nM]**
**10**	Me	Me	707
**20**	H	Me	53
**21**	Me	Et	9,535

a IC_50_ values were determined as the means of duplicates using commercial radiometric ^33^PanQinase assay by Reaction Biology.

After synthesizing compounds that inhibit MKK4 with IC_50_ values below 100 nM, we focused on achieving selectivity over RSKs. BI-D1870 inhibits the three RSK isoforms RSK2, RSK3 and RSK4 with similarly high potency. For selectivity testing, due to the high homology between these isoform, the inhibition potency of new structures toward RSK4 was determined.^30^ To investigate possible polar interactions of the amide in **BI-D1870**, the carbonyl was reduced which led to compound **22** (Table 4), with a significant loss of activity toward MKK4 (IC_50_ = 21.2 μM), indicating its essential role in the binding. Interestingly, the effect on RSK4 (IC_50_ = 669 nM) was not as significant as for MKK4. In our RSK-based MKK4 homology model, Lys187 is in proximity (∼2 Å) to carbonyl, which could be a possible interaction site, whereas in RSK2 no such interaction is observed.

**Table 4:**
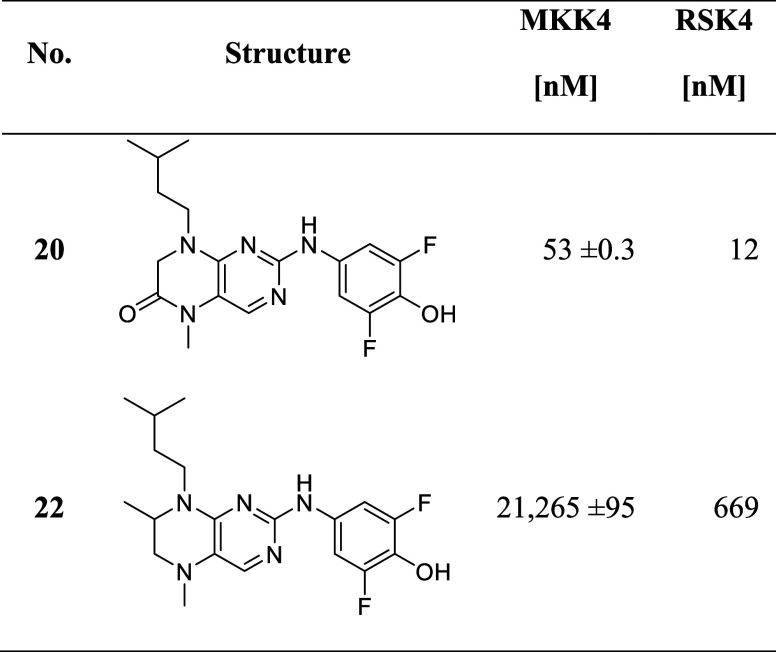
Variations of the Core Structure^a^

a IC_50_ values were determined as the means of duplicates using commercial radiometric ^33^PanQinase assay by Reaction Biology.

#### Variation of the N-8 Lipophilic Alkyl chain

We explored replacing the isopentyl side chain with different lipophilic moieties exploring size and shape and measured effects on MKK4 and RSK4 inhibition (Table 5). All derivatives that were investigated were combined with the previous findings from the demethylation approach of C-7 (compound **20**), which proved to be advantageous in terms of potency. At first, the isopentyl moiety was rigidized. The advantage of rigidization was seen in possibly more potent derivatives due to the entropic effects as well as in selectivity aspects due to less adaptable structures. Rigidizing the isopentyl moiety to its close analog, the cyclohexyl derivative **23,** led to a minor gain in MKK4 inhibition (IC_50_ = 45 nM), but unfortunately, also in a loss of selectivity against RSK4. In comparison, the aromatic counterpart, the phenyl analog (**24**), was less active with 189 nM. Next, the effect of the chain length was investigated with derivatives **25**–**30**. In this series, it was shown that a structural reduction of the alkyl length correlated with lower activity on all investigated kinases; therefore, no benefit was seen to further follow this strategy. Compound **28** was the most selective derivative (MKK4/RSK4 = 4.0) so far with a potency of 99 nM. In contrast, derivative **29** with a methylcyclopropyl group showed reduced activity on MKK4 (IC_50_ = 641 nM) and did not exhibit selectivity toward RSK (IC_50_ = 305 nM).

**Table 5:**
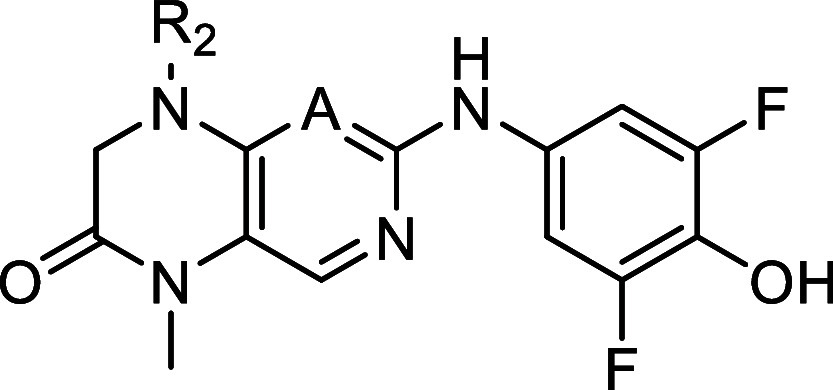

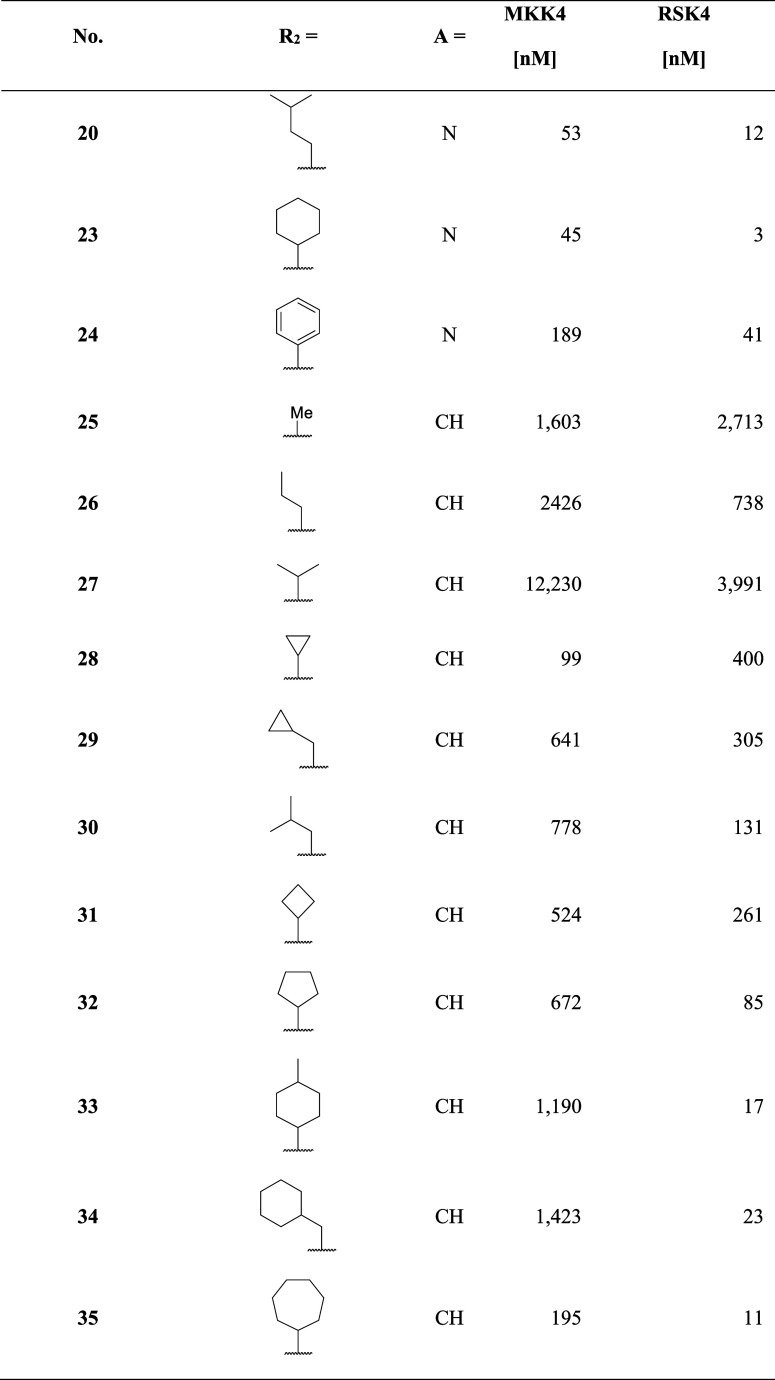
Potency of N-8 Modified Derivatives^a^

a IC_50_ values were determined as the means of duplicates using commercial radiometric ^33^PanQinase assay by Reaction Biology.

As compound **23** with its cyclohexyl moiety stood out in this series of derivatives, this structural class was further investigated regarding bulkiness and its effect on potency and selectivity. The series was designed from cycloheptyl down to cyclobutyl with compounds **31**–**35**. Among these derivatives a direct correlation between bulkiness and potency on RSK4 and MKK4 was observed, but for both kinases in a counteractive manner. Increasing bulkiness was positively correlating with potency on RSK. At the same time, we observed a trend that suggested that, in general, increased bulkiness in this position led to decreased potency against MKK4. Substitution of the cyclohexyl with a 4-methyl group (**33**) or introduction of a methylene group between N-8 and the cyclohexyl ring (**34**) led to a dramatic loss of MKK4 inhibition potency, so that the cyclohexyl and cyclopropyl substitutions were used for exploration of further structure modifications.

#### Variation of p*K*_a_ and Bulkiness of Difluorophenol Moiety

The modifications described in Tables 3–5 identified compounds with enhanced potency on MKK4 and improved selectivity over RSK4. We next turned to further optimization of the aromatic ring, bearing the phenol in the context of these improvements. The phenol moiety is crucial for binding to RSK4 and MKK4 if the phenol if in the right p*K*_a_ range. Therefore, closer investigation of this moiety, by altering the p*K*_a_ was imminent. 2,6-difluorobenzoic acid was introduced (**36**, Table 6) with a cpK_a_ of 2.4. This caused a significant drop of activity on both kinases MKK4 and RSK4, with RSK4 being more sensitive toward this modification. Combining, the activity-driving tetrafluorophenol from compound **16** with the cyclohexyl-1,4-dihydropyrido­[3,4-*b*]­pyrazin-3­(2*H*)-one scaffold (**23**) resulted in a highly active MKK4 inhibitor (**37**) (IC_50_ = 37 nM, cpK_a_ = 6.0, calculated with ChemDraw) with a 2-fold selectivity over RSK4 (IC_50_ = 84 nM). Substitution of one fluorine by a nitrile led to the 3-fluoro-2-hydroxybenzonitrile derivative **38**. The nitrile simultaneously increases the electron-withdrawing effect (cpK_a_ = 6.5) and the steric demand as well as the polarity. This modification led to an improvement in selectivity over RSK4 (RSK4/MKK4 = 6.7) with reasonable activity on MKK4 (IC_50_ = 268 nM). Merging the 3-fluoro-2-hydroxybenzonitrile with the selectivity-driving cyclopropyl derivative **28** yielded compound **39** with a slightly higher potency compared to compound **38** and an excellent selectivity over RSK4 with a factor of 37.4 (RSK4/MKK4). The substitution of the nitrile with the less electron-withdrawing trifluoromethyl (**41**, cpK_a_ = 6.9) led to a comparable MKK4 activity (IC_50_ = 35 nM) and still acceptable but lower selectivity over RSK4, compared to compound **39**. In analogy to compound **14**, a dehalogenation led to compound **40**. The effect of the dehalogenation was less prominent compared to **14** as MKK4 activity was still reasonable with 98 nM and selectivity was not completely lost. Replacement of the CF_3_ of compound **41** with a methyl ester provided compound **42**, which maintained MKK4 potency (IC_50_ = 73 nM) and retained 26-fold selectivity over RSK4. This suggests that the binding pocket of MKK4 can tolerate larger substituents than RSK4 in this position.

**Table 6:**
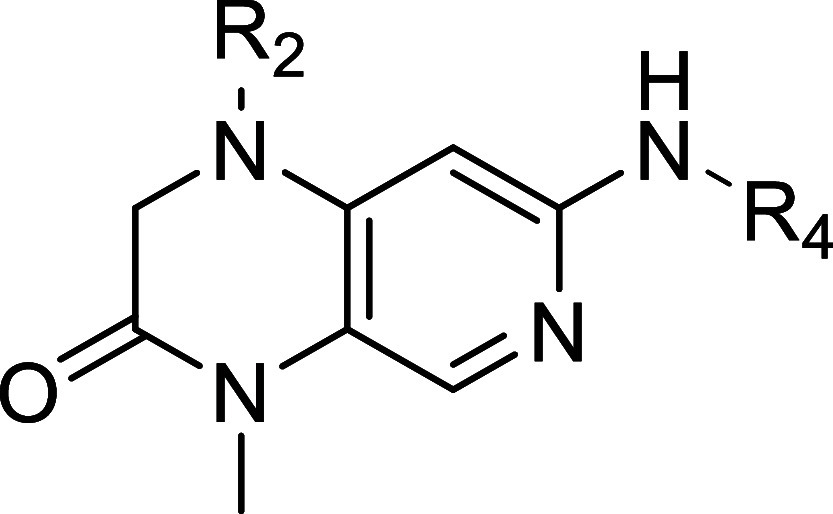

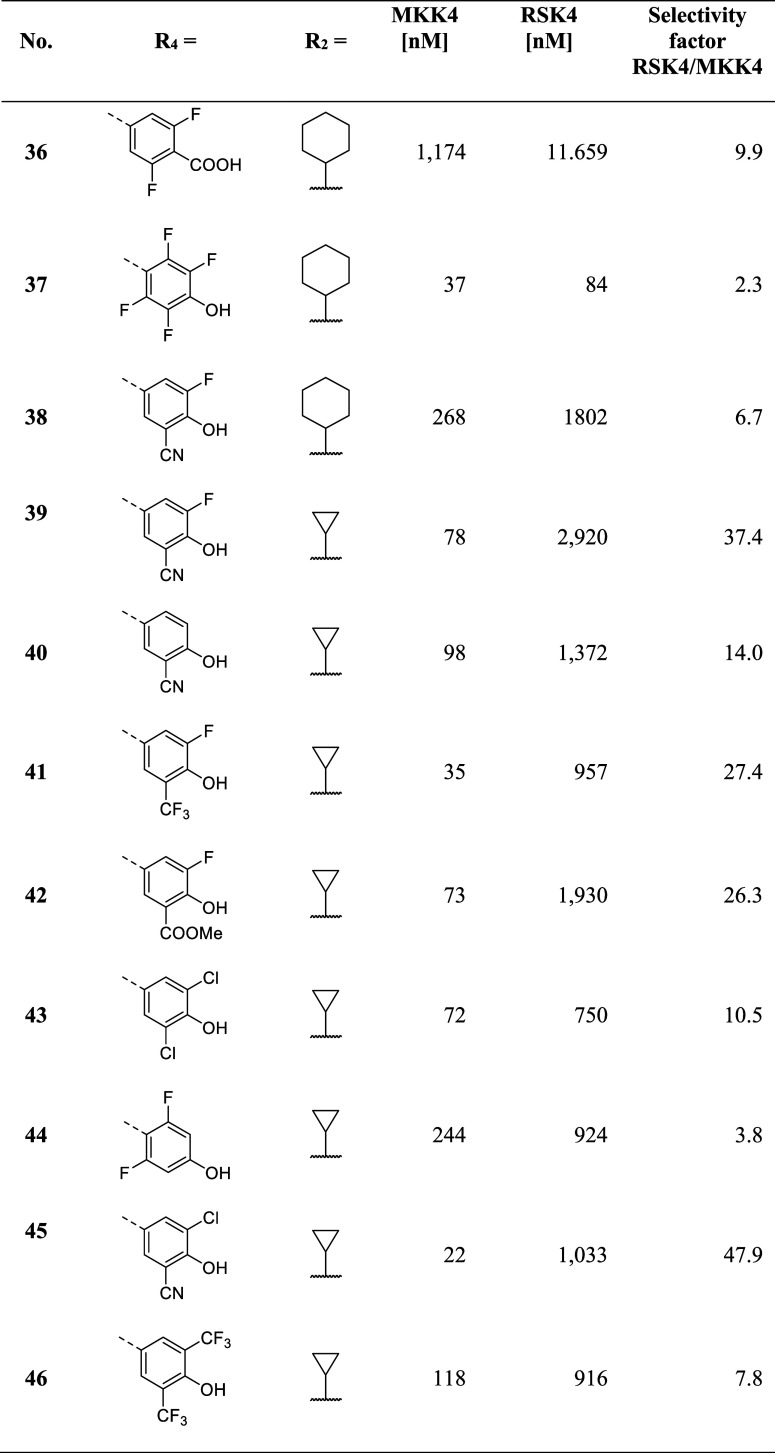
Modifications of the Phenol Moiety in Terms of p*K*_a_ Values and Steric Demand^a^

a IC_50_ values were determined as the means of duplicates using commercial radiometric ^33^PanQinase assay by Reaction Biology.

Compared to **28**, the substitution of the difluoro by a dichloro pattern (**43**) resulted in a slightly higher potency on both MKK4 and RSK4. Noteworthy was the change of the fluorine substitution pattern to 3,5-difluorophenol (compound **44**). Due to the *m*,*m*-positioning, accompanied by the decreasing cp*K*_a_ (8.6), there was a decreased MKK4 activity (IC_50_ = 244 nM) with moderate selectivity over RSK4 (IC_50_ = 924 nM). Substituting fluorine with chlorine resulted in compound **45** exhibiting potent inhibition (IC_50_ = 22 nM) and comparable selectivity with compound **39** (48-fold for compound **45** versus 37-fold for compound **39**). Given the good activity and selectivity profile of the trifluoromethyl substitution **41**, we also investigated whether increased electron withdrawal and steric demand on both sides of the phenol would result in greater MKK4 selectivity compared to RSK with compound **46**. This modification resulted in slightly reduced MKK4 activity (IC_50_ = 118 nM), while RSK4 remained largely unaffected compared to **41**.

Compounds **39** and **45** demonstrated promising activity toward MKK4 and exhibited selectivity over the original target RSK, suggesting that the observed effects are influenced by p*K*_a_ and steric factors.

#### Kinome Screen

To examine the selectivity of compound **39**, a screening against 350 wild-type kinases was conducted by Reaction Biology Kinase Panel Screening and compared to a screening of **BI-D1870** that was initially commissioned prior to the start of the SAR campaign. Both compounds were screened at two concentrations (1 μM and 10 μM; only 1 μM shown here). A representative extract of the data is presented in Table 7. The complete data set is provided in the Supporting Information. Residual activity was expressed as a percentage relative to the control. An overview of the selectivity screening of both compounds at 1 μM concentration is depicted in Figure 5.

**Table 7:**
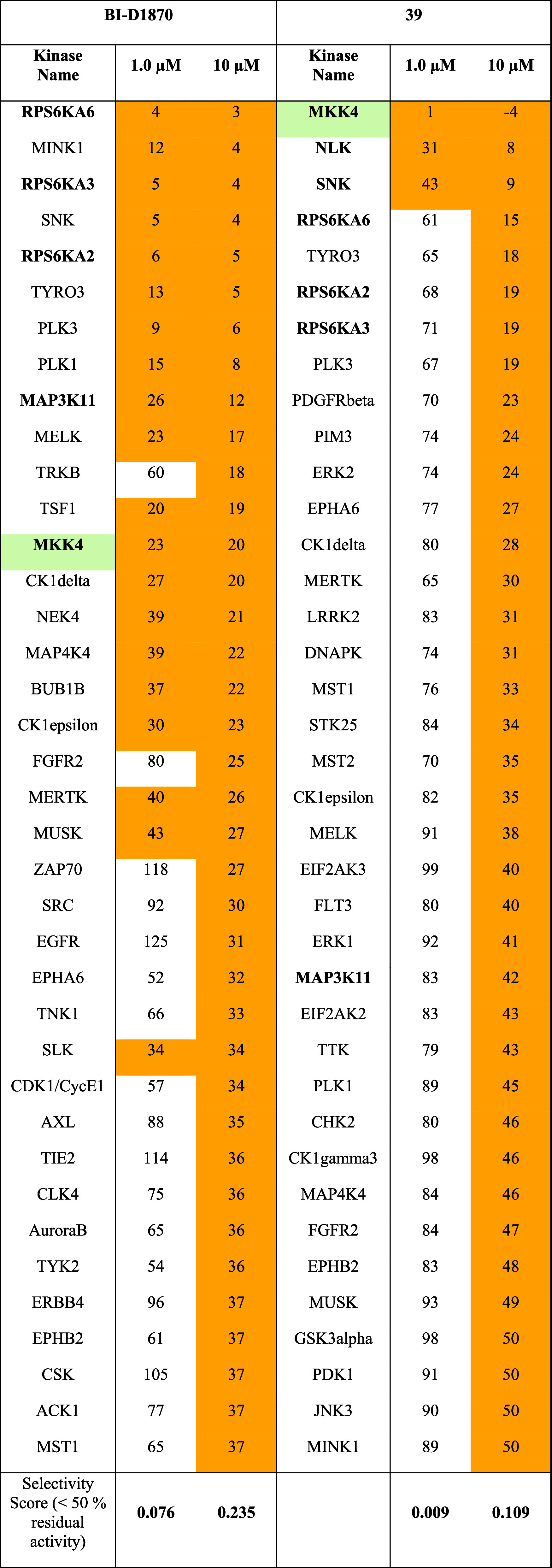
Representative Selection of Reaction Biology Kinome Screening Panel of Compound **39** and **BI-D1870** at Two Concentrations (1 μM and 10 μM) against 350 Wild-type Protein Kinases; Singlicate Measurement^a^

a Residual activities (% of control). Yellow: Residual activity <50%.

**Figure 5:**
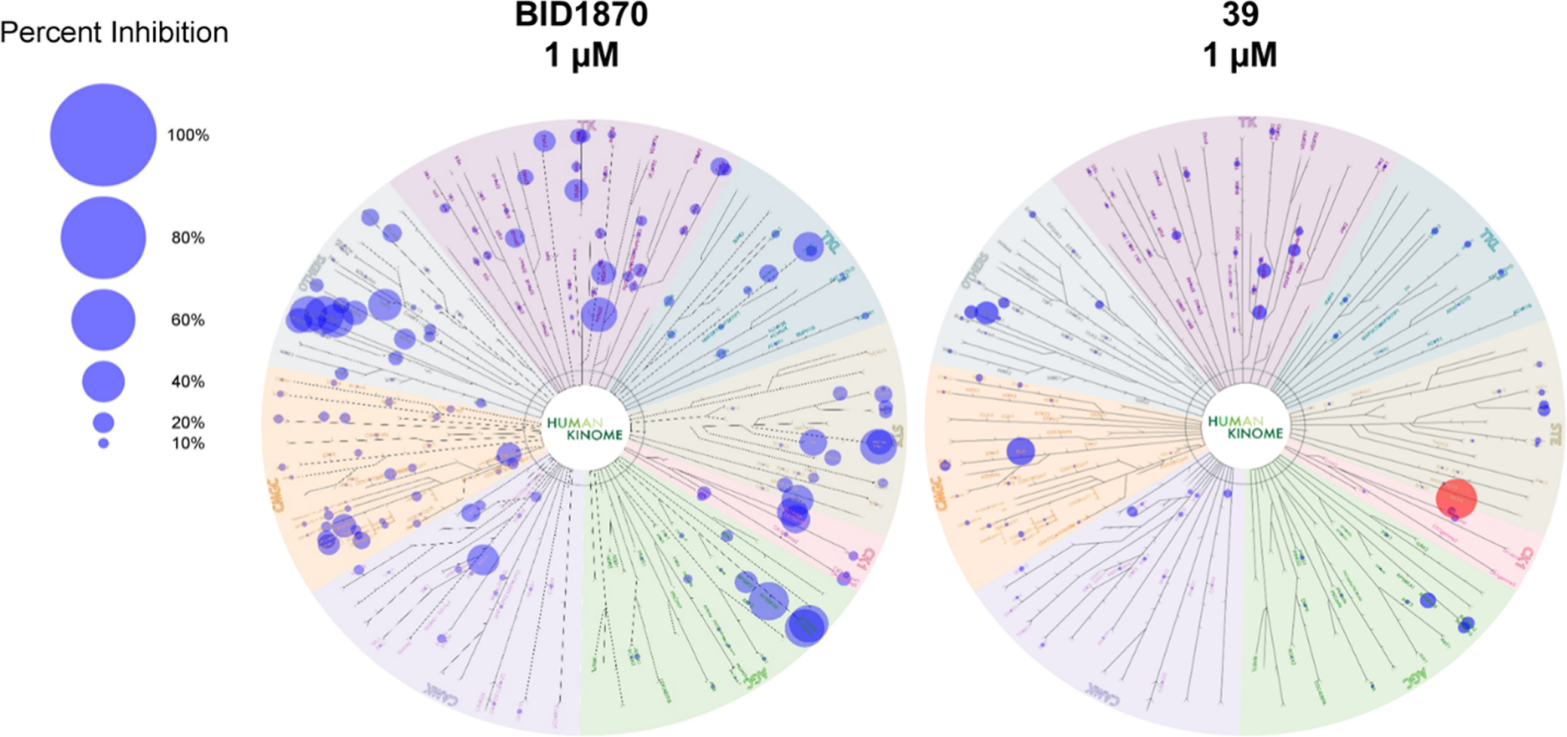
Kinome selectivity profile of **BI-D1870** and **39** at 1 μM measured in Reaction Biology kinome screening panel. Diameter of dots reflect % inhibition of 344 wildtype kinases (336 wildtype kinases for **BI-D1870**). Depicted are all protein kinases tested except atypical kinases (DNAPK, EEF2K, MTOR), CIT 1–450, CDK15/CycB1 and isoform PKMzeta. Red: MKK4. Figure created by Reaction Biology.

The **BI-D1870** compound has demonstrated to inhibit a broad spectrum of kinases, with 20 kinases exhibiting less than 50% activity at a 1 μM concentration. MKK4 exhibited residual activity at 23%, while the primary targets were the RSK family of kinases (RPS6KA2/3/6), for which the inhibitor was originally developed. Additionally, PLK3 (Polo-like kinase 3) and PLK2 (also referred to as SNK) were identified as targets in accordance with Boehringer Ingelheim’s PLK drug development campaign, which commenced with **BI-D1870**. At a compound concentration of 10 μM, the number of kinases exhibiting less than 50% residual activity increased to 80, thereby further underscoring the high level of activity observed on MINK1 (also designated as MAP4K6). The overall selectivity score according to Karaman et al.^31^ for **BI-D1870** in kinases with less than 50% residual activity at a concentration of 1 μM was 0.076, while at 10 μM, it was 0.235. Remarkably, compound **39** demonstrated the inhibition of only three kinases with less than 50% residual activity at a 1 μM concentration. One of which is MKK4, exhibiting 1% residual kinase activity. In addition, NLK (Nemo-like kinase) and PLK2 (SNK) were inhibited to a lesser extent, showing 82% and 43% residual activity at 1 μM, respectively. NLK was not significantly inhibited by the parent compound **BI-D1870**, indicating its emergence as a new off-target of compound **39**. We performed biochemical affinity assays to determine IC_50_ values for compounds **39** and **45** toward NLK and PLK2. Compound **39** demonstrated IC_50_ values of 977 nM for NLK and 1544 nM for SNK, whereas compound **45** exhibited significantly lower IC_50_ values of 609 nM (NLK) and 552 nM (SNK), indicating higher affinity for these off-targets. At a concentration of 10 μM, compound **39** inhibited fewer kinases overall, with only 38 showing less than 50% residual activity, representing a narrower off-target profile than observed for **BI-D1870** at the same concentration. Notably, **39** demonstrated a more pronounced inhibitory effect on MKK4 than the lower control, resulting in a residual activity of −4%. The selectivity score was 0.009 at 1 μM and 0.109 at 10 μM, indicating the potential for significantly increased selectivity and diminished off-target effects. This could lead to a reduction in cellular toxicity compared to **BI-D1870**, which demonstrated notable cytotoxic effects in cell-based assays—details of which will be discussed in the following section.

## Assessment of Cytotoxicity of **39** and **45**

**BI-D1870** has a relatively high cytotoxicity, which has been linked to its off-target activity.^32,33^ Therefore, **BI-D1870** and MKK4 inhibitors **39** and **45** were tested on their cytotoxicity in an XTT cell viability assay with Nras^G12 V^;Cdkn2a^ARF–/–^ HCC cells and NCI–H2030 NSCLC cells, which were exposed to the compounds in concentrations from 13.7 nM–90 μM (45.7 nM–30 μM for NCI–H2030) for 72 h. The HCC cell line was selected based on its high proliferative capacity, facilitating CETSA and toxicity studies, and its prior use in studying MKK4 inhibition effects in vitro. In line with earlier findings linking MKK4 inhibition to antiproliferative effects in HCC cells in a NASH-HCC context,^12^ this model serves as a surrogate to examine both selectivity and potential safety concerns. Cell viability was measured using XTT. While **BI-D1870** showed a TD_50_ of 5.7 μM, the toxic concentrations of both **39** and **45** were much higher, with no TD_50_ at the concentrations measured up to 30 μM (Figure 6). Similarly, in NCI–H2030 NSCLC cells, **BI-D1870** exhibited cell killing with an IC_50_ value of 10.9 μM (measured as average from triplicate, see Supporting Information Figure S77). In contrast, compounds **39** and **45** showed no measurable cytotoxicity, with IC_50_ values exceeding the upper tested concentration of 30 μM. These results further support that **BI-D1870** is more cytotoxic than **39** or **45**, consistent with our kinome screen findings demonstrating a significant reduction in off-target activity for the latter two compounds.

**Figure 6:**
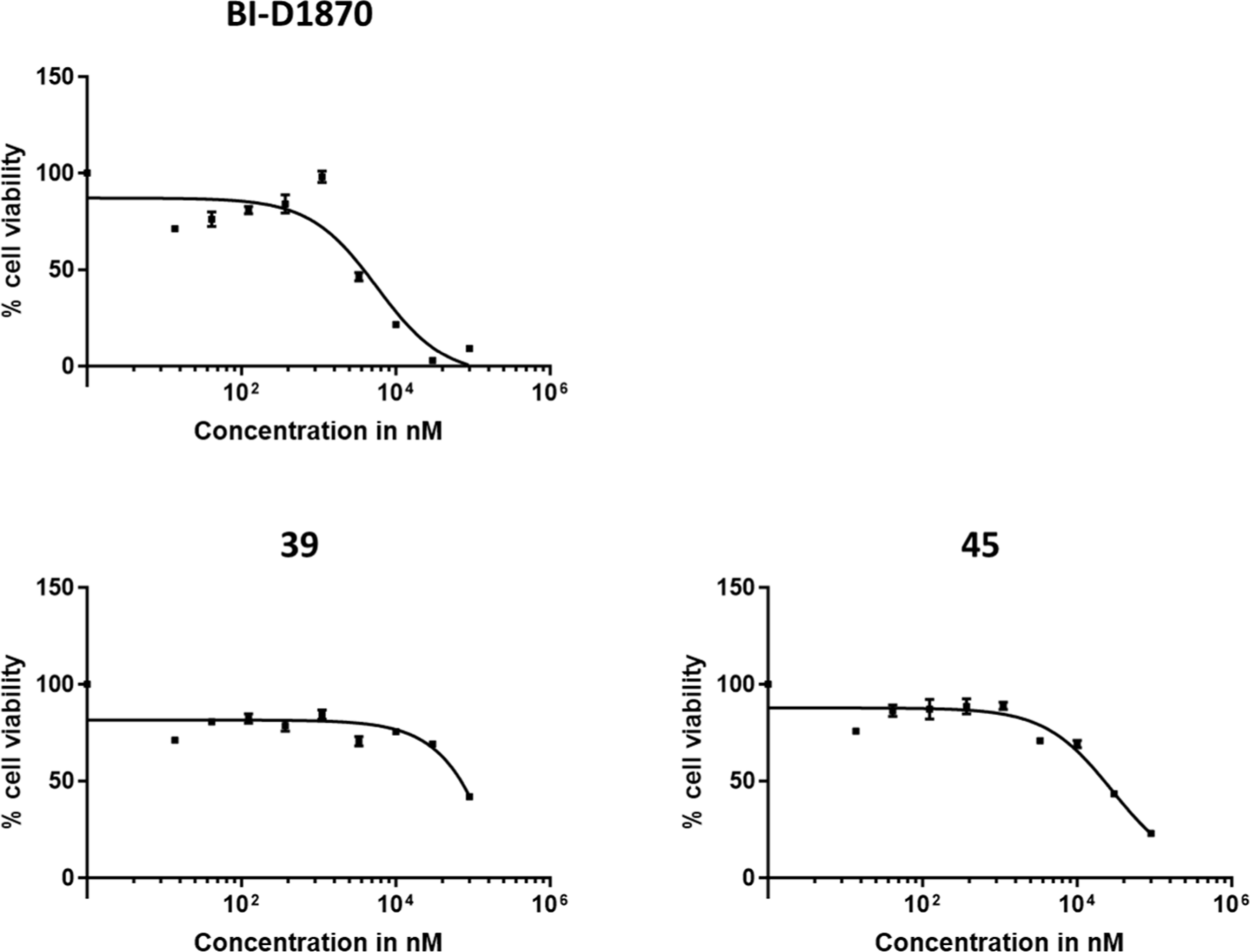
Cytotoxicity assay. **BI-D1870**, **39** and **45** were evaluated on murine Nras^G12 V^;Cdkn2a^ARF–/–^ HCC cells at concentrations ranging from 13.7 nM–90 μM (*n* = 3). Cell viability was determined by XTT assay.^34^ The images were generated using GraphPad Prism 10.1.1.

## Assessment of Metabolic Stability of **39** and **45**

To evaluate the metabolic stability of our compounds, **39** and **45** were incubated at 10 μM (Figure 7A) and 100 μM (Figure 7B) in mouse liver microsomes (MLM) for 2 h, with verapamil serving as a positive control. Both compounds showed no detectable metabolic degradation or metabolite formation, as assessed by LC–MS, indicating high stability in this in vitro system.

**Figure 7:**
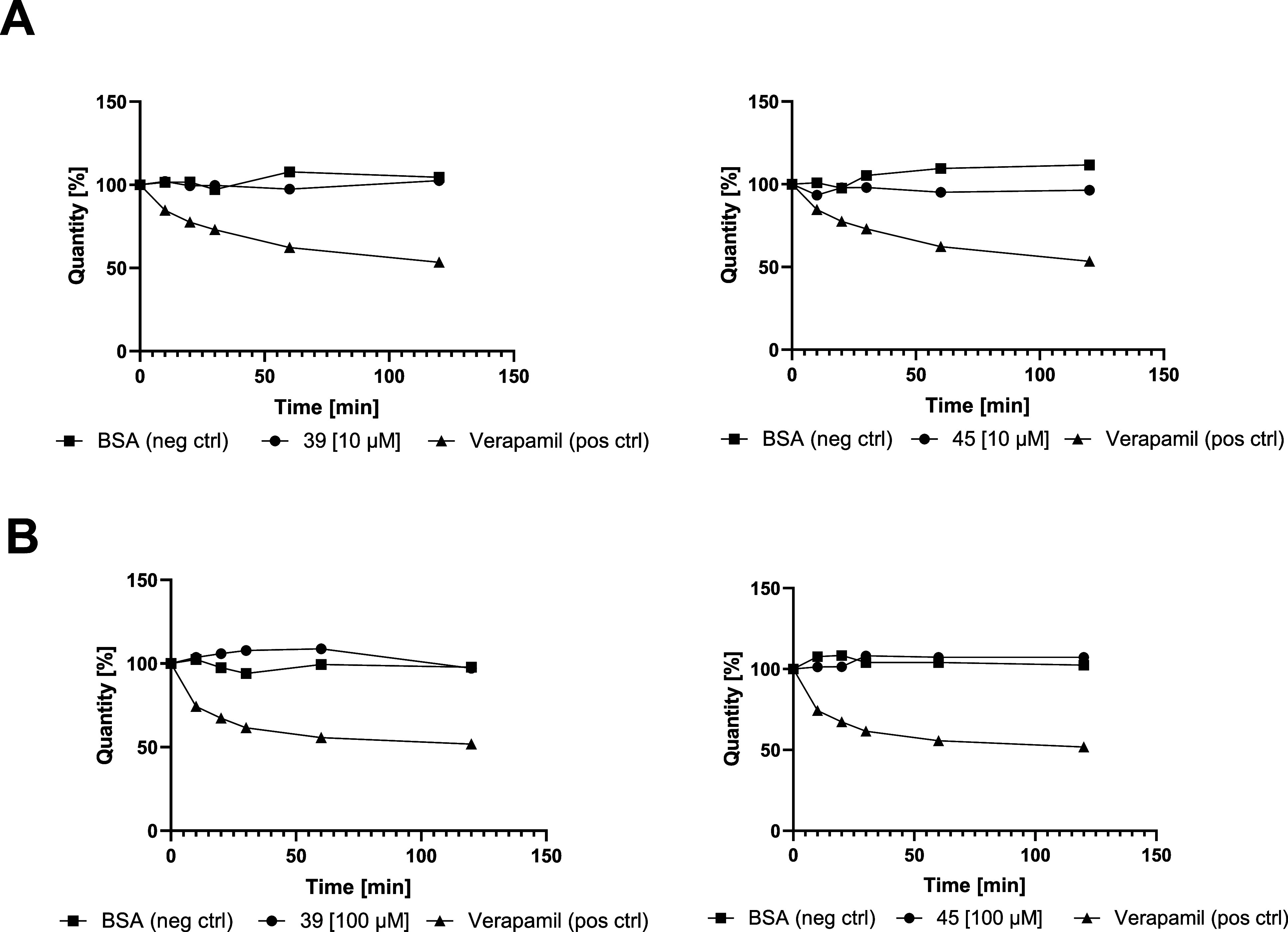
Metabolic stability in mouse liver microsomes. (A): compound **39** (left) or **45** (right) at 10 μM compound concentration; (B): compound **39** (left) or **45** (right) at 100 μM compound concentration; BSA: Bovine serum albumin as negative control; Verapamil as positive control. The images were generated using GraphPad Prism 10.1.1.

### Target Engagement

To further assess compound–target interactions, we first performed a thermal shift assay (TSA) to evaluate the ability of our compounds to thermally stabilize MKK4 in vitro. All three compounds, **BI-D1870**, compound **39**, and compound **45**, induced a measurable thermal stabilization of MKK4, with average Δ*T*_m_ values of 7.0 °C, 8.0 °C, and 9.3 °C, respectively (Figure 8A). Notably, compounds **39** and **45** induced greater thermal shifts than the parental **BI-D1870**, consistent with their increased biochemical affinity for MKK4. These findings suggest stronger target engagement at the molecular level, providing a rationale for their enhanced potency and justifying further evaluation in cellular assays.

**Figure 8:**
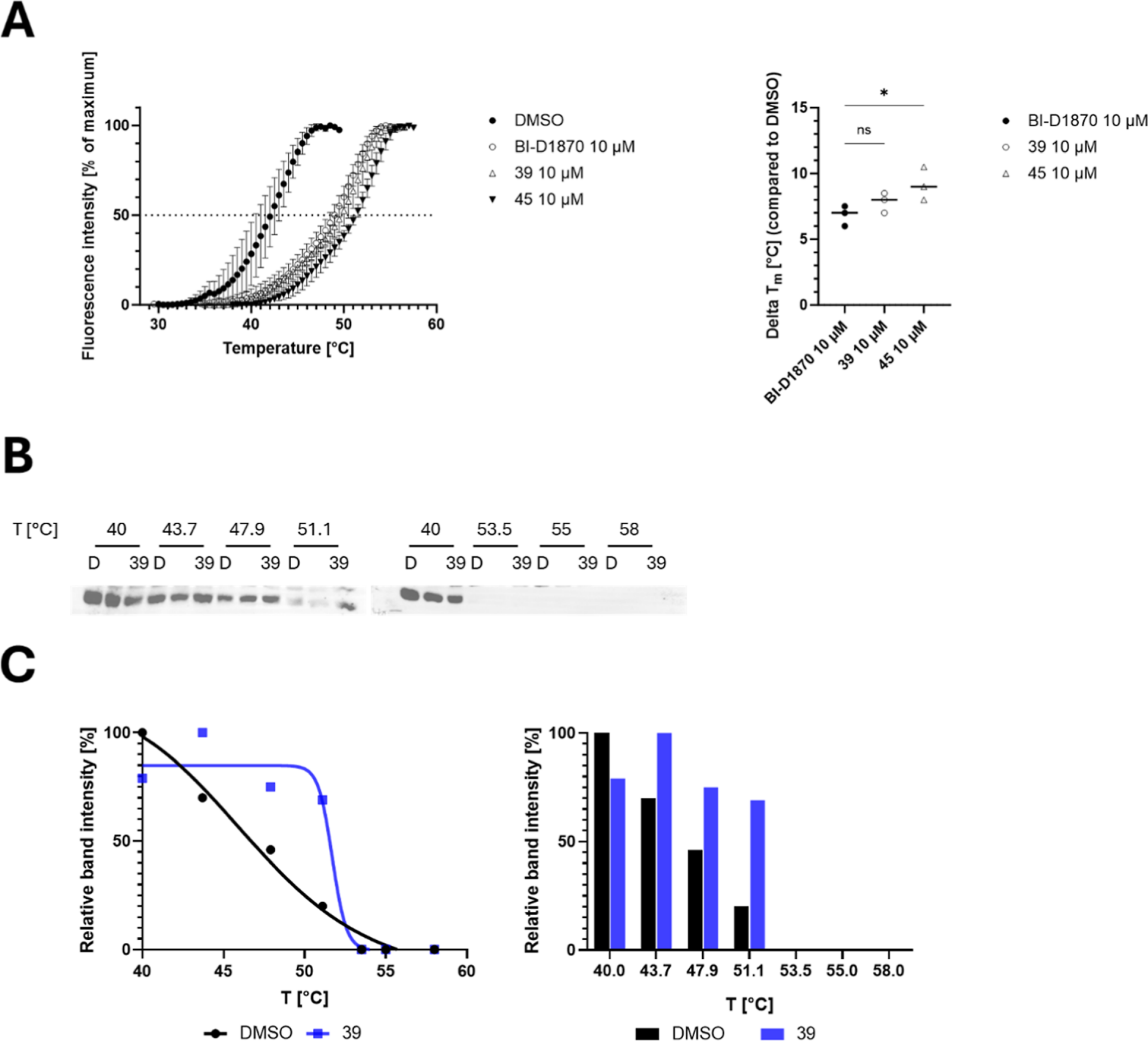
(A): Results of the thermal shift assay on MKK4 after treatment with 10 μM of **BI-D1870**, **39**, **45**, and DMSO. Values were averaged from triplicate determinations (*n* = 3). Results of cellular thermal shift assay on murine Nras^G12 V^; Cdkn2a^ARF–/–^ HCC cells after treatment with 10 μM compound **39** or DMSO for 1 h incubation time. (B): Western blot of temperature-treated cell lysates. D: DMSO negative control; (C): quantification based on Western blotting. Data was generated with ImageJ. The images were generated using GraphPad Prism 10.1.1.

To confirm intracellular target engagement, a cellular thermal shift assay (CETSA) was performed in murine Nras^G12 V^;Cdkn2a^ARF–/–^ HCC cells treated with 10 μM compound **39** for 1 h. This cell line was chosen based on prior use in studying MKK4 signaling and the feasibility of protein detection under CETSA conditions. A significant thermal stabilization of MKK4 was observed following treatment, indicating specific intracellular binding (Figure 8B,C).

### Chemistry

General synthesis of dihydropteridinones and 1,4-dihydropyrido­[3,4-*b*]­pyrazin-3­(2*H*)-one derivatives is depicted in Scheme 2. Synthesis of dihydropteridinones (A = N) was performed following literature with slight modifications.^35^ For the synthesis of 1,4-dihydropyrido­[3,4-*b*]­pyrazin-3­(2*H*)-ones (A = CH) the synthesis had to be modified. Bromoethylester **I** was substituted in an excess of at least three equivalents of amine **II**. For dihydropteridinones, secondary amine **III** was reacted with 2,4-dichloro-5-nitropyrimidine in a mixture of diethyl ether and water with potassium carbonate as a base not exceeding −10 °C in a nucleophilic aromatic substitution giving **V**. For 1,4-dihydropyrido­[3,4-*b*]­pyrazin-3­(2*H*)-ones, the less reactive 2,4-dichloro-5-nitropyridine had to be heated in acetonitrile (MeCN) to 50 °C using triethylamine (TEA) as base giving **V**. In a Béchamp-like reduction using elemental iron and acetic acid, a one-pot nitro-reduction and transesterification was performed, giving **VI**. Methylation was done by methyl iodide. Base was either sodium hydride or if R_1_ = H, a milder base like potassium carbonate was chosen due to witnessed methylation of the acidic α-carbonyl position in the presence of sodium hydride. For dihydropteridinones, **VII** was reacted with amine **VIII** using nucleophilic aromatic substitution reaction under slightly acidic conditions and high heat or Buchwald–Hartwig amination. Depending on the amine, different ligands and conditions were necessary. For 1,4-dihydropyrido­[3,4-*b*]­pyrazin-3­(2*H*)-ones, only Buchwald–Hartwig aminations were performed. Most of the time it was necessary to protect the phenol with benzyl or silyl ethers, that were cleaved after, using standard procedures according to the literature^36^ giving final compounds **IX**.

**Scheme 2:**
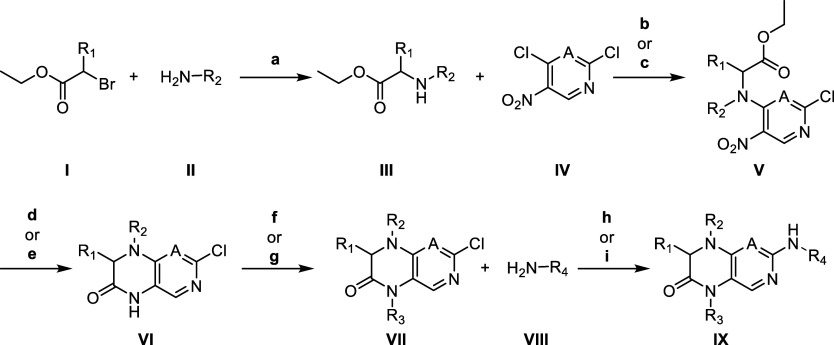
General Synthetic Route of Dihydropteridinone (A = N) and 1,4-dihydropyrido­[3,4-*b*]­pyrazin-3­(2H)-one (A = C)^a^

a (a) TEA, toluene or DCM, 0 °C – rt; (b) K_2_CO_3_, Et_2_O/H_2_O, −10 °C; (c) TEA, MeCN, 50 °C; (d) Fe, AcOH, 70–110 °C; (e) Zn, NH_4_OAc, 1,4-dioxane/H_2_O, 80 °C; (f) I-R_3_, NaH, DMF, −10 °C; (g) MeI, K_2_CO_3_, DMF, rt; (h) BrettPhos Pd G4 or XantPhos Pd G4 or XPhos Pd G4, K_2_CO_3_ or Cs_2_CO_3_ or KOtBu, 1,4-dioxane or 1,4-dioxane/tBuOH or toluene, 80 °C or reflux; (i) aq. HCl, 1,4-dioxane/H_2_O, reflux or MW irradiation.

In Scheme 3, the general procedure for commercially less accessible amines **XIV**, partly adapted from the literature,^37^ was conducted. 4,6-dichloropyridin-3-amine (**X**) reacted with 2-chloroacetyl chloride (**XI**) giving chloroacetamide **XII**. Amide-methylation was done with methyl iodide and potassium carbonate. Nucleophilic substitution of **XIII** with amine **XIV** was done in tetrahydrofuran (THF) with potassium carbonate as a base. In an intramolecular nucleophilic aromatic substitution reaction, secondary amine **XV** reacted to 1,4-dihydropyrido­[3,4-*b*]­pyrazin-3­(2*H*)-one **XVI**. Amination with amine **XVII** to **XVIII** was done according to Scheme 2 for 1,4-dihydropyrido­[3,4-*b*]­pyrazin-3­(2*H*)-ones. If required, a phenol-protecting group was cleaved after.

**Scheme 3:**
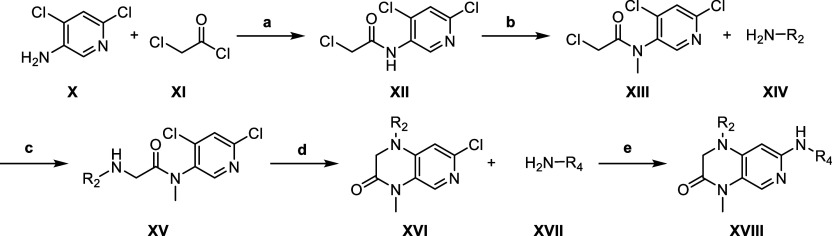
General Synthetic Route of 1,4-dihydropyrido­[3,4-*b*]­pyrazin-3­(2H)-one with Lower Expenses of Amines R_2_ ^a^

a (a) K_2_CO_3_, DCM/H_2_O, rt; (b) MeI, K_2_CO_3_, DMF, rt; (c) K_2_CO_3_, THF; (d) DIPEA, DMF, 100 °C; (e) BrettPhos Pd G4 or XantPhos Pd G4, 1,4-dioxane, reflux.

## Conclusion

We developed the first MKK4-selective dihydropyrido­[3,4-*b*]­pyrazin-3­(2H)-ones through a rational design campaign starting from the nonselective kinase inhibitor **BI-D1870**. Using a stepwise off-to-on-target strategy, we improved the scaffold’s potency against MKK4 while substantially reducing its broad off-target profile. Strategic modifications enabled us to establish structure–activity relationships (SAR) that suggested an RSK-like binding mode in MKK4. This, in turn, informed the development of a homology model used to guide subsequent compound optimization.

Compounds **39** and **45** emerged as the most promising inhibitors, with compound **39** demonstrating potent MKK4 inhibition (IC_50_ = 78 nM) and good selectivity over the parent target RSK4 (IC_50_ = 2920 nM). Kinome profiling of compound **39** yielded a selectivity score of 0.009 at 1 μM, markedly improved over **BI-D1870** (0.076 at 1 μM). In cytotoxicity assays, both compounds showed reduced toxicity compared to **BI-D1870**, likely due to improved kinome selectivity. CETSA analysis confirmed robust target engagement of MKK4, and compound **39** also demonstrated good metabolic stability in mouse liver microsomes.

Compared to previously reported MKK4 inhibitors such as natural product–derived compounds (e.g., 7,3′,4′-THIF) or earlier ATP-competitive scaffolds with limited selectivity and poor pharmacokinetics, our inhibitors offer a drug-like alternative with favorable in vitro properties. While **HRX215** has recently shown impressive in vivo efficacy in preclinical liver regeneration models and progressed into clinical trials,^12^ our compounds present a distinct chemical class that could complement ongoing efforts by serving as chemical probes or starting points for further optimization.

Similar to our recent report,^12^ future studies will focus on evaluating compound **39** in disease-relevant models of liver regeneration and cancer, as well as further improving selectivity and potency to support in vivo applications.

## Experimental Section

### Homology Modeling

Homology modeling was performed using the Schrödinger Drug Discovery suite for molecular modeling (version 2024.1). Protein–ligand complexes were prepared with the protein preparation workflow.^38^ Multiple sequence alignment (MUSCLE)^39^ and homology model of MKK4 (UniProt: P45985, kinase domain residues 98–367) were generated and optimized on the modeled frame of RSK2 (5D9K, chain A) using Prime.^40^

The figures were generated with PyMOL (The PyMOL Molecular Graphics System, Version 2.0 Schrödinger, LLC.).

### Cell Culture

Murine Nras^G12 V^;Cdkn2a^ARF–/–^ HCC cells, overexpressing an Nras mutant with a substitution of Glycin to Valin amino acid at position 12 and a deficiency of the Cdkn2a^ARF^ gene^41^ were used in the experiments. Nras^G12 V^;Cdkn2a^ARF–/–^ HCC cells were cultured in Dulbecco’s Modified Eagle Medium (DMEM) (Thermo Fischer Scientific) including 4.5 g/L d-glucose and l-glutamine. To prepare DMEM full medium, DMEM was additionally supplemented with 10% fetal bovine serum (PAN-Biotech), 1% sodium pyruvate (Thermo Fischer Scientific), 1% minimum essential medium nonessential amino acids (MEM NEAA) (Thermo Fischer Scientific) and 1% penicillin–streptomycin (Thermo Fischer Scientific). Cells were incubated at 37 °C with 5% CO_2_ in a humidified atmosphere. Cell lines were tested as mycoplasma negative.

### Cell Viability XTT Assay

For cell viability XTT assay, 750–1000 murine Nras^G12 V^;Cdkn2a^ARF–/–^ HCC cells or 3000–3500 human NCI–H2030 NSCLC cells were plated in a 96-well plate 24 h before treatment and stored at 37 °C in the incubator overnight. Ten mM inhibitor stock solutions of **BI-D1870**, **39** and **45** were serial diluted (1:3) to apply 9 concentrations between 13.7 nM–90 μM (45.7 nM–30 μM for NCI–H2030). Triplicates were used for each condition. DMSO was used as a control (100% cell viability) and wells with only medium but without cells were used as blank control. Cells were incubated for 72 h. Twenty-five μL XTT solution 1 mg/mL XTT + 2,5 μL/ml PMS in DMEM full medium was pipetted in each well and incubated for 2–4 h. The absorbance was measured at 450 nm in a Tecan Infinite M Plex plate reader. Data of XTT measurements were analyzed in Excel. Dose response curves including TD_50_ values were calculated and visualized in GraphPad Prism 10.1.1.

#### Metabolic Stability in Mouse Liver Microsomes

Pooled liver microsomes from mice (male) were purchased from Sekisui XenoTech, LLC, Kansas City, KS, USA.

Metabolic stability assays were performed in the presence of an NADPH-regenerating system consisting of 5 mM glucose-6-phosphate, 5 U/mL glucose-6-phosphate dehydrogenase, and 1 mM NADP^+^. Liver microsomes (20 mg/mL), NADPH-regenerating system, and 4 mM MgCl_2_·6H_2_O in 0.1 M TRIS–HCl-buffer (pH 7.4) were preincubated for 5 min at 37 °C and 750 rpm on a shaker. The reaction was started by adding the preheated compounds in DMSO at concentrations of 1 or 10 mM (**39**, **45**), respectively, resulting in respective final concentrations of 10 or 100 μM (**39**, **45**). The reaction was quenched at selected time points (0, 10, 20, 30, 60, and 120 min) by pipetting 100 μL of internal standard (ketoprofen) in acetonitrile at concentrations of 0.15 mM (**39**) and 0.1 mM (**45**), respectively. The samples were vortexed for 30 s and centrifuged (21,910 relative centrifugal force, 4 °C, 20 min). The supernatant was used directly for LC–MS analysis.

All compound incubations were conducted at least in triplicates. Additionally, negative control containing BSA (20 mg/mL) instead of liver microsomes and a positive control using Verapamil instead of compound were performed. A limit of 1% organic solvent during incubation was not exceeded.

Sample separation and detection were performed on an Alliance 2695 Separations Module HPLC system (Waters Corporation, Milford, MA, USA) equipped with a Phenomenex Kinetex 2.6 μm XB-C18 100 Å 50 × 3 mm column (Phenomenex Inc., Torrance, CA, USA) coupled to an Alliance 2996 Photodiode Array Detector and a MICROMASS QUATTRO micro API mass spectrometer (both Waters Corporation, Milford, MA, USA) using electrospray ionization in positive mode.

Mobile phase A: 90% water, 10% acetonitrile and additionally 0.1% formic acid (v/v), mobile phase B: 100% acetonitrile with additionally 0.1% formic acid (v/v). The gradient was set to 0–2.5 min 0% B, 2.5–10 min from 0 to 40% B, 10–12 min 40% B, 12–12.01 min from 40 to 0% B, 12.01–17 min 0% B (**39**) and 0–2.5 min 0% B, 2.5–10 min from 0 to 50% B, 10–12 min 50% B, 12–12.01 min from 50 to 0% B, 12.01–17 min 0% B (**45**) at a flow rate of 0.7 mL/min. Samples were maintained at 10 °C, the column temperature was set to 20 °C with an injection volume of 5 μL. Spray, cone, extractor, and RF lens voltages were at 4 kV, 30 V, 8 and 2 V, respectively. The source and desolvation temperatures were set to 120 and 350 °C, respectively, and the desolvation gas flow was set to 750 L/h.

Data analysis was conducted using MassLynx 4.1 software (Waters Corporation, Milford, MA, USA).

### Thermal Shift Assay

TSA was adapted from Krishna et al. 2013 and Deibler et al. 2019 and performed with 1 μg MKK4 protein (Reaction Biology). MKK4 protein was incubated with DMSO or 10 μM MKK4 inhibitor diluted from a 250 μM stock solution and 2.5x SYPRO Orange (Thermo Fischer) in TSA buffer containing 100 mM HEPES, pH 7.5 and 150 mM NaCl up to an end volume of 25 μL. The sample was pipetted and mixed in a 96-well PCR plate, incubated for 5 min, and centrifuged down before inserting the plate into a CFX Connect Real-Time PCR System. The installed Melt Curve range was set from 10.0 to 95 °C in increments of 0.5 °C for 10 s. Data were analyzed with CFX Maestro software as well as Excel to determine the difference in melting temperatures (Δ*T*_m_) compared to DMSO. Data was visualized in GraphPad Prism 10.1.1.

#### Cellular Thermal Shift Assay

Cellular target engagement between compound **39** and MKK4 in murine Nras^G12 V^;Cdkn2a^ARF–/–^ HCC cells was evaluated using a cellular thermal shift assay. Adherent murine Nras^G12 V^;Cdkn2a^ARF–/–^ HCC cells cells were seeded in three 15 cm diameter Petri dishes and grown until reaching 70–80% confluency. When the cells reached 70–80% confluency, cells were treated with either a 10 μM **39** solution in DMEM full medium or an equivalent amount of DMSO in DMEM full medium as a negative control. After incubating for 1 h at 37 °C in the incubator, cells were detached by trypsin and EDTA, combined in a 50 mL centrifuge tube, and centrifuged at 400 rcf for 5 min. The cell pellet was washed one time with phosphate-buffered saline (PBS). Each washed cell pellet was suspended in 1 mL of PBS containing protease and phosphatase inhibitors. The samples were exposed to temperatures ranging from 40 to 67 °C for 3 min. The temperatures used for the treatment gradient were 40, 43.7, 47.9, 51.1, 53.5, 55, 58, 62.3, 65.2, 67 °C. Afterward, cells were shock-frozen and lysed using lysis buffer (500 μL of 1 M TrisHCl, pH 7.5, 300 μL of 5 M NaCl solution, 50 μL of NP-40, 1 Complete Mini pill, fill up to 10 mL with bidistilled water). The cellular thermal stability of MKK4 was assessed by analyzing the lysates through Western blotting.

### Western Blotting Analysis

Total cell lysates were prepared in 4x laemmli sample loading buffer (200 mM TrisHCl pH 6.8, 8% (w/v) sodium dodecyl sulfate (SDS), 34.4% (v/v) Glycerol, 2.86 M β-Mercaptoethanol, 0.2% (w/v) Bromophenol blue). The samples were boiled for 10 min at 95 °C and subjected to a sodium dodecyl sulfate polyacrylamide gel electrophoresis (SDS-PAGE) using 10% SDS gels. Afterward proteins were transferred to polyvinylidene fluoride membranes (Merck Millipore) activated with 75% MeOH. The membranes were blocked using bovine serum albumin in Tris-buffered saline with 0.1% Tween 20 (TBS-T) buffer solution (2.5 g BSA in 50 mL TBS-T buffer) for 1 h at RT or overnight at 4 °C. The membranes were then probed with primary antibodies antitotal MKK4 (#9152, Cell Signaling Technology) and antivinculin (V9131, Sigma) for 1 h at RT or 1–2 days at 4 °C and then incubated with antirabbit (115–035–045, Dianova) or antimouse (115–035–062, Dianova) secondary antibodies at RT for 1 h. The membranes were detected with a ChemiDoc MP Imaging system (Bio-Rad).

### Radiometric ^33^PanQinase Assay

The IC_50_ values in ^33^PanQinase by Reaction Biology are determined through radiometric excitation of a scintillator that emits detectable light, allowing for quantification of the inhibitor’s binding. Specially coated ScintiPlates from PerkinElmer, which have a polystyrene-based scintillator layer, are used. The kinase (MKK4) transfers the radioactive terminal phosphate ([γ-^33^P]-ATP) to the substrate (JNK1). The radioactive substrate is in close proximity to the scintillator coating of the well, which leads to excitation of the scintillator when the ^33^P isotope decays and releases β-radiation. Inhibitors can reduce the activity of the kinase, resulting in less ^33^P being transferred to the substrate and less excitation of the scintillator. The [γ-^33^P]-ATP that is unbound does not cause any excitation because it is not close enough to the scintillator coating. This lack of proximity does not affect the readout. The assay conditions for the two most relevant kinases for this thesis, MKK4 and RPS6KA6 (also known as RSK4), which served as the testing kinases for the four RSK isoforms, are discussed in this section. MKK4 activity was measured at a protein concentration of 2.0 nM and an ATP concentration of 0.1 μM. The substrate of the enzyme was JNK1, whose kinase domain was rendered inactive by site-selective mutations K55R and K56R and was used at a concentration of 1.0 μg/50 μL. The conditions for RSK4 activity determination differed from MKK4, with a kinase concentration of 0.9 nM and an ATP concentration of 1.0 μM. The substrate GSK3(14–27) (glycogen synthase kinase 3) was used at a concentration of 2.0 μg/50 μL. For a more detailed explanation of the assay and the protocol, please refer to Reaction Biology.^42^

### Selectivity Profiling Assay

The compounds were tested in the Wild Type Kinase Panel from Reaction Biology based on their ^33^PanQinaseTM technology.^42^ For entire data column see Supporting Information.

### Chemistry

#### General

General. All commercially available reagents and solvents were used as received. Reactions sensitive to air or moisture were performed under an atmosphere of argon and/or in anhydrous solvents. Anhydrous solvents were purchased from Acros Organics (AcroSeal). Unless stated otherwise, extracts were dried over sodium sulfate prior to filtration. Thin layer chromatography was performed on TLC Silica Gel 60 F254 aluminum sheets provided by Merck, detection at λ = 254 and 366 nm. Flash chromatography was carried out on Interchim PuriFlash XS420 flash chromatography system and Grace Davison Davisil LC60A 20–45 mm silica. Purity of the compounds was determined by HPLC analysis on Agilent 1100 Series Liquid Chromatograph using a Phenomenex Luna C8 150 × 4.6 mm, 5 mm column with gradient elution (MeOH/0.01MKH_2_PO_4_ buffer, pH 2.3, flow rate 1.5 mL/min) and detection at λ = 230 and 254 nm. All final compounds were determined with >95% purity if not stated otherwise. Mass spectra were recorded on Advion DCMS interface (ESI voltage: 3.50 kV, capillary voltage: 187 V, source voltage: 44 V, capillary temperature: 250 °C, desolvation gas temperature: 250 °C, gas flow rate: 5L/min N_2_), elution of the spots with MeOH. High resolution mass spectra (ESI) of the final compounds were obtained from the Mass Spectrometry Department, Eberhard Karls Universitaet Tuebingen. NMR-spectra were measured on Bruker Avance 200 or 400 NMR spectrometers. The spectra were calibrated on the deuterated solvents and chemical shifts (d) are stated relative to tetramethylsilane in ppm. Prepared intermediate reagents that are listed in the Supporting Information are named R with consecutive numbering. p*K*_a_ values were calculated with ChemDraw or Advanced Chemistry Development (ACD/Laboratories) Software V11.02 (© 1994–2024 ACD/Laboratories).

Protein crystal structure and homology modeling images were created with the PyMOL Molecular Graphics System, Version 2.0 Schrödinger, LLC. or UCSF ChimeraX: Tools for structure building and analysis.^43^

### Synthesis Reactions

#### Synthesis of 2-((3,5-difluoro-4-hydroxyphenyl)­amino)-8-isopentyl-5,7-dimethyl-7,8-dihydropteridin-6­(5H)-one (BI-D1870) (**10**)

*N*-benzyl-3-methylbutan-1-amine, 1-bromo-3-methylbutane (40.6 g, 0.2241 mol), benzylamine (144.1 g, 1.3 mol) and distilled water (41 mL) were stirred at 90 °C for 24 h. After 24 h, sodium hydroxide (14 g) and brine were added. The aqueous layer was extracted three times with diethyl ether, the organic layers were combined and dried over sodium sulfate. After evaporation, the product was distilled at 112 °C (7 mbar), yielding 28.0 g (0.158 mol, 70%) of a colorless oil. ESI–MS *m*/*z*: [M + H]^+^ calcd for 178.2 (178.2); ^1^H NMR (400 MHz, DMSO-*d*_6_, δ): 7.34–7.18 (m, 5H), 3.67 (s, 2H), 2.48 (m, 2H), 1.60 (m, 1H), 1.36–1.28 (m, 2H), 0.84 (d, *J* = 6.6 Hz, 6H).

#### Ethyl *N*-benzyl-*N*-isopentylalaninate

*N*-benzyl-3-methylbutan-1-amine (28.0 g, 0.155 mol), ethyl-2-bromopropanoate (29.5 g, 0.166 mol), potassium carbonate (34.2 g, 0.248 mol), and DMF (250 mL) were heated to 110 °C in a dry flask under stirring at 110 °C for 3 h. After complete conversion the reaction was cooled to RT, filtered and the filtrate was evaporated. The residue was taken up in water, extracted 3x with diethyl ether and evaporated to dryness, yielding 42.6 g (0.154 mol, 99%) of brown oil. The crude product was used without further purification. ESI–MS *m*/*z*: [M + H]^+^ calcd for 278.4 (278.2)

#### Ethyl Isopentylalaninate

The crude ethyl *N*-benzyl-*N*-isopentylalaninate (40.0 g, 0.144 mol) from above was dissolved in EtOH (430 mL) in a reactor. Palladium on charcoal (4.0 g, 10%wt) and 37% aq. hydrochloric acid (14.4 mL) was added. The reactor was purged with Argon and subsequently filled with H_2_ up to 5 bar while stirring at room temperature until there was no loss in pressure for 30 h. The mixture was filtrated through a pad of Celite, evaporation of the filtrated methanol layer yielded a brown to yellow oil which was poured into diethyl ether, the mixture was ultrasonicated until a white precipitate formed. The product was filtrated off and washed with diethyl ether yielding 21.5 g (0.115 mol, 80%) of a white solid. ESI–MS *m*/*z*: [M + H]^+^ calcd for 188.2 (188.2); ^1^H NMR (400 MHz, DMSO-*d*_6_, δ): 4.28–4.15 (m, 2H), 4.06 (q, *J* = 7.0 Hz, 1H), 3.35 (s, 1H), 2.96–2.80 (m, 2H), 1.71–1.51 (m, 3H), 1.49 (d, *J* = 7.2 Hz, 3H), 1.23 (t, *J* = 7.1 Hz, 3H), 0.86 (d, *J* = 7.7 Hz, 6H). ^13^C NMR (101 MHz, DMSO-*d*_6_, δ): 169.9, 62.3, 54.7, 44.1, 34.7, 25.8, 22.6, 14.9, 14.4.

#### Ethyl *N*-(2-chloro-5-nitropyrimidin-4-yl)-*N*-isopentylalaninate

Ethyl isopentylalaninate (21.0 g, 0.112 mol) was dissolved in 200 mL water and 2,4-dichloro-5-nitropyrimidine (22.4 g, 0.116 mol) in 350 mL diethyl ether was added. The reaction mixture was cooled down to −10 °C and potassium carbonate (46.5 g, 0.336 mol) was added portion-wise. The mixture was stirred vigorously for 1 h at −5 °C, then the mixture slowly warmed to room temperature. After 3 h the aqueous layer was separated, and the organic layer was washed with water three times, dried over sodium sulfate, and evaporated to dryness yielding 32.6 g (0.095 mol, 84%) of an orange oil. The product was used without further purification.

#### 2-chloro-8-isopentyl-7-methyl-7,8-dihydropteridin-6­(5H)-one

Ethyl *N*-(2-chloro-5-nitropyrimidin-4-yl)-*N*-isopentylalaninate (32.5 g, 0.094 mol) was dissolved in 400 mL glacial acetic acid. The mixture was heated to 70 °C. The heat source was removed and Fe (27.6 g, 0.080 mol) was added. The temperature raised to 110 °C while stirring vigorously. After 20 min, the temperature decreased. When 70 °C was reached, the mixture was filtered hot and the filtrate was evaporated. The residue was mixed with 400 mL DCM and 42 mL of 37% aq. HCl. The phases were separated and the aqueous phase was extracted with DCM. The combined organic layers were washed with an aqueous ammonia solution (25%) and two times with brine. After drying over sodium sulfate, the solvent was evaporated. The obtained oil was sonicated in diethyl ether and a white-brown solid formed, which was filtered and evaporated to dryness yielding 13.42 g (0.047 mol, 53%). HPLC-DAD: 254 nm: 99.6%, 230 nm: 98.9%; ESI–MS *m*/*z*: [M – H]^−^ calcd for 267.2 (267.1.); Mp: 178 °C; ^1^H NMR (400 MHz, DMSO-*d*_6_, δ): 10.76 (s, 1H), 7.55 (s, 1H), 4.24 (q, *J* = 6.5 Hz, 1H), 3.91–3.78 (m, 1H), 3.22–3.10 (m, 1H), 1.61–1.39 (m, 3H), 1.35 (d, *J* = 6.7 Hz, 3H), 0.99–0.82 (m, 6H). ^13^C NMR (101 MHz, DMSO-*d*_6_, δ): 165.2, 152.3, 150.8, 137.7, 118.8, 55.9, 42.7, 39.5, 35.1, 25.5, 22.5, 22.3, 17.6.

#### 2-chloro-8-isopentyl-5,7-dimethyl-7,8-dihydropteridin-6­(5H)-one

2-chloro-8-isopentyl-7-methyl-7,8-dihydropteridin-6­(5H)-one (5.0 g, 18.6 mmol) and methyl iodide (10.6 g, 74.4 mmol) were dissolved in 51.8 mL DMA and cooled to −10 °C. Subsequently, 60% sodium hydride on paraffin (0.83 g, 20.8 mmol) was added while stirring for 2 h. After complete conversion the reaction was quenched with ice and aq. ammonia (25%). The reaction mixture was extracted three times with ethyl acetate. The combined organic layers were washed with brine, dried over sodium sulfate, loaded on Celite, evaporated, and purified using Flash chromatography yielding 4.24 g (15.0 mmol, 81%) of a white-brown solid. ESI–MS *m*/*z*: not detected [M + H]^+^ calcd for (283.1); ^1^H NMR (400 MHz, DMSO-*d*_6_, δ): 7.85 (s, 1H), 4.36 (q, *J* = 6.8 Hz, 1H), 3.93–3.82 (m, 1H), 3.21 (s, 3H), 3.20–3.12 (m, 1H), 1.60–1.40 (m, 3H), 1.33 (d, *J* = 6.8 Hz, 3H), 0.93–0.88 (m, 6H).

#### 2-((3,5-difluoro-4-hydroxyphenyl)­amino)-8-isopentyl-5,7-dimethyl-7,8-dihydropteridin-6­(5H)-one (BI-D1870) (**10**)

To 2-chloro-8-isopentyl-5,7-dimethyl-7,8-dihydropteridin-6­(5H)-one (200 mg, 0.71 mmol) was added 4-amino-2,6-difluorophenol hydrochloride (257 mg, 1.41 mmol), Cs_2_CO_3_ (461 mg, 1.41 mmol), anhydrous 1,4-dioxane (3 mL) and t-BuOH (0.75 mL). The mixture was gassed with Ar while sonicating. Xantphos Pd G3 (13 mg, 0.014 mmol) was added, the tube was sealed and irradiated at 160 °C for 1 h in a microwave reactor. The reaction was cooled to room temperature, diluted with DCM, filtered and evaporated. The residue was purified using Flash chromatography giving a brown solid (91.8 mg, 33%). HPLC-DAD: 254 nm: 95.3%, 230 nm: 96.9%; ESI–HRMS *m*/*z*: [M + H]+ calcd for 392.18926; found, 392.18987; ^1^H NMR (400 MHz, DMSO-*d*_6_, δ): 9.38 (d, *J* = 15.8 Hz, 1H), 9.15 (s, 1H), 7.80 (s, 1H), 7.49–7.37 (m, 2H), 4.25 (q, *J* = 6.7 Hz, 1H), 4.04–3.93 (m, 1H), 3.21 (s, 3H), 3.17–3.09 (m, 1H), 1.68–1.43 (m, 3H), 1.27 (d, *J* = 6.7 Hz, 3H), 0.94–0.88 (m, *J* = 7.7, 6.7 Hz, 6H). ^13^C NMR (101 MHz, DMSO-*d*_6_, δ): 163.8, 155.3, 152.22 (dd, *J* = 237.7, 9.0 Hz), 150.5, 138.6, 133.19 (t, *J* = 12.8 Hz), 126.6 (t, *J* = 16.6 Hz), 114.6, 101.4 (dd, *J* = 18.1, 9.2 Hz), 56.1, 56.0, 42.9, 35.6, 27.7, 25.7, 22.5, 22.4, 18.6, 17.0. IR (ATR) [cm^–1^]: 3275, 2953, 2869, 1661, 1517, 1430, 1220, 1140, 1009, 773.

#### Synthesis of 8-isopentyl-5,7-dimethyl-2-(phenylamino)-7,8-dihydropteridin-6­(5H)-one (**11**)

To a solution of 2-chloro-8-isopentyl-5,7-dimethyl-7,8-dihydropteridin-6­(5H)-one (100 mg, 0.35 mmol) in EtOH (2 mL), water (8 mL) and conc. HCl (0.1 mL) was added aniline (66 mg, 0.71 mmol). The reaction was refluxed for 16 h. After complete conversion, the reaction mixture was evaporated under reduced pressure and subsequently partitioned between NaHCO_3_ and MeOH/DCM (1:3). The aqueous layer was separated and extracted 3x with MeOH/DCM (1:3 v/v). The combined organic layers were combined, dried over sodium sulfate, evaporated under reduced pressure and purified using Flash chromatography, yielding a brown solid (117 mg, 97%). HPLC-DAD: 254 nm: 100%, 230 nm: 98.1%; ESI–HRMS *m*/*z*: [M + H]+ calcd for 340.21319; found, 340.21348; ^1^H NMR (400 MHz, DMSO-*d*_6_, δ): 9.09 (s, 1H), 7.80 (s, 1H), 7.74 (d, *J* = 8.1 Hz, 2H), 7.21 (t, *J* = 7.8 Hz, 2H), 6.87 (t, *J* = 7.2 Hz, 1H), 4.26 (q, *J* = 6.7 Hz, 1H), 4.07–3.97 (m, 1H), 3.23 (s, 3H), 3.18–3.08 (m, 1H), 1.68–1.46 (m, 3H), 1.28 (d, *J* = 6.7 Hz, 3H), 0.97–0.90 (m, 6H). ^13^C NMR (101 MHz, DMSO-*d*_6_, δ): 163.8, 155.7, 150.5, 141.3, 138.7, 128.3, 120.4, 118.0, 114.3, 56.1, 42.9, 35.6, 27.7, 25.8, 22.5, 22.5, 17.0. IR (ATR) [cm^–1^]: 3262, 3190, 2955, 2928, 2870, 1664, 1576, 1436, 1261, 1230, 1165.

##### Synthesis of 2-((4-hydroxyphenyl)­amino)-8-isopentyl-5,7-dimethyl-7,8-dihydropteridin-6­(5H)-one (**12**)

To a solution of 2-chloro-8-isopentyl-5,7-dimethyl-7,8-dihydropteridin-6­(5H)-one (100 mg, 0.35 mmol) in EtOH (1 mL), water (4 mL) and conc. HCl (0.1 mL) was added 4-aminophenol (77 mg, 0.71 mmol). The reaction was refluxed for 16 h. The reaction mixture was evaporated under reduced pressure and subsequently partitioned between sat. NaHCO_3_ solution and MeOH/DCM (1:3). The aqueous layer was separated and extracted 3x with MeOH/DCM (1:3). The combined organic layers were combined and dried over sodium sulfate and evaporated under reduced pressure. A brown-gray solid formed, which was triturated with diethyl ether and filtered, yielding a gray solid (52 mg, 41%). HPLC-DAD: 254 nm: 97.0%, 230 nm: could not be determined; ESI–HRMS *m*/*z*: [M + H]+ calcd for 356.20810; found, 356.20850; ^1^H NMR (400 MHz, DMSO-*d*_6_, δ): 8.92 (s, 1H), 8.75 (s, 1H), 7.74 (s, 1H), 7.47 (d, *J* = 8.8 Hz, 2H), 6.64 (d, *J* = 8.8 Hz, 2H), 4.22 (q, *J* = 6.6 Hz, 1H), 4.05–3.93 (m, 1H), 3.21 (s, 3H), 3.14–3.02 (m, 1H), 1.66–1.44 (m, 3H), 1.26 (d, *J* = 6.7 Hz, 3H), 0.97–0.89 (m, 6H). ^13^C NMR (101 MHz, DMSO-*d*_6_, δ): 164.2, 156.5, 152.0, 151.0, 139.4, 133.5, 120.5, 115.2, 114.2, 56.6, 43.2, 36.1, 28.2, 26.3, 23.0, 22.9, 17.3. IR (ATR) [cm-1]: 3275, 2954, 2870, 1647, 1607, 1433, 1256, 1214, 1164.

#### 2-((3,5-difluorophenyl)­amino)-8-isopentyl-5,7-dimethyl-7,8-dihydropteridin-6­(5H)-one (**13**)

To a solution of 2-chloro-8-isopentyl-5,7-dimethyl-7,8-dihydropteridin-6­(5H)-one (100 mg, 0.35 mmol) in EtOH (1 mL), water (4 mL) and conc. HCl (0.1 mL) was added 3,5-difluoroaniline (91 mg, 0.71 mmol). The reaction was refluxed for 16 h. The reaction mixture was evaporated under reduced pressure and subsequently partitioned between sat. NaHCO_3_ solution and MeOH/DCM (1:3). The aqueous layer was separated and extracted 3x with MeOH/DCM (1:3). The combined organic layers were combined and dried over sodium sulfate, evaporated under reduced pressure and purified using Flash chromatography, yielding a white solid (25 mg, 19%). HPLC-DAD: 254 nm: 98.4%, 230 nm: 98.0%; ESI–HRMS *m*/*z*: [M + H]+ calcd for 376.19434; found, 376.19499; ^1^H NMR (400 MHz, MeOD, δ): 7.76 (s, 1H), 7.35 (d, 2H), 6.47–6.37 (m, *J* = 9.1, 2.2 Hz, 1H), 4.28 (q, *J* = 6.8 Hz, 1H), 4.18–4.02 (m, 1H), 3.23–3.16 (m, 1H), 1.77–1.53 (m, 3H), 1.38 (d, *J* = 6.8 Hz, 3H), 1.00–0.91 (m, 6H). ^13^C NMR (101 MHz, DMSO-*d*_6_, δ): 157.9, 135.5 (dd, *J* = 241.1, 15.6 Hz), 147.4, 143.1, 135.5 (t, *J* = 13.7 Hz), 129.6, 107.2, 92.5 (dd, *J* = 20.4, 8.7 Hz), 87.0 (t, *J* = 26.4 Hz), 48.8, 35.4, 27.6, 19.1, 17.9, 13.5, 13.4, 8.0. IR (ATR) [cm-1]: 3272, 2954, 2932, 2870, 1669, 1598, 1576, 1427, 1250, 1147, 1108.

#### Synthesis of 2-((3-fluoro-4-hydroxyphenyl)­amino)-8-isopentyl-5,7-dimethyl-7,8-dihydropteridin-6­(5H)-one (**14**)

To 2-chloro-8-isopentyl-5,7-dimethyl-7,8-dihydropteridin-6­(5H)-one (85 mg, 0.30 mmol) was added 4-amino-2-fluorophenol (76 mg, 0.60 mmol), Cs_2_CO_3_ (196 mg, 0.60 mmol), anhydrous 1,4-dioxane (2 mL) and t-BuOH (0.5 mL). The mixture was flushed with argon while ultrasonicating. Xantphos Pd G3 (6 mg, 0.006 mmol) was added, the tube was sealed and microwave irradiated to 160 °C at 300 W for 1 h in a microwave reactor. The reaction was cooled to room temperature, diluted with DCM, filtered and evaporated. The residue was purified using Flash chromatography giving a beige solid (54 mg, 48%). HPLC-DAD: 254 nm: 97.7%, 230 nm: 97.4%; ESI–HRMS *m*/*z*: [M + H]^+^ calcd for 374.19868; found, 374.19898; ^1^H NMR (400 MHz, DMSO-*d*_6_, δ): 8.96 (s, 1H), 8.15 (s, 1H), 7.79 (s, 1H), 7.72–7.62 (m, 1H), 7.25–7.16 (m, 1H), 6.84–6.76 (m, 1H), 4.23 (q, *J* = 6.7 Hz, 1H), 4.04–3.93 (m, 1H), 3.21 (s, 1H), 3.15–3.06 (m, 1H), 1.68–1.43 (m, 3H), 1.26 (d, *J* = 6.7 Hz, 1H), 0.97–0.85 (m, 6H). 13C NMR (101 MHz, DMSO-*d*_6_, δ): 164.2, 163.6, 156.1, 152.1, 151.0, 149.7, 139.1, 138.8, 138.7, 134.2, 134.1, 117.8, 117.8, 114.8, 114.8, 114.6, 107.2, 107.0, 56.6, 43.3, 36.1, 28.2, 26.2, 22.9, 17.4. IR (ATR) [cm-1]: 3267, 2954, 1647, 1431, 1248, 1232, 1060, 972.

#### Synthesis of 4-((8-isopentyl-5,7-dimethyl-6-oxo-5,6,7,8-tetrahydropteridin-2-yl)­amino)­benzoic acid (**15**)

To a solution of 2-chloro-8-isopentyl-5,7-dimethyl-7,8-dihydropteridin-6­(5H)-one (100 mg, 0.35 mmol) in EtOH (2 mL), water (8 mL) and conc. HCl (0.2 mL) was added 4-aminobenzoic acid (97 mg, 0.71 mmol). The reaction was refluxed for 32 h. The product precipitated from the reaction mixture as a white solid. It was filtered and washed with water and diethyl ether. The residue was dissolved in methanol, loaded on silica gel and purified using Flash chromatography giving the titled product as a white solid (64 mg, 47%). HPLC-DAD: 254 nm: 100%, 230 nm: 100%; ESI–HRMS *m*/*z*: [M + H]+ calcd for 384.20302; found, 384.20360; ^1^H NMR (400 MHz, DMSO-*d*_6_, δ): 12.46 (s, 1H), 9.57 (s, 1H), 7.83 (dd, *J* = 21.4, 8.5 Hz, 5H), 4.29 (q, *J* = 6.6 Hz, 1H), 4.08–3.97 (m, 1H), 3.24 (s, 3H), 3.21–3.12 (m, 1H), 1.70–1.47 (m, 3H), 1.30 (d, *J* = 6.7 Hz, 3H), 1.00–0.90 (m, 6H). ^13^C NMR (101 MHz, DMSO-*d*_6_, δ): 167.2, 163.9, 155.1, 150.5, 145.6, 138.5, 130.2, 121.9, 116.7, 115.1, 56.2, 43.1, 35.5, 27.8, 25.9, 22.5, 22.5, 17.2. IR (ATR) [cm-1]: 3293, 2950, 2867, 2376, 2344, 1664, 1598, 1424, 1235.

#### 8-isopentyl-5,7-dimethyl-2-((2,3,5,6-tetrafluoro-4-hydroxyphenyl)­amino)-7,8-dihydropteridin-6­(5H)-one (**16**)

2-((4-(benzyloxy)-2,3,5,6-tetrafluorophenyl)­amino)-8-isopentyl-5,7-dimethyl-7,8-dihydropteridin-6­(5H)-one (230 mg, 0.44 mmol) was dissolved in EtOH (10 mL) and conc. HCl (0.25 mL). Pd/C (23 mg) was added. The mixture was stirred for 24 h in a hydrogen atmosphere. After full conversion, the reaction mixture was filtered over Celite, evaporated and purified using Flash chromatography yielding transparent brownish crystals (106 mg, 56%). HPLC-DAD: 254 nm: 99.3%, 230 nm: 98.6%; ESI–HRMS *m*/*z*: [M + H]+ calcd for 428.17041; found, 428.17059; 1H NMR (400 MHz, DMSO-*d*_6_, δ): 11.20 (s, 1H), 8.46 (s, 1H), 7.69 (s, 1H), 4.19 (q, *J* = 6.7 Hz, 1H), 3.70–3.58 (m, 1H), 3.18 (s, 3H), 3.01–2.91 (m, 1H), 1.43–1.27 (m, 3H), 1.24 (d, *J* = 6.8 Hz, 3H), 0.77 (d, *J* = 4.8 Hz, 6H). 13C NMR (101 MHz, DMSO-*d*_6_, δ): 163.69, 156.54, 150.65, 138.47, 114.69, 56.21, 42.86, 35.44, 27.73, 25.63, 22.17, 22.04, 17.30. IR (ATR) [cm-1]: 2953, 1669, 1663, 1570, 1476, 1436, 1430, 1327, 1244, 1011, 969.

#### 2-((3,5-difluoro-4-hydroxybenzyl)­amino)-8-isopentyl-5,7-dimethyl-7,8-dihydropteridin-6­(5H)-one (**17**)

Conc. HCl (0.7 mL) was added to 2-((3,5-difluoro-4-(methoxymethoxy)­benzyl)­amino)-8-isopentyl-5,7-dimethyl-7,8-dihydropteridin-6­(5H)-one (400 mg, 0.89 mmol) (see Supporting Information for detailed synthesis) in MeOH (12 mL). The mixture was stirred at room temperature for 3 h. It was extracted 3x with EtOAc. The combined organic layers were washed with water, brine, dried over sodium sulfate, evaporated and purified using Flash chromatography giving a brown solid (292 mg, 81%). HPLC-DAD: 254 nm: 97.3%, 230 nm: 97.1%; ESI–HRMS *m*/*z*: [M + H]+ calcd for 406.20491; found, 406.20544; ^1^H NMR (400 MHz, DMSO-*d*_6_, δ): 9.91 (s, 1H), 7.62 (s, 1H), 7.20 (s, 1H), 6.97–6.84 (m, 2H), 4.32 (ddd, *J* = 51.0, 15.7, 6.3 Hz, 2H), 4.16 (q, *J* = 6.7 Hz, 1H), 3.89 (s, 1H), 3.17 (d, *J* = 7.8 Hz, 3H), 3.05–2.95 (m, 1H), 1.52–1.30 (m, 3H), 1.23 (d, *J* = 6.7 Hz, 3H), 0.87–0.77 (m, 6H). 13C NMR (101 MHz, DMSO-*d*_6_, δ): 163.55, 157.56,152.10 (dd, *J* = 241.4, 7.1 Hz), 150.83, 137.59, 132.24–132.03 (m), 131.78 (t, *J* = 16.3 Hz), 113.27, 110.00 (dd, *J* = 14.8, 6.8 Hz), 56.06, 43.28, 35.49, 27.68, 25.51, 22.28, 16.97. IR (ATR) [cm-1]: 3356, 2953, 2868, 1661, 1574, 1519, 1011, 779.

#### Synthesis of 7-((3,5-difluoro-4-hydroxyphenyl)­amino)-1-isopentyl-2,4-dimethyl-1,4-dihydropyrido­[3,4-*b*]­pyrazin-3­(2H)-one (**18**)

Ethyl *N*-(2-chloro-5-nitropyridin-4-yl)-*N*-isopentylalaninate.

Ethyl isopentylalaninate (2.2 g, 11.7 mmol) was dissolved in water (20 mL). 2,4-dichloro-5-nitrpyridine (2.49 g, 12.9 mmol) in diethyl ether (40 mL) was added slowly. K_2_CO_3_ was added portionwise and the reaction was stirred for 16 h at rt. Upon completion, the phases were separated, the aqueous phase was extracted 3x with diethyl ether. The combined organic layers were dried over sodium sulfate, evaporated and purified using Flash chromatography giving the desired product as a crude orange oil that was used without further purification. ESI–MS *m*/*z*: [M + H]^+^ calcd for 344.3 (344.2).

#### 6-chloro-4-isopentyl-3,4-dihydropyrido­[2,3-*b*]­pyrazin-2­(1H)-one

Ethyl *N*-(2-chloro-5-nitropyridin-4-yl)-N-isopentylalaninate (5.0 g, 14.6 mmol) was dissolved in glacial acetic acid and heated to 70 °C. After removal of the heat source, 30 g of iron was added in portions. The temperature raised to 110 °C. The reaction mixture was cooled to 90 °C and stirred for 20 min at this temperature. The mixture was subsequently filtered and the filtrate was evaporated. The pH was set to 5–7 with NaHCO3 solution. The iron hydroxide was filtered off. The phases are separated. The organic phase was washed with water and brine, dried over MgSO4 and evaporated to dryness. The product was precipitated from ether yielding a dark brown oil that was purified using flash chromatography giving the desired product as a brown oil (2.14 g, 55%). ^1^H NMR (400 MHz, DMSO-*d*_6_, δ): 10.92 (s, 1H), 7.66 (s, 1H), 6.80 (s, 1H), 4.10 (q, *J* = 6.6 Hz, 1H), 3.58–3.47 (m, 1H), 3.24–3.11 (m, 1H), 1.67–1.55 (m, 1H), 1.51–1.37 (m, 2H), 1.24 (d, *J* = 6.7 Hz, 3H), 0.94–0.86 (m, 6H).

#### 7-chloro-1-isopentyl-2,4-dimethyl-1,4-dihydropyrido­[3,4-*b*]­pyrazin-3­(2H)-one

7-chloro-1-isopentyl-2-methyl-1,4-dihydropyrido­[3,4-*b*]­pyrazin-3­(2H)-one (2.14 g, 8.0 mmol) and methyl iodide (4.54 g, 32 mmol) were added to DMF (22 mL) and cooled to −10 °C. Subsequently, sodium hydride (60% on paraffin oil, 639 mg, 16 mmol) was added while stirring for 2 h. TLC confirmed conversion and the reaction was quenched with ice and aq. ammonia (25%). The mixture was extracted with EtOAc (3x). The combined organic layers were dried over sodium sulfate, filtered, evaporated and purified using Flash chromatography yielding a dark brown thick oil (1.76 g, 78%). ^1^H NMR (400 MHz, DMSO-*d*_6_, δ): 7.85 (s, 1H), 6.70 (s, 1H), 4.16 (q, *J* = 6.8 Hz, 1H), 3.52–3.43 (m, 1H), 3.28 (s, 3H), 3.17–3.08 (m, 1H), 1.65–1.54 (m, 1H), 1.48–1.40 (m, 2H), 1.14 (d, *J* = 6.8 Hz, 3H), 0.94–0.87 (m, 6H).

#### 7-((4-(benzyloxy)-3,5-difluorophenyl)­amino)-1-isopentyl-2,4-dimethyl-1,4-dihydropyrido­[3,4-*b*]­pyrazin-3­(2H)-one

7-chloro-1-isopentyl-2,4-dimethyl-1,4-dihydropyrido­[3,4-*b*]­pyrazin-3­(2H)-one (250 mg, 0.89 mmol), 4-(benzyloxy)-3,5-difluoroaniline (418 mg, 1.78 mmol), Cs_2_CO_3_ (580 mg, 1.79 mmol) and Pd G3 Xantphos (17 mg, 0.018 mmol) was added to a dry flask with a reflux condenser in an argon atmosphere. Anhydrous 1,4-dioxane/tBuOH (3:1, 19 mL) was added. The mixture was stirred to reflux for 3 h. The reaction was cooled to room temperature, diluted with water, extracted with EtOAc, washed with brine and dried over sodium sulfate. The residue was purified using Flash chromatography giving the titled product as a yellow solid (422 mg, 0.88 mmol, 99%). ESI–MS *m*/*z*: [M + H]^+^ calcd for 481.2 (481.2); ^1^H NMR (400 MHz, DMSO-*d*_6_, δ): 9.78 (s, 1H), 7.64 (s, 1H), 7.44–7.34 (m, 5H), 7.30–7.21 (m, 2H), 6.17 (s, 1H), 5.09 (d, *J* = 12.2 Hz, 2H), 4.24 (q, *J* = 6.7 Hz, 1H), 3.44–3.35 (m, 3H), 3.25 (s, 3H), 1.63–1.39 (m, 3H), 1.23 (d, *J* = 6.8 Hz, 3H), 0.93–0.86 (m, 6H).

#### 7-((3,5-difluoro-4-hydroxyphenyl)­amino)-1-isopentyl-2,4-dimethyl-1,4-dihydropyrido­[3,4-*b*]­pyrazin-3­(2H)-one (**18**)

7-((4-(benzyloxy)-3,5-difluorophenyl)­amino)-1-isopentyl-2,4-dimethyl-1,4-dihydropyrido­[3,4-*b*]­pyrazin-3­(2H)-one (420 mg, 0.87 mmol) was dissolved in EtOH (10 mL). Pd/C (42 mg) was added. The mixture was stirred for 24 h in a hydrogen atmosphere. After full conversion, the reaction mixture was filtered over a Celite pad, evaporated, purified using Flash chromatography and recrystallized from toluene yielding a white solid (79 mg, 0.2 mmol, 23%). HPLC-DAD: 254 nm: 100%, 230 nm: 100%; ESI–HRMS *m*/*z*: [M + H]^+^ calcd for 391.19401; found, 391.19443; ^1^H NMR (400 MHz, DMSO-*d*_6_, δ): 9.30 (s, 1H), 8.76 (s, 1H), 7.73 (s, 1H), 7.40–7.30 (m, 2H), 6.04 (s, 1H), 4.06 (q, *J* = 6.7 Hz, 1H), 3.25 (s, 3H), 3.09 (dt, *J* = 8.5, 7.6 Hz, 1H), 1.67–1.55 (m, 1H), 1.53–1.42 (m, 2H), 1.10 (d, *J* = 6.7 Hz, 3H), 0.98–0.87 (m, 6H). 13C NMR (101 MHz, DMSO-*d*_6_, δ): 165.44, 153.61, 153.52, 152.70, 151.25, 151.16, 142.12, 134.38, 131.58, 125.98, 125.82, 119.94, 100.99, 100.90, 100.81, 100.73, 91.86, 57.25, 45.15, 34.97, 28.10, 25.69, 22.44, 22.36, 14.77. IR (ATR) [cm^–1^]: 3209, 2955, 1646, 1609, 1476, 1436, 1219, 1144, 1011.

#### Synthesis of (*S*)-6-((3,5-difluoro-4-hydroxyphenyl)­amino)-4-isopentyl-1,3-dimethyl-3,4-dihydropyrido­[2,3-*b*]­pyrazin-2­(1H)-one (**19**)

tert-butyl-(*S*)-(1-((2,6-dichloropyridin-3-yl)­amino)-1-oxopropan-2-yl)­carbamate.

2,6-dichloropyridin-3-amine (4.31 g, 26.4 mmol) and Boc- *l* -alanine (5.0 g, 26.4 mmol) were dissolved in pyridine (35 mL) and the solution was cooled to 0 °C. Propylphosphonic acid (50% EtOAc solution, 20 mL) was added and the reaction was stirred for 16 h. Upon completion, the reaction was added to ice water and basified using Na_2_CO_3_, and extracted with EtOAc (3x). Combined organic layers were dried over sodium sulfate, filtered, evaporated and purified using Flash chromatography giving the titled product as a white solid (7.69 g, 87%). ^1^H NMR (400 MHz, DMSO-*d*_6_, δ): 9.61 (s, 1H), 8.30 (d, *J* = 8.4 Hz, 1H), 7.57 (d, *J* = 8.4 Hz, 1H), 7.29 (d, *J* = 6.6 Hz, 1H), 4.29–4.17 (m, 1H), 1.39 (s, 9H), 1.29 (d, *J* = 7.2 Hz, 3H).

(*S*)-2-amino-N-(2,6-dichloropyridin-3-yl)­propenamide.

tert-butyl-(*S*)-(1-((2,6-dichloropyridin-3-yl)­amino)-1-oxopropan-2-yl)­carbamate (5.0 g, 15.0 mmol) was added to TFA 10% in DCM (200 mL) and stirred at rt for 2 h. The reaction was basified using sat. NaHCO_3_ solution. The mixture was extracted 3x with EtOAc. The combined organic layers were dried over sodium sulfate, filtered and evaporated giving an orange oil (1.3 g, 37%) that was used without further purification.

(*S*)–N-(2,6-dichloropyridin-3-yl)-2-(isopentylamino)­propenamide.

(*S*)-2-amino-N-(2,6-dichloropyridin-3-yl)­propenamide (2.1 g, 9.0 mmol), NaOAc (1.47 g, 17.9 mmol) and isovaleraldehyde (1.16 g, 13.5 mmol) were mixed in DCE (40 mL) in a dry flask. The mixture was stirred for 10 min. Subsequently, sodium triacetoxy borohydride (3.8 g, 17.9 mmol) was added portion wise and stirred for 16 h under Ar atmosphere. The reaction was quenched with saturated NaHCO_3_ solution and stirred for 10 min. The phases were separated and the aqueous phase was extracted with ether. The combined organic layers were washed again with water, dried over sodium sulfate, and the solvent was evaporated yielding a brown oil (1.15 g, 42%). ESI–MS *m*/*z*: [M + H]^+^ calcd for 304.2 (304.1); ^1^H NMR (400 MHz, DMSO-*d*_6_, δ): 8.08 (d, *J* = 8.2 Hz, 1H), 7.71 (d, J = 8.2, 1H), 3.38 (q, *J* = 6.8 Hz, 1H), 2.84–2.69 (m, 2H), 1.70–1.58 (m, 1H), 1.45–1.31 (m, 4H), 1.29 (d, *J* = 6.9 Hz, 3H), 0.81–0.76 (m, 6H).

(*S*)-6-chloro-4-isopentyl-3-methyl-3,4-dihydropyrido­[2,3-*b*]­pyrazin-2­(1H)-one.

(*S*)–N-(2,6-dichloropyridin-3-yl)-2-(isopentylamino)­propenamide (1.13 g, 3.71 mmol) was dissolved in DMF (37 mL) and DIPEA (3.83 g, 29.7 mmol) was added. Temperature was increased to 160 °C and the mixture was stirred for 48 h. Upon completion, the reaction was cooled, diluted with water and extracted with DCM, dried over sodium sulfate filtered and evaporated giving the titled product as a brown solid (950 mg, 96%). ESI–MS *m*/*z*: [M + H]^+^ calcd for 265.9 (266.1); ^1^H NMR (400 MHz, DMSO-*d*_6_, δ): 10.59 (s, 1H), 6.96 (d, *J* = 7.8 Hz, 1H), 6.62 (d, *J* = 7.8 Hz, 1H), 4.08 (q, *J* = 6.7 Hz, 1H), 3.90–3.81 (m, 1H), 3.09–2.97 (m, 1H), 1.64–1.39 (m, 3H), 1.20 (d, *J* = 6.8 Hz, 3H), 0.94–0.86 (m, 6H).

(*S*)-6-chloro-4-isopentyl-1,3-dimethyl-3,4-dihydropyrido­[2,3-*b*]­pyrazin-2­(1H)-one.

(*S*)-6-chloro-4-isopentyl-3-methyl-3,4-dihydropyrido­[2,3-*b*]­pyrazin-2­(1H)-one (1.0 g, 3.7 mmol) and methyl iodide (2.12 g, 14.9 mmol) were added to DMF (10 mL) and cooled to 0 °C. Subsequently, sodium hydride (60% on paraffin oil, 300 mg, 7.5 mmol) was added while stirring for 2 h. TLC confirmed a conversion into a new product and the reaction was quenched ice and aq. ammonia (25%). It was extracted with EtOAc (3x). The combined organic layers were dried over sodium sulfate, filtered, evaporated and purified using Flash chromatography yielding a brown solid (560 mg, 53%). ^1^H NMR (400 MHz, DMSO-*d*_6_, δ): 7.26 (d, *J* = 8.1 Hz, 1H), 6.73 (d, *J* = 8.0 Hz, 1H), 4.21 (q, *J* = 6.7 Hz, 1H), 3.93–3.83 (m, 1H), 3.22 (s, 3H), 3.09–2.98 (m, 1H), 1.60–1.42 (m, 3H), 1.16 (d, *J* = 6.7 Hz, 3H), 0.93–0.87 (m, 6H).

(*S*)-6-((4-(benzyloxy)-3,5-difluorophenyl)­amino)-4-isopentyl-1,3-dimethyl-3,4-dihydropyrido­[2,3-*b*]­pyrazin-2­(1H)-one.

(*S*)-6-chloro-4-isopentyl-1,3-dimethyl-3,4-dihydropyrido­[2,3-*b*]­pyrazin-2­(1H)-one (530 mg, 1.89 mmol), 4-(benzyloxy)-3,5-difluoroaniline (510 mg, 2.17 mmol), Cs_2_CO_3_ (1.23 g, 3.77 mmol) and Pd G3 Xantphos (35 mg, 0.038 mmol) was added to a dry flask with a reflux condenser in an Ar atmosphere. Dry 1,4-dioxane/tBuOH (3:1, 40 mL) was added. The mixture was stirred to reflux for 3 h. The reaction was cooled to room temperature, diluted with water, extracted with EtOAc, washed with brine and dried over sodium sulfate. The residue was purified using Flash chromatography giving the titled product as a yellow solid (676 mg, 75%).

#### (*S*)-6-((3,5-difluoro-4-hydroxyphenyl)­amino)-4-isopentyl-1,3-dimethyl-3,4-dihydropyrido­[2,3-*b*]­pyrazin-2­(1H)-one (**19**)

(*S*)-6-((4-(benzyloxy)-3,5-difluorophenyl)­amino)-4-isopentyl-1,3-dimethyl-3,4-dihydropyrido­[2,3-*b*]­pyrazin-2­(1H)-one (660 mg, 1.37 mmol) was dissolved in EtOH (30 mL). Pd/C (66 mg) was added. The mixture was stirred for 24 h in a hydrogen atmosphere. After full conversion, the reaction mixture was filtered over a Celite pad, evaporated, purified using Flash chromatography and recrystallized from toluene yielding a white solid (58 mg, 11%). HPLC-DAD: 254 nm: 93.7%, 230 nm: 93.9%; ESI–HRMS *m*/*z*: [M + H]^+^ calcd for 391.19401; found, 391.19450; ^1^H NMR (400 MHz, DMSO-*d*_6_, δ): 9.28 (s, 1H), 8.80 (s, 1H), 7.35–7.25 (m, 2H), 7.19 (d, *J* = 8.3 Hz, 1H), 6.12 (d, *J* = 8.4 Hz, 1H), 4.11 (q, *J* = 6.7 Hz, 1H), 3.97–3.87 (m, 1H), 3.19 (s, 3H), 3.10–2.99 (m, 1H), 1.71–1.60 (m, 1H), 1.55–1.47 (m, 2H), 1.12 (d, *J* = 6.7 Hz, 3H), 0.91 (t, *J* = 6.9 Hz, 6H). 13C NMR (101 MHz, DMSO-*d*_6_, δ): 165.00, 153.68, 152.45 (dd, *J* = 237.7, 9.2 Hz), 149.79, 144.46, 134.24 (t, *J* = 12.7 Hz), 125.90 (t, *J* = 16.7 Hz), 123.46, 115.02, 100.88–100.28 (m), 98.15, 56.65, 43.42, 36.27, 28.22, 25.72, 22.50, 22.40, 14.71. IR (ATR) [cm^–1^]: 2953, 2868, 1592, 1517, 1474, 1221, 1131, 1002.

#### 2-((3,5-difluoro-4-hydroxyphenyl)­amino)-8-isopentyl-5-methyl-7,8-dihydropteridin-6­(5H)-one (**20**)

2-chloro-8-isopentyl-5-methyl-7,8-dihydropteridin-6­(5H)-one (100 mg, 0.37 mmol), 4-amino-2,6-difluoro-phenol (76 mg, 0.52 mmol) and conc. HCl (0.1 mL) were dissolved in 1,4-dioxane and irradiated at 160 °C in a microwave reactor for 45 min. The solvent was evaporated, the residue was taken up in EtOAc and water. The phases were separated, and the aqueous phase was extracted with EtOAc (2x). The combined organic layers were dried over sodium sulfate, evaporated and purified using Flash chromatography giving the titled product as a beige solid (101 mg, 72%). HPLC-DAD: 254 nm: 100%, 230 nm: 100%; ESI–HRMS *m*/*z*: [M + H]^+^ calcd for 378.17361; found, 378.17379; ^1^H NMR (400 MHz, DMSO-*d*_6_, δ): 9.37 (s, 1H), 9.07 (s, 1H), 7.72 (s, 1H), 7.47–7.38 (m, 2H), 4.15 (s, 2H), 3.53 (t, *J* = 7.5 Hz, 2H), 3.20 (s, 3H), 1.69–1.57 (m, 1H), 1.55–1.47 (q, *J* = 15.2, 7.1 Hz, 2H), 0.92 (d, *J* = 6.5 Hz, 6H). ^13^C NMR (101 MHz, DMSO-*d*_6_, δ): 160.8, 155.1, 152.2 (dd, *J* = 237.6, 9.0 Hz), 150.7, 138.0, 133.3 (t, *J* = 12.7 Hz), 126.5 (t, *J* = 16.7 Hz), 114.7, 101.32 (dd, *J* = 18.3, 9.4 Hz), 49.8, 44.7, 33.7, 27.3, 25.7, 22.4. IR (ATR) [cm-1]: 3272, 2957, 2920, 2870, 1647, 1522, 1435, 1236, 1011, 768.

#### 2-((3,5-difluoro-4-hydroxyphenyl)­amino)-5-ethyl-8-isopentyl-7-methyl-7,8-dihydropteridin-6­(5H)-one (**21**)

2-chloro-5-ethyl-8-isopentyl-7-methyl-7,8-dihydropteridin-6­(5H)-one (262 mg, 0.88 mmol) was dissolved in sulfolane. 4-amino-2,6-difluorophenol (128 mg, 0.88 mmol) was added and stirred for 0.5 h at 170 °C. The mixture was mixed with diethyl ether, the residue was purified with Flash chromatography (DCM and 10% MeOH in DCM), yielding 88 mg (25%) of an orange solid. HPLC-DAD: 254 nm: 97.8%, 230 nm: 94.8%; ESI–HRMS *m*/*z*: [M + H]^+^ calcd for 406.20491; found, 406.20526; ^1^H NMR (400 MHz, DMSO-*d*_6_, δ): 9.41 (s, 1H), 9.14 (s, 1H), 7.86 (s, 1H), 7.49–7.37 (m, 2H), 4.22 (q, *J* = 6.7 Hz, 1H), 4.06–3.73 (m, 3H), 3.18–3.08 (m, 1H), 1.66–1.45 (m, 3H), 1.25 (d, *J* = 6.7 Hz, 3H), 1.10 (t, *J* = 7.0 Hz, 3H), 0.94–0.89 (m, 6H). ^13^C NMR (101 MHz, DMSO-*d*_6_, δ): 163.4, 155.2, 152.22 (dd, *J* = 237.7, 9.0 Hz), 150.9, 138.4, 133.2 (t, *J* = 12.7 Hz), 126.63 (t, *J* = 16.6 Hz), 113.0, 101.5 (dd, *J* = 17.8, 9.3 Hz), 56.1, 43.0, 35.6, 35.4, 25.8, 22.5, 22.4, 16.7, 12.0. IR (ATR) [cm^–1^]: 2955, 2871, 1653, 1576, 1519, 1429, 1367, 1319, 1224, 1142.

#### 2,6-difluoro-4-((8-isopentyl-5,7-dimethyl-5,6,7,8-tetrahydropteridin-2-yl)­amino)­phenol (**22**)

*N*-(4-(benzyloxy)-3,5-difluorophenyl)-8-isopentyl-5,7-dimethyl-5,6,7,8-tetrahydropteridin-2-amine (260 mg, 0.56 mmol) was dissolved in EtOH (10 mL) and HCl conc. (0.25 mL). Pd/C (26 mg) was added. The mixture was stirred for 24 h in a hydrogen atmosphere. After full conversion, the reaction mixture was filtered over Celite, evaporated and purified using Flash chromatography yielding a gray solid (79 mg, 37%). HPLC-DAD: 254 nm: 96.5%, 230 nm: 95.6%; ESI–HRMS *m*/*z*: [M + H]^+^ calcd for 378.20999; found, 378.21032; ^1^H NMR (400 MHz, DMSO-*d*_6_, δ): 8.79 (s, 1H), 8.14 (s, 1H), 7.42–7.33 (m, 2H), 7.23 (s, 1H), 3.95–3.85 (m, 1H), 3.75–3.68 (m, 1H), 3.24–3.14 (m, 1H), 2.93–2.81 (m, 2H), 2.70 (s, 3H), 1.67–1.56 (m, 1H), 1.52–1.44 (m, 2H), 1.25 (d, *J* = 6.3 Hz, 3H), 0.94–0.89 (m, 6H). 13C NMR (101 MHz, DMSO-*d*_6_, δ): 163.60, 152.79, 152.32 (dd, *J* = 237.4, 9.0 Hz), 151.63, 133.99–133.55 (m), 132.35, 126.21–125.84 (m), 123.69, 101.21–100.70 (m), 53.89, 51.27, 44.68, 38.87, 35.97, 26.30, 22.99, 19.16. IR (ATR) [cm^–1^]: 2956, 2868, 2786, 1636, 1515, 1418, 1234, 1017, 844, 757.

#### 8-cyclohexyl-2-((3,5-difluoro-4-hydroxyphenyl)­amino)-5-methyl-7,8-dihydropteridin-6­(5H)-one (**23**)

2-chloro-8-cyclohexyl-5-methyl-7,8-dihydropteridin-6­(5H)-one (200 mg, 0.71 mmol), 4-amino-2,6-difluorophenol (193 mg, 0.82 mmol), Cs_2_CO_3_ (464 mg, 1.42 mmol) and Pd G3 Brettphos (13 mg, 0.014 mmol) was added to a dry flask with a reflux condenser in an argon atmosphere. Anhydrous 1,4-dioxane was added. The mixture was stirred to reflux for 3 h. The reaction was cooled to room temperature, diluted with water, extracted with EtOAc, washed with brine and dried over sodium sulfate. The residue was purified using Flash chromatography giving the titled product as a brown solid (44 mg, 16%). HPLC-DAD: 254 nm: 100%, 230 nm: 100%; ESI–HRMS *m*/*z*: [M + H]^+^ calcd for 390.17361; found, 390.17433; ^1^H NMR (400 MHz, DMSO-*d*_6_, δ): 9.39 (s, 1H), 9.12 (s, 1H), 7.74 (s, 1H), 7.48–7.38 (m, 2H), 4.53–4.43 (m, 1H), 4.06 (s, 2H), 3.18 (s, 3H), 1.89–1.78 (m, *J* = 12.5 Hz, 2H), 1.74–1.49 (m, 5H), 1.45–1.31 (m, 2H), 1.25–1.08 (m, 1H). 13C NMR (101 MHz, DMSO-*d*_6_, δ): 161.13, 155.05, 152.22 (dd, *J* = 237.5, 8.9 Hz), 150.48, 138.32, 133.28 (t, *J* = 12.8 Hz), 126.53 (t, *J* = 16.7 Hz), 114.87, 101.65–101.10 (m), 52.79, 44.90, 27.85, 27.27, 25.42, 25.00. IR (ATR) [cm^–1^]: 3285, 2931, 2855, 1668, 1606, 1580, 1518, 1431, 1422, 1220.

#### 2-((3,5-difluoro-4-hydroxyphenyl)­amino)-5-methyl-8-phenyl-7,8-dihydropteridin-6­(5H)-one (**24**)

2-chloro-8-cyclohexyl-5-methyl-7,8-dihydropteridin-6­(5H)-one (200 mg, 0.75 mmol), 4-amino-2,6-difluorophenol (202 mg, 0.86 mmol), Cs_2_CO_3_ (486 mg, 1.49 mmol) and Pd G3 Brettphos (14 mg, 0.015 mmol) was added to a dry flask with a reflux condenser in an argon atmosphere. Anhydrous 1,4-dioxane (11 mL) was added. The mixture was stirred to reflux for 3 h. The reaction was cooled to room temperature, diluted with water, extracted with EtOAc, washed with brine and dried over sodium sulfate. The residue was purified using Flash chromatography giving the titled product as a beige solid (107 mg, 37%). HPLC-DAD: 254 nm: 99.8%, 230 nm: 98.0%; ESI–HRMS: [M + H]+ calcd for 390.17361; found, 390.17433; ESI–MS *m*/*z*: [M – H]^−^ calcd for 381.8 (382.1); ^1^H NMR (400 MHz, DMSO-*d*_6_, δ): 9.27 (s, 1H), 9.12 (s, 1H), 7.97 (s, 1H), 7.50–7.42 (m, 4H), 7.34–7.28 (m, 1H), 7.20–7.10 (m, 2H), 4.51 (s, 2H), 3.30 (s, 3H). 13C NMR (101 MHz, DMSO-*d*_6_, δ): 161.31, 154.73, 152.04 (dd, *J* = 237.7, 8.8 Hz), 151.05, 141.31, 140.08, 132.87 (t, *J* = 12.9 Hz), 129.14, 126.46 (t, *J* = 16.4 Hz), 126.33, 125.48, 115.23, 101.52–100.88 (m), 52.31, 27.59.

#### 7-((3,5-difluoro-4-hydroxyphenyl)­amino)-1,4-dimethyl-1,4-dihydropyrido­[3,4-*b*]­pyrazin-3­(2H)-one (**25**)

7-((4-(benzyloxy)-3,5-difluorophenyl)­amino)-1,4-dimethyl-1,4-dihydropyrido­[3,4-*b*]­pyrazin-3­(2H)-one (314 mg, 0.77 mmol) and Pd/C (31 mg) were dissolved in EtOAc (18 mL). The mixture was purged with hydrogen gas for 16 h. Upon completion, the mixture was filtered over Celite, evaporated and purified using Flash chromatography with a gradient of 100% PE to 100% EtOAc giving the desired compound as a transparent solid (15 mg, 6%). HPLC-DAD: 254 nm: 99.2%, 230 nm: 96.1%; ESI–HRMS *m*/*z*: [M + H]^+^ calcd for 321.11576; found, 321.11632; ^1^H NMR (400 MHz, DMSO-*d*_6_, δ): 9.29 (s, 1H), 8.81 (s, 1H), 7.71 (s, 1H), 7.43–7.32 (m, 2H), 6.00 (s, 1H), 3.88 (s, 2H), 3.25 (s, 3H), 2.80 (s, 3H). 13C NMR (101 MHz, DMSO-*d*_6_, δ): 162.99, 152.67, 152.37 (dd, *J* = 237.7, 9.4 Hz), 144.38, 134.43 (t, *J* = 12.4 Hz), 131.12, 125.90 (t, *J* = 16.5 Hz), 120.17, 100.94–100.51 (m), 91.17, 53.08, 35.98, 27.73. IR (ATR) [cm-1]: 3351, 2923, 2851, 1643, 1504, 1231, 1008, 842, 820, 799.

#### 7-((3,5-difluoro-4-hydroxyphenyl)­amino)-4-methyl-1-propyl-1,4-dihydropyrido­[3,4-*b*]­pyrazin-3­(2H)-one (**26**)

7-((4-(benzyloxy)-3,5-difluorophenyl)­amino)-4-methyl-1-propyl-1,4-dihydropyrido­[3,4-*b*]­pyrazin-3­(2H)-one (120 mg, 0.27 mmol) was dissolved in EtOH (5 mL) and conc. HCl (0.2 mL). Pd/C (12 mg) was added. The mixture was stirred for 24 h in a hydrogen atmosphere. After full conversion, the reaction mixture was filtered over Celite, evaporated and purified using Flash chromatography yielding a beige solid (48 mg, 50%). HPLC-DAD: 254 nm: 97.6%, 230 nm: 97.0%; ESI–HRMS *m*/*z*: [M + H]^+^ calcd for 349.14706; found, 349.14735; ^1^H NMR (700 MHz, DMSO-*d*_6_, δ): 9.27 (s, 1H), 8.75 (s, 1H), 7.69 (s, 1H), 7.41–7.33 (m, 2H), 6.02 (s, 2H), 3.24 (s, 3H), 3.16–3.14 (m, 2H), 1.63–1.56 (m, 2H), 0.92 (t, *J* = 7.4 Hz, 3H). 13C NMR (176 MHz, DMSO-*d*_6_, δ): 175.5, 162.7, 152.7, 152.41 (dd, *J* = 237.7, 9.0 Hz), 143.0, 134.52 (t, *J* = 12.8 Hz), 131.4, 125.84 (t, *J* = 16.7 Hz), 119.9, 100.62 (dd, *J* = 20.5, 6.3 Hz), 90.7, 51.1, 50.1, 27.7, 17.4, 11.2. IR (ATR) [cm-1]: 2920, 1653, 1646, 1605, 1505, 1213, 1010, 801.

#### 7-((3,5-difluoro-4-hydroxyphenyl)­amino)-1-isopropyl-4-methyl-1,4-dihydropyrido­[3,4-*b*]­pyrazin-3­(2H)-one (**27**)

7-((4-(benzyloxy)-3,5-difluorophenyl)­amino)-1-isopropyl-4-methyl-1,4-dihydropyrido­[3,4-*b*]­pyrazin-3­(2H)-one (200 mg, 0.46 mmol) was dissolved in EtOH (5.0 mL). Pd/C (20 mg) was added. The mixture was stirred for 24 h in a hydrogen atmosphere. After full conversion, the reaction mixture was filtered over Celite, evaporated and purified using Flash chromatography yielding a white solid (135 mg, 85%). HPLC-DAD: 254 nm: 97.7%, 230 nm: 96.3%; ESI–MS *m*/*z*: [M + H]^+^ calcd for 349.14706; found, 349.14704; ^1^H NMR (400 MHz, DMSO-*d*_6_, δ): 9.23 (s, 1H), 8.76 (s, 1H), 7.73 (s, 1H), 7.42–7.30 (m, 2H), 6.14 (s, 1H), 3.98–3.87 (m, 1H), 3.75 (s, 2H), 3.25 (s, 3H), 1.19 (d, *J* = 6.6 Hz, 6H). IR (ATR) [cm-1]: 2968, 2925, 1654, 1604, 1502, 1436, 1222, 1013, 803.

#### 1-cyclopropyl-7-((3,5-difluoro-4-hydroxyphenyl)­amino)-4-methyl-1,4-dihydropyrido­[3,4-*b*]­pyrazin-3­(2H)-one (**28**)

7-((4-(benzyloxy)-3,5-difluorophenyl)­amino)-1-cyclopropyl-4-methyl-1,4-dihydropyrido­[3,4-*b*]­pyrazin-3­(2H)-one (150 mg, 0.34 mmol) was dissolved in EtOH (5 mL) and conc. HCl (0.2 mL). Pd/C (15 mg) was added. The mixture was stirred for 24 h in a hydrogen atmosphere. After full conversion, the reaction mixture was filtered over a Celite pad, evaporated and purified using Flash chromatography yielding transparent brownish crystals (62 mg, 52%). HPLC-DAD: 254 nm: 95.2%, 230 nm: 94.6%; ESI–HRMS *m*/*z*: [M + H]^+^ calcd for 347.13141; found, 347.13169; ^1^H NMR (400 MHz, DMSO-*d*_6_, δ): 9.26 (s, 1H), 8.88 (s, 1H), 7.75 (s, 1H), 7.44–7.33 (m, 2H), 6.49 (s, 1H), 3.86 (s, 2H), 3.23 (s, 3H), 2.41–2.35 (m, 1H), 0.88–0.57 (m, 4H). 13C NMR (101 MHz, DMSO-*d*_6_, δ): 163.5, 152.4, 152.37 (dd, *J* = 237.6, 9.1 Hz), 144.8, 134.40 (t, *J* = 12.7 Hz), 131.5, 125.93 (t, *J* = 16.9 Hz), 120.5, 100.72 (dd, *J* = 18.0, 9.0 Hz), 93.0, 51.8, 29.8, 27.8, 7.3. IR (ATR) [cm^–1^]: 3290, 2921, 1645, 1608, 1504, 1354, 1215, 1013.

#### 1-(cyclopropylmethyl)-7-((3,5-difluoro-4-hydroxyphenyl)­amino)-4-methyl-1,4-dihydropyrido­[3,4-*b*]­pyrazin-3­(2H)-one (**29**)

7-((4-(benzyloxy)-3,5-difluorophenyl)­amino)-1-(cyclopropylmethyl)-4-methyl-1,4-dihydropyrido­[3,4-*b*]­pyrazin-3­(2H)-one (130 mg, 0.29 mmol) was dissolved in EtOH (5 mL) and conc. HCl (0.2 mL). Pd/C (13 mg) was added. The mixture was stirred for 24 h in a hydrogen atmosphere. After full conversion, the reaction mixture was filtered over Celite, evaporated and purified using Flash chromatography yielding an orange solid (75 mg, 72%). HPLC-DAD: 254 nm: 98.2%, 230 nm: 97.8%; ESI–HRMS *m*/*z*: [M + H]^+^ calcd for 361.14706; found, 361.14764; ^1^H NMR (400 MHz, DMSO-*d*_6_, δ): 9.25 (s, 1H), 8.76 (s, 1H), 7.72 (s, 1H), 7.44–7.29 (m, 1H), 6.14 (s, 1H), 4.00 (s, 2H), 3.25 (s, 3H), 3.10 (d, *J* = 6.7 Hz, 2H), 1.08–0.98 (m, 1H), 0.60–0.22 (m, 4H). 13C NMR (101 MHz, DMSO-*d*_6_, δ): 163.0, 152.7, 152.38 (dd, *J* = 237.5, 9.1 Hz), 143.3, 134.47 (t, *J* = 12.9 Hz), 131.5, 125.84 (t, *J* = 16.8 Hz), 120.1, 100.62 (dd, *J* = 17.7, 9.3 Hz), 91.0, 52.4, 51.0, 27.7, 6.7, 3.3. IR (ATR) [cm-1]: 3298, 2920, 1646, 1607, 1502, 1216, 1010, 801.

#### 7-((3,5-difluoro-4-hydroxyphenyl)­amino)-4-methyl-1-propyl-1,4-dihydropyrido­[3,4-*b*]­pyrazin-3­(2H)-one (**30**)

7-((4-(benzyloxy)-3,5-difluorophenyl)­amino)-1-isobutyl-4-methyl-1,4-dihydropyrido­[3,4-*b*]­pyrazin-3­(2H)-one (146 mg, 0.32 mmol) was dissolved in EtOH (5 mL) and conc. HCl (0.2 mL). Pd/C was added. The mixture was stirred for 24 h in a hydrogen atmosphere. After full conversion, the reaction mixture was filtered over Celite, evaporated and purified using Flash chromatography yielding a gray solid (82 mg, 70%). HPLC-DAD: 254 nm: 97.6%, 230 nm: 96.9%; ESI–HRMS *m*/*z*: [M + H]^+^ calcd for 363.16271; found, 363.16287; ^1^H NMR (400 MHz, DMSO-*d*_6_, δ): 10.12 (s, 1H), 9.88 (s, 1H), 7.41 (s, 1H), 7.04 (d, *J* = 8.5 Hz, 1H), 6.21 (s, 1H), 4.21 (s, 2H), 3.22 (s, 3H), 3.15 (d, *J* = 6.4 Hz, 2H), 2.12–1.96 (m, 1H), 0.91 (d, *J* = 6.6 Hz, 6H). 13C NMR (101 MHz, DMSO-*d*_6_, δ): 161.3, 152.55 (dd, *J* = 241.7, 8.8 Hz), 148.4, 146.3, 131.07–130.65 (m), 129.13–128.62 (m), 120.2, 107.05–106.48 (m), 88.1, 56.6, 51.8, 27.9, 25.4, 19.9. IR (ATR) [cm-1]: 2958, 1689, 1610, 1559, 1517, 1468, 1437, 1357, 1314.

#### Synthesis of 1-cyclobutyl-7-((3,5-difluoro-4-hydroxyphenyl)­amino)-4-methyl-1,4-dihydropyrido­[3,4-*b*]­pyrazin-3­(2H)-one (**31**)

4,6-dichloropyridin-3-amine.

2,4-dichloro-5-nitropyridine (25 g, 0.13 mol) was dissolved in anhydrous acetic acid (350 mL). The mixture was heated to 60 °C and powdered iron (21.7 g, 0.39 mol) was added. The temperature raised while stirring vigorously. After 20 min, the temperature decreased. After another 20 min, the reaction mixture was poured in water and extracted 3x with EtOAc. The combined organic layers were washed 2x with 10% ammonia in water solution and brine, dried over sodium sulfate, evaporated. The residual oil was taken up in a mixture of hexane/diethyl ether and slowly evaporated, giving a white precipitate (17.75 g, 85%). ESI–MS *m*/*z*: [M + H]^+^ calcd for 162.9 (162.9); ^1^H NMR (400 MHz, CDCl_3_, δ): 7.91 (s, 1H), 7.23 (s, 1H), 3.98 (s, 2H). 13C NMR (101 MHz, CDCl_3_, δ): 139.8, 139.3, 136.3, 129.8, 124.0.

#### 2-chloro-*N*-(4,6-dichloropyridin-3-yl)­acetamide

4,6-dichloropyridin-3-amine (4.3 g, 22.3 mmol) was dissolved in 90 mL dichloromethane and 45 mL water and K_2_CO_3_ (6.16 g, 44.6 mmol) was added. The reaction mixture was cooled to 0 °C and chloroacetyl chloride (5.03 g, 44.6 mmol) was added dropwise. After 30 min the organic phase was separated and evaporated giving the desired product as a white substance without further purification (5.1 g, 96%). ESI–MS *m*/*z*: [M + H]^+^ calcd for 238.9 (238.9); ^1^H NMR (400 MHz, CDCl_3_, δ): 9.32 (s, 1H), 8.74 (s, 1H), 7.42 (s, 1H), 4.25 (s, 2H). 13C NMR (101 MHz, CDCl_3_, δ): 164.0, 146.9, 142.5, 134.9, 130.4, 124.2, 43.0.

#### 2-chloro-*N*-(4,6-dichloropyridin-3-yl)-*N*-methylacetamide

2-chloro-*N*-(4,6-dichloropyridin-3-yl)­acetamide (5.29 g, 22.1 mmol) and K_2_CO_3_ (6.1 g, 44.2 mmol) were added to DMF (50 mL) at rt. Subsequently, methyl iodide (6.27 g, 44.2 mmol) was added while stirring. After 4 h, HPLC confirmed a conversion into a new product and water was added to the reaction. The mixture was extracted 3x with EtOAc. The combined organic layers were washed with water and brine, dried over sodium sulfate, evaporated, and purified using Flash chromatography yielding a yellow oil (2.7 g, 49%). ^1^H NMR (400 MHz, CDCl_3_, δ): 8.50 (s, 1H), 7.55 (s, 1H), 3.47 (dd, *J* = 119.6, 10.2 Hz, 2H), 3.23 (s, 3H). 13C NMR (101 MHz, CDCl_3_, δ): 167.9, 152.2, 150.0, 144.6, 136.8, 125.9, 37.2, −4.4.

#### 2-(cyclobutylamino)-*N*-(4,6-dichloropyridin-3-yl)-*N*-methylacetamide

2-chloro-*N*-(4,6-dichloropyridin-3-yl)-*N*-methylacetamide (1.0 g, 3.9 mmol) was placed in THF and combined with K_2_CO_3_ (1.36 g, 9.9 mmol) and cyclobutylamine (0.56 g, 7.9 mmol). The reaction mixture was stirred for 2 h at rt., then diluted with water. The aqueous phase was extracted twice with ethyl acetate. The combined organic phases were dried over sodium sulfate, evaporated and purified using Flash chromatography giving a brown oil (0.93 g, 82%). ESI–MS *m*/*z*: [M + H]^+^ calcd for 288.0 (288.1).

#### 7-chloro-1-cyclobutyl-4-methyl-1,4-dihydropyrido­[3,4-*b*]­pyrazin-3­(2H)-one

2-(cyclobutylamino)-*N*-(4,6-dichloropyridin-3-yl)-*N*-methylacetamide (870 mg, 3 mmol) and DIPEA (1.17 g, 9.1 mmol) were dissolved in DMF (15 mL). The reaction was stirred for 4 h at 100 °C. Upon completion, water was added and the precipitate was suction filtered and dried giving purely the desired product was a brown solid (650 mg, 86%). ESI–MS *m*/*z*: [M + H]^+^ calcd for 252.2 (252.1); ^1^H NMR (400 MHz, CDCl_3_): δ = 7.78 (s, 1H), 6.49 (s, 1H), 3.92 (quint, *J* = 8.2 Hz, 1H), 3.83 (s, 2H), 3.36 (s, 3H), 2.40–1.75 (m) ppm. 13C NMR (101 MHz, CDCl_3_, δ): 164.2, 146.9, 144.0, 133.5, 125.9, 106.1, 52.8, 47.3, 28.6, 27.7, 14.9.

#### 7-((4-(benzyloxy)-3,5-difluorophenyl)­amino)-1-cyclobutyl-4-methyl-1,4-dihydropyrido­[3,4-*b*]­pyrazin-3­(2H)-one

7-chloro-1-cyclobutyl-4-methyl-1,4-dihydropyrido­[3,4-*b*]­pyrazin-3­(2H)-one (300 mg, 1.19 mmol), 4-(benzyloxy)-3,5-difluoroaniline (561 mg, 2.38 mmol), Brettphos Pd G3 (22 mg, 0.024 mmol) and KOtBu (267 mg, 2.38 mmol) were added to a dry flask with a stirrer in an argon atmosphere. Anhydrous 1,4-dioxane (17 mL) was added. The mixture was heated to 80 °C for 4 h in an argon atmosphere. After full conversion, the reaction mixture was filtered over Celite, evaporated and purified using Flash chromatography yielding a brown solid (164 mg, 31%) that was used in the next step without further purification. ESI–MS *m*/*z*: [M + H]^+^ calcd for 451.4 (451.2).

#### 1-cyclobutyl-7-((3,5-difluoro-4-hydroxyphenyl)­amino)-4-methyl-1,4-dihydropyrido­[3,4-*b*]­pyrazin-3­(2H)-one (**31**)

7-((4-(benzyloxy)-3,5-difluorophenyl)­amino)-1-cyclobutyl-4-methyl-1,4-dihydropyrido­[3,4-*b*]­pyrazin-3­(2H)-one (164 mg, 0.36 mmol) was dissolved in EtOH (5 mL) and conc. HCl (0.2 mL). Pd/C (16 mg) was added. The mixture was stirred for 24 h in a hydrogen atmosphere. After full conversion, the reaction mixture was filtered over Celite, evaporated and purified using Flash chromatography yielding brown crystals (90 mg, 69%). HPLC-DAD: 254 nm: 100%, 230 nm: 97.3%; ESI–HRMS *m*/*z*: [M + H]^+^ calcd for 361.14706; found, 361.14721; ^1^H NMR (400 MHz, DMSO-*d*_6_, δ): 9.29 (s, 1H), 8.78 (s, 1H), 7.74 (s, 1H), 7.40–7.30 (m, 2H), 6.05 (s, 1H), 3.90–3.80 (m, 1H), 3.71 (s, 2H), 3.24 (s, 3H), 2.32–2.22 (m, 2H), 2.16–2.01 (m, 2H), 1.78–1.67 (m, 2H). 13C NMR (101 MHz, DMSO-*d*_6_, δ): 163.7, 152.5, 152.39 (dd, *J* = 237.7, 9.0 Hz), 143.4, 134.36 (t, *J* = 12.8 Hz), 131.5, 125.95 (t, *J* = 16.7 Hz), 120.7, 100.68 (dd, *J* = 17.9, 9.0 Hz), 92.6, 52.2, 47.0, 27.8, 27.0, 14.4. IR (ATR) [cm^–1^]: 3214, 2952, 2852, 1643, 1612, 1423, 1231, 1019, 1011, 802.

#### 1-cyclopentyl-7-((3,5-difluoro-4-hydroxyphenyl)­amino)-4-methyl-1,4-dihydropyrido­[3,4-*b*]­pyrazin-3­(2H)-one (**32**)

7-((4-(benzyloxy)-3,5-difluorophenyl)­amino)-1-cyclopentyl-4-methyl-1,4-dihydropyrido­[3,4-*b*]­pyrazin-3­(2H)-one (210 mg, 0.45 mmol) was dissolved in EtOH (5 mL). Pd/C (21 mg) was added. The mixture was stirred for 24 h in a hydrogen atmosphere. After full conversion, the reaction mixture was filtered over Celite, evaporated and purified using Flash chromatography yielding a brown solid (38 mg, 22%). HPLC-DAD: 254 nm: 96.2%, 230 nm: 95.7%; ESI–HRMS *m*/*z*: [M + H]^+^ calcd for 375.16271; found, 375.16296; ^1^H NMR (400 MHz, DMSO-*d*_6_, δ): 9.59 (s, 1H), 9.05 (s, 1H), 7.60 (s, 1H), 7.30–7.21 (m, 2H), 6.23 (s, 1H), 4.12–4.03 (m, 1H), 3.89 (s, 2H), 3.24 (s, 3H), 1.93–1.81 (m 2H), 1.79–1.53 (m, 6H). IR (ATR) [cm-1]: 2950, 2870, 1663, 1603, 1502, 1436, 1220, 1185, 1008.

#### 7-((3,5-difluoro-4-hydroxyphenyl)­amino)-4-methyl-1-((1s,4s)-4-methylcyclohexyl)-1,4-dihydropyrido­[3,4-*b*]­pyrazin-3­(2H)-one (**33**)

7-((4-(benzyloxy)-3,5-difluorophenyl)­amino)-4-methyl-1-((1s,4s)-4-methylcyclohexyl)-1,4-dihydropyrido­[3,4-*b*]­pyrazin-3­(2H)-on (200 mg, 0.41 mmol) was dissolved in EtOH (5 mL) and conc. HCl (0.2 mL). Pd/C (20 mg) was added. The mixture was stirred for 24 h in a hydrogen atmosphere. After full conversion, the reaction mixture was filtered over Celite, evaporated and purified using Flash chromatography yielding a gray solid (132 mg, 81%). HPLC-DAD: 254 nm: 98.5%, 230 nm: 97.3%; ESI–HRMS *m*/*z*: [M + H]^+^ calcd for 403.19401; found, 403.19416; ^1^H NMR (400 MHz, DMSO-*d*_6_, δ): 9.27 (s, 1H), 8.72 (s, 1H), 7.72 (s, 1H), 7.40–7.31 (m, 2H), 6.13 (s, 1H), 3.78 (s, 2H), 3.48–3.38 (m, 1H), 3.24 (s, 3H), 1.79 (d, *J* = 12.0 Hz, 2H), 1.69 (d, *J* = 10.1 Hz, 2H), 1.58 (qd, *J* = 12.3, 3.0 Hz, 2H), 1.44–1.32 (m, 1H), 1.08 (qd, *J* = 13.0, 3.1 Hz, 2H), 0.90 (d, *J* = 6.5 Hz, 3H). 13C NMR (101 MHz, DMSO-*d*_6_, δ): 163.2, 152.8, 152.42 (dd, *J* = 237.6, 9.1 Hz), 134.5 (t, *J* = 12.7 Hz), 131.8, 125.84 (t, *J* = 16.7 Hz), 100.57 (dd, *J* = 18.0, 9.2 Hz), 91.3, 55.0, 45.3, 34.0, 31.4, 27.8, 27.0, 22.2. IR (ATR) [cm^–1^]: 3258, 2920, 2851, 1647, 1500, 1230, 1216, 1023.

#### 1-(cyclohexylmethyl)-7-((3,5-difluoro-4-hydroxyphenyl)­amino)-4-methyl-1,4-dihydropyrido­[3,4-*b*]­pyrazin-3­(2H)-one (**34**)

7-((4-(benzyloxy)-3,5-difluorophenyl)­amino)-1-(cyclohexylmethyl)-4-methyl-1,4-dihydropyrido­[3,4-*b*]­pyrazin-3­(2H)-one (98 mg, 0.20 mmol) was dissolved in EtOH (5 mL). Pd/C (10 mg) was added. The mixture was stirred for 24 h in a hydrogen atmosphere. After full conversion, the reaction mixture was filtered over Celite, evaporated and purified using Flash chromatography yielding a brown solid (30 mg, 38%). HPLC-DAD: 254 nm: 97.4%, 230 nm: 96.4%; ESI–HRMS *m*/*z*: [M + H]^+^ calcd for 403.19401; found, 403.19432; ^1^H NMR (400 MHz, DMSO-*d*_6_, δ): 9.26 (s, 1H), 8.72 (s, 1H), 7.68 (s, 1H), 7.40–7.30 (m, 2H), 6.03 (s, 1H), 3.95 (s, 2H), 3.25 (s, 3H), 3.02 (d, *J* = 6.9 Hz, 2H), 1.79–1.59 (m), 1.29–1.09 (m), 1.04–0.89 (m). 13C NMR (101 MHz, DMSO-*d*_6_, δ): 162.3, 152.6, 152.40 (dd, *J* = 237.6, 8.9 Hz), 143.3, 134.5, 131.5, 125.8, 119.6, 100.61 (dd, *J* = 17.8, 9.0 Hz), 90.6, 54.9, 52.4, 34.8, 30.5, 27.7, 26.0, 25.3. IR (ATR) [cm-1]: 2920, 2849, 1653, 1605, 1506, 1436, 1205, 1008, 802.

#### 1-cycloheptyl-7-((3,5-difluoro-4-hydroxyphenyl)­amino)-4-methyl-1,4-dihydropyrido­[3,4-*b*]­pyrazin-3­(2H)-one (**35**)

7-((4-(benzyloxy)-3,5-difluorophenyl)­amino)-1-cycloheptyl-4-methyl-1,4-dihydropyrido­[3,4-*b*]­pyrazin-3­(2H)-one (253 mg, 0.51 mmol) was dissolved in EtOH (5 mL) and conc. HCl (0.2 mL). Pd/C (21 mg) was added. The mixture was stirred for 24 h in a hydrogen atmosphere. After full conversion, the reaction mixture was filtered over Celite, evaporated and purified using Flash chromatography yielding the titled product as a beige solid (78 mg, 38%). HPLC-DAD: 254 nm: 100%, 230 nm: 99.3%; ESI–HRMS *m*/*z*: [M + H]^+^ calcd for 403.19401; found, 403.19419; ^1^H NMR (400 MHz, DMSO-*d*_6_, δ): 9.27 (s, 1H), 8.75 (s, 1H), 7.72 (s, 1H), 7.41–7.31 (m, 2H), 6.11 (s, 1H), 3.77 (s, 2H), 3.65–3.56 (m, 1H), 3.24 (s, 3H), 1.83–1.39 (m). 13C NMR (101 MHz, DMSO-*d*_6_, δ): 163.4, 152.40 (dd, *J* = 237.8, 9.2 Hz), 152.7, 143.3, 134.48 (t, *J* = 12.8 Hz), 131.8, 125.87 (t, *J* = 16.6 Hz), 120.5, 100.63 (dd, *J* = 17.9, 9.3 Hz), 91.6, 56.6, 45.4, 29.9, 27.8, 27.1, 24.9. IR (ATR) [cm^–1^]: 2920, 2852, 1653, 1646, 1616, 1605, 1503, 1222, 1013.

#### Methyl 4-((1-cyclohexyl-4-methyl-3-oxo-1,2,3,4-tetrahydropyrido­[3,4-*b*]­pyrazin-7-yl)­amino)-2,6-difluorobenzoate (**36**)

Methyl 4-((1-cyclohexyl-4-methyl-3-oxo-1,2,3,4-tetrahydropyrido­[3,4-*b*]­pyrazin-7-yl)­amino)-2,6-difluorobenzoate (109 mg, 0.25 mmol), was dissolved in THF (5 mL). One M LiOH solution in water (0.5 mL, 0.5 mmol) was added dropwise and stirred for 1 h at rt. The solvent was evaporated and the pH of the remaining aqueous phase was adjusted to 2. A precipitate formed that was suction filtered giving the titled product as a white solid (63 mg, 60%). HPLC-DAD: 254 nm: 98.1%, 230 nm: 97.0%; ESI–HRMS *m*/*z*: [M + H]^+^ calcd for 417.1735 (417.1733); ^1^H NMR (400 MHz, DMSO-*d*_6_, δ): 13.76–12.92 (m, 1H), 10.49 (s, 1H), 7.62 (s, 1H), 7.06 (d, *J* = 10.6 Hz, 2H), 6.67 (s, 1H), 4.14 (s, 2H), 3.78–3.69 (m, 1H), 3.26 (s, 3H), 1.84–1.71 (m), 1.69–1.50 (m), 1.38 (m), 1.14 (m). 13C NMR (101 MHz, DMSO-*d*_6_, δ): 162.0, 161.8, 161.04 (dd, *J* = 251.2, 9.3 Hz), 146.1, 145.9, 144.55 (t, *J* = 14.1 Hz), 122.30–122.05 (m), 122.0, 101.7, 101.4, 92.5, 67.0, 56.5, 45.2, 28.0, 27.5, 24.9. IR (ATR) [cm-1]: 3203, 2924, 2852, 2492, 1581, 1550, 1521, 1222, 1044.

#### 1-cyclohexyl-4-methyl-7-((2,3,5,6-tetrafluoro-4-hydroxyphenyl)­amino)-1,4-dihydropyrido­[3,4-*b*]­pyrazin-3­(2H)-one (**37**)

7-((4-(benzyloxy)-2,3,5,6-tetrafluorophenyl)­amino)-1-cyclohexyl-4-methyl-1,4-dihydropyrido­[3,4-*b*]­pyrazin-3­(2H)-one (222 mg, 0.43 mmol) was dissolved in EtOH (10 mL) and conc. HCl (0.25 mL). Pd/C (22 mg) was added. The mixture was stirred for 24 h in a hydrogen atmosphere. After full conversion, the reaction mixture was filtered over Celite, evaporated and purified using Flash chromatography yielding gray crystals (63 mg, 34%). HPLC-DAD: 254 nm: 97.6%, 230 nm: 94.6%; ESI–HRMS *m*/*z*: [M + H]^+^ calcd for 425.15952; found 425.16008; ^1^H NMR (400 MHz, DMSO-*d*_6_, δ): 12.74 (s, 1H), 11.72 (s, 1H), 9.79 (s, 1H), 7.31 (s, 1H), 6.37 (s, 1H), 4.13 (s, 2H), 3.72–3.64 (m, 1H), 3.21 (s, 3H), 1.86–1.70 (m, 4H), 1.67–1.50 (m, 3H), 1.43–1.28 (m, 2H), 1.23–1.09 (m, 1H). IR (ATR) [cm^–1^]: 2925, 1682, 1606, 1507, 1362, 1228, 977, 818.

#### 5-((1-cyclohexyl-4-methyl-3-oxo-1,2,3,4-tetrahydropyrido­[3,4-*b*]­pyrazin-7-yl)­amino)-3-fluoro-2-hydroxybenzonitrile (**38**)

*N*-(4-(benzyloxy)-3,5-difluorophenyl)-1-cyclohexyl-1H-imidazo­[4,5-*c*]­pyridin-6-amine (200 mg, 0.4 mmol) was dissolved in EtOH (11 mL). The mixture was purged with nitrogen and Pd/C (20 mg) was added. The mixture was purged with hydrogen and concentrated hydrochloric acid (0.1 mL) was added. The reaction was stirred for 4 h at room temperature. After completion, the reaction was filtered over Celite, evaporated and purified using Flash chromatography. The combined fractions were taken up in EtOAc and washed 3x with 2N NaOH solution. The combined aqueous phases were acidified and the resulting precipitate was suction filtered giving purely the desired product as a white powder (19 mg, 12%). HPLC-DAD: 254 nm: 99.6%, 230 nm: 100%; ESI–HRMS *m*/*z*: [M + H]^+^ calcd for 396.18303; found, 396.18327; ^1^H NMR (400 MHz, DMSO-*d*_6_, δ): 12.71 (s, 1H), 11.47 (s, 1H), 10.05 (s, 1H), 7.56 (d, *J* = 12.1 Hz, 1H), 7.46 (s, 1H), 7.40 (s, 1H), 6.36 (s, 1H), 4.12 (s, 2H), 3.67–3.60 (m, 1H), 3.22 (s, 3H), 1.86–1.08 (m). 13C NMR (101 MHz, DMSO-*d*_6_, δ): 161.7, 152.7, 150.3, 148.4, 145.8, 122.4, 120.7, 102.4, 88.5, 56.6, 45.2, 28.0, 27.4, 24.9, 24.8. IR (ATR) [cm^–1^]: 3412, 2933, 2862, 1669, 1599, 1538, 1494, 1219, 1153, 825.

#### 5-((1-cyclopropyl-4-methyl-3-oxo-1,2,3,4-tetrahydropyrido­[3,4-*b*]­pyrazin-7-yl)­amino)-3-fluoro-2-hydroxybenzonitrile (**39**)

2-(benzyloxy)-5-((1-cyclopropyl-4-methyl-3-oxo-1,2,3,4-tetrahydropyrido­[3,4-*b*]­pyrazin-7-yl)­amino)-3-fluorobenzonitrile (200 mg, 0.45 mmol) and Pd/C (20 mg) were dissolved in MeOH (11 mL). The mixture was purged with hydrogen for 16 h. Upon completion, the mixture was filtered over Celite, evaporated and purified using Flash chromatography with a gradient of 100% DCM to 10% MeOH/90% DCM/0.2 M NH_3_ giving a yellow solid (39 mg, 24%). HPLC-DAD: 254 nm: 99.6%, 230 nm: 99.0%; ESI–HRMS *m*/*z*: [M + H]+ calcd for 354.13608; found, 354.13651; ^1^H NMR (400 MHz, DMSO-*d*_6_, δ): 10.62 (s, 1H), 9.05 (s, 1H), 7.85 (dd, *J* = 13.7, 2.6 Hz, 1H), 7.77 (s, 1H), 7.75 (dd, *J* = 2.4, 1.3 Hz, 1H), 6.48 (s, 1H), 3.87 (s, 2H), 3.23 (s, 3H), 2.42–2.35 (m, 1H), 0.88–0.82 (m, 2H), 0.65–0.57 (m, 2H). 13C NMR (101 MHz, DMSO-*d*_6_, δ): 163.49, 152.29, 152.12, 149.92, 144.87, 140.77, 140.60, 135.58, 135.48, 131.45, 120.75, 116.48, 116.42, 115.49, 110.70, 110.47, 101.53, 101.46, 93.03, 51.76, 29.78, 27.80, 7.36. IR (ATR) [cm-1]: 2920, 2851, 2216, 1660, 1610, 1449, 1349, 1216, 1142, 1020, 820.

#### 5-((1-cyclopropyl-4-methyl-3-oxo-1,2,3,4-tetrahydropyrido­[3,4-*b*]­pyrazin-7-yl)­amino)-2-hydroxybenzonitrile (**40**)

2-(benzyloxy)-5-((1-cyclopropyl-4-methyl-3-oxo-1,2,3,4-tetrahydropyrido­[3,4-*b*]­pyrazin-7-yl)­amino)­benzonitrile (85.0 mg, 0.20 mmol) and Pd/C (8.5 mg) were dissolved in MeOH (4.5 mL). The mixture was purged with hydrogen for 16 h. Upon completion, the mixture was filtered over Celite, evaporated and purified using Flash chromatography with a gradient of 100% DCM to 10% MeOH/90% DCM/0.2 M NH_3_ giving the desired compound as a yellow solid (16 mg, 24%). HPLC-DAD: 254 nm: 96.6%, 230 nm: 97.9%; ESI–HRMS *m*/*z*: [M + H]^+^ calcd for 336.14550; found, 336.14573; ^1^H NMR (400 MHz, DMSO-*d*_6_, δ): 10.42 (s, 1H), 8.83 (s, 1H), 8.13 (d, *J* = 2.7 Hz, 1H), 7.74 (s, 1H), 7.51 (dd, *J* = 9.0, 2.7 Hz, 1H), 6.92 (d, *J* = 9.0 Hz, 1H), 6.49 (s, 1H), 3.85 (s, 2H), 3.22 (s, 3H), 2.40–2.33 (m, 1H), 0.87–0.81 (m, 2H), 0.64–0.56 (m, 2H). 13C NMR (101 MHz, DMSO-*d*_6_, δ): 163.5, 153.6, 152.6, 144.8, 134.8, 131.5, 124.9, 120.4, 120.2, 117.5, 116.6, 98.0, 92.7, 51.8, 29.8, 27.8, 7.4. IR (ATR) [cm^–1^]: 3360, 2923, 2851, 2212, 1621, 1429, 1328, 1273, 1216, 818, 685.

#### 1-cyclopropyl-7-((3-fluoro-4-hydroxy-5-(trifluoromethyl)­phenyl)­amino)-4-methyl-1,4-dihydropyrido­[3,4-*b*]­pyrazin-3­(2H)-one (**41**)

7-((4-(benzyloxy)-3-fluoro-5-(trifluoromethyl)­phenyl)­amino)-1-cyclopropyl-4-methyl-1,4-dihydropyrido­[3,4-*b*]­pyrazin-3­(2H)-one (314 mg, 0.65 mmol) and Pd/C (31 mg) were dissolved in MeOH (18 mL). The mixture was purged with hydrogen gas. Upon completion, the mixture was filtered over Celite, evaporated and purified using Flash chromatography giving the desired compound as a transparent solid (46 mg, 18%). HPLC-DAD: 254 nm: 97.0%, 230 nm: 97.4%; ESI–HRMS *m*/*z*: [M + H]^+^ calcd for 397.12821; found, 397.12894; ^1^H NMR (400 MHz, DMSO-*d*_6_, δ): 9.92 (s, 1H), 9.03 (s, 1H), 7.97 (dd, *J* = 13.6, 2.4 Hz, 1H), 7.75 (s, 1H), 7.61 (s, 1H), 6.49 (s, 1H), 3.87 (s, 2H), 3.23 (s, 3H), 2.42–2.35 (m, 1H), 0.84 (q, *J* = 6.7 Hz, 2H), 0.64–0.58 (m, 2H). 19F NMR (659 MHz, DMSO-*d*_6_, δ): −60.43 (s), −132.16 (d, *J* = 13.5 Hz). 13C NMR (101 MHz, DMSO-*d*_6_, δ): 163.50, 152.82, 152.31, 150.47, 144.85, 135.82, 135.65, 134.97, 134.87, 131.50, 120.66, 118.17, 110.05, 108.88, 108.64, 92.99, 51.79, 48.59, 29.79, 27.80, 7.34. IR (ATR) [cm^–1^]: 3353, 1661, 1617, 1500, 1356, 1229, 1117, 1019, 1005, 885.

#### yl)­amino)-3-fluoro-2-hydroxybenzoate (**42**)

Methyl-2-(benzyloxy)-5-((1-cyclopropyl-4-methyl-3-oxo-1,2,3,4-tetrahydropyrido­[3,4-*b*]­pyrazin-7-yl)­amino)-3-fluorobenzoate (134 mg, 0.28 mmol) and Pd/C 5% (13.4 mg) were dissolved in MeOH (7.5 mL). A drop of concentrated hydrochloric acid was added. The mixture was purged with hydrogen for 16 h. Upon completion, the mixture was filtered over Celite, evaporated and purified using Flash chromatography with a gradient of 100% DCM to 10% MeOH/90% DCM giving the desired compound as a white solid (43 mg, 40%). HPLC-DAD: 254 nm: 97.0%, 230 nm: 96.3%; ESI–HRMS *m*/*z*: [M + H]^+^ calcd for 387.14631; found, 387.14655; ^1^H NMR (400 MHz, DMSO-*d*_6_, δ): 9.91 (s, 1H), 8.99 (s, 1H), 8.02 (dd, *J* = 13.8, 2.6 Hz, 1H), 7.80 (dd, *J* = 2.4, 1.6 Hz, 1H), 7.74 (s, 1H), 6.50 (s, 1H), 3.91 (s, 3H), 3.87 (s, 2H), 3.23 (s, 3H), 2.42–2.34 (m, 1H), 0.88–0.82 (m, 2H), 0.65–0.59 (m, 2H). 13C NMR (101 MHz, DMSO-*d*_6_, δ): 168.4, 168.4, 163.4, 152.3, 151.7, 149.3, 144.9, 141.0, 140.9, 134.4, 134.3, 131.3, 120.6, 115.2, 115.2, 112.9, 112.8, 111.5, 111.3, 92.8, 52.6, 51.8, 29.8, 27.8, 7.3. IR (ATR) [cm^–1^]: 3224, 3071, 1671, 1602, 1476, 1437, 1363, 1228, 990, 786.

#### 1-cyclopropyl-7-((3,5-dichloro-4-hydroxyphenyl)­amino)-4-methyl-1,4-dihydropyrido­[3,4-*b*]­pyrazin-3­(2H)-one (**43**)

7-((4-((tert-butyldimethylsilyl)­oxy)-3,5-dichlorophenyl)­amino)-1-cyclopropyl-4-methyl-1,4-dihydropyrido­[3,4-*b*]­pyrazin-3­(2H)-one (305 mg, 0.62 mmol) was dissolved in THF. 1.0 M tetrabutylammonium fluoride in THF (3.1 mL, 3.1 mmol) was added and stirred for 2 h at rt. Upon completion, the solvent was evaporated and purified using Flash chromatography. Remaining grease was taken up in diethyl ether while the titled product precipitated as a gray solid (80 mg, 34%). HPLC-DAD: 254 nm: 98.0%, 230 nm: 96.5%; ESI–HRMS *m*/*z*: [M + H]^+^ calcd for 379.07231; found, 379.07276; ^1^H NMR (400 MHz, DMSO-*d*_6_, δ): 9.35 (s, 1H), 8.89 (s, 1H), 7.77 (s, 1H), 7.72 (s, 2H), 6.48 (s, 1H), 3.87 (s, 2H), 3.23 (s, 3H), 2.43–2.33 (m, 1H), 0.84 (dd, *J* = 6.7, 2.1 Hz, 2H), 0.61 (dd, *J* = 3.9, 2.3 Hz, 2H). 13C NMR (101 MHz, DMSO-*d*_6_, δ): 163.46, 152.24, 144.83, 141.79, 136.00, 131.49, 122.47, 120.59, 117.01, 92.97, 51.78, 29.77, 27.79, 7.34. IR (ATR) [cm^–1^]: 3228, 3052, 1653, 1598, 1477, 1429, 1258, 1161, 1148, 816.

#### 1-cyclopropyl-7-((2,6-difluoro-4-hydroxyphenyl)­amino)-4-methyl-1,4-dihydropyrido­[3,4-*b*]­pyrazin-3­(2H)-one (**44**)

7-((4-((tert-butyldimethylsilyl)­oxy)-2,6-difluorophenyl)­amino)-1-cyclopropyl-4-methyl-1,4-dihydropyrido­[3,4-*b*]­pyrazin-3­(2H)-one (80 mg, 0.17 mmol) was dissolved in THF. One M tetrabutylammonium fluoride in THF (1 mL, 0.87 mmol) was added and stirred for 2 h at rt. Upon completion, the solvent was evaporated and purified using Flash chromatography. Remaining grease was taken up in diethyl ether while the titled product precipitated as a white solid (80 mg, 34%). HPLC-DAD: 254 nm: 97.1%, 230 nm: 97.0%; ESI-HRMS *m*/*z*: [M + H]^+^ calcd for 347.13141; found, 347.13190; ^1^H NMR (400 MHz, DMSO-*d*_6_, δ): 10.10 (s, 1H), 7.77 (s, 1H), 7.53 (s, 1H), 6.54–6.42 (m, 2H), 6.34 (s, 1H), 3.82 (s, 2H), 3.17 (s, 3H), 2.40–2.28 (m, 1H), 0.83–0.74 (m, 2H), 0.61–0.52 (m, 2H). 13C NMR (101 MHz, DMSO-*d*_6_, δ): 163.43, 159.29 (dd, *J* = 243.9, 8.8 Hz), 155.75 (t, *J* = 14.4 Hz), 144.98, 132.02, 119.93, 108.96 (t, *J* = 17.3 Hz), 99.09 (d, *J* = 26.1 Hz), 90.84, 51.90, 29.77, 27.73, 7.27. IR (ATR) [cm^–1^]: 3256, 2796, 2590, 1667, 1616, 1517, 1476, 1262, 1148, 1022.

#### 3-chloro-5-((1-cyclopropyl-4-methyl-3-oxo-1,2,3,4-tetrahydropyrido­[3,4-*b*]­pyrazin-7-yl)­amino)-2-hydroxybenzonitrile (**45**)

7-chloro-1-cyclopropyl-4-methyl-1,4-dihydropyrido­[3,4-*b*]­pyrazin-3­(2H)-one (300 mg, 1.26 mmol), 5-amino-2-((tert-butyldimethylsilyl)­oxy)-3-chlorobenzonitrile (427 mg, 1.51 mmol), Xantphos Pd G4 (24 mg, 0.025 mmol) and KOtBu (170 mg, 1.51 mmol) were added to a dry flask with a stirrer in an argon atmosphere. Anhydrous 1,4-dioxane (17 mL) was added. The mixture was refluxed for 4 h in an argon atmosphere. The mixture was cooled to rt and filtered over Celite. The residue was evaporated and purified using normal phase Flash chromatography with a gradient of DCM to 10% MeOH in DCM and reverse phase using a gradient of 0.1% FA in water to 100% MeOH giving the desired product as a gray solid (33 mg, 7%). HPLC-DAD: 254 nm: 100%, 230 nm: 100%; ESI–HRMS *m*/*z*: [M + H]^+^calcd for 370.10653; found, 370.10707; ^1^H NMR (400 MHz, DMSO-*d*_6_, δ): 10.37 (s, 1H), 9.06 (s, 1H), 8.03 (d, *J* = 2.7 Hz, 1H), 7.92 (d, *J* = 2.6 Hz, 1H), 7.79 (s, 1H), 6.48 (s, 1H), 3.88 (s, 2H), 3.23 (s, 3H), 2.43–2.36 (m, 1H), 0.89–0.81 (m, 2H), 0.66–0.58 (m, 2H). 13C NMR (101 MHz, DMSO-*d*_6_, δ): 163.48, 152.03, 144.90, 131.48, 123.36, 122.35, 120.80, 119.31, 116.61, 102.17, 93.06, 51.76, 29.78, 27.81, 7.36. IR (ATR) [cm^–1^]: 2964, 2200, 1669, 1615, 1540, 1457, 1344, 1233, 835.

#### 1-cyclopropyl-7-((4-hydroxy-3,5-bis­(trifluoromethyl)­phenyl)­amino)-4-methyl-1,4-dihydropyrido­[3,4-*b*]­pyrazin-3­(2H)-one (**46**)

7-((4-(benzyloxy)-3,5-bis­(trifluoromethyl)­phenyl)­amino)-1-cyclopropyl-4-methyl-1,4-dihydropyrido­[3,4-*b*]­pyrazin-3­(2H)-one (58 mg, 0.11 mmol) and 0.05 mL 4 N HCl in 1,4-dioxane were added to EtOH (3 mL). The mixture was bubbled with nitrogen gas. Pd/C (6 mg) was added and the reaction mixture was bubbled with hydrogen gas for 16 h at rt. Upon completion, the mixture was filtered over Celite, evaporated and purified using Flash chromatography. The product was taken up in diethyl ether, sonicated and suction filtered yielding a white powder (22 mg, 46%). HPLC-DAD: 254 nm: 92.7%, 230 nm: 95.2%; ESI–HRMS *m*/*z*: [M + H]^+^calcd for 447.12502; found, 447.12571; ^1^H NMR (400 MHz, DMSO-*d*_6_, δ): 9.63 (s, 1H), 9.21 (s, 1H), 8.23 (s, 2H), 7.77 (s, 1H), 6.52 (s, 1H), 3.88 (s, 2H), 3.24 (s, 3H), 2.43–2.37 (m, 1H), 0.84 (dt, *J* = 6.7, 3.3 Hz, 2H), 0.65–0.58 (m, 2H). 13C NMR (101 MHz, DMSO-*d*_6_, δ): 163.52, 152.13, 144.94, 135.98, 131.52, 127.53, 124.81, 122.10, 121.74, 121.44, 121.15, 120.89, 118.53, 93.15, 51.77, 29.81, 27.83, 7.36. IR (ATR) [cm-1]: 3629, 2499, 3261, 3109, 1666, 1602, 1477, 1234, 1097, 827, 674.

## Supplementary Material







## Notes

Supporting Information—molecular strings, detailed synthetic procedures, NMR spectra, mass spectra, HPLC traces, thermal shift data, cellular viability data (PDF)
Molecular strings & IC50 values (CSV)
Homology model of MKK4 based on 4D9K with BID1870 (PDB)

## Abbreviations

ATF2: activating transcription factor 2
DCM: dichloromethane
DMA: *N*,*N*-dimethylacetamide
DMF: *N*,*N*-dimethylformamide
ELK1: ETS transcription factor
EtOAc: ethyl acetate
EtOH: ethanol
JNK1: c-Jun-N-terminal kinase 1
MAPK: mitogen-activated protein kinase
MAP3K11: mitogen-activated protein kinase kinase 11
MAP4K4: mitogen-activated kinase kinase 4
MeCN: acetonitrile
MeOH: methanol
MKK4: mitogen-activated protein kinase 4
MKK6: mitogen-activated kinase 6
MKK7: mitogen-activated protein kinase 7
MW: micro wave
NAFLD: nonalcoholic fatty liver disease
NASH: nonalcoholic steatohepatitis
RPS6KA6: ribosomal protein S6 kinase A6
RSK: ribosomal s6 kinase
rt: room temperature
shRNA: short hairpin RNA
TEA: triethylamine
THF: tetrahydrofuran.
