# Supplementary material for: Discovery of the First Highly Selective 1,4-dihydropyrido[3,4‑b]pyrazin-3(2H)-one MKK4 Inhibitor
Source: J Med Chem. 2025 Jul 11;68(14):14782–805. doi: 10.1021/acs.jmedchem.5c00919 (PMC12305495; doi:10.1021/acs.jmedchem.5c00919)
Supplement: Supplementary file 1 [file jm5c00919_si_001.pdf]

Supporting Information

Discovery of the First Highly Selective 1,4-dihydropyrido[3,4-b]pyrazin-3(2H)-one Based MKK4 Inhibitor

*Leon Katzengruber [a, b], Pascal Sander [a], Stefan Zwirner [c, d], Alexander Rasch [a], Eric Eberlein [a], Roland Selig [a, d], Wolfgang Albrecht [d], Lars Zender [c, e, f, g], Stefan A. Laufer [a, e, f, g, *]*

[a] Department of Pharmaceutical/Medicinal Chemistry, Eberhard Karls University Tübingen, Auf der Morgenstelle 8, 72076, Tübingen, Germany

[b] Department of Chemical and Systems Biology, Stanford University, 290 Jane Stanford Way, Stanford, CA 94305, USA.

[c] Department of Medical Oncology and Pneumology (Internal Medicine VIII), University Hospital Tübingen, 72076, Tübingen, Germany

[d] HepaRegenix GmbH, Eisenbahnstraße 63, 72072, Tübingen, Germany

[e] iFIT Cluster of Excellence (EXC2180) “Image Guided and Functionally Instructed Tumor Therapies”, Eberhard Karls University of Tübingen, 72076, Tübingen, Germany

[f] German Consortium for Translational Cancer Research (DKTK), Partner Site Tübingen, German Cancer Research Center (DKFZ), 69120, Heidelberg, Germany

[g] Tübingen Center for Academic Drug Discovery & Development (TüCAD$_2$), Auf der Morgenstelle 8, 72076, Tübingen, Germany, stefan.laufer@uni-tuebingen.de

Table of contents

Novel compounds with SMILES strings and IC_{50} values **S3 – S4**

Detailed synthesis including intermediates and precursors **S5 – S116**

NMR spectra of final compounds **S118 – S189**

HPLC traces of 39 & 45 **S190 – S191**

HRMS spectra of 39 & 45 **S192**

Selectivity profiling data **S193 – S208**

Thermal Shift Assay data of BI-D1870, 39, and 45 **S209**

NCI-2030 cellular toxicity data of BI-D1870, 39, and 45 **S210**

Novel compounds

Table S1: Novel MKK4 inhibitors and their biological activity. The compounds were tested in the [33]PanQinaseTM assay by Reaction Biology towards MKK4 and RSK4.

Compound_ID	SMILES	IC_{50} MKK4 [nM]	IC_{50} RSK4 [nM]
10	FC1=CC(NC2=NC(N(CCC(C)C)C(C)C(N3C)=O)=C3C=N2)=CC(F)=C1O	707 ±50.3	42 ±3
11	CC(C)CCN(C(C)C(N1C)=O)C2=C1C=NC(NC3=CC=CC=C3)=N2	33,450 ±9,380	n.d.
12	CC(C)CCN(C(C)C(N1C)=O)C2=C1C=NC(NC3=CC=C(O)C=C3)=N2	58,915 ±41,085	n.d.
13	CC(C)CCN(C(C)C(N1C)=O)C2=C1C=NC(NC3=CC(F)=CC(F)=C3)=N2	>100,000	n.d.
14	O=C(N1C)C(C)N(CCC(C)C)C2=C1C=NC(NC3=CC=C(O)C(F)=C3)=N2	2,145 ±274	n.d.
15	CC(C)CCN(C(C)C(N1C)=O)C2=C1C=NC(NC3=CC=C(C(O)=O)C=C3)=N2	2792 ±1,905	n.d.
16	O=C(N1C)C(C)N(CCC(C)C)C2=C1C=NC(NC3=C(F)C(F)=C(O)C(F)=C3F)=N2	73 ± 7.8	n.d.
17	O=C(N1C)C(C)N(CCC(C)C)C2=C1C=NC(NCC3=CC(F)=C(O)C(F)=C3)=N2	11,005 ±965	n.d.
18	CC(C)CCN(C(C)C(N1C)=O)C2=C1C=NC(NC3=CC(F)=C(O)C(F)=C3)=C2	390 ±0.5	n.d.
19	CC(C)CCN(C(C)C(N1C)=O)C2=C1C=CC(NC3=CC(F)=C(O)C(F)=C3)=N2	>100,000	n.d.
20	O=C(N1C)CN(CCC(C)C)C2=C1C=NC(NC3=CC(F)=C(O)C(F)=C3)=N2	53 ±0.3	12 ±0.6
21	CC(C)CCN(C(C)C(N1CC)=O)C2=C1C=NC(NC3=CC(F)=C(O)C(F)=C3)=N2	9,535 ±336	n.d.
22	FC1=CC(NC2=NC(N(CCC(C)C)C(C)CN3C)=C3C=N2)=CC(F)=C1O	669 ±29.6	n.d.
23	CN(C1=C(N(C2CCCCC2)C3)N=C(NC4=CC(F)=C(O)C(F)=C4)N=C1)C3=O	45 ±0.9	3 ±0
24	CN(C1=C(N(C2=CC=CC=C2)C3)N=C(NC4=CC(F)=C(O)C(F)=C4)N=C1)C3=O	189 ±8.1	41 ±5.9
25	FC1=C(O)C(F)=CC(NC2=CC(N(C)CC(N3C)=O)=C3C=N2)=C1	1,603 ±139	2,713 ±67.5
26	CCCN(CC(N1C)=O)C2=C1C=NC(NC3=CC(F)=C(O)C(F)=C3)=C2	2426 ±1	738 ±98.0
27	FC1=C(C(F)=CC(NC2=CC(N(C(C)C)C3)=C(C=N2)N(C)C3=O)=C1)O	12,230 ±250	3,991 ±413.5
28	FC1=CC(NC2=CC(N(C3CC3)CC(N4C)=O)=C4C=N2)=CC(F)=C1O	99 ±4.7	400 ±51.9
29	FC1=CC(NC2=CC(N(CC3CC3)CC(N4C)=O)=C4C=N2)=CC(F)=C1O	641 ±56	305 ±18.9
30	CC(C)CN(C1=C2C=NC(NC3=CC(F)=C(C(F)=C3)O)=C1)CC(N2C)=O	778 ±10.6	131 ±5.1
31	FC1=CC(NC2=CC(N(C3CCC3)CC(N4C)=O)=C4C=N2)=CC(F)=C1O	524 ±109	261 ±8.4
32	FC1=C(O)C(F)=CC(NC2=CC(N(CC(N3C)=O)C4CCCC4)=C3C=N2)=C1	672 ±62.7	85 ±0.5
33	CN(C1=C(N([C@H]2CC[C@@H](C)CC2)C3)C=C(NC4=CC(F)=C(O)C(F)=C4)N=C1)C3=O	1,190 ±89.5	17 ±0.4
34	FC1=C(O)C(F)=CC(NC2=CC(N(CC3=O)CC4CCCCC4)=C(N3C)C=N2)=C1	1,423 ±86.5	23 ±0.4
35	FC1=CC(NC2=CC(N(C3CCCCCC3)CC(N4C)=O)=C4C=N2)=CC(F)=C1O	195 ±17.5	11 ±2.3
36	CN(C1=C(N(C2CCCCC2)C3)C=C(NC4=CC(F)=C(C(O)=O)C(F)=C4)N=C1)C3=O	1,174 ±18.5	11.659 ±1,742
37	CN(C1=C(N(C2CCCCC2)C3)C=C(NC4=C(F)C(F)=C(O)C(F)=C4F)N=C1)C3=O	37 ±1.5	84 ±8.4
38	OC(C(F)=C1)=C(C#N)C=C1NC2=CC(N(C3CCCCC3)CC(N4C)=O)=C4C=N2	268 ±28.8	1802 ±71.5
39	FC1=CC(NC2=CC(N(C3CC3)CC(N4C)=O)=C4C=N2)=CC(C#N)=C1O	78 ±5.1	2,920 ±690
40	OC(C=C1)=C(C#N)C=C1NC2=CC(N(C3CC3)CC(N4C)=O)=C4C=N2	98 ±6.8	1,372 ±348
41	OC(C(F)=C1)=C(C(F)(F)F)C=C1NC2=CC(N(C3CC3)CC(N4C)=O)=C4C=N2	35 ±0.1	957 ±76.7
42	FC1=CC(NC2=CC(N(C3CC3)CC(N4C)=O)=C4C=N2)=CC(C(OC)=O)=C1O	73 ±5.0	1,930 ±184
43	ClC1=C(O)C(Cl)=CC(NC2=CC(N(C3CC3)CC(N4C)=O)=C4C=N2)=C1	72 ±5.4	750 ±4.4

**44**	OC(C=C1F)=CC(F)=C1NC2=CC(N(C3CC3)CC(N4C)=O)=C4C=N2	244 ±6.6	924 45.9
**45**	OC(C(Cl)=C1)=C(C#N)C=C1NC2=CC(N(C3CC3)CC(N4C)=O)=C4C=N2	22 ±4.1	1,033 ±162
**46**	CN(C1=C(N(C2CC2)C3)C=C(NC4=CC(C(F)(F)F)=C(O)C(C(F)(F)F)=C4)N=C1)C3=O	118 ±17.9	916 ±11.6

Detailed synthesis including intermediates and precursors

Synthesis of 2-((3,5-difluoro-4-hydroxyphenyl)amino)-8-isopentyl-5,7-dimethyl-7,8-dihydropteridin-6(5H)-one (BI-D1870) (10)

N-benzyl-3-methylbutan-1-amine

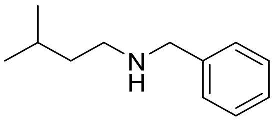

1-bromo-3-methylbutane (40.6 g, 0.2241 mol), benzylamine (144.1 g, 1.3 mol) and distilled water (41 mL) were stirred at 90 °C for 24 h. After this, sodium hydroxide (14 g) and brine were added. The water phase was extracted three times with diethyl ether, the organic phases were combined and dried over sodium sulfate. After evaporating the ether, the product was distilled at 112 °C (7 mbar), yielding 28.0 g (0.158 mol, 70 %) of a colorless oil. ESI-MS: *m/z* 178.2 $[M+H]^+$ (178.2 calcd.); ^{1}H NMR (400 MHz, DMSO-d_6) δ 7.34 – 7.18 (m, 5H), 3.67 (s, 2H), 2.48 (t, 2H), 1.60 (m, 1H), 1.36 – 1.28 (m, 2H), 0.84 (d, J = 6.6 Hz, 6H).

Ethyl *N*-benzyl-*N*-isopentylalaninate

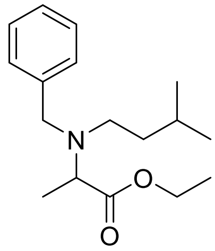

N-benzyl-3-methylbutan-1-amine (28.0 g, 0.155 mol), ethyl-2-bromopropanoate (29.5 g, 0.166 mol), potassium carbonate (34.2 g, 0.248 mol), and DMF (250 mL) were added to a dry flask and stirred at 110 °C for 3 h. After the reaction was cooled, it was filtered and the filtrate was evaporated. The residue was extracted with water and diethyl ether and evaporated to dryness, yielding 42.6 g (0.154 mol, 99 %) of a brown oil. The crude product was used without further purification. ESI-MS: *m/z* 278.4 $[M+H]^+$ (278.2 calcd.)

Ethyl isopentylalaninate

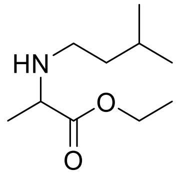

The crude mixture containing ethyl *N*-benzyl-*N*-isopentylalaninate (40.0 g, 0.144 mol) was dissolved in EtOH (430 mL) in a dry reactor. Palladium on charcoal (4.0 g, 10 %wt) and 37 % aq. hydrochloric acid (14.4 mL) was added. The reactor was purged with Argon and subsequently filled with H_2 up to 5 bar while stirring at room temperature until there was no loss in pressure anymore after 30 h. The mixture was poured over Celite and the residual ethanol was evaporated. The brown-yellow oil was poured over diethyl ether and was sonicated until a white precipitate formed. It was filtered and washed with diethyl ether yielding 21.5 g (0.115 mol, 80 %) of a white solid. ESI-MS: *m/z* 188.2 $[M+H]^+$ (188.2 calcd.); ^{1}H NMR (400 MHz, DMSO-

d6) δ 4.28 – 4.15 (m, 2H), 4.06 (q, J = 7.0 Hz, 1H), 3.35 (s, 1H), 2.96 – 2.80 (m, 2H), 1.71 – 1.51 (m, 3H), 1.49 (d, J = 7.2 Hz, 3H), 1.23 (t, J = 7.1 Hz, 3H), 0.86 (d, J = 7.7 Hz, 6H). ^{13}C NMR (101 MHz, DMSO-d6) δ 169.9, 62.3, 54.7, 44.1, 34.7, 25.8, 22.6, 14.9, 14.4.

Ethyl *N*-(2-chloro-5-nitropyrimidin-4-yl)-*N*-isopentylalaninate

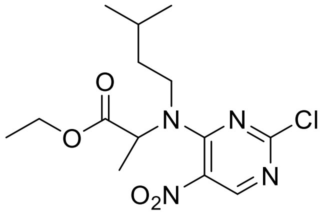

Ethyl isopentylalaninate (21.0 g, 0.112 mol) was dissolved in 200 mL water and 2,4-dichloro-5-nitropyrimidine (22.4 g, 0.116 mol) in 350 mL diethyl ether was added. The reaction mixture was cooled down to -10 °C and potassium carbonate (46.5 g, 0.336 mol) was added portion-wise. The mixture was stirred vigorously for 1 h at -5 °C, slowly warming up to room temperature. After 3 h the water phase was separated, and the organic phase was washed with water three times, dried over sodium sulfate, and evaporated to dryness yielding 32.6 g (0.095 mol, 84 %) of an orange oil. The product was used without further purification.

2-chloro-8-isopentyl-7-methyl-7,8-dihydropteridin-6(5H)-one

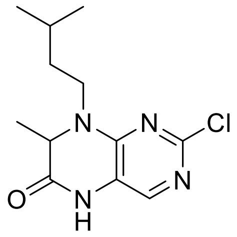

Ethyl *N*-(2-chloro-5-nitropyrimidin-4-yl)-*N*-isopentylalaninate (32.5 g, 0.094 mol) was dissolved in 400 mL glacial acetic acid. The mixture was heated to 70 °C. The heat source was

removed and Fe (27.6 g, 0.080 mol) was added. The temperature raised to 110 °C while stirring vigorously. After 20 min, the temperature decreased. When 70 °C was reached, the mixture was filtered hot and the filtrate was evaporated. The residue was mixed with 400 mL DCM and 42 mL of 37 % aq. HCl. The phases were separated and the aqueous phase was extracted with DCM. The combined organic layers were washed with an aqueous ammonia solution (25 %) and two times with brine. After drying over sodium sulfate, it was evaporated. The residue was sonicated in diethyl ether and a white-brown solid formed, that was filtered and evaporated to dryness yielding 13.42 g (0.047 mol, 53 %). HPLC-DAD: 254 nm: 99.6 %, 230 nm: 98.9 %; ESI-MS: *m/z* 267.2 $[M-H]^-$ (267.1 calcd.); Mp: 178 °C; ^1H NMR (400 MHz, DMSO-d_6) δ 10.76 (s, 1H), 7.55 (s, 1H), 4.24 (q, J = 6.5 Hz, 1H), 3.91 – 3.78 (m, 1H), 3.22 – 3.10 (m, 1H), 1.61 – 1.39 (m, 3H), 1.35 (d, J = 6.7 Hz, 3H), 0.99 – 0.82 (m, 6H). ^{13}C NMR (101 MHz, DMSO-d_6) δ 165.2, 152.3, 150.8, 137.7, 118.8, 55.9, 42.7, 39.5, 35.1, 25.5, 22.5, 22.3, 17.6.

2-chloro-8-isopentyl-5,7-dimethyl-7,8-dihydropteridin-6(5H)-one

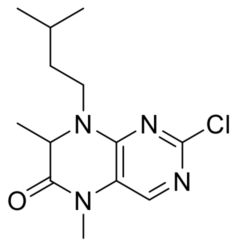

2-chloro-8-isopentyl-7-methyl-7,8-dihydropteridin-6(5H)-one (5.0 g, 18.6 mmol) and methyl iodide (10.6 g, 74.4 mmol) were dissolved in 51.8 mL DMA and cooled to –10 °C. Subsequently, 60 % sodium hydride on paraffin (0.83 g) was added while stirring for 2 h. HPLC confirmed a conversion into a new product and the reaction was quenched using ice and aq. ammonia (25 %). The reaction mixture was extracted three times with ethyl acetate. The combined organic layers were washed with brine, dried over sodium sulfate, loaded on Celite,

evaporated, and purified using Flash chromatography yielding 4.24 g (15.0 mmol, 81 %) of a white-brown solid. ESI-MS: *m/z* not detected $[M+H]^+$ (283.1 calcd.); ^1H NMR (400 MHz, DMSO-d_6) δ 7.85 (s, 1H), 4.36 (q, J = 6.8 Hz, 1H), 3.93 – 3.82 (m, 1H), 3.21 (s, 3H), 3.20 – 3.12 (m, 1H), 1.60 – 1.40 (m, 3H), 1.33 (d, J = 6.8 Hz, 3H), 0.93 – 0.88 (m, 6H).

2-((3,5-difluoro-4-hydroxyphenyl)amino)-8-isopentyl-5,7-dimethyl-7,8-dihydropteridin-6(5H)-one (BI-D1870) (10)

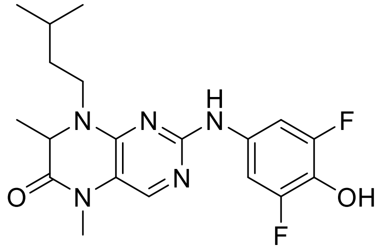

To 2-chloro-8-isopentyl-5,7-dimethyl-7,8-dihydropteridin-6(5H)-one (200 mg, 0.71 mmol) was added 4-amino-2,6-difluorophenol hydrochloride (257 mg, 1.41 mmol), Cs_2CO_3 (461 mg, 1.41 mmol), anhydrous 1,4-dioxane (3 mL) and t-BuOH (0.75 mL). The mixture was gassed with Ar while sonicating. Xantphos Pd G3 (13 mg, 0.014 mmol) was added, the tube was sealed and irradiated at 160 °C for 1 h in a microwave reactor. The reaction was cooled to room temperature, diluted with DCM, filtered and evaporated. The residue was purified using Flash chromatography giving a brown solid (91.8 mg, 33 %). HPLC-DAD: 254 nm: 95.3 %, 230 nm: 96.9 %; ESI-HRMS: *m/z* [M+H]+: 392.18926 calcd.; 392.18987 found; ^1H NMR (400 MHz, DMSO-d_6) δ 9.38 (d, J = 15.8 Hz, 1H), 9.15 (s, 1H), 7.80 (s, 1H), 7.49 – 7.37 (m, 2H), 4.25 (q, J = 6.7 Hz, 1H), 4.04 – 3.93 (m, 1H), 3.21 (s, 3H), 3.17 – 3.09 (m, 1H), 1.68 – 1.43 (m, 3H), 1.27 (d, J = 6.7 Hz, 3H), 0.94 – 0.88 (m, J = 7.7, 6.7 Hz, 6H). ^{13}C NMR (101 MHz, DMSO-d_6) δ 163.8, 155.3, 152.22 (dd, J = 237.7, 9.0 Hz), 150.5, 138.6, 133.19 (t, J = 12.8 Hz),

126.6 (t, J = 16.6 Hz), 114.6, 101.4 (dd, J = 18.1, 9.2 Hz), 56.1, 56.0, 42.9, 35.6, 27.7, 25.7, 22.5, 22.4, 18.6, 17.0. IR (ATR) [cm-1]: 3275, 2953, 2869, 1661, 1517, 1430, 1220, 1140, 1009, 773.

Synthesis of 8-isopentyl-5,7-dimethyl-2-(phenylamino)-7,8-dihydropteridin-6(5H)-one (11)

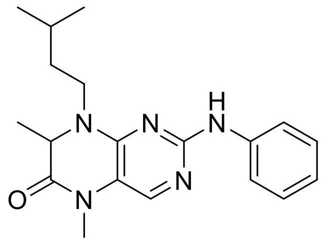

To a solution of 2-chloro-8-isopentyl-5,7-dimethyl-7,8-dihydropteridin-6(5H)-one (100 mg, 0.35 mmol) in EtOH (2 mL), water (8 mL) and conc. HCl (0.1 mL) was added aniline (66 mg, 0.71 mmol). The reaction was refluxed for 16 h. The reaction mixture was evaporated under reduced pressure and subsequently partitioned between $NAHCO_3$ and MeOH/DCM (1:3). The aqueous layer was separated and extracted 3x with MeOH/DCM (1:3). The combined organic layers were combined and dried over sodium sulfate and evaporated under reduced pressure and purified using Flash chromatography, yielding a brown solid (117 mg, 97 %). HPLC-DAD: 254 nm: 100 %, 230 nm: 98.1 %; ESI-HRMS: *m/z* [M+H]+: 340.21319 calcd.; 340.21348 found; ^{1}H NMR (400 MHz, DMSO-d_6) δ 9.09 (s, 1H), 7.80 (s, 1H), 7.74 (d, J = 8.1 Hz, 2H), 7.21 (t, J = 7.8 Hz, 2H), 6.87 (t, J = 7.2 Hz, 1H), 4.26 (q, J = 6.7 Hz, 1H), 4.07 – 3.97 (m, 1H), 3.23 (s, 3H), 3.18 – 3.08 (m, 1H), 1.68 – 1.46 (m, 3H), 1.28 (d, J = 6.7 Hz, 3H), 0.97 – 0.90 (m, 6H). ^{13}C NMR (101 MHz, DMSO-d_6) δ 163.8, 155.7, 150.5, 141.3, 138.7, 128.3, 120.4, 118.0, 114.3, 56.1, 42.9, 35.6, 27.7, 25.8, 22.5, 22.5, 17.0. IR (ATR) [cm-1]: 3262, 3190, 2955, 2928, 2870, 1664, 1576, 1436, 1261, 1230, 1165.

Synthesis of 8-isopentyl-5,7-dimethyl-2-(phenylamino)-7,8-dihydropteridin-6(5H)-one (12)

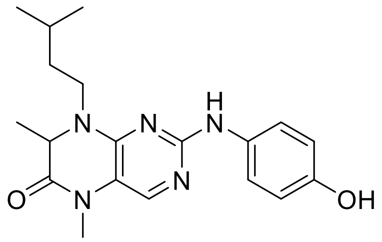

To a solution of 2-chloro-8-isopentyl-5,7-dimethyl-7,8-dihydropteridin-6(5H)-one (100 mg, 0.35 mmol) in EtOH (1 mL), water (4 mL) and conc. HCl (0.1 mL) was added 4-aminophenol (77 mg, 0.71 mmol). The reaction was refluxed for 16 h. The reaction mixture was evaporated under reduced pressure and subsequently partitioned between $NAHCO_3$ and MeOH/DCM (1:3). The aqueous layer was separated and extracted 3x with MeOH/DCM (1:3). The combined organic layers were combined and dried over sodium sulfate and evaporated under reduced pressure. A brown-grey solid formed, that was triturated with diethyl ether and filtered, yielding a grey solid (52 mg, 41 %). HPLC-DAD: 254 nm: 97.0 %, 230 nm: could not be determined; ESI-HRMS: *m/z* [M+H]+: 356.20810 calcd.; 356.20850 found; ^{1}H NMR (400 MHz, DMSO-d_6) δ 8.92 (s, 1H), 8.75 (s, 1H), 7.74 (s, 1H), 7.47 (d, J = 8.8 Hz, 2H), 6.64 (d, J = 8.8 Hz, 2H), 4.22 (q, J = 6.6 Hz, 1H), 4.05 – 3.93 (m, 1H), 3.21 (s, 3H), 3.14 – 3.02 (m, 1H), 1.66 – 1.44 (m, 3H), 1.26 (d, J = 6.7 Hz, 3H), 0.97 – 0.89 (m, 6H). ^{13}C NMR (101 MHz, DMSO-d_6) δ 164.2, 156.5, 152.0, 151.0, 139.4, 133.5, 120.5, 115.2, 114.2, 56.6, 43.2, 36.1, 28.2, 26.3, 23.0, 22.9, 17.3. IR (ATR) [cm-1]: 3275, 2954, 2870, 1647, 1607, 1433, 1256, 1214, 1164.

2-((3,5-difluorophenyl)amino)-8-isopentyl-5,7-dimethyl-7,8-dihydropteridin-6(5H)-one (13)

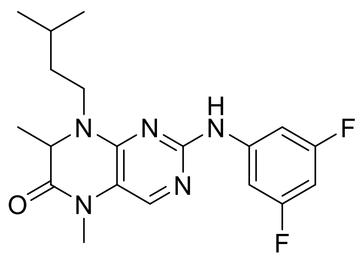

To a solution of 2-chloro-8-isopentyl-5,7-dimethyl-7,8-dihydropteridin-6(5H)-one (100 mg, 0.35 mmol) in EtOH (1 mL), water (4 mL) and conc. HCl (0.1 mL) was added 3,5-difluoroaniline (91 mg, 0.71 mmol). The reaction was refluxed for 16 h. The reaction mixture was evaporated under reduced pressure and subsequently partitioned between $NAHCO_3$ and MeOH/DCM (1:3). The aqueous layer was separated and extracted 3x with MeOH/DCM (1:3). The combined organic layers were combined and dried over sodium sulfate, evaporated under reduced pressure and purified using Flash chromatography, yielding a white solid (25 mg, 19 %). HPLC-DAD: 254 nm: 98.4 %, 230 nm: 98.0 %; ESI-HRMS: *m/z* [M+H]+: 376.19434 calcd.; 376.19499 found; ^{1}H NMR (400 MHz, MeOD) δ 7.76 (s, 1H), 7.35 (d, 2H), 6.47 – 6.37 (m, J = 9.1, 2.2 Hz, 1H), 4.28 (q, J = 6.8 Hz, 1H), 4.18 – 4.02 (m, 1H), 3.23 – 3.16 (m, 1H), 1.77 – 1.53 (m, 3H), 1.38 (d, J = 6.8 Hz, 3H), 1.00 – 0.91 (m, 6H). ^{13}C NMR (101 MHz, DMSO-d_6) δ 157.9, 135.5 (dd, J = 241.1, 15.6 Hz), 147.4, 143.1, 135.5 (t, J = 13.7 Hz), 129.6, 107.2, 92.5 (dd, J = 20.4, 8.7 Hz), 87.0 (t, J = 26.4 Hz), 48.8, 35.4, 27.6, 19.1, 17.9, 13.5, 13.4, 8.0. IR (ATR) [cm-1]: 3272, 2954, 2932, 2870, 1669, 1598, 1576, 1427, 1250, 1147, 1108.

Synthesis of 8-isopentyl-5,7-dimethyl-2-(phenylamino)-7,8-dihydropteridin-6(5H)-one (14)

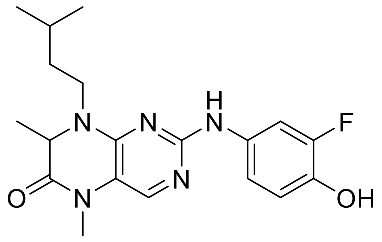

To 2-chloro-8-isopentyl-5,7-dimethyl-7,8-dihydropteridin-6(5H)-one (85 mg, 0.30 mmol) was added 4-amino-2-fluorophenol (76 mg, 0.60 mmol), Cs_2CO_3 (196 mg, 0.60 mmol), anhydrous 1,4-dioxane (2 mL) and t-BuOH (0.5 mL). The mixture was gassed with Ar while sonicating. Xantphos Pd G3 (6 mg, 0.006 mmol) was added, the tube was sealed and irradiated at 160 °C for 1 h in a microwave reactor. The reaction was cooled to room temperature, diluted with DCM, filtered and evaporated. The residue was purified using Flash chromatography giving a beige solid (54 mg, 48 %). HPLC-DAD: 254 nm: 97.7 %, 230 nm: 97.4 %; ESI-HRMS: *m/z* [M+H]+: 374.19868 calcd.; 374.19898 found; 1H NMR (400 MHz, DMSO) δ 8.96 (s, 1H), 8.15 (s, 1H), 7.79 (s, 1H), 7.72 – 7.62 (m, 1H), 7.25 – 7.16 (m, 1H), 6.84 – 6.76 (m, 1H), 4.23 (q, J = 6.7 Hz, 1H), 4.04 – 3.93 (m, 1H), 3.21 (s, 1H), 3.15 – 3.06 (m, 1H), 1.68 – 1.43 (m, 3H), 1.26 (d, J = 6.7 Hz, 1H), 0.97 – 0.85 (m, 6H). 13C NMR (101 MHz, DMSO) δ 164.2, 163.6, 156.1, 152.1, 151.0, 149.7, 139.1, 138.8, 138.7, 134.2, 134.1, 117.8, 117.8, 114.8, 114.8, 114.6, 107.2, 107.0, 56.6, 43.3, 36.1, 28.2, 26.2, 22.9, 17.4. IR (ATR) [cm-1]: 3267, 2954, 1647, 1431, 1248, 1232, 1060, 972.

Synthesis of 4-((8-isopentyl-5,7-dimethyl-6-oxo-5,6,7,8-tetrahydropteridin-2-yl)amino)benzoic acid (15)

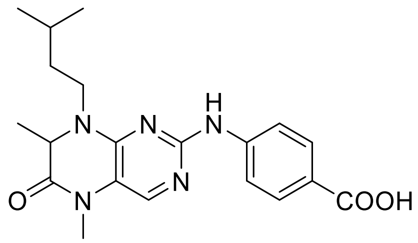

To a solution of 2-chloro-8-isopentyl-5,7-dimethyl-7,8-dihydropteridin-6(5H)-one (100 mg, 0.35 mmol) in EtOH (2 mL), water (8 mL) and conc. HCl (0.2 mL) was added 4-aminobenzoic acid (97 mg, 0.71 mmol). The reaction was refluxed for 32 h. The product precipitated from the reaction mixture as a white solid. It was filtered, and washed with water and diethyl ether. The residue was dissolved in methanol, loaded on silica gel and purified using Flash chromatography giving the titled product as a white solid (64 mg, 47 %). HPLC-DAD: 254 nm: 100 %, 230 nm: 100 %; ESI-HRMS: *m/z* [M+H]+: 384,20302 calcd.; 384.20360 found; ^{1}H NMR (400 MHz, DMSO-d$_6$) δ 12.46 (s, 1H), 9.57 (s, 1H), 7.83 (dd, J = 21.4, 8.5 Hz, 5H), 4.29 (q, J = 6.6 Hz, 1H), 4.08 – 3.97 (m, 1H), 3.24 (s, 3H), 3.21 – 3.12 (m, 1H), 1.70 – 1.47 (m, 3H), 1.30 (d, J = 6.7 Hz, 3H), 1.00 – 0.90 (m, 6H). ^{13}C NMR (101 MHz, DMSO-d$_6$) δ 167.2, 163.9, 155.1, 150.5, 145.6, 138.5, 130.2, 121.9, 116.7, 115.1, 56.2, 43.1, 35.5, 27.8, 25.9, 22.5, 22.5, 17.2. IR (ATR) [cm-1]: 3293, 2950, 2867, 2376, 2344, 1664, 1598, 1424, 1235.

Synthesis of 8-isopentyl-5,7-dimethyl-2-((2,3,5,6-tetrafluoro-4-hydroxyphenyl)amino)-7,8-dihydropteridin-6(5H)-one (16)

1-(benzyloxy)-2,3,5,6-tetrafluoro-4-nitrobenzene

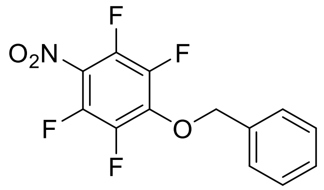

K_2CO_3 (2.67 g, 19.3 mmol) was added to a solution of 1,2,3,4,5-pentafluoro-6-nitrobenzene (**57**) (2.06 g, 9.7 mmol) and benzyl alcohol (1.05 g, 9.7 mmol) in DMF (13 mL). The resultant mixture was stirred at rt overnight. After the addition of water, a precipitate formed, that was filtered and washed with water yielding a brown solid (1.34 g, 46 %). 1H NMR (400 MHz, DMSO) δ 7.51 – 7.34 (m), 5.49 (s, 2H).

4-(benzyloxy)-2,3,5,6-tetrafluoroaniline

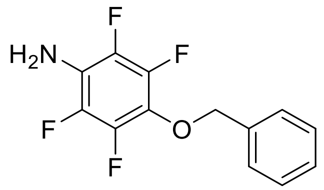

1-(benzyloxy)-2,3,5,6-tetrafluoro-4-nitrobenzene (2.0 g, 6.6 mmol) and NH_4Cl (3.2 g, 59.8 mmol) was dissolved in EtOH and refluxed. Powdered iron (1.11 g, 19.9 mmol) was added portion wise. The reaction was stirred for 2 h and cooled to room temperature. After filtration, the solvent was evaporated and the residue was taken up in EtOAc. The organic phase was washed with water and brine, dried over sodium sulfate and evaporated giving the titled product as brown crystals (526 mg, 29 %). 1H NMR (400 MHz, DMSO) δ 7.42 – 7.34 (m, 5H), 5.66 (s, 2H), 5.04 (s, 2H).

2-((4-(benzyloxy)-2,3,5,6-tetrafluorophenyl)amino)-8-isopentyl-5,7-dimethyl-7,8-dihydropteridin-6(5H)-one

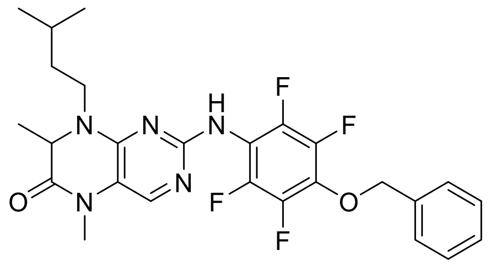

2-chloro-8-isopentyl-5,7-dimethyl-7,8-dihydropteridin-6(5H)-one (200 mg, 0.71 mmol), 4-(benzyloxy)-2,3,5,6-tetrafluoroaniline (221 mg, 0.81 mmol), Cs_2CO_3 (461 mg, 1.4 mmol) and Xantphos Pd G3 (13 mg, 0.014 mmol) were added to a dry flask with a reflux condenser in an argon atmosphere. Anhydrous 1,4-dioxane (11 mL) was added. The mixture was refluxed for 3 h. The reaction was cooled to room temperature, diluted with water, extracted with EtOAc, washed with brine and dried over sodium sulfate. The residue was purified using Flash chromatography giving a brown material (239 mg, 65 %). 1H NMR (400 MHz, DMSO) δ 8.74 (s, 1H), 7.71 (s, 1H), 7.48 – 7.35 (m, 5H), 5.26 (s, 2H), 4.20 (q, J = 6.7 Hz, 1H), 3.72 – 3.61 (m, 1H), 3.18 (s, 3H), 3.01 – 2.91 (m, 1H), 1.59 – 1.35 (m, 3H), 1.24 (d, J = 6.8 Hz, 3H), 0.78 (t, J = 5.4 Hz, 6H).

8-isopentyl-5,7-dimethyl-2-((2,3,5,6-tetrafluoro-4-hydroxyphenyl)amino)-7,8-dihydropteridin-6(5H)-one (16)

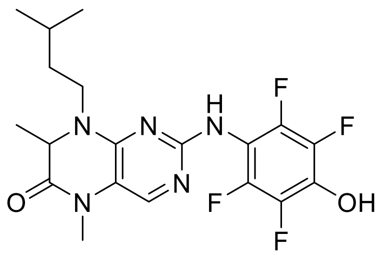

2-((4-(benzyloxy)-2,3,5,6-tetrafluorophenyl)amino)-8-isopentyl-5,7-dimethyl-7,8-dihydropteridin-6(5H)-one (230 mg, 0.44 mmol) was dissolved in EtOH (10 mL) and conc. HCl (0.25 mL). Pd/C (23 mg) was added. The mixture was stirred for 24 h in a hydrogen atmosphere. After full conversion, the reaction mixture was filtered over celite, evaporated and purified using Flash chromatography yielding transparent brownish crystals (106 mg, 56 %). HPLC-DAD: 254 nm: 99.3 %, 230 nm: 98.6 %; ESI-HRMS: *m/z* [M+H]+: 428.17041 calcd.; 428.17059 found; 1H NMR (400 MHz, DMSO) δ 11.20 (s, 1H), 8.46 (s, 1H), 7.69 (s, 1H), 4.19 (q, J = 6.7 Hz, 1H), 3.70 – 3.58 (m, 1H), 3.18 (s, 3H), 3.01 – 2.91 (m, 1H), 1.43 – 1.27 (m, 3H), 1.24 (d, J = 6.8 Hz, 3H), 0.77 (d, J = 4.8 Hz, 6H). 13C NMR (101 MHz, DMSO) δ 163.69, 156.54, 150.65, 138.47, 114.69, 56.21, 42.86, 35.44, 27.73, 25.63, 22.17, 22.04, 17.30. IR (ATR) [cm-1]: 2953, 1669, 1663, 1570, 1476, 1436, 1430, 1327, 1244, 1011, 969.

Synthesis of 2-((3,5-difluoro-4-hydroxybenzyl)amino)-8-isopentyl-5,7-dimethyl-7,8-dihydropteridin-6(5H)-one (17)

3,5-difluoro-4-(methoxymethoxy)benzonitrile

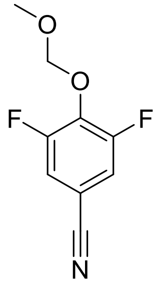

3,5-difluoro-4-hydroxybenzonitrile (**62**) (3.0 g, 19.3 mmol) was dissolved in DMF (15 mL) and cooled to 0 °C. DIPEA (1.2 g, 31.0 mmol) was added and stirred. Bromomethyl methyl ether (1.7 mL, 21.3 mmol) was added slowly to the reaction mixture. After 2 h, the reaction was stopped by addition of water and saturated sodium bicarbonate solution. The mixture was stirred for 15 min and extracted 3x with EtOAc, washed with brine, dried over sodium sulfate and evaporated, yielding a clean yellow oil (3.2 g 83 %). 1H NMR (400 MHz, DMSO) δ 7.93 – 7.84 (m, 2H), 5.27 (s, 2H), 3.48 (s, 3H).

(3,5-difluoro-4-(methoxymethoxy)phenyl)methanamine

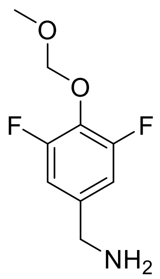

Cobalt dichloride monohydrate was dried until its color turned blue indicating it is free of water. Anhydrous $CoCl_2$ (1.5 g, 11.5 mmol) and 3,5-difluoro-4-(methoxymethoxy)benzonitrile (1.15 g, 5.8 mmol) were mixed in methanol (10 mL) and stirred. $NaBH_4$ (2.18 g, 57.7 mmol) was added portion wise. After conversion, aq. ammonia solution (25 %) was added. The mixture was filtered and extracted 3x with EtOAc. The solvent was evaporated, yielding a yellow oil (650 mg, 3.2 mmol). 1H NMR (400 MHz, DMSO) δ 8.66 (s, 3H), 7.44 – 7.37 (m, 2H), 5.15 (d, J = 5.4 Hz, 2H), 3.99 (d, J = 5.4 Hz, 2H), 3.47 (s, 3H).

2-((3,5-difluoro-4-(methoxymethoxy)benzyl)amino)-8-isopentyl-5,7-dimethyl-7,8-dihydropteridin-6(5H)-one

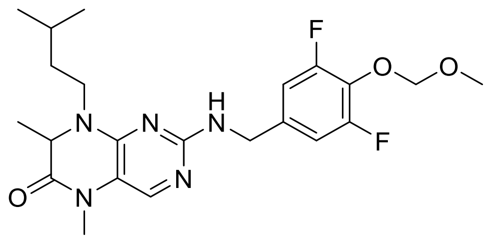

2-chloro-8-isopentyl-5,7-dimethyl-7,8-dihydropteridin-6(5H)-one (450 mg, 1.59 mmol), (3,5-difluoro-4-(methoxymethoxy)phenyl)methanamine (572 mg, 2.4 mmol), KOtBu (250 mg, 2.2 mmol) and Brettphos Pd G3 (29 mg, 0.03 mmol) were added to a dry flask in an argon atmosphere. Anhydrous 1,4-dioxane (25 mL) was added and the reaction was refluxed for 2 h. DCM was added and filtered over a Celite pad, evaporated and purified using Flash chromatography, giving a yellow solid (404 mg, 56 %) that was used without further purification.

2-((3,5-difluoro-4-hydroxybenzyl)amino)-8-isopentyl-5,7-dimethyl-7,8-dihydropteridin-6(5H)-one (17)

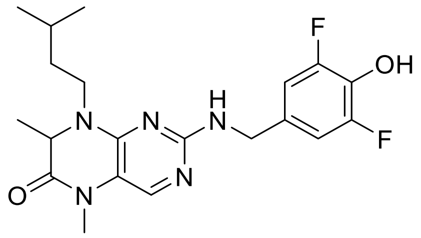

Conc. HCl (0.7 mL) was added to 2-((3,5-difluoro-4-(methoxymethoxy)benzyl)amino)-8-isopentyl-5,7-dimethyl-7,8-dihydropteridin-6(5H)-one (400 mg, 0.89 mmol) in MeOH (12 mL). The mixture was stirred at room temperature for 3 h. It was extracted 3x with EtOAc. The combined organic layers were washed with water, brine, dried over sodium sulfate, evaporated and purified using Flash chromatography giving a brown solid (292 mg, 81 %). HPLC-DAD: 254 nm: 97.3 %, 230 nm: 97.1 %; ESI-HRMS: *m/z* [M+H]+: 406.20491 calcd.; 406.20544 found; 1H NMR (400 MHz, DMSO) δ 9.91 (s, 1H), 7.62 (s, 1H), 7.20 (s, 1H), 6.97 – 6.84 (m, 2H), 4.32 (ddd, J = 51.0, 15.7, 6.3 Hz, 2H), 4.16 (q, J = 6.7 Hz, 1H), 3.89 (s, 1H), 3.17 (d, J = 7.8 Hz, 3H), 3.05 – 2.95 (m, 1H), 1.52 – 1.30 (m, 3H), 1.23 (d, J = 6.7 Hz, 3H), 0.87 – 0.77 (m, 6H). 13C NMR (101 MHz, DMSO) δ 163.55, 157.56,152.10 (dd, J = 241.4, 7.1 Hz), 150.83, 137.59, 132.24 – 132.03 (m), 131.78 (t, J = 16.3 Hz), 113.27, 110.00 (dd, J = 14.8, 6.8 Hz), 56.06, 43.28, 35.49, 27.68, 25.51, 22.28, 16.97. IR (ATR) [cm-1]: 3356, 2953, 2868, 1661, 1574, 1519, 1011, 779.

Synthesis of 7-((3,5-difluoro-4-hydroxyphenyl)amino)-1-isopentyl-2,4-dimethyl-1,4-dihydropyrido[3,4-b]pyrazin-3(2H)-one (28)

Ethyl *N*-(2-chloro-5-nitropyridin-4-yl)-*N*-isopentylalaninate

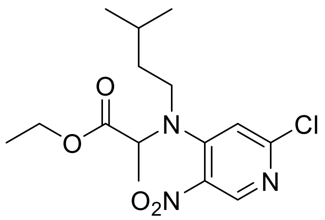

Ethyl isopentylalaninate (2.2 g, 11.7 mmol) was dissolved in water (20 mL). 2,4-dichloro-5-nitrpyridine (2.49 g, 12.9 mmol) in diethyl ether (40 mL) was added slowly. K_2CO_3 was added portionwise and the reaction was stirred for 16 h at rt. Upon completion, the phases were separated, the aqueous phase was extracted 3x with diethyl ether. The combined organic layers were dried over sodium sulfate, evaporated and purified using Flash chromatography giving the desired product as a crude orange oil that was used without further purification. ESI-MS: *m/z* 344.3 $[M+H]^+$ (344.2 calcd.).

6-chloro-4-isopentyl-3,4-dihydropyrido[2,3-b]pyrazin-2(1H)-one

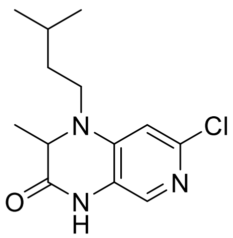

Ethyl *N*-(2-chloro-5-nitropyridin-4-yl)-N-isopentylalaninate (5.0 g, 14.6 mmol) was dissolved in glacial acetic acid and heated to 70 °C. After removal of the heat source, 30 g of iron was added in portions. The temperature raised to 110 °C. The reaction mixture was cooled to 90 °C

and stirred for 20 min at this temperature. The mixture was subsequently filtered and the filtrate was evaporated. The pH was set to 5-7 with NaHCO3 solution. The iron hydroxide was filtered off. The phases are separated. The organic phase was washed with water and brine, dried over MgSO4 and evaporated to dryness. The product was precipitated from ether yielding a dark brown oil that was purified using flash chromatography giving the desired product as a brown oil (2.14 g, 55 %). 1H NMR (400 MHz, DMSO) δ 10.92 (s, 1H), 7.66 (s, 1H), 6.80 (s, 1H), 4.10 (q, J = 6.6 Hz, 1H), 3.58 – 3.47 (m, 1H), 3.24 – 3.11 (m, 1H), 1.67 – 1.55 (m, 1H), 1.51 – 1.37 (m, 2H), 1.24 (d, J = 6.7 Hz, 3H), 0.94 – 0.86 (m, 6H).

7-chloro-1-isopentyl-2,4-dimethyl-1,4-dihydropyrido[3,4-b]pyrazin-3(2H)-one

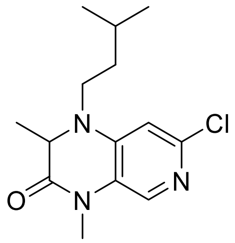

7-chloro-1-isopentyl-2-methyl-1,4-dihydropyrido[3,4-b]pyrazin-3(2H)-one (2.14 g, 8.0 mmol) and methyl iodide (4.54 g, 32 mmol) were added to DMF (22 mL) and cooled to -10 °C. Subsequently, sodium hydride (60% on paraffin oil, 639 mg, 16 mmol) was added while stirring for 2 h. TLC confirmed conversion and the reaction was quenched with ice and aq. ammonia (25%). The mixture was extracted with EtOAc (3x). The combined organic layers were dried over sodium sulfate, filtered, evaporated and purified using Flash chromatography yielding a dark brown thick oil (1.76 g, 78 %). 1H NMR (400 MHz, DMSO) δ 7.85 (s, 1H), 6.70 (s, 1H), 4.16 (q, J = 6.8 Hz, 1H), 3.52 – 3.43 (m, 1H), 3.28 (s, 3H), 3.17 – 3.08 (m, 1H), 1.65 – 1.54 (m, 1H), 1.48 – 1.40 (m, 2H), 1.14 (d, J = 6.8 Hz, 3H), 0.94 – 0.87 (m, 6H).

2-(benzyloxy)-1,3-difluoro-5-nitrobenzene

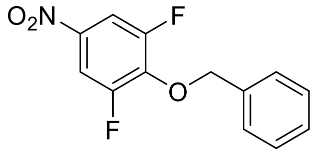

Potassium carbonate (36.6 g, 265 mmol) was added to a solution of 1,2,3-trifluoro-5-nitrobenzene (23.5 g, 132 mmol) and benzyl alcohol (36.3 g, 205 mmol) in DMF (150 mL). The resultant mixture was stirred at room temperature overnight. Subsequently, the reaction mixture was concentrated under reduced pressure and the residue was partitioned between ethyl acetate and water. A precipitate formed, that was washed with pentane and diethyl ether yielding 25.0 g (94.2 mmol, 71 %) of a yellow solid. HPLC-DAD: 254 nm: 97.0 %, 230 nm: 92.5 %; ESI-MS: *m/z* not detected $[M+H]^+$ (266.1 calcd.); ^1H NMR (400 MHz, $CDCl_3$) δ 7.82 (d, J = 7.8 Hz, 2H), 7.39 (m, 5H), 5.36 (s, 2H).

4-(benzyloxy)-3,5-difluoroaniline

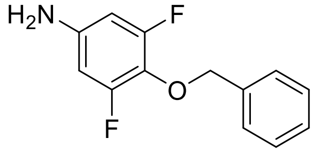

2-(benzyloxy)-1,3-difluoro-5-nitrobenzene (25.0 g, 94.3 mmol) and ammonium chloride (45.4 g, 848 mmol) were dissolved in 70 % v/v ethanol in water (800 mL). Iron powder (75 g, 283 mmol) was added and the mixture was refluxed. After 16 h, the mixture was cooled to room temperature, filtered over Celite, and evaporated. The residue was dissolved in DCM, extracted three times with water and brine, dried over sodium sulfate and evaporated to dryness, yielding 20.1 g (85.5 mmol, 91 %) of a brown solid. HPLC-DAD: 254 nm: 93.8 %, 230 nm: 95.8 %; ESI-MS: *m/z* not detected $[M+H]^+$ (236.1 calcd.); ^1H NMR (400 MHz, DMSO-d_6) δ 7.41 – 7.30 (m, 5H), 6.24 – 6.15 (m, 2H), 5.41 (s, 2H), 4.91 (s, 2H). ^{13}C NMR (101 MHz, DMSO-d_6) δ 157.6,

157.5, 155.2, 155.1, 145.9, 145.7, 145.6, 136.8, 128.3, 128.3, 128.2, 123.8, 123.6, 97.0, 96.9, 96.8, 96.8, 75.9.

7-((4-(benzyloxy)-3,5-difluorophenyl)amino)-1-isopentyl-2,4-dimethyl-1,4-dihydropyrido[3,4-b]pyrazin-3(2H)-one

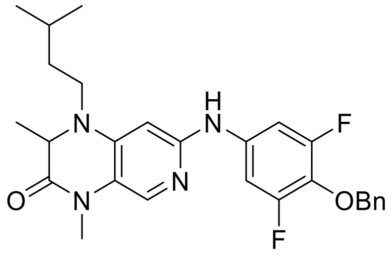

7-chloro-1-isopentyl-2,4-dimethyl-1,4-dihydropyrido[3,4-b]pyrazin-3(2H)-one (250 mg, 0.89 mmol), 4-(benzyloxy)-3,5-difluoroaniline (418 mg, 1.78 mmol), Cs_2CO_3 (580 mg, 1.79 mmol) and Pd G3 Xantphos (17 mg, 0.018 mmol) was added to a dry flask with a reflux condenser in an argon atmosphere. Anhydrous 1,4-dioxane/tBuOH (3:1, 19 mL) was added. The mixture was stirred to reflux for 3 h. The reaction was cooled to room temperature, diluted with water, extracted with EtOAc, washed with brine and dried over sodium sulfate. The residue was purified using Flash chromatography giving the titled product as a yellow solid (422 mg, 0.88 mmol, 99 %). ESI-MS: *m/z* 481.2 $[M+H]^+$ (481.2 calcd.); 1H NMR (400 MHz, DMSO) δ 9.78 (s, 1H), 7.64 (s, 1H), 7.44 – 7.34 (m, 5H), 7.30 – 7.21 (m, 2H), 6.17 (s, 1H), 5.09 (d, J = 12.2 Hz, 2H), 4.24 (q, J = 6.7 Hz, 1H), 3.44 – 3.35 (m, 3H), 3.25 (s, 3H), 1.63 – 1.39 (m, 3H), 1.23 (d, J = 6.8 Hz, 3H), 0.93 – 0.86 (m, 6H).

7-((3,5-difluoro-4-hydroxyphenyl)amino)-1-isopentyl-2,4-dimethyl-1,4-dihydropyrido[3,4-b]pyrazin-3(2H)-one (18)

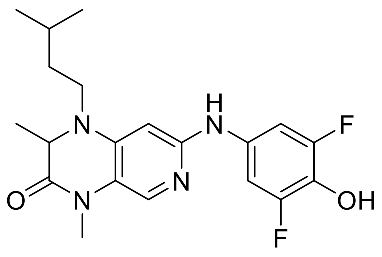

7-((4-(benzyloxy)-3,5-difluorophenyl)amino)-1-isopentyl-2,4-dimethyl-1,4-dihydropyrido[3,4-b]pyrazin-3(2H)-one (420 mg, 0.87 mmol) was dissolved in EtOH (10 mL). Pd/C (42 mg) was added. The mixture was stirred for 24 h in a hydrogen atmosphere. After full conversion, the reaction mixture was filtered over a Celite pad, evaporated, purified using Flash chromatography and recrystallized from toluene yielding a white solid (79 mg, 0.2 mmol, 23 %). HPLC-DAD: 254 nm: 100 %, 230 nm: 100 %; ESI-HRMS: *m/z* [M+H]+: 391.19401 calcd.; 391.19443 found; 1H NMR (400 MHz, DMSO) δ 9.30 (s, 1H), 8.76 (s, 1H), 7.73 (s, 1H), 7.40 – 7.30 (m, 2H), 6.04 (s, 1H), 4.06 (q, J = 6.7 Hz, 1H), 3.25 (s, 3H), 3.09 (dt, J = 8.5, 7.6 Hz, 1H), 1.67 – 1.55 (m, 1H), 1.53 – 1.42 (m, 2H), 1.10 (d, J = 6.7 Hz, 3H), 0.98 – 0.87 (m, 6H). 13C NMR (101 MHz, DMSO) δ 165.44, 153.61, 153.52, 152.70, 151.25, 151.16, 142.12, 134.38, 131.58, 125.98, 125.82, 119.94, 100.99, 100.90, 100.81, 100.73, 91.86, 57.25, 45.15, 34.97, 28.10, 25.69, 22.44, 22.36, 14.77. IR (ATR) [cm-1]: 3209, 2955, 1646, 1609, 1476, 1436, 1219, 1144, 1011.

Synthesis of (*S*)-6-((3,5-difluoro-4-hydroxyphenyl)amino)-4-isopentyl-1,3-dimethyl-3,4-dihydropyrido[2,3-b]pyrazin-2(1H)-one (19)

tert-butyl-(*S*)-(1-((2,6-dichloropyridin-3-yl)amino)-1-oxopropan-2-yl)carbamate

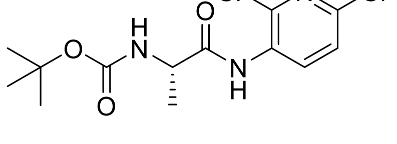

2,6-dichloropyridin-3-amine (4.31 g, 26.4 mmol) and Boc-*L*-alanine (5.0 g, 26.4 mmol) were dissolved in pyridine (35 mL) and the solution was cooled to 0 °C. Propylphosphonic acid (50 % EtOAc solution, 20 mL) was added and the reaction was stirred for 16 h. Upon completion, the reaction was added to ice water and basified using Na_2CO_3, and extracted with EtOAc (3x). Combined organic layers were dried over sodium sulfate, filtered, evaporated and purified using Flash chromatography giving the titled product as a white solid (7.69 g, 87 %). 1H NMR (400 MHz, DMSO) δ 9.61 (s, 1H), 8.30 (d, J = 8.4 Hz, 1H), 7.57 (d, J = 8.4 Hz, 1H), 7.29 (d, J = 6.6 Hz, 1H), 4.29 – 4.17 (m, 1H), 1.39 (s, 9H), 1.29 (d, J = 7.2 Hz, 3H).

(*S*)-2-amino-N-(2,6-dichloropyridin-3-yl)propenamide

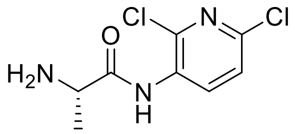

tert-butyl-(*S*)-(1-((2,6-dichloropyridin-3-yl)amino)-1-oxopropan-2-yl)carbamate (5.0 g, 15.0 mmol) was added to TFA 10 % in DCM (200 mL) and stirred at rt for 2 h. The reaction was basified using sat. $NaHCO_3$ solution. The mixture was extracted 3x with EtOAc. The combined organic layers were dried over sodium sulfate, filtered and evaporated giving an orange oil (1.3 g, 37 %) that was used without further purification.

(*S*)-N-(2,6-dichloropyridin-3-yl)-2-(isopentylamino)propenamide

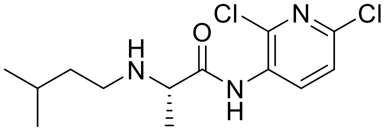

(*S*)-2-amino-N-(2,6-dichloropyridin-3-yl)propenamide (2.1 g, 9.0 mmol), NaOAc (1.47 g, 17.9 mmol) and isovaleraldehyde (1.16 g, 13.5 mmol) were mixed in DCE (40 mL) in a dry flask. The mixture was stirred for 10 min. Subsequently, sodium triacetoxy borohydride (3.8 g, 17.9 mmol) was added portion wise and stirred for 16 h under Ar atmosphere. The reaction was quenched with saturated $NaHCO_3$ solution and stirred for 10 min. The phases were separated and the aqueous phase was extracted with ether. The combined organic layers were washed again with water, dried over sodium sulfate, and the solvent was evaporated yielding a brown oil (1.15 g, 42 %). ESI-MS: *m/z* 304.2 $[M+H]^+$ (304.1 calcd.); 1H NMR (400 MHz, DMSO) δ 8.08 (d, J = 8.2 Hz, 1H), 7.71 (d, J = 8.2, 1H), 3.38 (q, J = 6.8 Hz, 1H), 2.84 – 2.69 (m, 2H), 1.70 – 1.58 (m, 1H), 1.45 – 1.31 (m, 4H), 1.29 (d, J = 6.9 Hz, 3H), 0.81 – 0.76 (m, 6H).

(*S*)-6-chloro-4-isopentyl-3-methyl-3,4-dihydropyrido[2,3-b]pyrazin-2(1H)-one

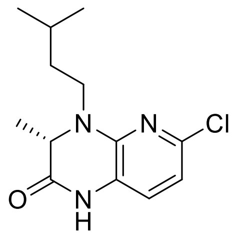

(*S*)-N-(2,6-dichloropyridin-3-yl)-2-(isopentylamino)propenamide (1.13 g, 3.71 mmol) was dissolved in DMF (37 mL) and DIPEA (3.83 g, 29.7 mmol) was added. Temperature was increased to 160 °C and the mixture was stirred for 48 h. Upon completion, the reaction was cooled, diluted with water and extracted with DCM, dried over sodium sulfate filtered and evaporated giving the titled product as a brown solid (950 mg, 96 %). ESI-MS: *m/z* 265.9 $[M+H]^+$ (266.1 calcd.); 1H NMR (400 MHz, DMSO) δ 10.59 (s, 1H), 6.96 (d, J = 7.8 Hz, 1H), 6.62 (d, J = 7.8 Hz, 1H), 4.08 (q, J = 6.7 Hz, 1H), 3.90 – 3.81 (m, 1H), 3.09 – 2.97 (m, 1H), 1.64 – 1.39 (m, 3H), 1.20 (d, J = 6.8 Hz, 3H), 0.94 – 0.86 (m, 6H).

(*S*)-6-chloro-4-isopentyl-1,3-dimethyl-3,4-dihydropyrido[2,3-b]pyrazin-2(1H)-one

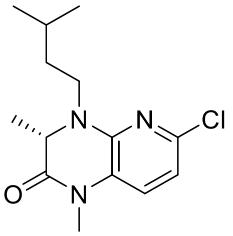

(*S*)-6-chloro-4-isopentyl-3-methyl-3,4-dihydropyrido[2,3-b]pyrazin-2(1H)-one (1.0 g, 3.7 mmol) and methyl iodide (2.12 g, 14.9 mmol) were added to DMF (10 mL) and cooled to 0 °C. Subsequently, sodium hydride (60 % on paraffin oil, 300 mg, 7.5 mmol) was added while stirring for 2 h. TLC confirmed a conversion into a new product and the reaction was quenched ice and aq. ammonia (25%). It was extracted with EtOAc (3x). The combined organic layers were dried over sodium sulfate, filtered, evaporated and purified using Flash chromatography yielding a brown solid (560 mg, 53 %). 1H NMR (400 MHz, DMSO) δ 7.26 (d, J = 8.1 Hz, 1H), 6.73 (d, J = 8.0 Hz, 1H), 4.21 (q, J = 6.7 Hz, 1H), 3.93 – 3.83 (m, 1H), 3.22 (s, 3H), 3.09 – 2.98 (m, 1H), 1.60 – 1.42 (m, 3H), 1.16 (d, J = 6.7 Hz, 3H), 0.93 – 0.87 (m, 6H).

(*S*)-6-((4-(benzyloxy)-3,5-difluorophenyl)amino)-4-isopentyl-1,3-dimethyl-3,4-dihydropyrido[2,3-b]pyrazin-2(1H)-one

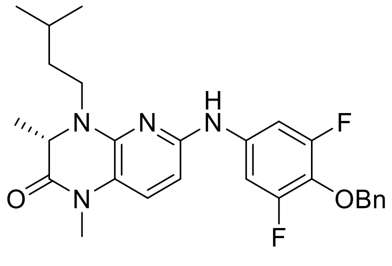

(*S*)-6-chloro-4-isopentyl-1,3-dimethyl-3,4-dihydropyrido[2,3-b]pyrazin-2(1H)-one (530 mg, 1.89 mmol) , 4-(benzyloxy)-3,5-difluoroaniline (510 mg, 2.17 mmol), Cs_2CO_3 (1.23 g, 3.77 mmol) and Pd G3 Xantphos (35 mg, 0.038 mmol) was added to a dry flask with a reflux

condenser in an Ar atmosphere. Dry 1,4-dioxane/tBuOH (3:1, 40 mL) was added. The mixture was stirred to reflux for 3 h. The reaction was cooled to room temperature, diluted with water, extracted with EtOAc, washed with brine and dried over sodium sulfate. The residue was purified using Flash chromatography giving the titled product as a yellow solid (676 mg, 75 %).

(*S*)-6-((3,5-difluoro-4-hydroxyphenyl)amino)-4-isopentyl-1,3-dimethyl-3,4-dihydropyrido[2,3-b]pyrazin-2(1H)-one (19)

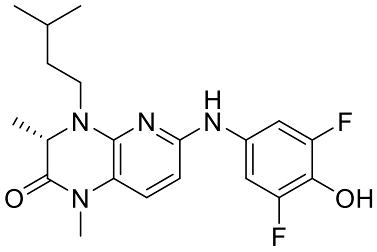

(*S*)-6-((4-(benzyloxy)-3,5-difluorophenyl)amino)-4-isopentyl-1,3-dimethyl-3,4-dihydropyrido[2,3-b]pyrazin-2(1H)-one (660 mg, 1.37 mmol) was dissolved in EtOH (30 mL). Pd/C (66 mg) was added. The mixture was stirred for 24 h in a hydrogen atmosphere. After full conversion, the reaction mixture was filtered over a Celite pad, evaporated, purified using Flash chromatography and recrystallized from toluene yielding a white solid (58 mg, 11 %). HPLC-DAD: 254 nm: 93.7 %, 230 nm: 93.9 %; ESI-HRMS: *m/z* [M+H]+: 391.19401 calcd.; 391.19450 found; 1H NMR (400 MHz, DMSO) δ 9.28 (s, 1H), 8.80 (s, 1H), 7.35 – 7.25 (m, 2H), 7.19 (d, J = 8.3 Hz, 1H), 6.12 (d, J = 8.4 Hz, 1H), 4.11 (q, J = 6.7 Hz, 1H), 3.97 – 3.87 (m, 1H), 3.19 (s, 3H), 3.10 – 2.99 (m, 1H), 1.71 – 1.60 (m, 1H), 1.55 – 1.47 (m, 2H), 1.12 (d, J = 6.7 Hz, 3H), 0.91 (t, J = 6.9 Hz, 6H). 13C NMR (101 MHz, DMSO) δ 165.00, 153.68, 152.45 (dd, J = 237.7, 9.2 Hz), 149.79, 144.46, 134.24 (t, J = 12.7 Hz), 125.90 (t, J = 16.7 Hz), 123.46, 115.02, 100.88

– 100.28 (m), 98.15, 56.65, 43.42, 36.27, 28.22, 25.72, 22.50, 22.40, 14.71. IR (ATR) [cm-1]: 2953, 2868, 1592, 1517, 1474, 1221, 1131, 1002.

Synthesis of 2-((3,5-difluoro-4-hydroxyphenyl)amino)-8-isopentyl-5-methyl-7,8-dihydropteridin-6(5H)-one (20)

Ethyl *N*-benzyl-*N*-isopentylglycinate

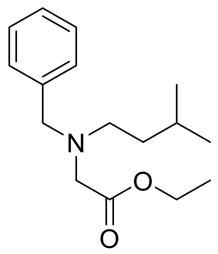

N-benzyl-3-methylbutan-1-amine (15.0 g, 0.085 mol), ethyl-2-bromoacetate (15.2 g, 0.091 mol), potassium carbonate (18.7 g, 0.135 mol), and DMF (125 mL) were added to a dry flask and stirred at 110 °C for 3 h. After the reaction was cooled, it was filtered and the filtrate was evaporated. The residue was extracted with water and diethyl ether and evaporated to dryness, yielding 21.5 g (0.082 mol, 96 %) of a brown oil. The crude product was used without further purification. ESI-MS: *m/z* 264.4 $[M+H]^+$ (264.2 calcd.)

Ethyl isopentylglycinate hydrochloride

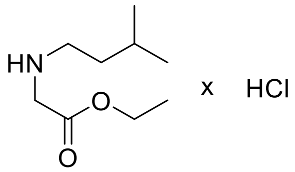

The crude mixture containing ethyl *N*-benzyl-*N*-isopentylglycinate (21.5 g, 0.081 mol) was dissolved in EtOH (232 mL) in a dry reactor. Palladium on charcoal (2.2 g, 10 %wt) and 37 % aq. hydrochloric acid (7.7 mL) was added. The reactor was purged with Argon and subsequently filled with H_2 up to 5 bar while stirring at room temperature until there was no loss in pressure anymore after 30 h. The mixture was poured over Celite and the residual ethanol was evaporated. The brown-yellow oil was poured over diethyl ether and was sonicated until a white precipitate formed. It was filtered and washed with diethyl ether yielding 14.8 g (0.071 mol, 86 %) of a white solid. HPLC-DAD: 254 nm: not detected, 230 nm: not detected; ESI-MS: *m/z* not detected $[M+H]^+$ (174.1 calcd.); ^{1}H NMR (400 MHz, DMSO-d_6) δ 9.54 (s, 1H), 4.20 (q, J = 7.1 Hz, 2H), 3.92 (s, 2H), 2.94 – 2.88 (m, 2H), 1.66 – 1.51 (m, 3H), 1.23 (t, J = 7.1 Hz, 3H), 0.87 (d, J = 6.3 Hz, 6H).

Ethyl *N*-(2-chloro-5-nitropyrimidin-4-yl)-N-isopentylglycinate

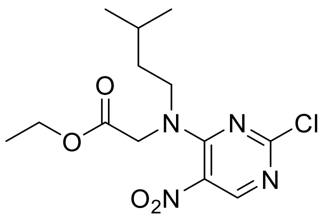

Ethyl isopentylglycinate hydrochloride (5.2 g, 24.9 mmol) was dissolved in 50 mL water and 2,4-dichloro-5-nitropyrimidine (5.0 g, 25.6 mmol) in 90 mL diethyl ether was added. The reaction mixture was cooled down to -10 °C and potassium carbonate (10.3 g, 74.6 mmol) was added portion-wise. The mixture was stirred vigorously for 1 h at -5 °C, slowly warming up to room temperature. After 3 h the water phase was separated, and the organic phase was washed with water three times, dried over sodium sulfate, and evaporated to dryness yielding 8.2 g (24.5 mmol, 84 %) of an orange oil. The product was used without further purification.

2-chloro-8-isopentyl-7,8-dihydropteridin-6(5H)-one

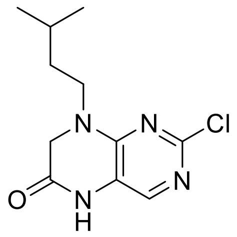

Ethyl *N*-(2-chloro-5-nitropyrimidin-4-yl)-N-isopentylglycinate (8.0 g, 24.5 mmol) was dissolved in 61 mL glacial acetic acid. The mixture was heated to 70 °C. The heat source was removed and Fe (6.1 g, 110 mmol) was added. The temperature raised to 110 °C while stirring vigorously. After 20 min, the temperature decreased. When 70 °C was reached, the mixture was filtered hot and the filtrate was evaporated. The residue was stirred in 60 mL water and 60 mL of DCM. The phases were separated and the aqueous phase was extracted with DCM. The combined organic layers were washed with water and brine. After drying over sodium sulfate, it was evaporated. The residue was sonicated in diethyl ether and a white-brown solid formed, that was filtered and evaporated to dryness yielding 2.8 g (11 mmol, 45 %). HPLC-DAD: 254 nm: 96.7 %, 230 nm: 95.2 %; ESI-MS: *m/z* 255.0 $[M+H]^+$ (255.1 calcd.); ^1H NMR (400 MHz, DMSO) δ 10.74 (s, 1H), 7.49 (s, 1H), 4.13 (s, 2H), 3.47 (t, J = 15.6, 8.1 Hz, 2H), 1.60 – 1.40 (m, 3H), 0.91 (d, J = 6.5 Hz, 6H).

2-chloro-8-isopentyl-5-methyl-7,8-dihydropteridin-6(5H)-one

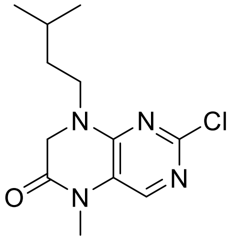

2-chloro-8-isopentyl-7,8-dihydropteridin-6(5H)-one (1.05 g, 4.1 mmol) and methyl iodide (2.3 g, 16.5 mmol) were dissolved in 11 mL DMA and cooled to –10 °C. Subsequently, 60 % sodium hydride on paraffin (0.18 g) was added while stirring for 2 h. HPLC confirmed a conversion into a new product and the reaction was quenched using ice and aq. ammonia (25 %). The reaction mixture was extracted three times with ethyl acetate. The combined organic layers were washed with brine, dried over sodium sulfate, loaded on Celite, evaporated, and purified using Flash chromatography yielding 0.71 g (2.65 mmol, 64 %) of a white-brown solid. ESI-MS: *m/z* 269.1 $[M+H]^+$ (269.1 calcd.); ^{1}H NMR (400 MHz, DMSO-d_6) δ 7.78 (s, 1H), 4.23 (s, 2H), 3.51 (d, J = 7.4 Hz, 2H), 3.20 (s, 3H), 1.61 – 1.49 (m, 1H), 1.49 – 1.42 (m, 2H), 0.92 (d, J = 6.5 Hz, 6H). ^{13}C NMR (101 MHz, DMSO-d_6) δ 161.8, 153.1, 151.9, 138.1, 121.3, 50.1, 44.9, 34.1, 28.0, 25.7, 22.8.

2-((3,5-difluoro-4-hydroxyphenyl)amino)-8-isopentyl-5-methyl-7,8-dihydropteridin-6(5H)-one (20)

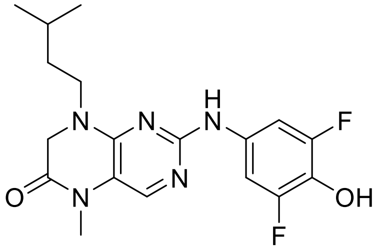

2-chloro-8-isopentyl-5-methyl-7,8-dihydropteridin-6(5H)-one (100 mg, 0.37 mmol), 4-amino-2,6-difluoro-phenol (76 mg, 0.52 mmol) and conc. HCl (0.1 mL) were dissolved in 1,4-dioxane and irradiated at 160 °C in a microwave reactor for 45 min. The solvent was evaporated, the residue was taken up in EtOAc and water. The phases were separated and the aqueous phase was extracted with EtOAc (2x). The combined organic layers were dried over sodium sulfate, evaporated and purified using Flash chromatography giving the titled product as a beige solid (101 mg, 72 %). HPLC-DAD: 254 nm: 100 %, 230 nm: 100 %; ESI-HRMS: *m/z* [M+H]+: 378.17361 calcd.; 378.17379 found; ^{1}H NMR (400 MHz, DMSO-d_6) δ 9.37 (s, 1H), 9.07 (s, 1H), 7.72 (s, 1H), 7.47 – 7.38 (m, 2H), 4.15 (s, 2H), 3.53 (t, J = 7.5 Hz, 2H), 3.20 (s, 3H), 1.69 – 1.57 (m, 1H), 1.55 – 1.47 (q, J = 15.2, 7.1 Hz, 2H), 0.92 (d, J = 6.5 Hz, 6H). ^{13}C NMR (101 MHz, DMSO) δ 160.8, 155.1, 152.2 (dd, J = 237.6, 9.0 Hz), 150.7, 138.0, 133.3 (t, J = 12.7 Hz), 126.5 (t, J = 16.7 Hz), 114.7, 101.32 (dd, J = 18.3, 9.4 Hz), 49.8, 44.7, 33.7, 27.3, 25.7, 22.4. IR (ATR) [cm-1]: 3272, 2957, 2920, 2870, 1647, 1522, 1435, 1236, 1011, 768.

Synthesis of 2-((3,5-difluoro-4-hydroxyphenyl)amino)-5-ethyl-8-isopentyl-7-methyl-7,8-dihydropteridin-6(5H)-one (21)

2-chloro-5-ethyl-8-isopentyl-7-methyl-7,8-dihydropteridin-6(5H)-one

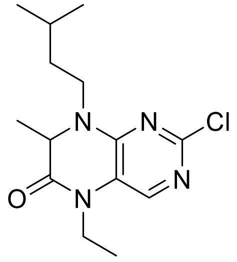

2-chloro-8-isopentyl-7-methyl-7,8-dihydropteridin-6(5H)-one (0.2 g, 0.74 mmol) and ethyl iodide (0.464 g, 2.98 mmol) were dissolved in 2 mL DMA and cooled to -10 °C. Subsequently, 60 % sodium hydride on paraffin (66 mg) was added while stirring for 2 h. HPLC confirmed a conversion into a new product and the reaction was quenched using ice and aq. ammonia (25 %). The reaction mixture was extracted three times with ethyl acetate. The combined organic layers were washed with brine, dried over sodium sulfate, and evaporated yielding 250 mg (0.84 mmol, 113 %) of a yellow solid. The crude mixture was used without further purification. ESI-MS: *m/z* not detected $[M+H]^+$ (297.1 calcd.);

2-((3,5-difluoro-4-hydroxyphenyl)amino)-5-ethyl-8-isopentyl-7-methyl-7,8-dihydropteridin-6(5H)-one (21)

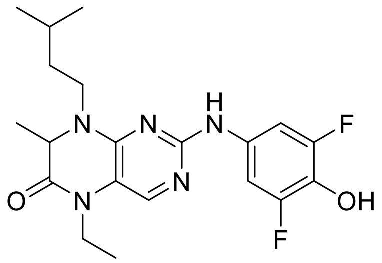

2-chloro-5-ethyl-8-isopentyl-7-methyl-7,8-dihydropteridin-6(5H)-one (262 mg, 0.88 mmol) was dissolved in sulfolane. 4-amino-2,6-difluorophenol (128 mg, 0.88 mmol) was added and stirred for 0.5 h at 170 °C. The mixture was mixed with diethyl ether, the residue was purified with Flash chromatography (DCM and 10 % MeOH in DCM), yielding 88 mg (25 %) of an orange solid. HPLC-DAD: 254 nm: 97.8 %, 230 nm: 94.8 %; ESI-HRMS: *m/z* [M+H]+: 406.20491 calcd.; 406.20526 found; ^{1}H NMR (400 MHz, DMSO-d_6) δ 9.41 (s, 1H), 9.14 (s, 1H), 7.86 (s, 1H), 7.49 – 7.37 (m, 2H), 4.22 (q, J = 6.7 Hz, 1H), 4.06 – 3.73 (m, 3H), 3.18 – 3.08 (m, 1H), 1.66 – 1.45 (m, 3H), 1.25 (d, J = 6.7 Hz, 3H), 1.10 (t, J = 7.0 Hz, 3H), 0.94 – 0.89 (m, 6H).

^{13}C NMR (101 MHz, DMSO-d6) δ 163.4, 155.2, 152.22 (dd, J = 237.7, 9.0 Hz), 150.9, 138.4, 133.2 (t, J = 12.7 Hz), 126.63 (t, J = 16.6 Hz), 113.0, 101.5 (dd, J = 17.8, 9.3 Hz), 56.1, 43.0, 35.6, 35.4, 25.8, 22.5, 22.4, 16.7, 12.0. IR (ATR) [cm-1]: 2955, 2871, 1653, 1576, 1519, 1429, 1367, 1319, 1224, 1142.

Synthesis of 2,6-difluoro-4-((8-isopentyl-5,7-dimethyl-5,6,7,8-tetrahydropteridin-2-yl)amino)phenol (22)

2-chloro-8-isopentyl-5,7-dimethyl-5,6,7,8-tetrahydropteridine

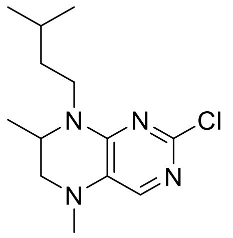

2-chloro-8-isopentyl-5,7-dimethyl-7,8-dihydropteridin-6(5H)-one (500 mg, 1.77 mmol) and anhydrous DCM (5 mL) were charged into a dry flask and stirred under nitrogen atmosphere for 15 min and cooled to 0 – 5 ˚C. Trimethylsilyl chloride (250 mg, 2.3 mmol) was added to the mixture. Lithium aluminum hydride (94 mg, 2.5 mmol) in anhydrous THF (5 mL) solution was added dropwise. After addition, the solution was allowed to warm to rt and stirred for 2 h. After completion, the reaction was quenched by addition of 2 M sodium hydroxide. The layers were separated and the aqueous phase was extracted with DCM (2x). The organic layers were combined and washed with brine solution, dried over sodium sulfate, filtered and evaporated giving a white solid (414 mg, 87 %). ESI-MS: *m/z* 269.1 $[M+H]^+$ (269.1 calcd.); 1H NMR (400

MHz, DMSO) δ 7.26 (s, 1H), 3.88 – 3.79 (m, 1H), 3.77 – 3.69 (m, 1H), 3.19 – 3.09 (m, 1H), 2.97 (d, J = 2.8 Hz, 2H), 2.76 (s, 3H), 1.57 – 1.38 (m, 3H), 1.22 (d, J = 6.4 Hz, 3H), 0.90 (d, J = 6.5 Hz, 6H).

N-(4-(benzyloxy)-3,5-difluorophenyl)-8-isopentyl-5,7-dimethyl-5,6,7,8-tetrahydropteridin-2-amine

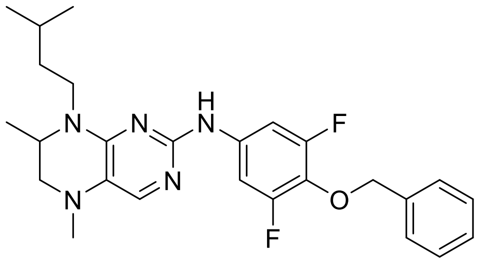

2-chloro-8-isopentyl-5,7-dimethyl-5,6,7,8-tetrahydropteridine (413 mg, 1.54 mmol), 4-(benzyloxy)-3,5-difluoroaniline (724.6 mg, 3.1 mmol), K_2CO_3 (426 mg, 3.1 mmol) and Xantphos Pd G3 (29 mg, 0.03 mmol) was added to a dry flask with a reflux condenser in an argon atmosphere. An anhydrous 1,4-dioxane/tBuOH mixture (3:1, 30 mL) was added. The mixture was refluxed for 3 h. The reaction was cooled to room temperature, diluted with water, extracted with EtOAc, washed with brine and dried over sodium sulfate. The residue was purified using Flash chromatography yielding a white solid (265 mg, 44 %). 1H NMR (400 MHz, DMSO) δ 8.88 (s, 1H), 7.52 – 7.26 (m, 9H), 5.02 (s, 2H), 3.95 – 3.85 (m, 1H), 3.74 – 3.67 (m, 1H), 3.23 – 3.14 (m, 1H), 2.93 – 2.81 (m, 2H), 2.71 (s, 3H), 1.66 – 1.56 (m, 1H), 1.53 – 1.42 (m, 2H), 1.25 (d, J = 6.3 Hz, 3H), 0.95 – 0.89 (m, 6H).

2,6-difluoro-4-((8-isopentyl-5,7-dimethyl-5,6,7,8-tetrahydropteridin-2-yl)amino)phenol (22)

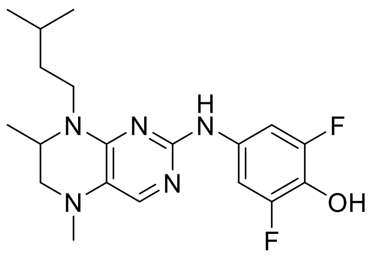

N-(4-(benzyloxy)-3,5-difluorophenyl)-8-isopentyl-5,7-dimethyl-5,6,7,8-tetrahydropteridin-2-amine (260 mg, 0.56 mmol) was dissolved in EtOH (10 mL) and HCl conc. (0.25 mL). Pd/C (26 mg) was added. The mixture was stirred for 24 h in a hydrogen atmosphere. After full conversion, the reaction mixture was filtered over Celite, evaporated and purified using Flash chromatography yielding a grey solid (79 mg, 37 %). HPLC-DAD: 254 nm: 96.5 %, 230 nm: 95.6 %; ESI-HRMS: *m/z* [M+H]+: 378.20999 calcd.; 378.21032 found; 1H NMR (400 MHz, DMSO) δ 8.79 (s, 1H), 8.14 (s, 1H), 7.42 – 7.33 (m, 2H), 7.23 (s, 1H), 3.95 – 3.85 (m, 1H), 3.75 – 3.68 (m, 1H), 3.24 – 3.14 (m, 1H), 2.93 – 2.81 (m, 2H), 2.70 (s, 3H), 1.67 – 1.56 (m, 1H), 1.52 – 1.44 (m, 2H), 1.25 (d, J = 6.3 Hz, 3H), 0.94 – 0.89 (m, 6H). 13C NMR (101 MHz, DMSO) δ 163.60, 152.79, 152.32 (dd, J = 237.4, 9.0 Hz), 151.63, 133.99 – 133.55 (m), 132.35, 126.21 – 125.84 (m), 123.69, 101.21 – 100.70 (m), 53.89, 51.27, 44.68, 38.87, 35.97, 26.30, 22.99, 19.16. IR (ATR) [cm-1]: 2956, 2868, 2786, 1636, 1515, 1418, 1234, 1017, 844, 757.

Synthesis of 8-cyclohexyl-2-((3,5-difluoro-4-hydroxyphenyl)amino)-5-methyl-7,8-dihydropteridin-6(5H)-one (23)

Ethyl cyclohexylglycinate

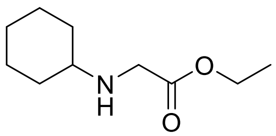

A mixture of cyclohexylamine (275 mL, 2.40 mol) and toluene (400 mL) was cooled in an ice bath. Over 2 h, ethylbromoacetate (66 mL, 599 mmol) was added. The reaction mixture was filtered and the filtrate was concentrated under reduced pressure. The organic layer was washed with 10 % ammonia solution and dried over sodium sulfate. Residual cyclohexylamine was removed by co-evaporation with toluene. This gave the titled product as a colorless oil (92.7 g, 84 %). ESI-MS: *m/z* 186.2 $[M+H]^+$ (186.1 calcd.); 1H NMR (400 MHz, DMSO) δ 3.61 (s, 3H), 3.33 (s, 2H), 2.39 – 2.30 (m, 1H), 1.80 – 1.47 (m), 1.25 – 0.90 (m).

Ethyl *N*-(2-chloro-5-nitropyrimidin-4-yl)-N-cyclohexylglycinate

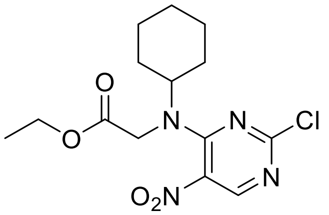

Ethyl cyclohexylglycinate (1.95 g, 11.4 mmol) was dissolved in water (20 mL) and 2,4-dichloro-5-nitropyrimidine (2.87 g, 14.8 mmol) in diethyl ether (33 mL) was added. The reaction mixture was cooled down to 0 °C and K_2CO_3 (7.23 g, 34.1 mmol) was added portion wise. The mixture was stirred vigorously for 1 h, slowly warming up to room temperature. After 16 h the water phase was separated, and the organic phase was washed with water three times, dried over sodium sulfate and evaporated to dryness yielding a yellow oil (3.56 g, 95%). ESI-MS: *m/z* 351.2 $[M+H]^+$ (351.1 calcd.); 1H NMR (400 MHz, DMSO) δ 8.81 (s, 1H), 4.33 (s, 2H), 3.66 (s, 3H), 1.84 – 1.71 (m), 1.64 – 1.50 (m), 1.25 – 1.03 (m).

2-chloro-8-cyclohexyl-7,8-dihydropteridin-6(5H)-one

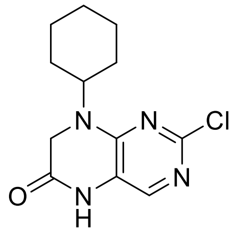

Ethyl *N*-(2-chloro-5-nitropyrimidin-4-yl)-N-cyclohexylglycinate (3.56 g, 10.8 mmol) was dissolved in glacial acetic acid (27 mL) and heated to 100 °C. Powdered iron (2.68 g, 48.7 mmol) was added portion wise and the reaction was stirred for 1 h. Upon completion, the mixture was filtered and the filtrate was concentrated in vacuo. The residue was taken up in water and EtOAc. The phases were separated and the aqueous phase was extracted with EtOAc (2x). The combined organic layers were washed with water and brine, dried over sodium sulfate and evaporated to dryness. The product was precipitated from ether yielding a brown solid (1.74 g, 60 %). ESI-MS: *m/z* 265.0 $[M-H]^-$ (265.0 calcd.); 1H NMR (400 MHz, DMSO) δ 10.75 (s, 1H), 7.51 (s, 1H), 4.06 (s, 2H), 1.84 – 1.74 (m, 2H), 1.67 – 1.47 (m, 5H), 1.40 – 1.27 (m, 2H), 1.18 – 1.05 (m, 1H).

2-chloro-8-cyclohexyl-5-methyl-7,8-dihydropteridin-6(5H)-one

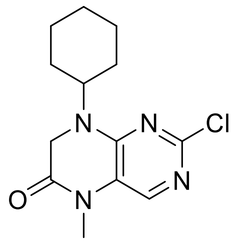

2-chloro-8-cyclohexyl-7,8-dihydropteridin-6(5H)-one (1.7 g, 6.4 mmol) and K_2CO_3 (1.75 g, 12.8 mmol) were added to DMF (18 mL) and cooled to 0 °C. Subsequently, methyl iodide (1.81 g, 12.7 mmol) was added while stirring for 16 h. Upon completion, water was added and a precipitate formed that was suction filtered, washed with water and dried giving a brown solid (1.24 g, 69 %). ESI-MS: *m/z* 281.1 $[M+H]^+$ (281.1 calcd.); 1H NMR (400 MHz, DMSO) δ 7.79 (s, 1H), 4.44 – 4.33 (m, 1H), 4.16 (s, 2H), 3.20 (s, 3H), 1.83 – 1.74 (m, J = 13.0 Hz, 2H), 1.68 – 1.48 (m, 5H), 1.40 – 1.27 (m, 2H), 1.18 – 1.05 (m, 1H).

8-cyclohexyl-2-((3,5-difluoro-4-hydroxyphenyl)amino)-5-methyl-7,8-dihydropteridin-6(5H)-one (23)

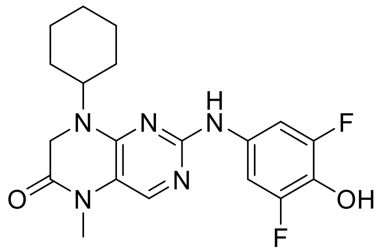

2-chloro-8-cyclohexyl-5-methyl-7,8-dihydropteridin-6(5H)-one (200 mg, 0.71 mmol), 4-amino-2,6-difluorophenol (193 mg, 0.82 mmol), Cs_2CO_3 (464 mg, 1.42 mmol) and Pd G3 Brettphos (13 mg, 0.014 mmol) was added to a dry flask with a reflux condenser in an argon atmosphere. Anhydrous 1,4-dioxane was added. The mixture was stirred to reflux for 3 h. The reaction was cooled to room temperature, diluted with water, extracted with EtOAc, washed with

brine and dried over sodium sulfate. The residue was purified using Flash chromatography giving the titled product as a brown solid (44 mg, 16 %). HPLC-DAD: 254 nm: 100 %, 230 nm: 100 %; ESI-HRMS: *m/z* [M+H]+: 390.17361 calcd.; 390.17433 found; 1H NMR (400 MHz, DMSO) δ 9.39 (s, 1H), 9.12 (s, 1H), 7.74 (s, 1H), 7.48 – 7.38 (m, 2H), 4.53 – 4.43 (m, 1H), 4.06 (s, 2H), 3.18 (s, 3H), 1.89 – 1.78 (m, J = 12.5 Hz, 2H), 1.74 – 1.49 (m, 5H), 1.45 – 1.31 (m, 2H), 1.25 – 1.08 (m, 1H). 13C NMR (101 MHz, DMSO) δ 161.13, 155.05, 152.22 (dd, J = 237.5, 8.9 Hz), 150.48, 138.32, 133.28 (t, J = 12.8 Hz), 126.53 (t, J = 16.7 Hz), 114.87, 101.65 – 101.10 (m), 52.79, 44.90, 27.85, 27.27, 25.42, 25.00. IR (ATR) [cm-1]: 3285, 2931, 2855, 1668, 1606, 1580, 1518, 1431, 1422, 1220.

Synthesis of 2-((3,5-difluoro-4-hydroxyphenyl)amino)-5-methyl-8-phenyl-7,8-dihydropteridin-6(5H)-one (24)

Methyl phenylglycinate hydrochloride

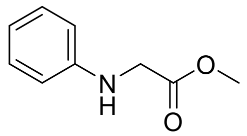

To a stirred solution of *N*-phenylglycine (2.41 g, 16.0 mmol) and trimethyl orthoformate (1.70 g, 16.0 mmol) in MeOH (44 mL) was added hydrogen chloride (22 mL, 4.0 M solution in dioxane) and the mixture was stirred overnight at rt. The organic solvent was removed under reduced pressure and taken up in diethyl ether and the hydrochloride precipitated, was filtered and washed with diethyl ether giving a white solid (3.06 g, 95 %). ESI-MS: *m/z* 389.9 $[M+H]^+$

(390.1 calcd.); 1H NMR (400 MHz, DMSO) δ 7.16 – 7.09 (m, 2H), 6.71 – 6.65 (m, 3H), 3.95 (s, 2H), 3.65 (s, 3H).

Methyl *N*-(2-chloro-5-nitropyrimidin-4-yl)-N-phenylglycinate

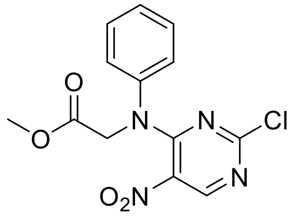

Methyl phenylglycinate hydrochloride (3.0 g, 14.9 mmol) and K_2CO_3 (9.46 g, 44.6 mmol) were suspended in acetone (38 mL) and at 0 °C combined with 2,4-dichloro-5-nitropyrimidine (1.73 g, 8.9 mmol). The mixture was stirred for 4 h at 40 °C. Water was added and the phases were separated and the aqueous phase was extracted with EtOAc (2x). The combined organic layers were dried over sodium sulfate, filtered, evaporated and purified using Flash chromatography giving the titled product as a yellow oil (3.77 g, 79 %). ESI-MS: *m/z* 345.0 $[M+Na]^+$ (345.0 calcd.); 1H NMR (400 MHz, DMSO) δ 8.85 (s, 1H), 7.41 – 7.35 (m, 2H), 7.32 – 7.28 (m, 3H), 4.82 (s, 2H), 3.67 (s, 3H).

2-chloro-8-cyclohexyl-7,8-dihydropteridin-6(5H)-one

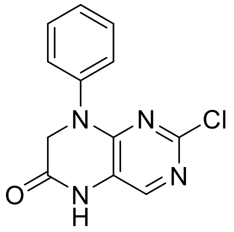

Methyl *N*-(2-chloro-5-nitropyrimidin-4-yl)-N-phenylglycinate (4.51 g, 8.3 mmol) was dissolved in glacial acetic acid (33 mL) and heated to 100 °C. Powdered iron (3.46 g, 62.9

mmol) was added portion wise and the reaction was stirred for 1 h. Upon completion, the mixture was filtered and the filtrate was concentrated in vacuo. The residue was taken up in water and EtOAc. The phases were separated and the aqueous phase was extracted with EtOAc (2x). The combined organic layers were washed with water and brine, dried over sodium sulfate and evaporated to dryness. The product was precipitated from ether yielding a brown solid (1.21 g, 33 %). 1H NMR (400 MHz, DMSO) δ 7.76 (s, 1H), 7.50 – 7.40 (m, 4H), 7.34 – 7.29 (m, 1H), 4.45 (s, 2H).

2-chloro-8-cyclohexyl-5-methyl-7,8-dihydropteridin-6(5H)-one

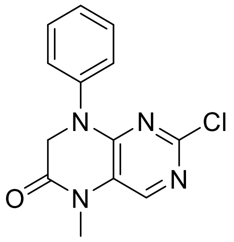

2-chloro-8-cyclohexyl-7,8-dihydropteridin-6(5H)-one (1.2 g, 4.6 mmol) and K_2CO_3 (1.27 g, 9.2 mmol) were added to DMF (12 mL) and cooled to 0 °C. Subsequently, methyl iodide (1.31 g, 9.2 mmol) was added while stirring for 2 h. Upon completion, the reaction was quenched with ice and aq. ammonia (25%). The precipitate was filtered and washed with water. The residue was dried yielding a brown solid (897 mg, 71 %) that was used without further purification.

2-((3,5-difluoro-4-hydroxyphenyl)amino)-5-methyl-8-phenyl-7,8-dihydropteridin-6(5H)-one (24)

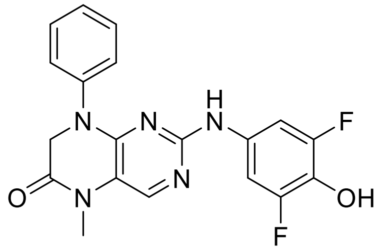

2-chloro-8-cyclohexyl-5-methyl-7,8-dihydropteridin-6(5H)-one (200 mg, 0.75 mmol), 4-amino-2,6-difluorophenol (202 mg, 0.86 mmol), Cs_2CO_3 (486 mg, 1.49 mmol) and Pd G3 Brettphos (14 mg, 0.015 mmol) was added to a dry flask with a reflux condenser in an argon atmosphere. Anhydrous 1,4-dioxane (11 mL) was added. The mixture was stirred to reflux for 3 h. The reaction was cooled to room temperature, diluted with water, extracted with EtOAc, washed with brine and dried over sodium sulfate. The residue was purified using Flash chromatography giving the titled product as a beige solid (107 mg, 37 %). HPLC-DAD: 254 nm: 99.8 %, 230 nm: 98.0 %; ESI-HRMS: [M+H]+: 390.17361 calcd.; 390.17433 found; ESI-MS: *m/z* 381.8 $[M-H]^-$ (382.1 calcd.); 1H NMR (400 MHz, DMSO) δ 9.27 (s, 1H), 9.12 (s, 1H), 7.97 (s, 1H), 7.50 – 7.42 (m, 4H), 7.34 – 7.28 (m, 1H), 7.20 – 7.10 (m, 2H), 4.51 (s, 2H), 3.30 (s, 3H). 13C NMR (101 MHz, DMSO) δ 161.31, 154.73, 152.04 (dd, J = 237.7, 8.8 Hz), 151.05, 141.31, 140.08, 132.87 (t, J = 12.9 Hz), 129.14, 126.46 (t, J = 16.4 Hz), 126.33, 125.48, 115.23, 101.52 – 100.88 (m), 52.31, 27.59.

Synthesis of 7-((3,5-difluoro-4-hydroxyphenyl)amino)-1,4-dimethyl-1,4-dihydropyrido[3,4-b]pyrazin-3(2H)-one (25)

Ethyl *N*-(2-chloro-5-nitropyridin-4-yl)-N-methylglycinate

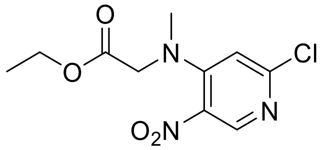

2,4-dichloro-5-nitropyridine (3.4 g, 17.6 mmol) and ethyl 2-(methylamino)acetate hydrochloride (3.0 g, 19.5 mmol) were dissolved in MeCN (50 mL) while stirring. TEA (6.9 g, 68 mmol) was slowly added. The reaction was stirred for 3 h at RT. The solvent was evaporated, the remains were taken up in EtOAc, washed with water and brine, dried over sodium sulfate and evaporated giving a crude brown oil (4.57 g, 95 %). ESI-MS: *m/z* 295.9 $[M+Na]^+$ (296.1 calcd.); 1H NMR (400 MHz, CDCl3) δ 8.59 (s, 1H), 6.74 (s, 1H), 4.27 (q, J = 7.1 Hz, 2H), 3.97 (s, 2H), 3.01 (s, 3H), 1.31 (t, J = 7.1 Hz, 3H). 13C NMR (101 MHz, CDCl3) δ 168.2, 154.9, 150.5, 148.1, 135.2, 111.0, 62.2, 55.0, 41.3, 14.3.

7-chloro-1-methyl-1,4-dihydropyrido[3,4-b]pyrazin-3(2H)-one

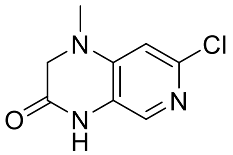

Ethyl *N*-(2-chloro-5-nitropyridin-4-yl)-N-methylglycinate (4.5 g, 16.4 mmol) and ammonium acetate (3.8 g, 49.3 mmol) were dissolved in 1,4-dioxane/H_2O (4:1, 70 mL). The mixture was heated to 80 °C. The heat source was removed and Zn (7.53 g, 115 mmol) was added. The temperature raised to 110 °C while stirring vigorously. After 20 min, the temperature decreased.

After another 20 min, the reaction mixture was poured in water, extracted 3x with EtOAc, dried over sodium sulfate, evaporated and purified using Flash chromatography yielding a white solid (635 mg, 20 %). ESI-MS: *m/z* 195.8 $[M-H]^-$ (196.0 calcd.); 1H NMR (400 MHz, DMSO) δ 10.66 (s, 1H), 7.55 (s, 1H), 6.64 (s, 1H), 3.91 (s, 2H), 2.85 (s, 3H). 13C NMR (101 MHz, DMSO) δ 163.81, 144.87, 143.09, 132.70, 123.04, 103.97, 52.38, 35.93.

7-chloro-1,4-dimethyl-1,4-dihydropyrido[3,4-b]pyrazin-3(2H)-one

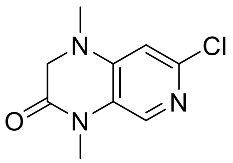

7-chloro-1-methyl-1,4-dihydropyrido[3,4-b]pyrazin-3(2H)-one (0.635 g, 3.2 mmol) and K_2CO_3 (0.58 g, 5.1 mmol) were added to DMF (7 mL) at rt. Subsequently, methyl iodide (0.22 mL, 3.5 mmol) was added while stirring. After 16 h, HPLC confirmed a conversion into a new product and water was added to the reaction. A precipitate formed that was suction filtered and dried, yielding a brown solid (0.6 g, 88 %). 1H NMR (400 MHz, DMSO) δ 7.80 (s, 1H), 6.68 (s, 1H), 4.00 (s, 2H), 3.26 (s, 3H), 2.86 (s, 3H). 13C NMR (101 MHz, DMSO) δ 163.0, 145.5, 144.6, 133.0, 125.0, 103.9, 52.5, 36.1, 27.8.

7-((4-(benzyloxy)-3,5-difluorophenyl)amino)-1,4-dimethyl-1,4-dihydropyrido[3,4-b]pyrazin-3(2H)-one

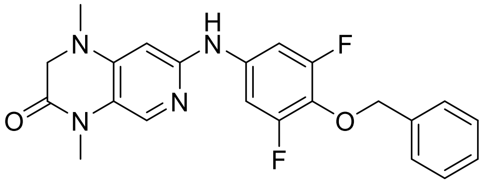

7-chloro-1,4-dimethyl-1,4-dihydropyrido[3,4-b]pyrazin-3(2H)-one (253 mg, 1.2 mmol), 4-(benzyloxy)-3,5-difluoroaniline (337 mg, 1.43 mmol), Brettphos Pd G3 (21.7 mg, 0.024 mmol) and Cs_2CO_3 (779 mg, 2.39 mmol) were added to a dry flask with a stirrer in an argon atmosphere. 1,4-dioxane(14 mL) was added. The mixture was refluxed for 4 h in an argon atmosphere. After full conversion, the reaction mixture was filtered over celite, evaporated and purified using Flash chromatography yielding a yellow-orange material (314 mg, 64 %). ESI-MS: *m/z* 411.1 $[M+H]^+$ (411.2 calcd.).

7-((3,5-difluoro-4-hydroxyphenyl)amino)-1,4-dimethyl-1,4-dihydropyrido[3,4-b]pyrazin-3(2H)-one (25)

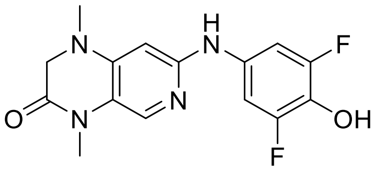

7-((4-(benzyloxy)-3,5-difluorophenyl)amino)-1,4-dimethyl-1,4-dihydropyrido[3,4-b]pyrazin-3(2H)-one (314 mg, 0.77 mmol) and Pd/C (31 mg) were dissolved in EtOAc (18 mL). The mixture was purged with hydrogen gas for 16 h. Upon completion, the mixture was filtered over celite, evaporated and purified using Flash chromatography with a gradient of 100 % PE to 100 % EtOAc giving the desired compound as a transparent solid (15 mg, 6 %). HPLC-DAD: 254 nm: 99.2 %, 230 nm: 96.1 %; ESI-HRMS: *m/z* [M+H]+: 321.11576 calcd.; 321.11632 found; 1H NMR (400 MHz, DMSO) δ 9.29 (s, 1H), 8.81 (s, 1H), 7.71 (s, 1H), 7.43 – 7.32 (m, 2H), 6.00 (s, 1H), 3.88 (s, 2H), 3.25 (s, 3H), 2.80 (s, 3H). 13C NMR (101 MHz, DMSO) δ 162.99, 152.67, 152.37 (dd, J = 237.7, 9.4 Hz), 144.38, 134.43 (t, J = 12.4 Hz), 131.12, 125.90 (t, J = 16.5 Hz), 120.17, 100.94 – 100.51 (m), 91.17, 53.08, 35.98, 27.73. IR (ATR) [cm-1]: 3351, 2923, 2851, 1643, 1504, 1231, 1008, 842, 820, 799.

Synthesis of 7-((3,5-difluoro-4-hydroxyphenyl)amino)-4-methyl-1-propyl-1,4-dihydropyrido[3,4-b]pyrazin-3(2H)-one (26)

Ethyl propylglycinate

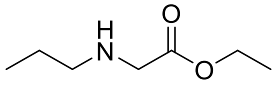

A mixture of propan-1-amine (12.7 g, 216 mmol), TEA (8.7 g, 86.2 mmol) and DCM (300 mL) was cooled in an ice bath. Over 1 h, ethylbromoacetate (12.0 g, 71.9 mmol) was added and stirred overnight. The reaction mixture was filtered and the filtrate was washed with aqueous ammonia solution, dried over sodium sulfate and evaporated. Residual n-propylamine was removed by evaporation under reduced pressure giving the titled product as a colorless oil (7.04 g, 68 %) that was used without further purification.

Ethyl *N*-(2-chloro-5-nitropyridin-4-yl)-*N*-propylglycinate

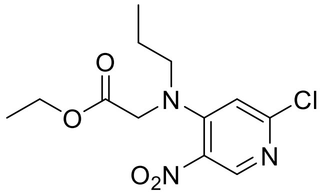

2,4-dichloro-5-nitropyridine (3.18 g, 16.5 mmol) and ethyl propylglycinate (2.66 g, 18.3 mmol) were dissolved in acetonitrile while stirring. TEA was slowly added. The reaction was stirred overnight at RT. The solvent was evaporated, the remains were taken up with EtOAc, washed with water and brine, dried over sodium sulfate, evaporated and purified using Flash chromatography giving the titled product as a yellow oil (1.46 g, 29 %). 1H NMR (400 MHz, DMSO) δ 8.56 (s, 1H), 7.16 (s, 1H), 4.19 (s, 2H), 4.13 (q, J = 7.1 Hz, 2H), 3.30 – 3.25 (m, 2H), 1.62 – 1.49 (m, 2H), 1.20 (t, J = 7.1 Hz, 3H), 0.82 (t, J = 7.3 Hz, 3H).

7-chloro-1-propyl-1,4-dihydropyrido[3,4-b]pyrazin-3(2H)-one

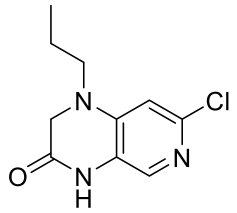

Ethyl N-(2-chloro-5-nitropyridin-4-yl)-N-propylglycinate (1.46 g, 4.8 mmol) was dissolved in glacial acetic acid (20 mL). The mixture was heated to 70 °C and powdered iron (1.35 g, 24 mmol) was added portion wise. The temperature raised to 110 °C and was kept stirring for 1 h. The solids were filtered, the filtrate was extracted 3x with EtOAc, The combined organic layers were washed with brine. After drying over sodium sulfate, it was evaporated and purified using Flash chromatography yielding a brown solid (801 mg, 74 %). ESI-MS: *m/z* 224.0 $[M-H]^-$ (224.1 calcd.); 1H NMR (400 MHz, DMSO) δ 10.67 (s, 1H), 7.53 (s, 1H), 6.66 (s, 1H), 3.95 (s, 2H), 3.26 – 3.19 (m, 2H), 1.61 – 1.48 (m, 2H), 0.89 (t, J = 7.4 Hz, 3H).

7-chloro-4-methyl-1-propyl-1,4-dihydropyrido[3,4-b]pyrazin-3(2H)-one

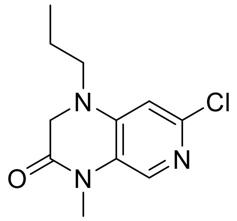

7-chloro-1-propyl-1,4-dihydropyrido[3,4-b]pyrazin-3(2H)-one (800 mg, 3.5 mmol) and K_2CO_3 (636 mg, 5.7 mmol) were added to DMF (8 mL) at rt. Subsequently, methyl iodide (755 mg, 5.3 mmol) was added while stirring. After 16 h, HPLC confirmed a conversion into a new product and water was added to the reaction. A precipitate formed that was suction filtered and dried, yielding a brown solid (632 mg) that was used in the next step without further purification.

7-((4-(benzyloxy)-3,5-difluorophenyl)amino)-4-methyl-1-propyl-1,4-dihydropyrido[3,4-b]pyrazin-3(2H)-one

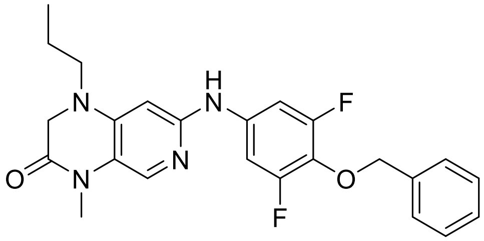

7-chloro-4-methyl-1-propyl-1,4-dihydropyrido[3,4-b]pyrazin-3(2H)-on (200 mg, 0.83 mmol), 4-(benzyloxy)-3,5-difluoroaniline (393 mg, 1.67 mmol), Brettphos Pd G3 (15 mg, 0.017 mmol) and KOtBu (187 mg, 1.67 mmol) were added to a dry flask with a stirrer in an argon atmosphere. Anhydrous 1,4-dioxane (11 mL) was added. The mixture was heated to 80 °C for 4 h in an argon atmosphere. After full conversion, the reaction mixture was filtered over celite, evaporated and purified using Flash chromatography yielding a brown solid (120 mg, 33 %) that was used in the next step without further purification.

7-((3,5-difluoro-4-hydroxyphenyl)amino)-4-methyl-1-propyl-1,4-dihydropyrido[3,4-b]pyrazin-3(2H)-one (26)

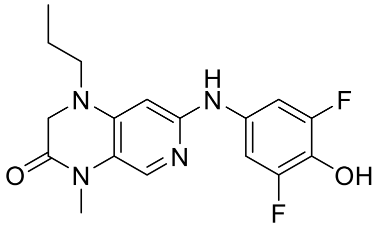

7-((4-(benzyloxy)-3,5-difluorophenyl)amino)-4-methyl-1-propyl-1,4-dihydropyrido[3,4-b]pyrazin-3(2H)-one (120 mg, 0.27 mmol) was dissolved in EtOH (5 mL) and conc. HCl (0.2 mL). Pd/C (12 mg) was added. The mixture was stirred for 24 h in a hydrogen atmosphere. After full conversion, the reaction mixture was filtered over Celite, evaporated and purified using

Flash chromatography yielding a beige solid (48 mg, 50 %). HPLC-DAD: 254 nm: 97.6 %, 230 nm: 97.0 %; ESI-HRMS: *m/z* [M+H]+: 349.14706 calcd.; 349.14735 found; 1H NMR (700 MHz, DMSO) δ 9.27 (s, 1H), 8.75 (s, 1H), 7.69 (s, 1H), 7.41 – 7.33 (m, 2H), 6.02 (s, 2H), 3.24 (s, 3H), 3.16 – 3.14 (m, 2H), 1.63 – 1.56 (m, 2H), 0.92 (t, J = 7.4 Hz, 3H). 13C NMR (176 MHz, DMSO) δ 175.5, 162.7, 152.7, 152.41 (dd, J = 237.7, 9.0 Hz), 143.0, 134.52 (t, J = 12.8 Hz), 131.4, 125.84 (t, J = 16.7 Hz), 119.9, 100.62 (dd, J = 20.5, 6.3 Hz), 90.7, 51.1, 50.1, 27.7, 17.4, 11.2. IR (ATR) [cm-1]: 2920, 1653, 1646, 1605, 1505, 1213, 1010, 801.

Synthesis of 7-((3,5-difluoro-4-hydroxyphenyl)amino)-1-isopropyl-4-methyl-1,4-dihydropyrido[3,4-b]pyrazin-3(2H)-one (27)

Ethyl isopropylglycinate hydrochloride

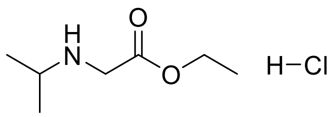

A mixture of isopropylamine (17 g, 287 mmol) and toluene (40 mL) was cooled in an ice bath. Over 2 h, ethylbromoacetate (12 g, 72 mmol) was added. The reaction mixture was filtered and the filtrate was concentrated under reduced pressure. The organic layer was washed with 10 % ammonia solution and dried over sodium sulfate. Residual isopropylamine was evaporated under reduced pressure. The residue was taken up in a 4 M HCl in 1,4-dioxane solution (35 mL) and stirred for 1 h. Diethyl ether was added and a white precipitate formed that was suction filtered and dried giving the titled product as a white crystals (11.9 g, 91 %). ESI-MS: *m/z* 146.3 $[M+H]^+$ (146.1 calcd.); 1H NMR (400 MHz, CDCl3) δ 9.75 (s, 2H), 4.25 (q, J = 6.9 Hz, 2H), 3.81 (s, 2H), 3.61 (bs, 1H), 1.48 (d, J = 5.8 Hz, 6H), 1.29 (t, J = 6.9 Hz, 3H). 13C NMR (101 MHz,

CDCl3) δ 166.05 (d, J = 5.2 Hz), 62.54 (d, J = 7.7 Hz), 50.89 (d, J = 9.9 Hz), 44.36 (d, J = 14.2 Hz), 19.06 (s), 14.07 (d, J = 13.8 Hz).

Ethyl *N*-(2-chloro-5-nitropyridin-4-yl)-*N*-isopropylglycinate

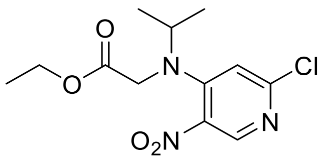

2,4-dichloro-5-nitropyridine (2.87 g, 14.9 mmol) and ethyl isopropylglycinate hydrochloride (3.0 g, 16.5 mmol) were dissolved in MeCN (30 mL) while stirring. TEA (6.9 mL, 49.5 mmol) was slowly added. The reaction was stirred overnight at rt. The solvent was evaporated, the remains were taken up with EtOAc, washed with water and brine, dried over sodium sulfate, evaporated and purified using Flash chromatography giving the titled product as a yellow oil (3.2 g, 64 %). ESI-MS: *m/z* 324.4 $[M+H]^+$ (324.1 calcd.); 1H NMR (400 MHz, DMSO) δ 8.54 (s, 1H), 7.19 (s, 1H), 4.24 (s, 2H), 4.08 (q, J = 7.1 Hz, 2H), 3.83 – 3.75 (m, 1H), 1.17 – 1.13 (m, 9H). 13C NMR (101 MHz, DMSO) δ 169.0, 153.4, 149.6, 147.6, 136.0, 112.2, 61.2, 53.7, 45.5, 19.3, 14.0.

7-chloro-1-isopropyl-1,4-dihydropyrido[3,4-b]pyrazin-3(2H)-one

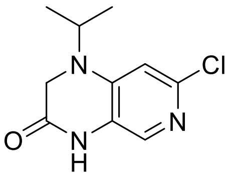

Ethyl *N*-(2-chloro-5-nitropyridin-4-yl)-*N*-isopropylglycinate (3.2 g, 10.6 mmol) was dissolved in glacial acetic acid (45 mL). The mixture was heated to 70 °C and powdered iron (3.0 g, 53 mmol) was added portion wise. The temperature raised to 110 °C and was kept stirring for 1 h.

The solids were filtered, the filtrate was extracted 3x with EtOAc, The combined organic layers were washed with brine. After drying over sodium sulfate, it was evaporated and purified using Flash chromatography yielding a brown solid (2.2 g, 92 %). ESI-MS: *m/z* 224.4 $[M-H]^-$ (224.1 calcd.); 1H NMR (400 MHz, DMSO) δ 10.67 (s, 1H), 7.57 (s, 1H), 6.76 (s, 1H), 4.17 – 4.08 (m, 1H), 3.79 (s, 2H), 1.14 (d, J = 6.6 Hz, 6H).

7-chloro-1-isopropyl-4-methyl-1,4-dihydropyrido[3,4-b]pyrazin-3(2H)-one

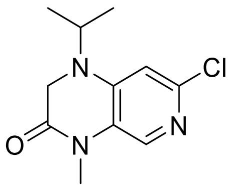

7-chloro-1-isopropyl-1,4-dihydropyrido[3,4-b]pyrazin-3(2H)-on (2.2 g, 9.7 mmol) and K_2CO_3 (1.75 g, 15.6 mmol) were added to DMF (23 mL) at rt. Subsequently, methyl iodide (2.08 g, 14.6 mmol) was added while stirring. After 16 h, HPLC confirmed a conversion into a new product and water was added to the reaction. A precipitate formed that was suction filtered and dried, yielding a brown solid (1.19 g, 51 %). 1H NMR (400 MHz, DMSO) δ 7.82 (s, 1H), 6.82 (s, 1H), 4.19 – 4.08 (m, 1H), 3.85 (d, J = 11.7 Hz, 2H), 3.26 (s, 3H), 1.15 (d, J = 6.6 Hz, 6H). 13C NMR (101 MHz, DMSO) δ 163.3, 145.7, 143.6, 133.6, 125.2, 104.0, 46.5, 43.6, 39.5, 27.9, 17.4.

7-((4-(benzyloxy)-3,5-difluorophenyl)amino)-1-isopropyl-4-methyl-1,4-dihydropyrido[3,4-b]pyrazin-3(2H)-one

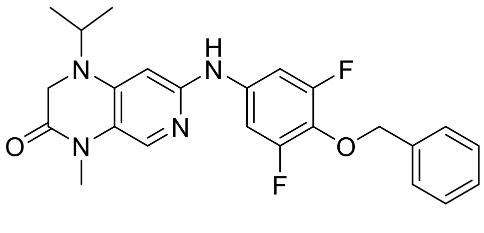

7-chloro-1-isopropyl-4-methyl-1,4-dihydropyrido[3,4-b]pyrazin-3(2H)-one (200 mg, 0.83 mmol), 4-(benzyloxy)-3,5-difluoroaniline (294 mg, 1.25 mmol), Xantphos Pd G3 (16 mg, 0.017 mmol) and KOtBu (187 mg, 1.67 mmol) were added to a dry flask with a stirrer in an argon atmosphere. Anhydrous 1,4-dioxane (11 mL) was added. The mixture was refluxated for 16 h in an argon atmosphere. After full conversion, the reaction mixture was filtered over celite, evaporated and purified using Flash chromatography yielding transparent yellow crystals (211 mg, 58 %). ESI-MS: *m/z* 439.7 $[M+H]^+$ (439.2 calcd.); 1H NMR (400 MHz, DMSO) δ 9.01 (s, 1H), 7.76 (s, 1H), 7.46 – 7.30 (m, 7H), 6.17 (s, 1H), 5.02 (s, 2H), 3.93 (hept, J = 6.5 Hz, 1H), 3.76 (s, 2H), 3.25 (s, 3H), 1.19 (d, J = 6.6 Hz, 6H). 13C NMR (101 MHz, DMSO) δ 163.3, 152.3, 143.3, 136.6, 131.6, 128.3, 128.2, 120.8, 100.5, 100.2, 91.8, 75.8, 46.4, 44.0, 39.5, 27.8, 17.4.

7-((3,5-difluoro-4-hydroxyphenyl)amino)-1-isopropyl-4-methyl-1,4-dihydropyrido[3,4-b]pyrazin-3(2H)-one (27)

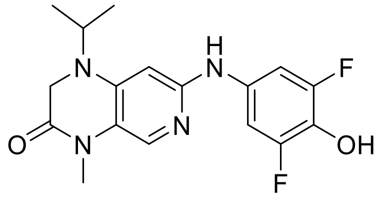

7-((4-(benzyloxy)-3,5-difluorophenyl)amino)-1-isopropyl-4-methyl-1,4-dihydropyrido[3,4-b]pyrazin-3(2H)-one (200 mg, 0.46 mmol) was dissolved in EtOH (5.0 mL). Pd/C (20 mg) was added. The mixture was stirred for 24 h in a hydrogen atmosphere. After full conversion, the reaction mixture was filtered over celite, evaporated and purified using Flash chromatography yielding a white solid (135 mg, 85 %). HPLC-DAD: 254 nm: 97.7 %, 230 nm: 96.3 %; ESI-MS: *m/z* [M+H]+: 349.14706 calcd.; 349.14704 found; 1H NMR (400 MHz, DMSO) δ 9.23 (s, 1H),

8.76 (s, 1H), 7.73 (s, 1H), 7.42 – 7.30 (m, 2H), 6.14 (s, 1H), 3.98 – 3.87 (m, 1H), 3.75 (s, 2H), 3.25 (s, 3H), 1.19 (d, J = 6.6 Hz, 6H). IR (ATR) [cm-1]: 2968, 2925, 1654, 1604, 1502, 1436, 1222, 1013, 803.

Synthesis of 1-cyclopropyl-7-((3,5-difluoro-4-hydroxyphenyl)amino)-4-methyl-1,4-dihydropyrido[3,4-b]pyrazin-3(2H)-one (28)

Ethyl cyclopropylglycinate

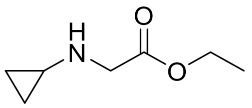

A mixture of cyclopropanamine 10.3 g, 180 mmol), TEA (7.3 g, 72 mmol) and DCM (240 mL) was cooled in an ice bath. Over 1 h, ethylbromoacetate (10.0 g, 60 mmol) was added and stirred overnight. The reaction mixture was filtered and the filtrate was washed with aqueous ammonia solution, dried over sodium sulfate and evaporated. Residual cyclopropanamine was removed by evaporation under reduced pressure giving the titled product as a colorless oil (5.5 g, 64 %). 1H NMR (400 MHz, CDCl3) δ 4.13 (q, J = 7.1 Hz, 2H), 3.38 (s, 2H), 2.21 – 2.12 (m, 1H), 2.01 (s, 1H), 1.22 (t, J = 7.1 Hz, 3H), 0.39 – 0.27 (m, 4H). 13C NMR (101 MHz, CDCl3) δ 172.9, 60.8, 50.8, 30.0, 14.4, 6.5.

Ethyl *N*-(2-chloro-5-nitropyridin-4-yl)-*N*-cyclopropylglycinate

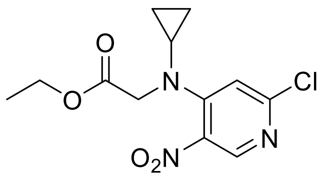

2,4-dichloro-5-nitropyridine (3.22 g, 16.7 mmol) and (2.66 g, 18.5 mmol) were dissolved in MeCN (20 mL) while stirring. TEA (3.75 g, 37.1 mmol) was slowly added. The reaction was stirred overnight at rt. The solvent was evaporated, the remains were taken up with EtOAc, washed with water and brine, dried over sodium sulfate, evaporated and purified using Flash chromatography giving the titled product as a yellow oil (4.0 g, 80 %). ESI-MS: *m/z* 322.0 $[M+Na]^+$ (322.1 calcd.); 1H NMR (400 MHz, CDCl3) δ 8.56 (s, 1H), 6.88 (s, 1H), 4.22 (q, J = 7.1 Hz, 2H), 4.14 (s, 2H), 2.99 – 2.93 (m, 1H), 1.29 (t, J = 7.1 Hz, 3H), 0.84 – 0.69 (m, 4H). 13C NMR (101 MHz, CDCl3) δ 168.9, 154.5, 150.3, 147.5, 136.2, 112.4, 62.1, 54.2, 36.1, 14.2, 9.6.

7-chloro-1-cyclopropyl-1,4-dihydropyrido[3,4-b]pyrazin-3(2H)-one

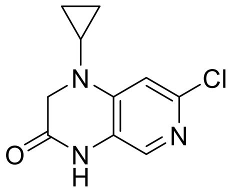

Ethyl *N*-(2-chloro-5-nitropyridin-4-yl)-*N*-cyclopropylglycinate (4.0 g, 13.3 mmol) was dissolved in glacial acetic acid (55 mL). The mixture was heated to 70 °C and powdered iron (3.73 g, 66.7 mmol) was added portion wise. The temperature raised to 110 °C and was kept stirring for 1 h. The solids were filtered, the filtrate was extracted 3x with EtOAc, The combined organic layers were washed with brine. After drying over sodium sulfate, it was evaporated and purified using Flash chromatography yielding a brown solid (2.86 g, 96 %). ESI-MS: *m/z* 221.9 $[M-H]^-$ (222.1 calcd.); 1H NMR (400 MHz, DMSO) δ 7.61 (s, 1H), 6.89 (s, 1H), 3.89 (s, 2H), 2.49 – 2.43 (m, 1H), 0.91 – 0.59 (m, 4H). 13C NMR (101 MHz, DMSO) δ 164.6, 144.4, 143.7, 133.2, 123.8, 105.8, 50.8, 21.3, 7.3.

7-chloro-1-cyclopropyl-4-methyl-1,4-dihydropyrido[3,4-b]pyrazin-3(2H)-one

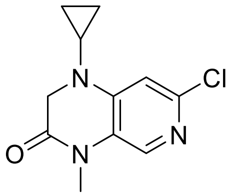

7-chloro-1-cyclopropyl-1,4-dihydropyrido[3,4-b]pyrazin-3(2H)-one (2.85 g, 12.7 mmol) and K_2CO_3 (2.29 g, 20.4 mmol) were added to DMF (30 mL) at rt. Subsequently, methyl iodide (2.71 g, 19.1 mmol) was added while stirring. After 16 h, HPLC confirmed a conversion into a new product and water was added to the reaction. A precipitate formed that was suction filtered and dried, yielding a brown solid (2.43 g, 80 %). 1H NMR (400 MHz, DMSO) δ 7.87 (s, 1H), 6.96 (s, 1H), 3.98 (s, 2H), 3.25 (s, 3H), 2.49 – 2.45 (m, 1H), 0.92 – 0.58 (m, 4H). 13C NMR (101 MHz, DMSO) δ 163.9, 145.4, 145.3, 133.6, 125.8, 105.8, 51.3, 29.9, 28.1, 7.5.

7-((4-(benzyloxy)-3,5-difluorophenyl)amino)-1-cyclopropyl-4-methyl-1,4-dihydropyrido[3,4-b]pyrazin-3(2H)-one

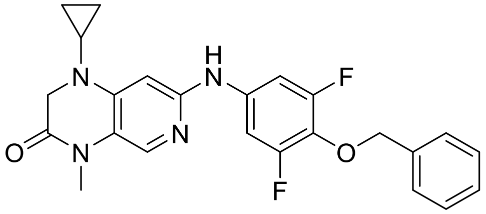

7-chloro-1-isobutyl-4-methyl-1,4-dihydropyrido[3,4-b]pyrazin-3(2H)-one (200 mg, 0.84 mmol), 4-(benzyloxy)-3,5-difluoroaniline (396 mg, 1.68 mmol), Brettphos Pd G3 (15 mg, 0.017 mmol) and KOtBu (189 mg, 1.68 mmol) were added to a dry flask with a stirrer in an argon atmosphere. Anhydrous 1,4-dioxane (11 mL) was added. The mixture was refluxated for 4 h in an argon atmosphere. After full conversion, the reaction mixture was filtered over celite,

evaporated and purified using Flash chromatography yielding a yellow-brown solid (120 mg, 33 %) that was used in the next step without further purification.

1-cyclopropyl-7-((3,5-difluoro-4-hydroxyphenyl)amino)-4-methyl-1,4-dihydropyrido[3,4-b]pyrazin-3(2H)-one (28)

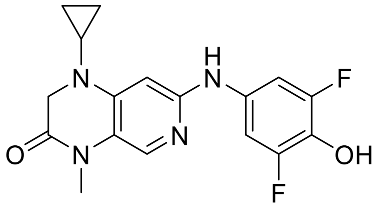

7-((4-(benzyloxy)-3,5-difluorophenyl)amino)-1-cyclopropyl-4-methyl-1,4-dihydropyrido[3,4-b]pyrazin-3(2H)-one (150 mg, 0.34 mmol) was dissolved in EtOH (5 mL) and conc. HCl (0.2 mL). Pd/C (15 mg) was added. The mixture was stirred for 24 h in a hydrogen atmosphere. After full conversion, the reaction mixture was filtered over a Celite pad, evaporated and purified using Flash chromatography yielding transparent brownish crystals (62 mg, 52 %). HPLC-DAD: 254 nm: 95.2 %, 230 nm: 94.6 %; ESI-HRMS: *m/z* [M+H]+: 347.13141 calcd.; 347.13169 found; 1H NMR (400 MHz, DMSO) δ 9.26 (s, 1H), 8.88 (s, 1H), 7.75 (s, 1H), 7.44 – 7.33 (m, 2H), 6.49 (s, 1H), 3.86 (s, 2H), 3.23 (s, 3H), 2.41 – 2.35 (m, 1H), 0.88 – 0.57 (m, 4H). 13C NMR (101 MHz, DMSO) δ 163.5, 152.4, 152.37 (dd, J = 237.6, 9.1 Hz), 144.8, 134.40 (t, J = 12.7 Hz), 131.5, 125.93 (t, J = 16.9 Hz), 120.5, 100.72 (dd, J = 18.0, 9.0 Hz), 93.0, 51.8, 29.8, 27.8, 7.3. IR (ATR) [cm-1]: 3290, 2921, 1645, 1608, 1504, 1354, 1215, 1013.

Synthesis of 1-(cyclopropylmethyl)-7-((3,5-difluoro-4-hydroxyphenyl)amino)-4-methyl-1,4-dihydropyrido[3,4-b]pyrazin-3(2H)-one (29)

Ethyl (cyclopropylmethyl)glycinate

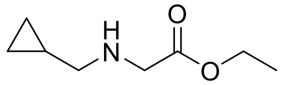

A mixture of cyclopropylmethanamine (7.86 g, 111 mmol), TEA (4.5 g, 44 mmol) and DCM (150 mL) was cooled in an ice bath. Over 1 h, ethylbromoacetate (6.15 g, 36.8 mmol) was added and stirred overnight. The reaction mixture was filtered and the filtrate was washed with aqueous ammonia solution, dried over sodium sulfate and evaporated. Residual cyclopropylmethanamine was removed by evaporation under reduced pressure giving the titled product as a colorless oil (4.86 g, 84 %). 1H NMR (400 MHz, CDCl3) δ 4.17 (q, J = 7.1 Hz, 2H), 3.41 (s, 2H), 2.46 (d, J = 6.9 Hz, 2H), 1.76 (s, 1H), 1.26 (t, J = 7.1 Hz, 3H), 0.97 – 0.86 (m, 1H), 0.51 – 0.09 (m, 4H). 13C NMR (101 MHz, CDCl3) δ 172.7, 60.8, 54.7, 50.8, 14.4, 11.2, 3.4.

Ethyl N-(2-chloro-5-nitropyridin-4-yl)-N-(cyclopropylmethyl)glycinate

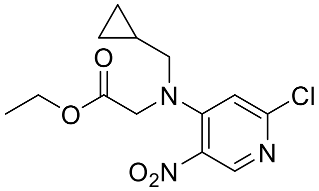

2,4-dichloro-5-nitropyridine (3.3 g, 17.2 mmol) and ethyl (cyclopropylmethyl)glycinate (3.0 g, 19.1 mmol). were dissolved in acetonitrile while stirring. TEA (3.86 g, 38.2 mmol) was slowly added. The reaction was stirred overnight at rt. The solvent was evaporated, the remains were taken up with EtOAc, washed with water and brine, dried over sodium sulfate, evaporated and purified using Flash chromatography giving the titled product as a yellow oil (4.1 g, 76 %). 1H

NMR (400 MHz, CDCl3) δ 8.58 (s, 1H), 6.87 (s, 1H), 4.24 (q, J = 7.1 Hz, 2H), 4.14 (s, 2H), 3.15 (d, J = 6.7 Hz, 2H), 1.30 (t, J = 7.1 Hz, 3H), 1.11 – 0.97 (m, 1H), 0.73 – 0.15 (m, 4H). 13C NMR (101 MHz, CDCl3) δ 168.8, 155.0, 150.6, 148.1, 135.7, 112.3, 61.9, 57.7, 51.7, 14.3, 8.4, 4.3.

7-chloro-1-(cyclopropylmethyl)-1,4-dihydropyrido[3,4-b]pyrazin-3(2H)-one

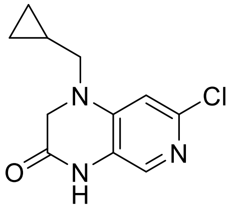

Ethyl N-(2-chloro-5-nitropyridin-4-yl)-N-(cyclopropylmethyl)glycinate (4.0 g, 12.7 mmol) was dissolved in glacial acetic acid (55 mL). The mixture was heated to 70 °C and powdered iron (3.56 g, 63.7 mmol) was added portion wise. The temperature raised to 110 °C and was kept stirring for 1 h. The solids were filtered, the filtrate was extracted 3x with EtOAc, The combined organic layers were washed with brine. After drying over sodium sulfate, it was evaporated and purified using Flash chromatography yielding a brown solid (2.77 g, 91 %). ESI-MS: *m/z* 235.9 $[M+H]^+$ (236.1 calcd.); 1H NMR (400 MHz, DMSO) δ 10.68 (s, 1H), 7.56 (s, 1H), 6.73 (s, 1H), 4.02 (s, 2H), 3.18 (d, J = 6.8 Hz, 2H), 1.06 – 0.94 (m, 1H), 0.56 – 0.22 (m, 4H). 13C NMR (101 MHz, DMSO) δ 163.8, 144.9, 142.3, 133.2, 122.9, 103.9, 52.1, 50.3, 7.1, 3.2.

7-chloro-1-(cyclopropylmethyl)-4-methyl-1,4-dihydropyrido[3,4-b]pyrazin-3(2H)-one

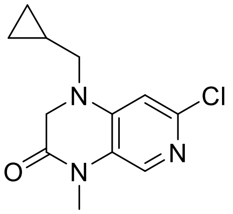

7-chloro-1-(cyclopropylmethyl)-1,4-dihydropyrido[3,4-b]pyrazin-3(2H)-one (2.76 g, 11.6 mmol) and K_2CO_3 (2.09 g, 18.6 mmol) were added to DMF (30 mL) at rt. Subsequently, methyl iodide (2.47 g, 17.4 mmol) was added while stirring. After 16 h, HPLC confirmed a conversion into a new product and water was added to the reaction. A precipitate formed that was suction filtered and dried, yielding a brown solid (1.90 g, 65 %). 1H NMR (400 MHz, DMSO) δ 7.81 (s, 1H), 6.78 (s, 1H), 4.11 (s, 2H), 3.27 (s, 3H), 3.19 (d, J = 6.8 Hz, 2H), 1.06 – 0.94 (m, 1H), 0.57 – 0.25 (m, 4H). 13C NMR (101 MHz, DMSO) δ 163.1, 145.6, 143.7, 133.4, 124.9, 103.8, 52.2, 50.4, 27.9, 7.0, 3.3.

7-((4-(benzyloxy)-3,5-difluorophenyl)amino)-1-(cyclopropylmethyl)-4-methyl-1,4-dihydropyrido[3,4-b]pyrazin-3(2H)-one

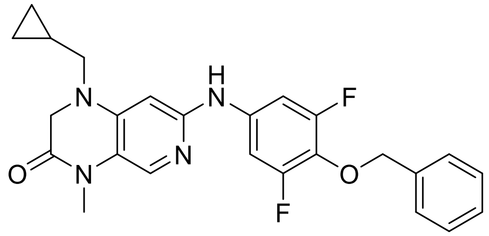

7-chloro-1-(cyclopropylmethyl)-4-methyl-1,4-dihydropyrido[3,4-b]pyrazin-3(2H)-one (200 mg, 0.79mmol), 4-(benzyloxy)-3,5-difluoroaniline (374 mg, 1.59 mmol), Brettphos Pd G3 (14 mg, 0.016 mmol) and KOtBu (178 mg, 1.59 mmol) were added to a dry flask with a stirrer in an argon atmosphere. Anhydrous 1,4-dioxane (11 mL) was added. The mixture was refluxated for 4 h in an argon atmosphere. After full conversion, the reaction mixture was filtered over celite, evaporated and purified using Flash chromatography yielding a yellow-brown solid (130 mg, 36 %) that was used in the next step without further purification.

1-(cyclopropylmethyl)-7-((3,5-difluoro-4-hydroxyphenyl)amino)-4-methyl-1,4-dihydropyrido[3,4-b]pyrazin-3(2H)-one (29)

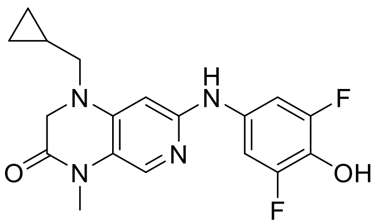

7-((4-(benzyloxy)-3,5-difluorophenyl)amino)-1-(cyclopropylmethyl)-4-methyl-1,4-dihydropyrido[3,4-b]pyrazin-3(2H)-one (130 mg, 0.29 mmol) was dissolved in EtOH (5 mL) and conc. HCl (0.2 mL). Pd/C (13 mg) was added. The mixture was stirred for 24 h in a hydrogen atmosphere. After full conversion, the reaction mixture was filtered over celite, evaporated and purified using Flash chromatography yielding an orange solid (75 mg, 72 %). HPLC-DAD: 254 nm: 98.2 %, 230 nm: 97.8 %; ESI-HRMS: *m/z* [M+H]+: 361.14706 calcd.; 361.14764 found; 1H NMR (400 MHz, DMSO) δ 9.25 (s, 1H), 8.76 (s, 1H), 7.72 (s, 1H), 7.44 – 7.29 (m, 1H), 6.14 (s, 1H), 4.00 (s, 2H), 3.25 (s, 3H), 3.10 (d, J = 6.7 Hz, 2H), 1.08 – 0.98 (m, 1H), 0.60 – 0.22 (m, 4H). 13C NMR (101 MHz, DMSO) δ 163.0, 152.7, 152.38 (dd, J = 237.5, 9.1 Hz), 143.3, 134.47 (t, J = 12.9 Hz), 131.5, 125.84 (t, J = 16.8 Hz), 120.1, 100.62 (dd, J = 17.7, 9.3 Hz), 91.0, 52.4, 51.0, 27.7, 6.7, 3.3. IR (ATR) [cm-1]: 3298, 2920, 1646, 1607, 1502, 1216, 1010, 801.

Synthesis of 7-((3,5-difluoro-4-hydroxyphenyl)amino)-4-methyl-1-propyl-1,4-dihydropyrido[3,4-b]pyrazin-3(2H)-one (30)

Ethyl isobutylglycinate

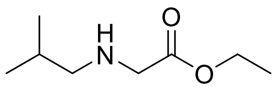

A mixture of 2-methylpropan-1-amine (17.5 g, 240 mmol) and toluene (240 mL) was cooled in an ice bath. Over 2 h, ethylbromoacetate (10.0 g, 60 mmol) was added. The reaction mixture was filtered and the filtrate was concentrated under reduced pressure. The organic layer was washed with 10% ammonia solution and dried over sodium sulfate. Residual 2-methylpropan-1-amine was co-evaporated with toluene under reduced pressure giving the titled product as a colorless oil (8.9 g, 93 %). 1H NMR (400 MHz, CDCl3) δ 4.15 (q, J = 7.1 Hz, 2H), 3.35 (s, 2H), 2.38 (d, J = 6.8 Hz, 2H), 1.76 – 1.65 (m, 1H), 1.24 (t, J = 7.1 Hz, 3H), 0.89 (d, J = 6.7 Hz, 6H).

Ethyl N-(2-chloro-5-nitropyridin-4-yl)-N-isobutylglycinate

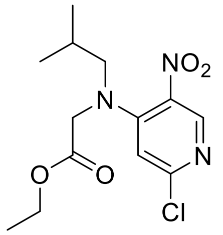

2,4-dichloro-5-nitropyridine (3.03 g, 15.7 mmol) and ethyl isobutylglycinate (2.5 g, 15.7 mmol) were dissolved in MeCN (30 mL) while stirring. TEA (4.4 mL, 31.4 mmol) was slowly added. The reaction was stirred overnight at RT. The solvent was evaporated, the remains were taken up with EtOAc, washed with water and brine, dried over sodium sulfate and evaporated giving a yellow oil (4.0 g, 81 %) that was used without further purification. ESI-MS: *m/z* 338.6

$[M+Na]^+$ (338.1 calcd.); 1H NMR (400 MHz, DMSO) δ 8.59 (s, 1H), 7.35 (s, 1H), 4.20 (s, 2H), 4.09 (q, J = 7.1 Hz, 2H), 3.20 (d, J = 7.4 Hz, 2H), 1.95 – 1.83 (m, 1H), 1.16 (t, J = 7.1 Hz, 3H), 0.83 (d, J = 6.6 Hz, 6H). 13C NMR (101 MHz, DMSO) δ 168.2, 153.2, 149.7, 147.5, 112.5, 60.8, 59.4, 53.7, 26.3, 19.4, 13.8.

7-chloro-1-isobutyl-1,4-dihydropyrido[3,4-b]pyrazin-3(2H)-one

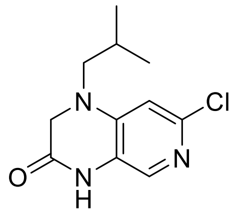

Ethyl N-(2-chloro-5-nitropyridin-4-yl)-N-isobutylglycinate (4.0 g, 12.7 mmol) was dissolved in glacial acetic acid (55 mL). The mixture was heated to 70 °C and powdered iron (3.5 g, 63.3 mmol) was added portionwise. The temperature raised to 110 °C and was kept stirring for 1 h. The solids were filtered, the filtrate was extracted 3x with EtOAc, The combined organic layers were washed with brine. After drying over sodium sulfate, it was evaporated and purified using Flash chromatography yielding a brown solid (1.34 g, 38 %). ESI-MS: *m/z* 240.3 $[M+H]^+$ (240.1 calcd.); 1H NMR (400 MHz, DMSO) δ 10.67 (s, 1H), 7.53 (s, 1H), 6.69 (s, 1H), 3.95 (s, 2H), 3.10 (d, J = 7.6 Hz, 2H), 2.07 – 1.95 (m, 1H), 0.89 (d, J = 6.6 Hz, 6H).

7-chloro-1-isobutyl-4-methyl-1,4-dihydropyrido[3,4-b]pyrazin-3(2H)-one

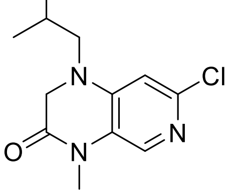

7-chloro-1-isobutyl-1,4-dihydropyrido[3,4-b]pyrazin-3(2H)-one (1.22 g, 5.1 mmol) and K_2CO_3 (914 mg, 8.1 mmol) were added to DMF (13 mL) at rt. Subsequently, methyl iodide (1.08 g, 7.6 mmol) was added while stirring. After 16 h, HPLC confirmed a conversion into a new product and water was added to the reaction. A precipitate formed that was suction filtered and dried, yielding a brown solid (706 mg, 55 %). 1H NMR (400 MHz, DMSO) δ 8.23 (s, 1H), 7.51 (s, 1H), 4.33 (s, 2H), 4.00 (s, 3H), 3.41 (d, J = 7.7 Hz, 2H), 2.16 – 2.04 (m, 1H), 0.92 (d, J = 6.6 Hz, 6H).

7-((4-(benzyloxy)-3,5-difluorophenyl)amino)-1-isobutyl-4-methyl-1,4-dihydropyrido[3,4-b]pyrazin-3(2H)-one

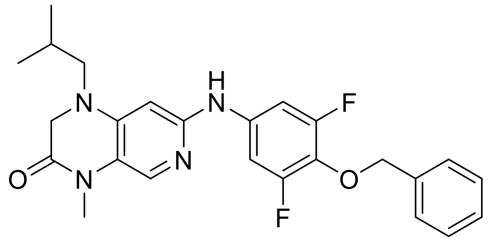

7-chloro-1-isobutyl-4-methyl-1,4-dihydropyrido[3,4-b]pyrazin-3(2H)-one (170 mg, 0.67 mmol), 4-(benzyloxy)-3,5-difluoroaniline (315 mg, 1.34 mmol) Brettphos Pd G3 (12 mg, 0.013 mmol) and KOtBu (150 mg, 1.34 mmol) were added to a dry flask with a stirrer in an argon atmosphere. Anhydrous 1,4-dioxane (10 mL) was added. The mixture heated to 80 °C for 4 h in an argon atmosphere. After full conversion, the reaction mixture was filtered over celite, evaporated and purified using Flash chromatography yielding a dark brown solid (146 mg, 48 %) that was used in the next step without further purification.

7-((3,5-difluoro-4-hydroxyphenyl)amino)-4-methyl-1-propyl-1,4-dihydropyrido[3,4-b]pyrazin-3(2H)-one (30)

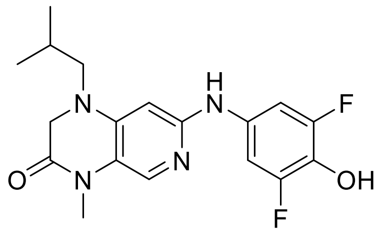

7-((4-(benzyloxy)-3,5-difluorophenyl)amino)-1-isobutyl-4-methyl-1,4-dihydropyrido[3,4-b]pyrazin-3(2H)-one (146 mg, 0.32 mmol) was dissolved in EtOH (5 mL) and conc. HCl (0.2 mL). Pd/C was added. The mixture was stirred for 24 h in a hydrogen atmosphere. After full conversion, the reaction mixture was filtered over celite, evaporated and purified using Flash chromatography yielding a gray solid (82 mg, 70 %). HPLC-DAD: 254 nm: 97.6 %, 230 nm: 96.9 %; ESI-HRMS: *m/z* [M+H]+: 363.16271 calcd.; 363.16287 found; 1H NMR (400 MHz, DMSO) δ 10.12 (s, 1H), 9.88 (s, 1H), 7.41 (s, 1H), 7.04 (d, J = 8.5 Hz, 1H), 6.21 (s, 1H), 4.21 (s, 2H), 3.22 (s, 3H), 3.15 (d, J = 6.4 Hz, 2H), 2.12 – 1.96 (m, 1H), 0.91 (d, J = 6.6 Hz, 6H). 13C NMR (101 MHz, DMSO) δ 161.3, 152.55 (dd, J = 241.7, 8.8 Hz), 148.4, 146.3, 131.07 – 130.65 (m), 129.13 – 128.62 (m), 120.2, 107.05 – 106.48 (m), 88.1, 56.6, 51.8, 27.9, 25.4, 19.9. IR (ATR) [cm-1]: 2958, 1689, 1610, 1559, 1517, 1468, 1437, 1357, 1314.

Synthesis of 1-cyclobutyl-7-((3,5-difluoro-4-hydroxyphenyl)amino)-4-methyl-1,4-dihydropyrido[3,4-b]pyrazin-3(2H)-one (31)

4,6-dichloropyridin-3-amine

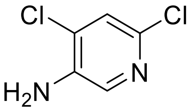

2,4-dichloro-5-nitropyridine (25 g, 0.13 mol) was dissolved in anhydrous acetic acid (350 mL). The mixture was heated to 60 °C and powdered iron (21.7 g, 0.39 mol) was added. The temperature raised while stirring vigorously. After 20 min, the temperature decreased. After another 20 min, the reaction mixture was poured in water and extracted 3x with EtOAc. The combined organic layers were washed 2x with 10% ammonia in water solution and brine, dried over sodium sulfate, evaporated. The residual oil was taken up in a mixture of hexane/diethyl ether and slowly evaporated, giving a white precipitate (17.75 g, 85 %). ESI-MS: *m/z* 162.9 $[M+H]^+$ (162.9 calcd.); 1H NMR (400 MHz, CDCl3) δ 7.91 (s, 1H), 7.23 (s, 1H), 3.98 (s, 2H). 13C NMR (101 MHz, CDCl3) δ 139.8, 139.3, 136.3, 129.8, 124.0.

2-chloro-*N*-(4,6-dichloropyridin-3-yl)acetamide

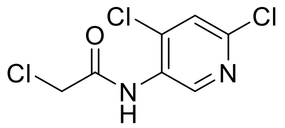

4,6-dichloropyridin-3-amine (4.3 g, 22.3 mmol) was dissolved in 90 mL dichloromethane and 45 mL water and K_2CO_3 (6.16 g, 44.6 mmol) was added. The reaction mixture was cooled to 0° C and chloroacetyl chloride (5.03 g, 44.6 mmol) was added dropwise. After 30 minutes the organic phase was separated and evaporated giving the desired product as a white substance without further purification (5.1 g, 96 %). ESI-MS: *m/z* 238.9 $[M+H]^+$ (238.9 calcd.); 1H NMR (400 MHz, CDCl3) δ 9.32 (s, 1H), 8.74 (s, 1H), 7.42 (s, 1H), 4.25 (s, 2H). 13C NMR (101 MHz, CDCl3) δ 164.0, 146.9, 142.5, 134.9, 130.4, 124.2, 43.0.

2-chloro-*N*-(4,6-dichloropyridin-3-yl)-*N*-methylacetamide

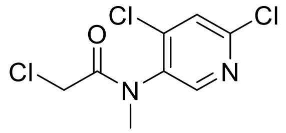

2-chloro-*N*-(4,6-dichloropyridin-3-yl)acetamide (5.29 g, 22.1 mmol) and K_2CO_3 (6.1 g, 44.2 mmol) were added to DMF (50 mL) at rt. Subsequently, methyl iodide (6.27 g, 44.2 mmol) was added while stirring. After 4 h, HPLC confirmed a conversion into a new product and water was added to the reaction. The mixture was extracted 3x with EtOAc. The combined organic layers were washed with water and brine, dried over sodium sulfate, evaporated, and purified using Flash chromatography yielding a yellow oil (2.7 g, 49 %). 1H NMR (400 MHz, CDCl3) δ 8.50 (s, 1H), 7.55 (s, 1H), 3.47 (dd, J = 119.6, 10.2 Hz, 2H), 3.23 (s, 3H). 13C NMR (101 MHz, CDCl3) δ 167.9, 152.2, 150.0, 144.6, 136.8, 125.9, 37.2, -4.4.

2-(cyclobutylamino)-*N*-(4,6-dichloropyridin-3-yl)-*N*-methylacetamide

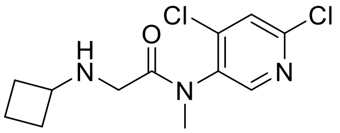

2-chloro-*N*-(4,6-dichloropyridin-3-yl)-*N*-methylacetamide (1.0 g, 3.9 mmol) was placed in THF and combined with K_2CO_3 (1.36 g, 9.9 mmol) and cyclobutylamine (0.56 g, 7.9 mmol). The reaction mixture was stirred for 2 h at rt., then diluted with water. The aqueous phase was extracted twice with ethyl acetate. The combined organic phases were dried over sodium sulfate, evaporated and purified using Flash chromatography giving a brown oil (0.93 g, 82 %). ESI-MS: *m/z* 288.0 $[M+H]^+$ (288.1 calcd.).

7-chloro-1-cyclobutyl-4-methyl-1,4-dihydropyrido[3,4-b]pyrazin-3(2H)-one

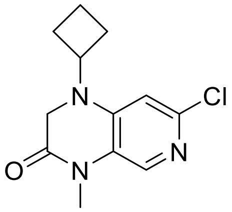

2-(cyclobutylamino)-*N*-(4,6-dichloropyridin-3-yl)-*N*-methylacetamide (870 mg, 3 mmol) and DIPEA (1.17 g, 9.1 mmol) were dissolved in DMF (15 mL). The reaction was stirred for 4 h at 100 °C. Upon completion, water was added and the precipitate was suction filtered and dried giving purely the desired product was a brown solid (650 mg, 86 %). ESI-MS: *m/z* 252.2 $[M+H]^+$ (252.1 calcd.); 1H-NMR (400 MHz, CDCl3): δ = 7.78 (s, 1H), 6.49 (s, 1H), 3.92 (quint, J = 8.2 Hz, 1H), 3.83 (s, 2H), 3.36 (s, 3H), 2.40 – 1.75 (m) ppm. 13C NMR (101 MHz, CDCl3) δ 164.2, 146.9, 144.0, 133.5, 125.9, 106.1, 52.8, 47.3, 28.6, 27.7, 14.9.

7-((4-(benzyloxy)-3,5-difluorophenyl)amino)-1-cyclobutyl-4-methyl-1,4-dihydropyrido[3,4-b]pyrazin-3(2H)-one

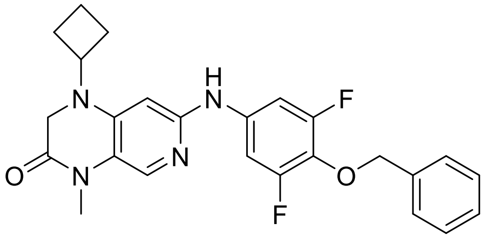

7-chloro-1-cyclobutyl-4-methyl-1,4-dihydropyrido[3,4-b]pyrazin-3(2H)-one (300 mg, 1.19 mmol), 4-(benzyloxy)-3,5-difluoroaniline (561 mg, 2.38 mmol), Brettphos Pd G3 (22 mg, 0.024 mmol) and KOtBu (267 mg, 2.38 mmol) were added to a dry flask with a stirrer in an argon atmosphere. Anhydrous 1,4-dioxane (17 mL) was added. The mixture was heated to 80 °C for 4 h in an argon atmosphere. After full conversion, the reaction mixture was filtered over

celite, evaporated and purified using Flash chromatography yielding a brown solid (164 mg, 31 %) that was used in the next step without further purification. ESI-MS: *m/z* 451.4 $[M+H]^+$ (451.2 calcd.).

1-cyclobutyl-7-((3,5-difluoro-4-hydroxyphenyl)amino)-4-methyl-1,4-dihydropyrido[3,4-b]pyrazin-3(2H)-one (31)

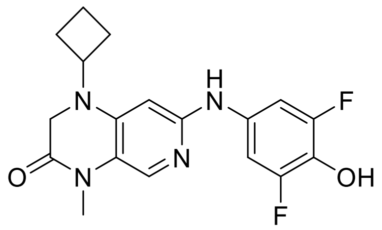

7-((4-(benzyloxy)-3,5-difluorophenyl)amino)-1-cyclobutyl-4-methyl-1,4-dihydropyrido[3,4-b]pyrazin-3(2H)-one (164 mg, 0.36 mmol) was dissolved in EtOH (5 mL) and conc. HCl (0.2 mL). Pd/C (16 mg) was added. The mixture was stirred for 24 h in a hydrogen atmosphere. After full conversion, the reaction mixture was filtered over celite, evaporated and purified using Flash chromatography yielding brown crystals (90 mg, 69 %). HPLC-DAD: 254 nm: 100 %, 230 nm: 97.3 %; ESI-HRMS: *m/z* [M+H]+: 361.14706 calcd.; 361.14721 found; 1H NMR (400 MHz, DMSO) δ 9.29 (s, 1H), 8.78 (s, 1H), 7.74 (s, 1H), 7.40 – 7.30 (m, 2H), 6.05 (s, 1H), 3.90 – 3.80 (m, 1H), 3.71 (s, 2H), 3.24 (s, 3H), 2.32 – 2.22 (m, 2H), 2.16 – 2.01 (m, 2H), 1.78 – 1.67 (m, 2H). 13C NMR (101 MHz, DMSO) δ 163.7, 152.5, 152.39 (dd, J = 237.7, 9.0 Hz), 143.4, 134.36 (t, J = 12.8 Hz), 131.5, 125.95 (t, J = 16.7 Hz), 120.7, 100.68 (dd, J = 17.9, 9.0 Hz), 92.6, 52.2, 47.0, 27.8, 27.0, 14.4. IR (ATR) [cm-1]: 3214, 2952, 2852, 1643, 1612, 1423, 1231, 1019, 1011, 802.

Synthesis of 1-cyclopentyl-7-((3,5-difluoro-4-hydroxyphenyl)amino)-4-methyl-1,4-dihydropyrido[3,4-b]pyrazin-3(2H)-one (32)

Ethyl cyclopentylglycinate

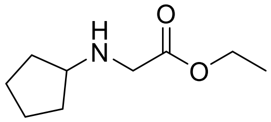

A mixture of cyclopentylamine (23.8 g, 240 mmol) and toluene (40 mL) was cooled in an ice bath. Over 2 h, ethylbromoacetate (10 g, 60 mmol) was added. The reaction mixture was filtered and the filtrate was concentrated under reduced pressure. The organic layer was washed with 10 % ammonia solution and dried over sodium sulfate. Residual cyclopentylamine was removed by co-evaporation with toluene. This gave the titled product as a colorless oil (8.53 g, 77 %). ESI-MS: *m/z* 172.3 $[M+H]^+$ (172.1 calcd.); 1H NMR (400 MHz, CDCl3) δ 4.14 (q, J = 7.1 Hz, 2H), 3.34 (s, 2H), 3.02 (q, J = 6.4 Hz, 1H), 1.80 – 1.62 (m, 5H), 1.54 – 1.44 (m, J = 13.3, 6.5 Hz, 2H), 1.36 – 1.27 (m, J = 13.5, 7.1 Hz, 2H), 1.23 (t, J = 7.2 Hz, 3H). 13C NMR (101 MHz, CDCl3) δ 172.74, 60.74, 59.48, 49.93, 33.06, 24.04, 14.29.

Ethyl *N*-(2-chloro-5-nitropyridin-4-yl)-N-cyclopentylglycinate

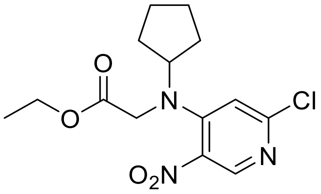

2,4-dichloro-5-nitropyridine (2.54 g, 13.1 mmol) and ethyl cyclopentylglycinate (2.5 g, 14.6 mmol) were dissolved in MeCN (30 mL) while stirring. TEA (4.1 mL, 29.2 mmol) was slowly added. The reaction was stirred overnight at 60 °C. The solvent was evaporated, the remains

were taken up with EtOAc, washed with water and brine, dried over sodium sulfate, evaporated and purified using Flash chromatography. The product was received quantitatively as a yellow oil (4.3 g). ESI-MS: *m/z* 350.4 $[M+Na]^+$ (350.1 calcd.).

7-chloro-1-cyclopentyl-1,4-dihydropyrido[3,4-b]pyrazin-3(2H)-one

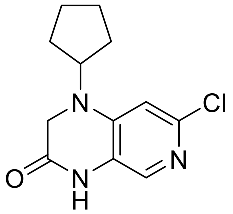

Ethyl *N*-(2-chloro-5-nitropyridin-4-yl)-*N*-cyclopentylglycinate (4.3 g, 13.1 mmol) was dissolved in glacial acetic acid (60 mL). The mixture was heated to 70 °C and powdered iron (3.6 g, 65.3 mmol) was added portion wise. The temperature raised to 110 °C and was kept stirring for 1 h. The solids were filtered, the filtrate was extracted 3x with EtOAc, The combined organic layers were washed with brine. After drying over sodium sulfate, it was evaporated and purified using Flash chromatography yielding a brown solid (1.35 g, 41 %). ESI-MS: *m/z* 252.2 $[M-H]^-$ (252.1 calcd.); 1H NMR (400 MHz, DMSO) δ 10.68 (s, 1H), 7.57 (s, 1H), 6.82 (s, 1H), 4.29 – 4.19 (m, 1H), 3.81 (s, 2H), 1.89 – 1.78 (m, 2H), 1.71 – 1.54 (m, 6H).

7-chloro-1-cyclopentyl-4-methyl-1,4-dihydropyrido[3,4-b]pyrazin-3(2H)-one

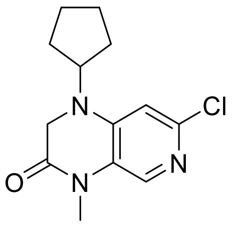

7-chloro-1-cyclopentyl-1,4-dihydropyrido[3,4-b]pyrazin-3(2H)-on (1.34 g, 5.3 mmol) and K_2CO_3 (956 mg, 8.52 mmol) were added to DMF (14 mL) at rt. Subsequently, methyl iodide

(0.5 mL, 8.0 mmol) was added while stirring. After 16 h, HPLC confirmed a conversion into a new product and water was added to the reaction. A precipitate formed that was suction filtered and dried, yielding a brown solid (1.18 g, 83 %) that was used without further purification.

7-((4-(benzyloxy)-3,5-difluorophenyl)amino)-1-cyclopentyl-4-methyl-1,4-dihydropyrido[3,4-b]pyrazin-3(2H)-one

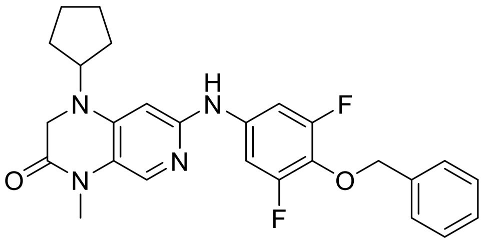

7-chloro-1-cyclopentyl-4-methyl-1,4-dihydropyrido[3,4-b]pyrazin-3(2H)-one (200 mg, 0.73 mmol), 4-(benzyloxy)-3,5-difluoroaniline (354 mg, 1.51 mmol), Xanphos Pd G3 (14.3 mg, 0.015 mmol) and K2CO3 (208 mg, 1.51 mmol) were added to a dry flask with a stirrer in an argon atmosphere. 1,4-dioxane and tBuOH (3:1, 15 mL) were added. The mixture was refluxated for 16 h in an argon atmosphere. After full conversion, the reaction mixture was filtered over celite, evaporated and purified using Flash chromatography yielding transparent yellow crystals (210 mg, 60 %) that were used without further purification.

1-cyclopentyl-7-((3,5-difluoro-4-hydroxyphenyl)amino)-4-methyl-1,4-dihydropyrido[3,4-b]pyrazin-3(2H)-one (32)

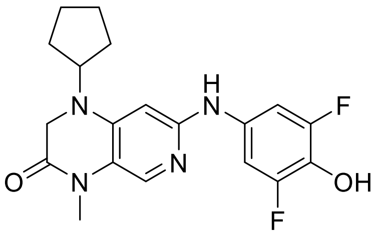

7-((4-(benzyloxy)-3,5-difluorophenyl)amino)-1-cyclopentyl-4-methyl-1,4-dihydropyrido[3,4-b]pyrazin-3(2H)-one (210 mg, 0.45 mmol) was dissolved in EtOH (5 mL). Pd/C (21 mg) was added. The mixture was stirred for 24 h in a hydrogen atmosphere. After full conversion, the reaction mixture was filtered over celite, evaporated and purified using Flash chromatography yielding a brown solid (38 mg, 22 %). HPLC-DAD: 254 nm: 96.2 %, 230 nm: 95.7 %; ESI-HRMS: *m/z* [M+H]+: 375.16271 calcd.; 375.16296 found; 1H NMR (400 MHz, DMSO) δ 9.59 (s, 1H), 9.05 (s, 1H), 7.60 (s, 1H), 7.30 – 7.21 (m, 2H), 6.23 (s, 1H), 4.12 – 4.03 (m, 1H), 3.89 (s, 2H), 3.24 (s, 3H), 1.93 – 1.81 (m 2H), 1.79 – 1.53 (m, 6H). IR (ATR) [cm-1]: 2950, 2870, 1663, 1603, 1502, 1436, 1220, 1185, 1008.

Synthesis of 7-((3,5-difluoro-4-hydroxyphenyl)amino)-4-methyl-1-((1s,4s)-4-methylcyclohexyl)-1,4-dihydropyrido[3,4-b]pyrazin-3(2H)-one (33)

Ethyl ((1r,4r)-4-methylcyclohexyl)glycinate

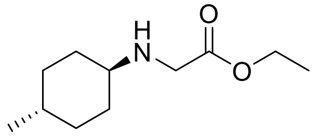

A mixture of (1r,4r)-4-methylcyclohexan-1-amine (5.08 g, 44.9 mmol), TEA (2.5 mL, 18.0 mmol) and toluene (60 mL) was cooled in an ice bath. Over 2 h, ethylbromoacetate (2.5 g, 15.0 mmol) was added. The reaction mixture was filtered and the filtrate was concentrated under reduced pressure. The organic layer was washed with 10 % ammonia solution and dried over sodium sulfate. Residual (1r,4r)-4-methylcyclohexan-1-amine was co-evaporated with toluene under reduced pressure giving the titled product in a crude colorless oil (2.83 g, 95 %) that was used without further purification.

Ethyl-*N*-(2-chloro-5-nitropyridin-4-yl)-*N*-((1s,4s)-4-methylcyclohexyl)glycinate

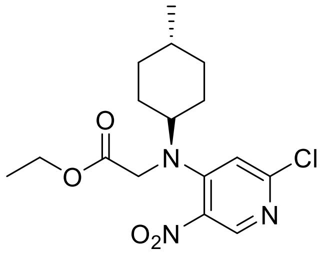

2,4-dichloro-5-nitropyridine (2.32 g, 12.0 mmol) and ethyl ((1r,4r)-4-methylcyclohexyl)glycinate (2.66 g, 13.3 mmol) were dissolved in MeCN (20 mL) while stirring. TEA (3.7 mL, 26.7 mmol) was slowly added. The reaction was stirred overnight at RT. The solvent was evaporated, the remains were taken up with EtOAc, washed with water and brine, dried over sodium sulfate, evaporated and purified using Flash chromatography giving the titled product as an orange oil (2.85 g, 67 %). ESI-MS: *m/z* 378.8 $[M+Na]^+$ (378.2 calcd.); 1H NMR (400 MHz, DMSO) δ 8.53 (s, 1H), 7.19 (s, 1H), 4.26 (s, 2H), 4.07 (q, J = 7.1 Hz, 2H), 3.38 – 3.32 (m, 1H), 1.69 (m), 1.61 – 1.48 (m,), 1.36 – 1.27 (m,), 1.20 – 1.07 (m), 1.04 – 0.92 (m), 0.84 (d, J = 6.5 Hz, 3H).

7-chloro-1-((1s,4s)-4-methylcyclohexyl)-1,4-dihydropyrido[3,4-b]pyrazin-3(2H)-one

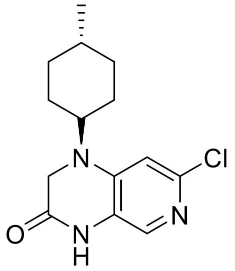

Ethyl-*N*-(2-chloro-5-nitropyridin-4-yl)-*N*-((1s,4s)-4-methylcyclohexyl)glycinate (2.9 g, 8.0 mmol) was dissolved in glacial acetic acid (40 mL). The mixture was heated to 70 °C and powdered iron (2.2 g, 40 mmol) was added portion wise. The temperature raised to 110 °C and

was kept stirring for 1 h. The solids were filtered, the filtrate was extracted 3x with EtOAc, The combined organic layers were washed with brine. After drying over sodium sulfate, it was evaporated and purified using Flash chromatography yielding a brown solid (1.64 g, 73 %). ESI-MS: *m/z* 278.1 $[M-H]^-$ (278.1 calcd.); 1H NMR (400 MHz, DMSO) δ 10.67 (s, 1H), 7.55 (s, 1H), 6.74 (s, 1H), 3.82 (s, 2H), 3.71 – 3.61 (m, 1H), 1.71 (d, J = 11.9 Hz, 2H), 1.65 – 1.50 (m, 4H), 1.39 – 1.28 (m, 1H), 1.24 – 1.11 (m, 2H), 0.88 (d, J = 6.4 Hz, 3H).

7-chloro-4-methyl-1-((1s,4s)-4-methylcyclohexyl)-1,4-dihydropyrido[3,4-b]pyrazin-3(2H)-one

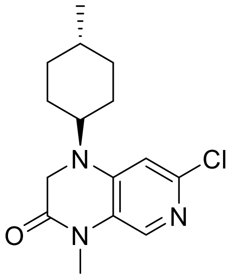

7-chloro-1-((1s,4s)-4-methylcyclohexyl)-1,4-dihydropyrido[3,4-b]pyrazin-3(2H)-one (1.64 g, 5.9 mmol) and K_2CO_3 (1.05 g, 9.4 mmol) were added to DMF (17 mL) at rt. Subsequently, methyl iodide (1.25 g, 8.8 mmol) was added while stirring. After 16 h, HPLC confirmed a conversion into a new product and water was added to the reaction. A precipitate formed that was suction filtered and dried, yielding a brown solid (1.49 g, 87 %). 1H NMR (400 MHz, DMSO) δ 7.81 (s, 1H), 6.81 (s, 1H), 3.90 (s, 2H), 3.73 – 3.64 (m, 1H), 3.25 (s, 3H), 1.74 – 1.68 (m, 2H), 1.65 – 1.50 (m, 4H), 1.40 – 1.29 (m, 1H), 1.25 – 1.13 (m, 2H), 0.88 (d, J = 6.4 Hz, 3H).

7-((4-(benzyloxy)-3,5-difluorophenyl)amino)-4-methyl-1-((1s,4s)-4-methylcyclohexyl)-1,4-dihydropyrido[3,4-b]pyrazin-3(2H)-on

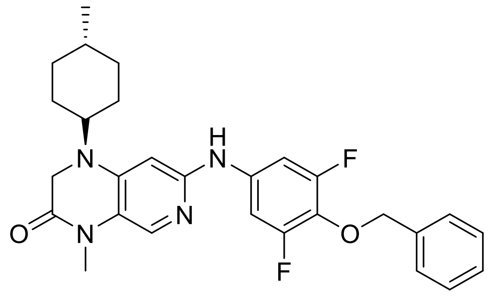

7-chloro-4-methyl-1-((1s,4s)-4-methylcyclohexyl)-1,4-dihydropyrido[3,4-b]pyrazin-3(2H)-one (200 mg, 0.68 mmol), 4-(benzyloxy)-3,5-difluoroaniline (320 mg, 1.36 mmol), Brettphos Pd G3 (12 mg, 0.014 mmol) and KOtBu (153 mg, 1.36 mmol) were added to a dry flask with a stirrer in an argon atmosphere. Anhydrous 1,4-dioxane (11 mL) was added. The mixture was heated for 4 h at 80 °C in an argon atmosphere. After full conversion, the reaction mixture was filtered over celite, evaporated and purified using Flash chromatography yielding an off-white solid material (200 mg, 60 %) that was used in the next step without further purification.

7-((3,5-difluoro-4-hydroxyphenyl)amino)-4-methyl-1-((1s,4s)-4-methylcyclohexyl)-1,4-dihydropyrido[3,4-b]pyrazin-3(2H)-one (33)

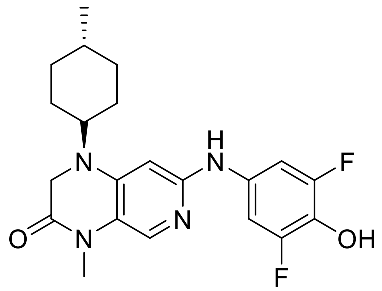

7-((4-(benzyloxy)-3,5-difluorophenyl)amino)-4-methyl-1-((1s,4s)-4-methylcyclohexyl)-1,4-dihydropyrido[3,4-b]pyrazin-3(2H)-on (200 mg, 0.41 mmol) was dissolved in EtOH (5 mL) and

conc. HCl (0.2 mL). Pd/C (20 mg) was added. The mixture was stirred for 24 h in a hydrogen atmosphere. After full conversion, the reaction mixture was filtered over celite, evaporated and purified using Flash chromatography yielding a gray solid (132 mg, 81 %). HPLC-DAD: 254 nm: 98.5 %, 230 nm: 97.3 %; ESI-HRMS: *m/z* [M+H]+: 403.19401 calcd.; 403.19416 found; 1H NMR (400 MHz, DMSO) δ 9.27 (s, 1H), 8.72 (s, 1H), 7.72 (s, 1H), 7.40 – 7.31 (m, 2H), 6.13 (s, 1H), 3.78 (s, 2H), 3.48 – 3.38 (m, 1H), 3.24 (s, 3H), 1.79 (d, J = 12.0 Hz, 2H), 1.69 (d, J = 10.1 Hz, 2H), 1.58 (qd, J = 12.3, 3.0 Hz, 2H), 1.44 – 1.32 (m, 1H), 1.08 (qd, J = 13.0, 3.1 Hz, 2H), 0.90 (d, J = 6.5 Hz, 3H). 13C NMR (101 MHz, DMSO) δ 163.2, 152.8, 152.42 (dd, J = 237.6, 9.1 Hz), 134.5 (t, J = 12.7 Hz), 131.8, 125.84 (t, J = 16.7 Hz), 100.57 (dd, J = 18.0, 9.2 Hz), 91.3, 55.0, 45.3, 34.0, 31.4, 27.8, 27.0, 22.2. IR (ATR) [cm-1]: 3258, 2920, 2851, 1647, 1500, 1230, 1216, 1023.

Synthesis of 1-(cyclohexylmethyl)-7-((3,5-difluoro-4-hydroxyphenyl)amino)-4-methyl-1,4-dihydropyrido[3,4-b]pyrazin-3(2H)-one (34)

Ethyl (cyclohexylmethyl)glycinate

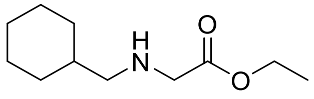

A mixture of cyclohexylmethanamine (19.0 g, 168 mmol) and toluene (170 mL) was cooled in an ice bath. Over 2 h, ethylbromoacetate (7.0 g, 42 mmol) was added. The reaction mixture was filtered and the filtrate was concentrated under reduced pressure. The organic layer was washed with 10 % ammonia solution and dried over sodium sulfate. Residual cyclohexylmethanamine was co-evaporated with toluene under reduced pressure giving the titled product as a colorless oil

(7.2 g, 86 %). 1H NMR (400 MHz, CDCl3) δ 4.16 (q, J = 7.1 Hz, 2H), 3.35 (s, 2H), 2.40 (d, J = 6.7 Hz, 2H), 1.80 – 0.82 (m).

Ethyl *N*-(2-chloro-5-nitropyridin-4-yl)-*N*-cyclomethylhexylglycinate

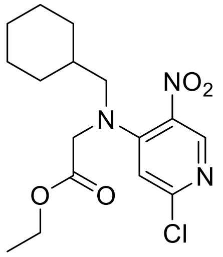

2,4-dichloro-5-nitropyridine (3.87 g, 20.1 mmol) and ethyl (cyclohexylmethyl)glycinate (4.0 g, 20.1 mmol) were dissolved in MeCN (40 mL) while stirring. TEA (5.6 mL, 40.1 mmol) was slowly added. The reaction was stirred overnight at RT. The solvent was evaporated, the remains were taken up with EtOAc, washed with water and brine, dried over sodium sulfate, evaporated and purified using Flash chromatography giving the titled product as an orange oil (4.44 g, 62 %). ESI-MS: *m/z* 378.5 $[M+Na]^+$ (378.1 calcd.); 1H NMR (400 MHz, CDCl3) δ 8.59 (s, 1H), 6.96 (s, 1H), 4.18 (q, J = 7.1 Hz, 2H), 3.91 (s, 2H), 3.19 (d, J = 7.2 Hz, 2H), 1.80 – 1.54 (m, 8H), 1.26 (t, J = 7.1 Hz, 4H). 13C NMR (101 MHz, CDCl3) δ 168.48 (s), 155.92 – 154.52 (m), 148.15 (s), 113.23 (s), 61.95 (s), 59.13 – 58.36 (m), 54.83 (s), 36.17 (s), 30.98 (s), 25.84 (s), 25.30 (s), 14.21 (s).

7-chloro-1-(cyclohexylmethyl)-1,4-dihydropyrido[3,4-b]pyrazin-3(2H)-one

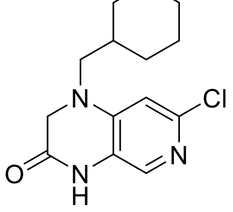

Ethyl *N*-(2-chloro-5-nitropyridin-4-yl)-*N*-cyclomethylhexylglycinate (2.2 g, 6.1 mmol) was dissolved in glacial acetic acid (30 mL). The mixture was heated to 70 °C and powdered iron (1.7 g, 30.5 mmol) was added portion wise. The temperature raised to 110 °C and was kept stirring for 1 h. The solids were filtered, the filtrate was extracted 3x with EtOAc, The combined organic layers were washed with brine. After drying over sodium sulfate, it was evaporated and purified using Flash chromatography yielding a brown solid (1.21 g, 79 %). ESI-MS: *m/z* 278.4 $[M-H]^-$ (278.1 calcd.); 1H NMR (400 MHz, DMSO) δ 10.66 (s, 1H), 7.52 (s, 1H), 6.67 (s, 1H), 3.95 (s, 2H), 3.11 (d, J = 7.3 Hz, 2H), 1.75 – 1.57 (m, 6H), 1.25 – 1.07 (m, 3H), 1.03 – 0.90 (m, 2H).

7-chloro-1-(cyclohexylmethyl)-4-methyl-1,4-dihydropyrido[3,4-b]pyrazin-3(2H)-one

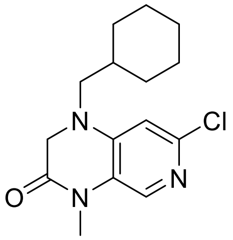

7-chloro-1-(cyclohexylmethyl)-1,4-dihydropyrido[3,4-b]pyrazin-3(2H)-one (1.2 g, 4.3 mmol) and K_2CO_3 (770 mg, 6.86 mmol) were added to DMF (12 mL) at rt. Subsequently, methyl iodide (0.4 mL, 6.4 mmol) was added while stirring. After 16 h, HPLC confirmed a conversion into a new product and water was added to the reaction. A precipitate formed that was suction filtered and dried, yielding a brown solid that was used without further purification (899 mg, 71 %).

7-((4-(benzyloxy)-3,5-difluorophenyl)amino)-1-(cyclohexylmethyl)-4-methyl-1,4-dihydropyrido[3,4-b]pyrazin-3(2H)-one

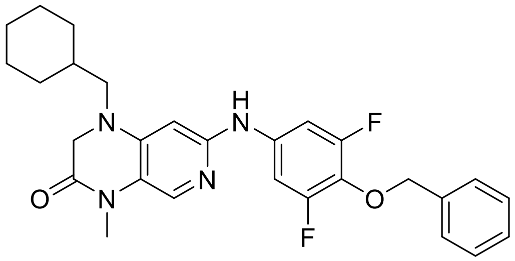

7-chloro-1-(cyclohexylmethyl)-4-methyl-1,4-dihydropyrido[3,4-b]pyrazin-3(2H)-one (200 mg, 0.68 mmol), 4-(benzyloxy)-3,5-difluoroaniline (320 mg, 1.36 mmol), Xantphos Pd G3 (13 mg, 0.014 mmol) and K_2CO_3 (188 mg, 1.36 mmol) were added to a dry flask with a stirrer in an argon atmosphere. Anhydrous 1,4-dioxane and tBuOH (3:1, 15 mL) were added. The mixture was refluxed for 16 h in an argon atmosphere. After full conversion, the reaction mixture was filtered over celite, evaporated and purified using Flash chromatography yielding transparent yellow crystals that were used without further purification (98 mg, 29 %).

1-(cyclohexylmethyl)-7-((3,5-difluoro-4-hydroxyphenyl)amino)-4-methyl-1,4-dihydropyrido[3,4-b]pyrazin-3(2H)-one (34)

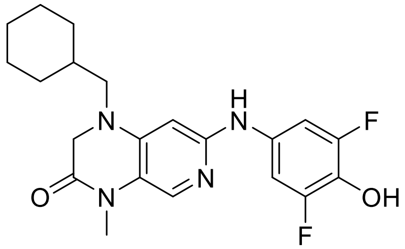

7-((4-(benzyloxy)-3,5-difluorophenyl)amino)-1-(cyclohexylmethyl)-4-methyl-1,4-dihydropyrido[3,4-b]pyrazin-3(2H)-one (98 mg, 0.20 mmol) was dissolved in EtOH (5 mL). Pd/C (10 mg) was added. The mixture was stirred for 24 h in a hydrogen atmosphere. After full

conversion, the reaction mixture was filtered over celite, evaporated and purified using Flash chromatography yielding a brown solid (30 mg, 38 %). HPLC-DAD: 254 nm: 97.4 %, 230 nm: 96.4%; ESI-HRMS: *m/z* [M+H]+: 403.19401 calcd.; 403.19432 found; 1H NMR (400 MHz, DMSO) δ 9.26 (s, 1H), 8.72 (s, 1H), 7.68 (s, 1H), 7.40 – 7.30 (m, 2H), 6.03 (s, 1H), 3.95 (s, 2H), 3.25 (s, 3H), 3.02 (d, J = 6.9 Hz, 2H), 1.79 – 1.59 (m), 1.29 – 1.09 (m), 1.04 – 0.89 (m). 13C NMR (101 MHz, DMSO) δ 162.3, 152.6, 152.40 (dd, J = 237.6, 8.9 Hz), 143.3, 134.5, 131.5, 125.8, 119.6, 100.61 (dd, J = 17.8, 9.0 Hz), 90.6, 54.9, 52.4, 34.8, 30.5, 27.7, 26.0, 25.3. IR (ATR) [cm-1]: 2920, 2849, 1653, 1605, 1506, 1436, 1205, 1008, 802.

Synthesis of 1-cycloheptyl-7-((3,5-difluoro-4-hydroxyphenyl)amino)-4-methyl-1,4-dihydropyrido[3,4-b]pyrazin-3(2H)-one (35)

Ethyl cycloheptylglycinate

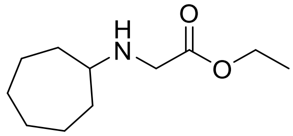

A flask with a stirrer bar was charged with cycloheptylamine (20.3 g, 180 mmol), TEA (7.3 g, 72 mmol) and toluene (200 mL). The solution was cooled in an ice bath. Ethyl bromoacetate (10 g, 60 mmol) was added dropwise to the stirred solution over 1 h and the resulting mixture was stirred overnight at room temperature. The mixture was washed 3x with 25 % ammonia in water solution and brine, dried over sodium sulfate and the remaining amine was co-evaporated with toluene giving the titled product as a colorless oil (11.2 g, 93 %). ESI-MS: *m/z* 222.3 $[M+Na]^+$ (222.1 calcd.); 1H-NMR (400 MHz, CDCl3): δ = 4.18 (q, J = 7.2 Hz, 2H), 3.39 (s, 2H), 2.60 (sept, J = 4.1 Hz, 1H), 1.88 – 1.32 (m), 1.27 (t, J = 7.1 Hz, 3H) ppm. 13C NMR (101 MHz, CDCl3) δ 172.6, 60.5, 58.5, 48.7, 34.6, 28.2, 24.0, 14.1.

Ethyl *N*-(2-chloro-5-nitropyridin-4-yl)-*N*-cycloheptylglycinate

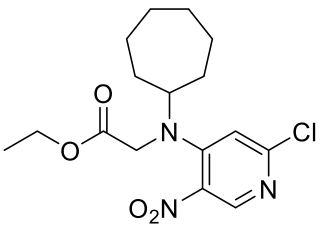

2,4-dichloro-5-nitropyridine (3.0 g, 15.5 mmol) and ethyl cycloheptylglycinate (3.1 g, 15.5 mmol) were dissolved in MeCN (150 mL) while stirring. TEA (4.3 mL, 31.1 mmol) was

slowly added. The reaction was stirred overnight at RT. The solvent was evaporated, the remains were taken up with EtOAc, washed with water and brine, dried over sodium sulfate, evaporated und purified using Flash chromatography (3.47 g, 63 %). ESI-MS: *m/z* 378.1 $[M+Na]^+$ (378.1 calcd.); 1H-NMR (400 MHz, CDCl3): δ = 8.50 (s, 1H), 6.81 (s, 1H), 4.14 (q, J = 7.1 Hz, 2H), 4.00 (s, 2H), 3.49 (tt, J = 10.1, 3.5 Hz , 1H), 2.07 – 1.42 (m), 1.23 (t, J = 7.1 Hz, 3H) ppm.

7-chloro-1-cycloheptyl-1,4-dihydropyrido[3,4-b]pyrazin-3(2H)-one

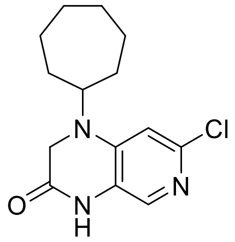

Ethyl *N*-(2-chloro-5-nitropyridin-4-yl)-*N*-cycloheptylglycinate (4.0 g, 11.2 mmol) was dissolved in glacial acetic acid (60 mL). The mixture was heated to 70 °C and powdered iron (3.14 g, 56.2 mmol) was added portionwise. The temperature raised to 110 °C and was kept stirring for 1 h. The solids were filtered, the filtrate was extracted 3x with EtOAc, The combined organic layers were washed with brine. After drying over sodium sulfate, it was evaporated and purified using Flash chromatography yielding a brown solid (1.41 g, 45 %). ESI-MS: *m/z* 278.2 $[M-H]^-$ (278.2 calcd.); 1H NMR (400 MHz, CDCl3) δ 9.42 (s, 1H), 7.72 (s, 1H), 6.51 (s, 1H), 3.92 (s, 2H), 3.72 – 3.63 (m, 1H), 1.93 – 1.49 (m, 12H). 13C NMR (101 MHz, CDCl3) δ 165.2, 146.9, 142.5, 134.2, 122.3, 105.2, 58.0, 45.3, 30.9, 27.6, 25.2.

7-chloro-1-cycloheptyl-4-methyl-1,4-dihydropyrido[3,4-b]pyrazin-3(2H)-one

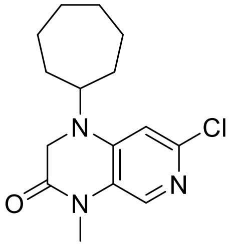

7-chloro-1-cycloheptyl-1,4-dihydropyrido[3,4-b]pyrazin-3(2H)-one (1.32 g, 4.7 mmol) and K_2CO_3 (848 mg, 7.56 mmol) were added to DMF (14 mL) at rt. Subsequently, methyl iodide (1.0 g, 7.1 mmol) was added while stirring. After 16 h, HPLC confirmed full conversion and water was added to the reaction. A precipitate formed that was suction filtered and dried, yielding a brown solid. 1H NMR (400 MHz, CDCl3) δ 7.76 (s, 1H), 6.53 (s, 1H), 3.90 (s, 2H), 3.70 – 3.61 (m, 1H), 3.35 (s, 3H), 1.89 – 1.52 (m, 12H). 13C NMR (101 MHz, CDCl3) δ 163.8, 147.1, 144.0, 133.6, 125.5, 105.0, 57.9, 45.5, 30.9, 28.6, 27.6, 25.2.

7-((4-(benzyloxy)-3,5-difluorophenyl)amino)-1-cycloheptyl-4-methyl-1,4-dihydropyrido[3,4-b]pyrazin-3(2H)-one

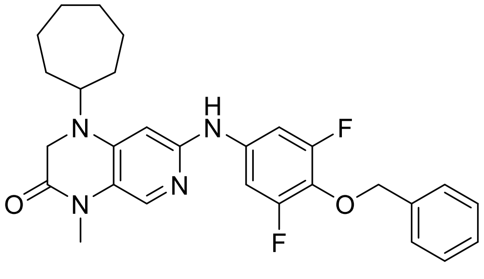

7-chloro-1-cycloheptyl-4-methyl-1,4-dihydropyrido[3,4-b]pyrazin-3(2H)-one (250 mg, 0.85 mmol), 4-(benzyloxy)-3,5-difluoroaniline (400 mg, 1.7 mmol) Brettphos Pd G3 (15 mg, 0.017 mmol) and KOtBu (191 mg, 1.7 mmol) were added to a dry flask with a stirrer in an argon atmosphere. Anhydrous 1,4-dioxane (14 mL) was added. The mixture was heated to 80 °C for 4

h in an argon atmosphere. After full conversion, the reaction mixture was filtered over celite, evaporated and purified using Flash chromatography yielding a brown solid (253 mg, 60 %) that was used in the next step without further purification. ESI-MS: *m/z* 493.5 $[M+H]^+$ (493.2 calcd.).

1-cycloheptyl-7-((3,5-difluoro-4-hydroxyphenyl)amino)-4-methyl-1,4-dihydropyrido[3,4-b]pyrazin-3(2H)-one (35)

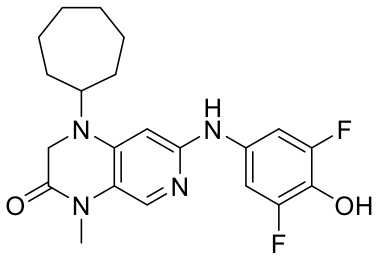

7-((4-(benzyloxy)-3,5-difluorophenyl)amino)-1-cycloheptyl-4-methyl-1,4-dihydropyrido[3,4-b]pyrazin-3(2H)-one (253 mg, 0.51 mmol) was dissolved in EtOH (5 mL) and conc. HCl (0.2 mL). Pd/C (21 mg) was added. The mixture was stirred for 24 h in a hydrogen atmosphere. After full conversion, the reaction mixture was filtered over celite, evaporated and purified using Flash chromatography yielding the titled product as a beige solid (78 mg, 38 %). HPLC-DAD: 254 nm: 100 %, 230 nm: 99.3 %; ESI-HRMS: *m/z* [M+H]+: 403.19401 calcd.; 403.19419 found; 1H NMR (400 MHz, DMSO) δ 9.27 (s, 1H), 8.75 (s, 1H), 7.72 (s, 1H), 7.41 – 7.31 (m, 2H), 6.11 (s, 1H), 3.77 (s, 2H), 3.65 – 3.56 (m, 1H), 3.24 (s, 3H), 1.83 – 1.39 (m). 13C NMR (101 MHz, DMSO) δ 163.4, 152.40 (dd, J = 237.8, 9.2 Hz), 152.7, 143.3, 134.48 (t, J = 12.8 Hz), 131.8, 125.87 (t, J = 16.6 Hz), 120.5, 100.63 (dd, J = 17.9, 9.3 Hz), 91.6, 56.6, 45.4, 29.9, 27.8, 27.1, 24.9. IR (ATR) [cm-1]: 2920, 2852, 1653, 1646, 1616, 1605, 1503, 1222, 1013.

Synthesis of methyl 4-((1-cyclohexyl-4-methyl-3-oxo-1,2,3,4-tetrahydropyrido[3,4-b]pyrazin-7-yl)amino)-2,6-difluorobenzoate (36)

Ethyl *N*-(2-chloro-5-nitropyridin-4-yl)-N-cyclohexylglycinate

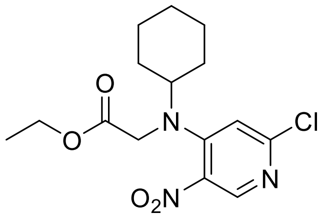

Ethyl cyclohexylglycinate (3.84 g, 20.7 mmol) was dissolved in water (38 mL) and 2,4-dichloro-5-nitropyrimidine (4.4 g, 22.8 mmol) in diethyl ether (65 mL) was added. The reaction mixture was cooled down to -10 °C and K_2CO_3 (8.8 g, 41.5 mmol) was added portion wise. The mixture was stirred vigorously for 1 h at -5 °C, slowly warming up to room temperature. After 16 h, the water phase was separated, and the organic phase was washed with water three times, dried over sodium sulfate and evaporated to dryness yielding a yellow oil (6.1 g, 86 %). 1H NMR (400 MHz, CDCl3) δ 8.50 (s, 1H), 6.86 (s, 1H), 4.15 (q, J = 7.2 Hz, 2H), 4.00 (s, 2H), 3.40 – 3.31 (m, 1H), 1.99 –1.2 (m).

7-chloro-1-cyclohexyl-1,4-dihydropyrido[3,4-b]pyrazin-3(2H)-one

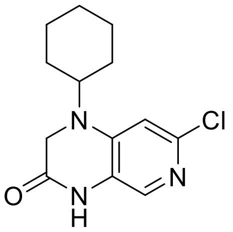

To a solution of ethyl *N*-(2-chloro-5-nitropyridin-4-yl)-N-cyclohexylglycinate (6.0 g, 17.6 mmol) in EtOH (370 mL) was added powdered iron (5.9 g, 105 mmol). The mixture was heated to reflux, followed by addition of 1 M aqueous HCl solution (14 ml). The mixture was refluxed for 2 h, then cooled to rt, the insoluble material was filtered. The solvent was evaporated to give a crude residue, that was purified using Flash chromatography to give the titled product as a brown solid (1.4 g, 30 %). 1H NMR (400 MHz, DMSO) δ 10.68 (s, 1H), 7.55 (s, 1H), 6.75 (s, 1H), 3.83 (s, 2H), 3.68 (s, 1H), 1.75 (m, 2H), 1.62 (m, 3H), 1.55 – 1.40 (m, 4H), 1.12 (m, 1H).

7-chloro-1-cyclohexyl-4-methyl-1,4-dihydropyrido[3,4-b]pyrazin-3(2H)-one

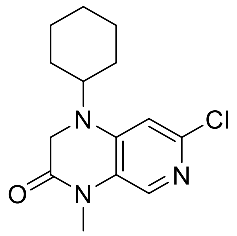

7-chloro-1-cyclohexyl-1,4-dihydropyrido[3,4-b]pyrazin-3(2H)-one (1.4 g, 5.2 mmol) and Cs_2CO_3 (3.42 g, 10.5 mmol) were added to DMF (14.5 mL) and cooled to -10 °C. Subsequently, methyl iodide (1.49 g, 10.5 mmol) was added while stirring and warmed to room temperature. After 24 h, HPLC confirmed a conversion into a new product and the reaction was quenched with water and aq. ammonia (25 %). A precipitate formed that was suction filtered and dried, yielding a brown solid (1.08 g, 74 %). 1H NMR (400 MHz, DMSO) δ 7.81 (s, 1H), 6.82 (s, 1H), 3.91 (s, 2H), 3.77 – 3.63 (m, 1H), 3.25 (s, 3H), 1.88 – 1.29 (m,), 1.25 – 1.02 (m,). 13C NMR (101 MHz, DMSO) δ 163.3, 145.8, 143.6, 133.7, 125.2, 104.0, 54.7, 44.8, 27.9, 27.5, 25.0, 24.8.

Methyl 4-((1-cyclohexyl-4-methyl-3-oxo-1,2,3,4-tetrahydropyrido[3,4-b]pyrazin-7-yl)amino)-2,6-difluorobenzoate

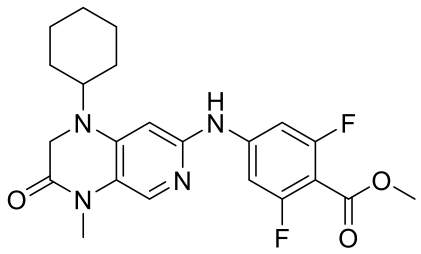

7-chloro-1-cyclohexyl-4-methyl-1,4-dihydropyrido[3,4-b]pyrazin-3(2H)-one (135 mg, 0.48 mmol), methyl 4-amino-2,6-difluorobenzoate (**207**) (135 mg, 0.72 mmol), K_2CO_3 (108 mg, 0.97 mmol) and Brettphos Pd G3 (8.7 mg, 0.0097 mmol) were added to a dry flask. In an argon atmosphere, dry a mixture of 1,4-dioxane and tBuOH (3:1, 10 mL) was added. The reaction was refluxed overnight. The mixture was suction filtered over Celite, evaporated and purified using Flash chromatography. A white precipitate was afforded (109 mg, 52 %). ESI-MS: *m/z* 429.6 $[M-H]^-$ (429.2 calcd.).

Methyl 4-((1-cyclohexyl-4-methyl-3-oxo-1,2,3,4-tetrahydropyrido[3,4-b]pyrazin-7-yl)amino)-2,6-difluorobenzoate (36)

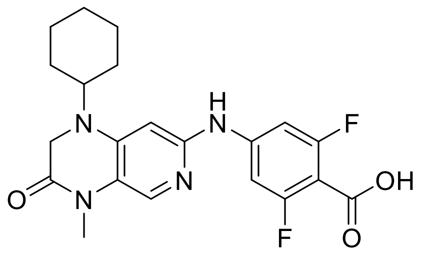

Methyl 4-((1-cyclohexyl-4-methyl-3-oxo-1,2,3,4-tetrahydropyrido[3,4-b]pyrazin-7-yl)amino)-2,6-difluorobenzoate (109 mg, 0.25 mmol), was dissolved in THF (5 mL). 1 M LiOH solution in water (0.5 mL, 0.5 mmol) was added dropwise and stirred for 1 h at rt. The solvent was evaporated and the pH of the remaining aqueous phase was adjusted to 2. A precipitate formed that was suction filtered giving the titled product as a white solid (63 mg, 60 %). HPLC-DAD: 254 nm: 98.1 %, 230 nm: 97.0 %; ESI-HRMS: *m/z* 417.1735 $[M+H]^+$ (417.1733 calcd.); 1H NMR (400 MHz, DMSO) δ 13.76 – 12.92 (m, 1H), 10.49 (s, 1H), 7.62 (s, 1H), 7.06 (d, J = 10.6 Hz, 2H), 6.67 (s, 1H), 4.14 (s, 2H), 3.78 – 3.69 (m, 1H), 3.26 (s, 3H), 1.84 – 1.71 (m), 1.69 – 1.50 (m), 1.38 (m), 1.14 (m). 13C NMR (101 MHz, DMSO) δ 162.0, 161.8, 161.04 (dd, J = 251.2, 9.3 Hz), 146.1, 145.9, 144.55 (t, J = 14.1 Hz), 122.30 – 122.05 (m), 122.0, 101.7, 101.4, 92.5, 67.0, 56.5, 45.2, 28.0, 27.5, 24.9. IR (ATR) [cm-1]: 3203, 2924, 2852, 2492, 1581, 1550, 1521, 1222, 1044.

Synthesis of 1-cyclohexyl-4-methyl-7-((2,3,5,6-tetrafluoro-4-hydroxyphenyl)amino)-1,4-dihydropyrido[3,4-b]pyrazin-3(2H)-one (37)

7-((4-(benzyloxy)-2,3,5,6-tetrafluorophenyl)amino)-1-cyclohexyl-4-methyl-1,4-dihydropyrido[3,4-b]pyrazin-3(2H)-one

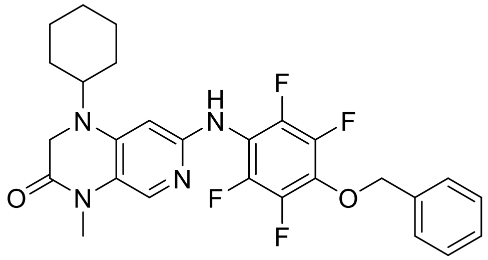

7-chloro-1-cyclohexyl-4-methyl-1,4-dihydropyrido[3,4-b]pyrazin-3(2H)-one (400 mg, 1.43 mmol), 4-(benzyloxy)-2,3,5,6-tetrafluoroaniline (427 mg, 1.57 mmol), Cs_2CO_3 (932 mg, 2.86 mmol) and Xantphos Pd G3 (26 mg, 0.029 mmol) was added to a dry flask with a reflux condenser in an argon atmosphere. An anhydrous 1,4-dioxane/tBuOH mixture (3:1, 30 mL) was added. The mixture was refluxed for 3 h. The reaction was cooled to room temperature filtered over Celite, diluted with water, extracted with EtOAc, washed with brine and dried over sodium sulfate, evaporated and purified using Flash chromatography yielding a dark brown solid (206 mg, 28 %). ESI-MS: *m/z* 515.2 $[M+H]^+$ (515.2 calcd.); 1H NMR (400 MHz, CDCl3) δ 7.64 (s, 1H), 7.48 – 7.43 (m, 2H), 7.42 – 7.35 (m, 3H), 5.89 (s, 1H), 5.22 (s, 2H), 3.86 (s, 2H), 3.33 (s, 3H), 1.95 – 1.69 (m, 7H), 1.53 – 1.39 (m, 3H), 1.35 – 1.28 (m, 3H), 1.21 – 1.12 (m, 2H), 0.93 – 0.79 (m, 5H).

1-cyclohexyl-4-methyl-7-((2,3,5,6-tetrafluoro-4-hydroxyphenyl)amino)-1,4-dihydropyrido[3,4-b]pyrazin-3(2H)-one (37)

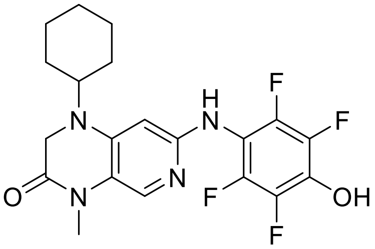

7-((4-(benzyloxy)-2,3,5,6-tetrafluorophenyl)amino)-1-cyclohexyl-4-methyl-1,4-dihydropyrido[3,4-b]pyrazin-3(2H)-one (222 mg, 0.43 mmol) was dissolved in EtOH (10 mL) and conc. HCl (0.25 mL). Pd/C (22 mg) was added. The mixture was stirred for 24 h in a hydrogen atmosphere. After full conversion, the reaction mixture was filtered over celite, evaporated and purified using Flash chromatography yielding gray crystals (63 mg, 34 %). HPLC-DAD: 254 nm: 97.6 %, 230 nm: 94.6 %; ESI-HRMS: *m/z* [M+H]+: 425.15952 calcd.; 425.16008 found; 1H NMR (400 MHz, DMSO) δ 12.74 (s, 1H), 11.72 (s, 1H), 9.79 (s, 1H), 7.31 (s, 1H), 6.37 (s, 1H), 4.13 (s, 2H), 3.72 – 3.64 (m, 1H), 3.21 (s, 3H), 1.86 – 1.70 (m, 4H), 1.67 – 1.50 (m, 3H), 1.43 – 1.28 (m, 2H), 1.23 – 1.09 (m, 1H). IR (ATR) [cm-1]: 2925, 1682, 1606, 1507, 1362, 1228, 977, 818.

Synthesis of 5-((1-cyclohexyl-4-methyl-3-oxo-1,2,3,4-tetrahydropyrido[3,4-b]pyrazin-7-yl)amino)-3-fluoro-2-hydroxybenzonitrile (38)

2,3-difluoro-5-nitrobenzonitrile

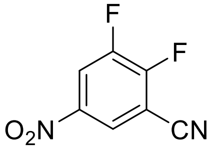

H_2SO_4 (6.1 mL) was added to a dry flask with a magnetic stirrer and cooled to 0 °C. Fuming nitric acid (5.1 mL) was slowly added and stirred. The nitrating acid was added dropwise to a flask containing 2,3-difluorobenzonitrile (5.0 g, 35.9 mmol) while keeping the temperature below 5 °C. After conversion, the mixture was poured over ice, extracted 3x with EtOAc. The combined organic layers were dried over brine, and sodium sulfate, evaporated and purified using Flash chromatography yielding the desired product as a yellow oil that slowly crystallized (3.0 g, 45 %). 1H NMR (400 MHz, CDCl3) δ 8.41 – 8.34 (m, 1H).

2-(benzyloxy)-3-fluoro-5-nitrobenzonitrile

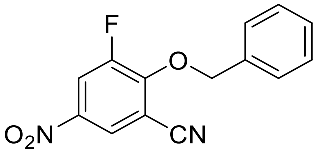

K_2CO_3 (1.5 g, 10.9 mmol) was added to a mixture of 2,3-difluoro-5-nitrobenzonitrile (1.0 g, 5.4 mmol) and benzyl alcohol (0.91 g, 8.4 mmol) in DMF (6.4 mL). The resultant mixture was stirred at rt overnight. The reaction mixture was concentrated under reduced pressure and the residue was partitioned between ethyl acetate and water. The phases were separated and the

aqueous phase was extracted 2x with EtOAc. The combined organic layers were washed with brine, dried over sodium sulfate, evaporated and purified using Flash chromatography giving a yellow oil (0.8 g, 96 %). 1H NMR (400 MHz, CDCl3) δ 8.25 (dd, J = 2.6, 1.5 Hz, 1H), 8.17 (dd, J = 11.4, 2.7 Hz, 1H), 7.48 – 7.36 (m, 5H), 5.55 (d, J = 2.1 Hz, 2H).

5-amino-2-(benzyloxy)-3-fluorobenzonitrile

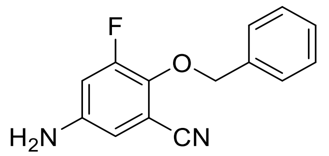

2-(benzyloxy)-3-fluoro-5-nitrobenzonitrile (0.93 g, 3.4 mmol) was dissolved in a dioxane/water mixture (3:1) (20 mL). Ammonium acetate (0.94 g, 6.8 mmol) and zinc (1.56 g, 23.9 mmol) were added and heated to 80 °C. The hot mixture was filtered over celite and extracted 3x with DCM. The combined organic layers were dried over sodium sulfate and evaporated giving the desired product as yellow crystals (0.798 g, 96 %). 1H NMR (400 MHz, DMSO) δ 7.46 – 7.32 (m, 5H), 6.73 (dd, J = 13.7, 2.6 Hz, 1H), 6.59 (dd, J = 2.6, 1.2 Hz, 1H), 5.60 (s, 2H), 5.04 (s, 2H).

N-(4-(benzyloxy)-3,5-difluorophenyl)-1-cyclohexyl-1H-imidazo[4,5-c]pyridin-6-amine

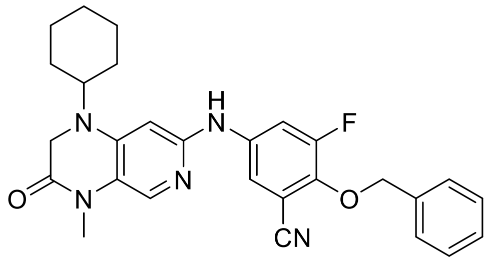

7-chloro-1-cyclohexyl-4-methyl-1,4-dihydropyrido[3,4-b]pyrazin-3(2H)-one (150 mg, 0.64 mmol), 5-amino-2-(benzyloxy)-3-fluorobenzonitrile (162 mg, 0.67 mmol), Xantphos Pd G3

(12 mg, 0.013 mmol) and Cs_2CO_3 (415 mg, 1.3 mmol) were added to a dry flask with a stirrer in an argon atmosphere. 1,4-dioxane and tBuOH (4:1) were added. The mixture was refluxed for 4 h in an argon atmosphere. After full conversion, the reaction mixture was filtered over celite, evaporated giving a crude orange oil that was used without further purification (210 mg, 76 %).

5-((1-cyclohexyl-4-methyl-3-oxo-1,2,3,4-tetrahydropyrido[3,4-b]pyrazin-7-yl)amino)-3-fluoro-2-hydroxybenzonitrile (38)

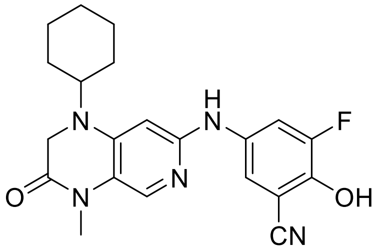

N-(4-(benzyloxy)-3,5-difluorophenyl)-1-cyclohexyl-1H-imidazo[4,5-c]pyridin-6-amine (200 mg, 0.4 mmol) was dissolved in EtOH (11 mL). The mixture was purged with nitrogen and Pd/C (20 mg) was added. The mixture was purged with hydrogen and concentrated hydrochloric acid (0.1 mL) was added. The reaction was stirred for 4 h at room temperature. After completion, the reaction was filtered over celite, evaporated and purified using Flash chromatography. The combined fractions were taken up in EtOAc and washed 3x with 2N NaOH solution. The combined aqueous phases were acidified and the resulting precipitate was suction filtered giving purely the desired product as a white powder (19 mg, 12 %). HPLC-DAD: 254 nm: 99.6 %, 230 nm: 100 %; ESI-HRMS: *m/z* [M+H]+ = 396.18303 calcd.; 396.18327 found; 1H NMR (400 MHz, DMSO) δ 12.71 (s, 1H), 11.47 (s, 1H), 10.05 (s, 1H), 7.56 (d, J = 12.1 Hz, 1H), 7.46 (s, 1H), 7.40 (s, 1H), 6.36 (s, 1H), 4.12 (s, 2H), 3.67 – 3.60 (m, 1H), 3.22 (s, 3H), 1.86 – 1.08 (m). 13C NMR (101 MHz, DMSO) δ 161.7, 152.7, 150.3, 148.4, 145.8, 122.4, 120.7, 102.4, 88.5,

56.6, 45.2, 28.0, 27.4, 24.9, 24.8. IR (ATR) [cm-1]: 3412, 2933, 2862, 1669, 1599, 1538, 1494, 1219, 1153, 825.

Synthesis of 5-((1-cyclopropyl-4-methyl-3-oxo-1,2,3,4-tetrahydropyrido[3,4-b]pyrazin-7-yl)amino)-3-fluoro-2-hydroxybenzonitrile (39)

2-(benzyloxy)-5-((1-cyclopropyl-4-methyl-3-oxo-1,2,3,4-tetrahydropyrido[3,4-b]pyrazin-7-yl)amino)-3-fluorobenzonitrile

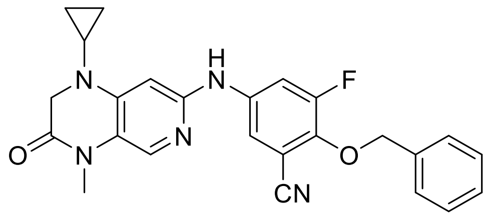

7-chloro-1-cyclopropyl-4-methyl-1,4-dihydropyrido[3,4-b]pyrazin-3(2H)-one (200 mg, 0.84 mmol), 5-amino-2-(benzyloxy)-3-fluorobenzonitrile (245 mg, 1 mmol), Brettphos Pd G3 (15.2 mg, 0.016 mmol) and Cs_2CO_3 (548 mg, 1.68 mmol) were added to a dry flask with a stirrer in an argon atmosphere. Anhydrous 1,4-dioxane (11 mL) was added. The mixture was refluxed for 4 h in an argon atmosphere. After full conversion, the reaction mixture was filtered over celite, evaporated and purified using Flash chromatography yielding a colorless solid that was used without further purification (170 mg, 46 %).

5-((1-cyclopropyl-4-methyl-3-oxo-1,2,3,4-tetrahydropyrido[3,4-b]pyrazin-7-yl)amino)-3-fluoro-2-hydroxybenzonitrile (39)

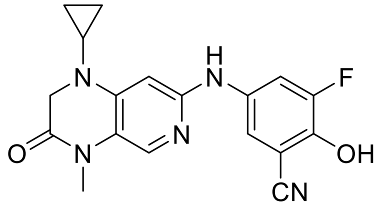

2-(benzyloxy)-5-((1-cyclopropyl-4-methyl-3-oxo-1,2,3,4-tetrahydropyrido[3,4-b]pyrazin-7-yl)amino)-3-fluorobenzonitrile (200 mg, 0.45 mmol) and Pd/C (20 mg) were dissolved in MeOH (11 mL). The mixture was purged with hydrogen for 16 h. Upon completion, the mixture was filtered over Celite, evaporated and purified using Flash chromatography with a gradient of 100 % DCM to 10% MeOH/90% DCM/0.2 M NH_3 giving a yellow solid (39 mg, 24 %). HPLC-DAD: 254 nm: 99.6 %, 230 nm: 99.0 %; ESI-HRMS: *m/z* [M+H]+: 354.13608 calcd.; 354.13651 found; 1H NMR (400 MHz, DMSO) δ 10.62 (s, 1H), 9.05 (s, 1H), 7.85 (dd, J = 13.7, 2.6 Hz, 1H), 7.77 (s, 1H), 7.75 (dd, J = 2.4, 1.3 Hz, 1H), 6.48 (s, 1H), 3.87 (s, 2H), 3.23 (s, 3H), 2.42 – 2.35 (m, 1H), 0.88 – 0.82 (m, 2H), 0.65 – 0.57 (m, 2H). 13C NMR (101 MHz, DMSO) δ 163.49, 152.29, 152.12, 149.92, 144.87, 140.77, 140.60, 135.58, 135.48, 131.45, 120.75, 116.48, 116.42, 115.49, 110.70, 110.47, 101.53, 101.46, 93.03, 51.76, 29.78, 27.80, 7.36. IR (ATR) [cm-1]: 2920, 2851, 2216, 1660, 1610, 1449, 1349, 1216, 1142, 1020, 820.

Synthesis of 5-((1-cyclopropyl-4-methyl-3-oxo-1,2,3,4-tetrahydropyrido[3,4-b]pyrazin-7-yl)amino)-2-hydroxybenzonitrile (40)

2-(benzyloxy)-5-nitrobenzonitrile

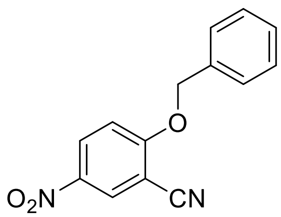

K_2CO_3 (3.44 g, 24.9 mmol) was added to a solution of 2-fluoro-5-nitrobenzonitrile (2.07 g, 12.5 mmol) and benzyl alcohol (3.2 g, 19.3 mmol) in *N*,*N*-dimethyl form amide (70 mL). The resultant mixture was stirred at RT for 3 h. After addition, a pink product precipitated that was

suction filtered, dried and used without further purification (3.19 g). 1H NMR (400 MHz, DMSO) δ 8.71 (d, J = 2.8 Hz, 1H), 8.53 (dd, J = 9.4, 2.8 Hz, 1H), 7.60 – 7.35 (m, 6H), 5.46 (s, 2H). 13C NMR (101 MHz, DMSO) δ 164.3, 140.7, 135.1, 130.5, 129.9, 128.6, 128.5, 127.7, 114.4, 114.1, 101.6, 71.4.

5-amino-2-(benzyloxy)benzonitrile

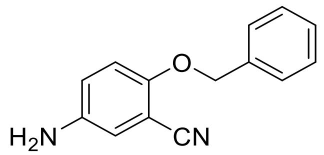

2-(benzyloxy)-5-nitrobenzonitrile (3.1 g, 12.2 mmol) was dissolved in a 1,4-dioxane/water mixture (3:1, 20 mL). Ammonium formate (3.1 g, 48.8 mmol) and Zn (5.58 g, 85.4 mmol) were added and heated to 80 °C for 1 h. The hot mixture was filtered over Celite and extracted 3x with DCM. The combined organic layers were dried over sodium sulfate and evaporated giving a yellow oil that slowly crystallized (2.67 g, 98 %). ESI-MS: *m/z* 247.3 $[M+Na]^+$ (247.2 calcd.); 1H NMR (400 MHz, DMSO) δ 7.47 – 7.30 (m, 6H), 7.05 (d, J = 8.7 Hz, 1H), 6.84 (dd, J = 8.9, 2.8 Hz, 1H), 6.80 (d, J = 2.7 Hz, 1H), 5.11 (s, 2H), 5.09 (s, 2H).13C NMR (101 MHz, DMSO) δ 151.0, 143.2, 136.7, 128.4, 127.9, 127.5, 120.3, 117.0, 116.7, 115.4, 101.1, 70.5.

2-(benzyloxy)-5-((1-cyclopropyl-4-methyl-3-oxo-1,2,3,4-tetrahydropyrido[3,4-b]pyrazin-7-yl)amino)benzonitrile

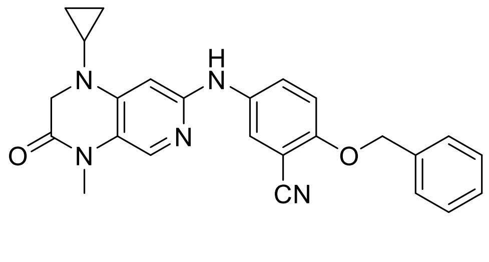

7-chloro-1-cyclopropyl-4-methyl-1,4-dihydropyrido[3,4-b]pyrazin-3(2H)-one (200 mg, 0.84 mmol), 5-amino-2-(benzyloxy)benzonitrile (283 mg, 1.26 mmol), Brettphos Pd G3 (15.3 mg, 0.016 mmol) and Cs_2CO_3 (548 mg, 1.7 mmol) were added to a dry flask with a stirrer in an argon atmosphere. 1,4-dioxane and tBuOH (4:1) were added. The mixture was refluxed for 4 h in an argon atmosphere. After full conversion, the reaction mixture was filtered over celite, evaporated and purified using Flash chromatography yielding transparent yellow crystals (134 mg, 37 %) that were used without further purification.

5-((1-cyclopropyl-4-methyl-3-oxo-1,2,3,4-tetrahydropyrido[3,4-b]pyrazin-7-yl)amino)-2-hydroxybenzonitrile (40)

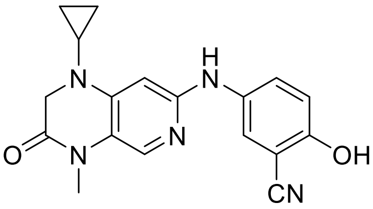

2-(benzyloxy)-5-((1-cyclopropyl-4-methyl-3-oxo-1,2,3,4-tetrahydropyrido[3,4-b]pyrazin-7-yl)amino)benzonitrile (85.0 mg, 0.20 mmol) and Pd/C (8.5 mg) were dissolved in MeOH (4.5 mL). The mixture was purged with hydrogen for 16 h. Upon completion, the mixture was filtered over celite, evaporated and purified using Flash chromatography with a gradient of 100 % DCM to 10 % MeOH/90 % DCM/0.2 M NH_3 giving the desired compound as a yellow solid (16 mg, 24 %). HPLC-DAD: 254 nm: 96.6 %, 230 nm: 97.9 %; ESI-HRMS: *m/z* [M+H]+: 336.14550 calcd.; 336.14573 found; 1H NMR (400 MHz, DMSO) δ 10.42 (s, 1H), 8.83 (s, 1H), 8.13 (d, J = 2.7 Hz, 1H), 7.74 (s, 1H), 7.51 (dd, J = 9.0, 2.7 Hz, 1H), 6.92 (d, J = 9.0 Hz, 1H), 6.49 (s, 1H), 3.85 (s, 2H), 3.22 (s, 3H), 2.40 – 2.33 (m, 1H), 0.87 – 0.81 (m, 2H), 0.64 – 0.56 (m, 2H). 13C NMR (101 MHz, DMSO) δ 163.5, 153.6, 152.6, 144.8, 134.8, 131.5, 124.9, 120.4,

120.2, 117.5, 116.6, 98.0, 92.7, 51.8, 29.8, 27.8, 7.4. IR (ATR) [cm-1]: 3360, 2923, 2851, 2212, 1621, 1429, 1328, 1273, 1216, 818, 685.

Synthesis of 1-cyclopropyl-7-((3-fluoro-4-hydroxy-5-(trifluoromethyl)phenyl)amino)-4-methyl-1,4-dihydropyrido[3,4-b]pyrazin-3(2H)-one (41)

1,2-difluoro-5-nitro-3-(trifluoromethyl)benzene

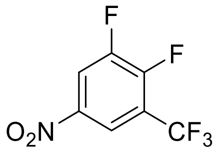

H_2SO_4 (4.5 mL) was added to a dry flask with a magnetic stirrer and cooled to 0 °C. Fuming nitric acid (3.9 mL) was slowly added and stirred. The nitrating acid was added dropwise to a flask containing 1,2-difluoro-3-(trifluoromethyl)benzene (5.0 g, 27.5 mmol) while keeping the temperature below 5 °C. After conversion, the mixture was poured over ice, extracted 3x with EtOAc. The combined organic layers were dried over brine, and sodium sulfate and evaporated yielding the desired product as a crude yellow oil (6.08 g, 98 %).

2-(benzyloxy)-1-fluoro-5-nitro-3-(trifluoromethyl)benzene

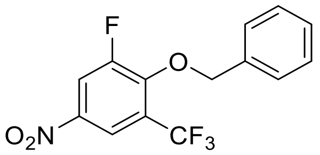

K_2CO_3 (6.7 g, 48.4 mmol) was added to a solution of 1,2-difluoro-5-nitro-3-(trifluoromethyl)benzene (5.5 g, 24.2 mmol) and benzyl alcohol (8.5 g, 37.5 mmol) in DMF (35 mL). The resultant mixture was stirred at rt overnight. The reaction mixture was concentrated

under reduced pressure and water was added giving a red precipitate, that was suction filtered (6.32 g, 83 %). 1H NMR (400 MHz, CDCl3) δ 8.34 – 8.30 (m, 1H), 8.20 (dd, J = 11.5, 2.7 Hz, 1H), 7.48 – 7.32 (m, 5H), 5.42 (d, J = 2.3 Hz, 2H).

4-(benzyloxy)-3-fluoro-5-(trifluoromethyl)aniline

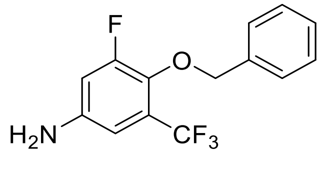

2-(benzyloxy)-1-fluoro-5-nitro-3-(trifluoromethyl)benzene (6.3 g, 0.020 mol) was dissolved in a 1,4-dioxane/water mixture (3:1, 40 mL). Ammonium acetate (6.16 g, 0.08 mol) and zinc (9.15 g, 0.14 mol) were added and heated to 80 °C for 1 h. The hot mixture was filtered over celite and extracted 3x with DCM. The combined organic layers were dried over sodium sulfate, evaporated, and purified using Flash chromatography giving a yellow oil (4.094 g, 72 %). 1H NMR (400 MHz, CDCl3) δ 7.51 – 7.47 (m, 2H), 7.41 – 7.34 (m, 3H), 6.67 – 6.65 (m, 1H), 6.61 (dd, J = 12.3, 2.7 Hz, 1H), 5.02 (s, 2H), 3.61 (bs, 2H).

7-((4-(benzyloxy)-3-fluoro-5-(trifluoromethyl)phenyl)amino)-1-cyclopropyl-4-methyl-1,4-dihydropyrido[3,4-b]pyrazin-3(2H)-one

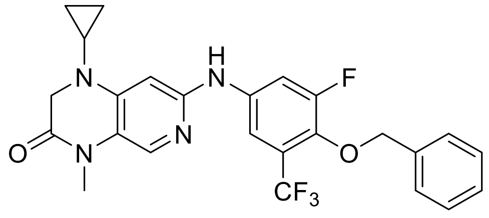

7-chloro-1-cyclopropyl-4-methyl-1,4-dihydropyrido[3,4-b]pyrazin-3(2H)-one (253 mg, 1.06 mmol), 4-(benzyloxy)-3-fluoro-5-(trifluoromethyl)aniline (364 mg, 1.28 mmol), Brettphos

Pd G3 (19.3 mg, 0.021 mmol) and Cs_2CO_3 (693 mg, 2.13 mmol) were added to a dry flask with a stirrer in an argon atmosphere. 1,4-dioxane and tBuOH (4:1, 19 mL) were added. The mixture was refluxed for 4 h in an argon atmosphere. After full conversion, the reaction mixture was filtered over celite, evaporated and purified using Flash chromatography yielding an orange material (314 mg, 61 %) that was used without further purification. ESI-MS: *m/z* 484.9 $[M-H]^-$ (485.2 calcd.).

1-cyclopropyl-7-((3-fluoro-4-hydroxy-5-(trifluoromethyl)phenyl)amino)-4-methyl-1,4-dihydropyrido[3,4-b]pyrazin-3(2H)-one (41)

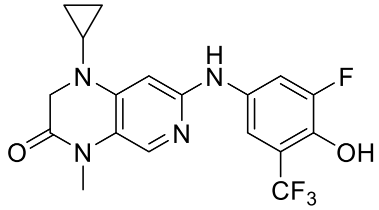

7-((4-(benzyloxy)-3-fluoro-5-(trifluoromethyl)phenyl)amino)-1-cyclopropyl-4-methyl-1,4-dihydropyrido[3,4-b]pyrazin-3(2H)-one (314 mg, 0.65 mmol) and Pd/C (31 mg) were dissolved in MeOH (18 mL). The mixture was purged with hydrogen gas. Upon completion, the mixture was filtered over celite, evaporated and purified using Flash chromatography giving the desired compound as a transparent solid (46 mg, 18 %). HPLC-DAD: 254 nm: 97.0 %, 230 nm: 97.4 %; ESI-HRMS: *m/z* [M+H]+: 397.12821 calcd.; 397.12894 found; 1H NMR (400 MHz, DMSO) δ 9.92 (s, 1H), 9.03 (s, 1H), 7.97 (dd, J = 13.6, 2.4 Hz, 1H), 7.75 (s, 1H), 7.61 (s, 1H), 6.49 (s, 1H), 3.87 (s, 2H), 3.23 (s, 3H), 2.42 – 2.35 (m, 1H), 0.84 (q, J = 6.7 Hz, 2H), 0.64 – 0.58 (m, 2H). 19F NMR (659 MHz, DMSO) δ -60.43 (s), -132.16 (d, J = 13.5 Hz). 13C NMR (101 MHz, DMSO) δ 163.50, 152.82, 152.31, 150.47, 144.85, 135.82, 135.65, 134.97, 134.87, 131.50, 120.66, 118.17,

110.05, 108.88, 108.64, 92.99, 51.79, 48.59, 29.79, 27.80, 7.34. IR (ATR) [cm-1]: 3353, 1661, 1617, 1500, 1356, 1229, 1117, 1019, 1005, 885.

Synthesis of methyl 5-((1-cyclopropyl-4-methyl-3-oxo-1,2,3,4-tetrahydropyrido[3,4-b]pyrazin-7-yl)amino)-3-fluoro-2-hydroxybenzoate (42)

Methyl 2,3-difluoro-5-nitrobenzoate

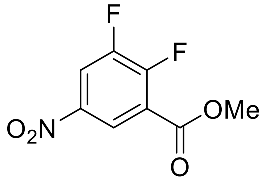

H_2SO_4 (3 mL) was added to a dry flask with a magnetic stirrer and cooled to 0 °C. Fuming nitric acid (2.5 mL) was slowly added and stirred. The nitrating acid was added dropwise to a flask containing methyl 2,3-difluorobenzoate (3 g, 17.4 mmol) while keeping the temperature below 5 °C. After conversion, the mixture was poured over ice, extracted 3x with EtOAc. The combined organic layers were dried over brine, and sodium sulfate, evaporated and purified using Flash chromatography. Two fractions were isolated. The desired product, slowly crystallized at room temperature giving yellow crystals (1.17 g, 5.4 mmol). 1H NMR (400 MHz, CDCl3) δ 3.92 – 3.87 (m, 1H), 3.53 – 3.47 (m, 1H), -0.75 (s, 3H).

Methyl 2-(benzyloxy)-3-fluoro-5-nitrobenzoate

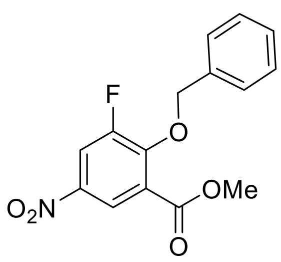

K_2CO_3 (1.49. g, 10.8 mmol) was added to a solution of Methyl 2,3-difluoro-5-nitrobenzoate (1.17 g, 5.4 mmol) and benzyl alcohol (1.8 g, 8.4 mmol) in DMF (7.5 mL). The resultant mixture was stirred at rt overnight. The reaction was concentrated under reduced pressure and the residue was partitioned between ethyl acetate and water. The phases were separated and the aqueous phase was extracted 2x with EtOAc. The combined organic layers were washed with brine, dried over sodium sulfate, evaporated and purified using Flash chromatography giving a red oil (1.44 g, 87 %) that was used in the next step without further purification.

Methyl 5-amino-2-(benzyloxy)-3-fluorobenzoate

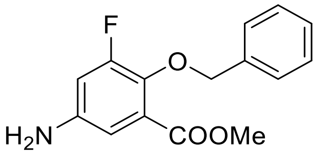

Methyl 2-(benzyloxy)-3-fluoro-5-nitrobenzoate (1.4 g, 4.6 mmol) was dissolved in a dioxane/water mixture (3:1, 20 mL). Ammonium formate (0.58 g, 9.2 mmol) and zinc (2.1 g, 32 mmol) were added and heated to 80 °C for 1 h. The hot mixture was filtered over celite and extracted 3x with DCM. The combined organic layers were dried over sodium sulfate and evaporated giving a yellow oil (1.09 g, 86 %) that was used without further purification. ESI-MS: *m/z* 298.3 $[M+Na]^+$ (298.2 calcd.).

Methyl 2-(benzyloxy)-5-((1-cyclopropyl-4-methyl-3-oxo-1,2,3,4-tetrahydropyrido[3,4-b]pyrazin-7-yl)amino)-3-fluorobenzoate

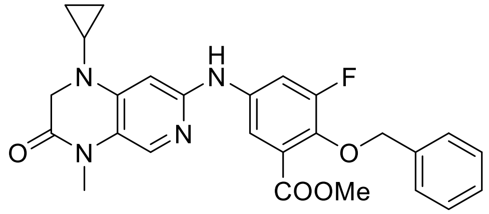

7-chloro-1-cyclopropyl-4-methyl-1,4-dihydropyrido[3,4-b]pyrazin-3(2H)-one (210 mg, 0.88 mmol), methyl 5-amino-2-(benzyloxy)-3-fluorobenzoate (364 mg, 1.33 mmol), Brettphos Pd G3 (16 mg, 0.018 mmol) and Cs_2CO_3 (575 mg, 1.77 mmol) were added to a dry flask with a stirrer in an argon atmosphere. 1,4-dioxane and tBuOH (4:1, 15 ml) were added. The mixture was refluxed for 4 h in an argon atmosphere. After full conversion, the reaction mixture was filtered over celite, evaporated and purified using Flash chromatography yielding a yellow solid material (134 mg, 32 %). ESI-MS: *m/z* 477.8 $[M+H]^+$ (477.5 calcd.).

Methyl 5-((1-cyclopropyl-4-methyl-3-oxo-1,2,3,4-tetrahydropyrido[3,4-b]pyrazin-7-yl)amino)-3-fluoro-2-hydroxybenzoate (42)

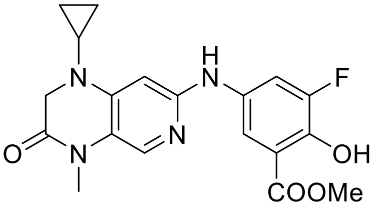

Methyl-2-(benzyloxy)-5-((1-cyclopropyl-4-methyl-3-oxo-1,2,3,4-tetrahydropyrido[3,4-b]pyrazin-7-yl)amino)-3-fluorobenzoate (134 mg, 0.28 mmol) and Pd/C 5% (13.4 mg) were dissolved in MeOH (7.5 mL). A drop of concentrated hydrochloric acid was added. The mixture was purged with hydrogen for 16 h. Upon completion, the mixture was filtered over celite, evaporated and purified using Flash chromatography with a gradient of 100 % DCM to 10 % MeOH/90 % DCM giving the desired compound as a white solid (43 mg, 40 %). HPLC-DAD: 254 nm: 97.0 %, 230 nm: 96.3 %; ESI-HRMS: *m/z* [M+H]+: 387.14631 calcd.; 387.14655

found; 1H NMR (400 MHz, DMSO) δ 9.91 (s, 1H), 8.99 (s, 1H), 8.02 (dd, J = 13.8, 2.6 Hz, 1H), 7.80 (dd, J = 2.4, 1.6 Hz, 1H), 7.74 (s, 1H), 6.50 (s, 1H), 3.91 (s, 3H), 3.87 (s, 2H), 3.23 (s, 3H), 2.42 – 2.34 (m, 1H), 0.88 – 0.82 (m, 2H), 0.65 – 0.59 (m, 2H). 13C NMR (101 MHz, DMSO) δ 168.4, 168.4, 163.4, 152.3, 151.7, 149.3, 144.9, 141.0, 140.9, 134.4, 134.3, 131.3, 120.6, 115.2, 115.2, 112.9, 112.8, 111.5, 111.3, 92.8, 52.6, 51.8, 29.8, 27.8, 7.3. IR (ATR) [cm-1]: 3224, 3071, 1671, 1602, 1476, 1437, 1363, 1228, 990, 786.

Synthesis of 1-cyclopropyl-7-((3,5-dichloro-4-hydroxyphenyl)amino)-4-methyl-1,4-dihydropyrido[3,4-b]pyrazin-3(2H)-one (43)

4-((tert-butyldimethylsilyl)oxy)-3,5-dichloroaniline

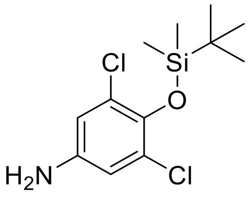

4-amino-2,6-dichlorophenol (1 g, 5.6 mmol) was dissolved in anhydrous DMF (5 mL). The solution was charged with imidazole (0.766 g, 11.2 mmol) and *tert*-butylchlorodimethylsilane (1.27 g, 8.4 mmol) and stirred for 1 h. Upon completion, water was added and the mixture was extracted 3x with DCM. The combined organic layers were dried over sodium sulfate, evaporated and purified using Flash chromatography giving a brown-transparent oil (1.47 g, 90 %). ESI-MS: *m/z* 291.8 $[M+H]^+$ (292.1 calcd.); 1H NMR (400 MHz, CDCl3) δ 6.61 (s, 2H), 1.04 (s, 9H), 0.25 (s, 6H). 13C NMR (101 MHz, CDCl3) δ 140.94, 140.80, 127.07, 115.68, 26.20, 18.97, -3.15.

7-((4-((tert-butyldimethylsilyl)oxy)-3,5-dichlorophenyl)amino)-1-cyclopropyl-4-methyl-1,4-dihydropyrido[3,4-b]pyrazin-3(2H)-one

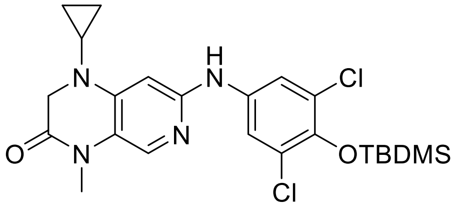

7-chloro-1-cyclopropyl-4-methyl-1,4-dihydropyrido[3,4-b]pyrazin-3(2H)-one (300 mg, 1.3 mmol), 4-((tert-butyldimethylsilyl)oxy)-3,5-dichloroaniline (443 mg, 1.51 mmol), Xphos Pd G4 (21.7 mg, 0.024 mmol) and KOtBu (283 mg, 2.39 mmol) were added to a dry flask with a stirrer in an argon atmosphere. Anhydrous toluene (14 mL) was added. The mixture was refluxed for 4 h in an argon atmosphere. After full conversion, the reaction mixture was filtered over celite, evaporated and purified using Flash chromatography yielding a brown oily material (314 mg, 64 %). ESI-MS: *m/z* 493.0 $[M+H]^+$ (493.2 calcd.).

1-cyclopropyl-7-((3,5-dichloro-4-hydroxyphenyl)amino)-4-methyl-1,4-dihydropyrido[3,4-b]pyrazin-3(2H)-one (43)

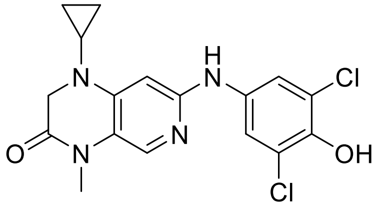

7-((4-((tert-butyldimethylsilyl)oxy)-3,5-dichlorophenyl)amino)-1-cyclopropyl-4-methyl-1,4-dihydropyrido[3,4-b]pyrazin-3(2H)-one (305 mg, 0.62 mmol) was dissolved in THF. 1.0 M tetrabutylammonium fluoride in THF (3.1 mL, 3.1 mmol) was added and stirred for 2 h at rt. Upon completion, the solvent was evaporated and purified using Flash chromatography. Remaining grease was taken up in diethyl ether while the titled product precipitated as a grey

solid (80 mg, 34 %). HPLC-DAD: 254 nm: 98.0 %, 230 nm: 96.5 %; ESI-HRMS: *m/z* [M+H]+ = 379.07231 calcd.; 379.07276 found; 1H NMR (400 MHz, DMSO) δ 9.35 (s, 1H), 8.89 (s, 1H), 7.77 (s, 1H), 7.72 (s, 2H), 6.48 (s, 1H), 3.87 (s, 2H), 3.23 (s, 3H), 2.43 – 2.33 (m, 1H), 0.84 (dd, J = 6.7, 2.1 Hz, 2H), 0.61 (dd, J = 3.9, 2.3 Hz, 2H). 13C NMR (101 MHz, DMSO) δ 163.46, 152.24, 144.83, 141.79, 136.00, 131.49, 122.47, 120.59, 117.01, 92.97, 51.78, 29.77, 27.79, 7.34. IR (ATR) [cm-1]: 3228, 3052, 1653, 1598, 1477, 1429, 1258, 1161, 1148, 816.

Synthesis of 1-cyclopropyl-7-((2,6-difluoro-4-hydroxyphenyl)amino)-4-methyl-1,4-dihydropyrido[3,4-b]pyrazin-3(2H)-one (44)

4-((*tert*-butyldimethylsilyl)oxy)-2,6-difluoroaniline

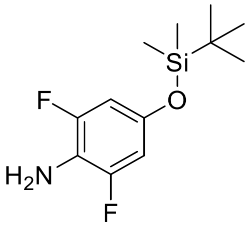

4-amino-3,5-difluorophenol (500 mg, 3.5 mmol) was dissolved in dry DMF (5 mL). The solution was charged with imidazole (469 mg, 6.9 mmol) and *tert*-butylchlorodimethylsilane (780 mg, 5.2 mmol) and stirred for 1 h. Upon completion, water was added and the mixture was extracted 3x with DCM. The combined organic layers were dried over sodium sulfate, evaporated and purified using Flash chromatography giving a transparent oil (670 mg, 75 %). ESI-MS: *m/z* 260.0 $[M+H]^+$ (260.1 calcd.); 1H NMR (400 MHz, DMSO) δ 6.45 (d, J = 9.9 Hz, 0H), 4.66 (s, 0H), 0.92 (s, 1H), 0.15 (s, 1H).

7-((4-((tert-butyldimethylsilyl)oxy)-2,6-difluorophenyl)amino)-1-cyclopropyl-4-methyl-1,4-dihydropyrido[3,4-b]pyrazin-3(2H)-one

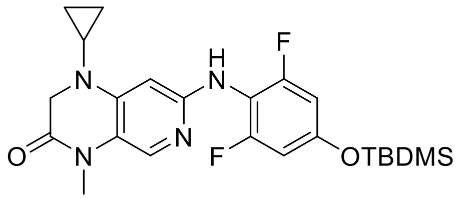

7-chloro-1-cyclopropyl-4-methyl-1,4-dihydropyrido[3,4-b]pyrazin-3(2H)-one (157 mg, 0.66 mmol), 4-((*tert*-butyldimethylsilyl)oxy)-2,6-difluoroaniline (206 mg, 0.79 mmol), Xphos Pd G4 (11 mg, 0.013 mmol) and KOtBu (148 mg, 1.3 mmol) were added to a dry flask with a stirrer in an argon atmosphere. Anhydrous toluene (9 mL) was added. The mixture was refluxed for 4 h in an argon atmosphere. After full conversion, the reaction mixture was filtered over celite, evaporated and purified using Flash chromatography yielding an off-white material (80 mg, 26 %).

1-cyclopropyl-7-((2,6-difluoro-4-hydroxyphenyl)amino)-4-methyl-1,4-dihydropyrido[3,4-b]pyrazin-3(2H)-one (44)

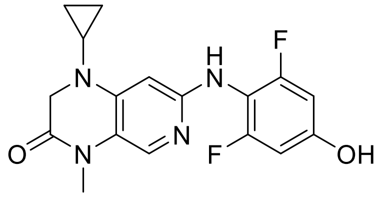

7-((4-((tert-butyldimethylsilyl)oxy)-2,6-difluorophenyl)amino)-1-cyclopropyl-4-methyl-1,4-dihydropyrido[3,4-b]pyrazin-3(2H)-one (80 mg, 0.17 mmol) was dissolved in THF. 1 M tetrabutylammonium fluoride in THF (1 mL, 0.87 mmol) was added and stirred for 2 h at rt. Upon completion, the solvent was evaporated and purified using Flash chromatography. Remaining grease was taken up in diethyl ether while the titled product precipitated as a white

solid (80 mg, 34 %). HPLC-DAD: 254 nm: 97.1 %, 230 nm: 97.0 %; ESI-HRMS: *m/z* [M+H]+ = 347.13141 calcd.; 347.13190 found; 1H NMR (400 MHz, DMSO) δ 10.10 (s, 1H), 7.77 (s, 1H), 7.53 (s, 1H), 6.54 – 6.42 (m, 2H), 6.34 (s, 1H), 3.82 (s, 2H), 3.17 (s, 3H), 2.40 – 2.28 (m, 1H), 0.83 – 0.74 (m, 2H), 0.61 – 0.52 (m, 2H). 13C NMR (101 MHz, DMSO) δ 163.43, 159.29 (dd, J = 243.9, 8.8 Hz), 155.75 (t, J = 14.4 Hz), 144.98, 132.02, 119.93, 108.96 (t, J = 17.3 Hz), 99.09 (d, J = 26.1 Hz), 90.84, 51.90, 29.77, 27.73, 7.27. IR (ATR) [cm-1]: 3256, 2796, 2590, 1667, 1616, 1517, 1476, 1262, 1148, 1022.

Synthesis of 3-chloro-5-((1-cyclopropyl-4-methyl-3-oxo-1,2,3,4-tetrahydropyrido[3,4-b]pyrazin-7-yl)amino)-2-hydroxybenzonitrile (45)

3-chloro-2-hydroxy-5-nitrobenzonitrile

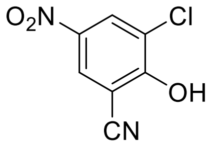

3-chloro-2-hydroxybenzonitrile (5.0 g, 32.6 mmol) was dissolved in glacial acetic acid (20 mL). The reaction was cooled to 5 °C. A mixture of fuming nitric acid (3.0 mL) and glacial acetic acid (6.0 mL) was added dropwise keeping the temperature between 5 – 10 °C. After full addition of the nitrating acid, the reaction was stirred until full consumption of the precursor ~ 1 h. Upon completion, the mixture was poured over ice water. The precipitate was suction filtered giving a beige powder (4.56 g, 71 %). ESI-MS: *m/z* 196.9 $[M-H]^-$ (197.0 calcd.); 1H NMR (400 MHz, DMSO) δ 11.47 (s, 1H), 8.50 (d, J = 2.8 Hz, 1H), 8.43 (d, J = 2.8 Hz, 1H). 13C NMR (101 MHz, DMSO) δ 163.17, 137.27, 129.40, 128.98, 122.92, 115.41, 101.62.

5-amino-3-chloro-2-hydroxybenzonitrile

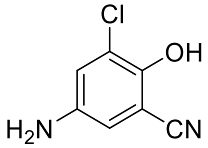

3-chloro-2-hydroxy-5-nitrobenzonitrile (4.5 g, 7.9 mmol) and NH_4OAc (8.73 g, 113.3 mmol) were dissolved in 1,4-dioxane/H_2O (4:1, 40 mL). The mixture was heated to 80 °C. The heat source was removed and Zn dust (10.4 g, 158.6 mmol) was added. The temperature raised to 110 °C while stirring vigorously. After 20 min the temperature decreased and the reaction was cooled to room temperature and the solvent was evaporated. The residue was taken up in water and EtOAc. The phases was separated and the aqueous phase was extracted 2x with EtOAc. The combined organic layers were washed with brine, dried over sodium sulfate and evaporated giving the titled product as a brown-red solid (1.49 g, 39 %). ESI-MS: *m/z* 166.9 $[M-H]^-$ (167.0 calcd.); 1H NMR (400 MHz, DMSO) δ 9.71 (s, 1H), 6.92 (d, J = 2.7 Hz, 1H), 6.71 (d, J = 2.7 Hz, 1H), 5.22 (s, 2H). 13C NMR (101 MHz, DMSO) δ 145.28, 143.15, 123.27, 120.05, 116.73, 115.38, 102.99.

5-amino-2-((tert-butyldimethylsilyl)oxy)-3-chlorobenzonitrile

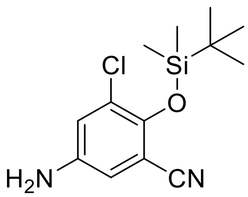

5-amino-3-chloro-2-hydroxybenzonitrile (1.45 g, 8.6 mmol) was dissolved in dry DMF (5 mL). The solution was charged with imidazole (1.17 g, 17.2 mmol) and tert-butylchlorodimethylsilane (1.95 g, 13.0 mmol) and stirred for 1 h. Upon completion, water was added and the mixture was extracted 3x with DCM. The combined organic layers were dried

over sodium sulfate, evaporated and purified using Flash chromatography giving a white solid (1.25 g, 51 %). ESI-MS: *m/z* 281.0 $[M-H]^-$ (281.1 calcd.).

3-chloro-5-((1-cyclopropyl-4-methyl-3-oxo-1,2,3,4-tetrahydropyrido[3,4-b]pyrazin-7-yl)amino)-2-hydroxybenzonitrile (45)

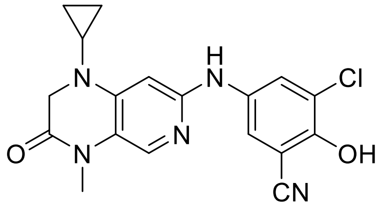

7-chloro-1-cyclopropyl-4-methyl-1,4-dihydropyrido[3,4-b]pyrazin-3(2H)-one (300 mg, 1.26 mmol), 5-amino-2-((tert-butyldimethylsilyl)oxy)-3-chlorobenzonitrile (427 mg, 1.51 mmol), Xantphos Pd G4 (24 mg, 0.025 mmol) and KOtBu (170 mg, 1.51 mmol) were added to a dry flask with a stirrer in an argon atmosphere. Anhydrous 1,4-dioxane (17 mL) was added. The mixture was refluxed for 4 h in an argon atmosphere. The mixture was cooled to rt and filtered over celite. The residue was evaporated and purified using normal phase Flash chromatography with a gradient of DCM to 10 % MeOH in DCM and reverse phase using a gradient of 0.1 % FA in water to 100 % MeOH giving the desired product as a grey solid (33 mg, 7 %). HPLC-DAD: 254 nm: 100 %, 230 nm: 100 %; ESI-HRMS: *m/z* [M+H]+ = 370.10653 calcd.; 370.10707 found; 1H NMR (400 MHz, DMSO-d_6) δ 10.37 (s, 1H), 9.06 (s, 1H), 8.03 (d, J = 2.7 Hz, 1H), 7.92 (d, J = 2.6 Hz, 1H), 7.79 (s, 1H), 6.48 (s, 1H), 3.88 (s, 2H), 3.23 (s, 3H), 2.43 – 2.36 (m, 1H), 0.89 – 0.81 (m, 2H), 0.66 – 0.58 (m, 2H). 13C NMR (101 MHz, DMSO) δ 163.48, 152.03, 144.90, 131.48, 123.36, 122.35, 120.80, 119.31, 116.61, 102.17, 93.06, 51.76, 29.78, 27.81, 7.36. IR (ATR) [cm-1]: 2964, 2200, 1669, 1615, 1540, 1457, 1344, 1233, 835.

Synthesis of 1-cyclopropyl-7-((4-hydroxy-3,5-bis(trifluoromethyl)phenyl)amino)-4-methyl-1,4-dihydropyrido[3,4-b]pyrazin-3(2H)-one (46)

2-bromo-5-nitro-1,3-bis(trifluoromethyl)benzene

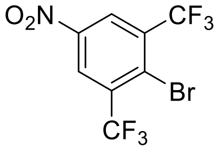

2-bromo-1,3-bis(trifluoromethyl)benzene (9.85 g, 33.6 mmol) was dissolved in sulfuric acid (0.5 mL). The reaction was cooled to 5 °C. A mixture of fuming nitric acid (3.1 mL) and sulfuric acid (6.2 mL) was added dropwise keeping the temperature under 10 °C. The reaction was carefully heated to 50 °C and stirred for 16 h. The reaction was quenched over ice water, the precipitate was suction filtered, giving the titled product as colorless crystals (10.5 g, 93 %). 1H NMR (400 MHz, CDCl3) δ 8.73 (s, 2H). 13C NMR (101 MHz, CDCl3) δ 146.46, 134.98 (q, J = 32.8 Hz), 126.86, 125.93 (q, J = 5.7 Hz), 121.64 (q, J = 275.1 Hz).

2-(benzyloxy)-5-nitro-1,3-bis(trifluoromethyl)benzene

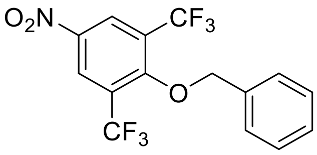

KOH was stirred in benzyl alcohol at rt. 2-bromo-5-nitro-1,3-bis(trifluoromethyl)benzene in DMSO was added dropwise and stirred for 30 min. Water was added and the aqueous phase was extracted 3x with EtOAc. The combined organic layers were washed with water and brine, dried over sodium sulfate, evaporated and purified using Flash chromatography giving the titled compound as yellow needles (1.31 g, 48 %) that were used without further purification.

4-(benzyloxy)-3,5-bis(trifluoromethyl)aniline

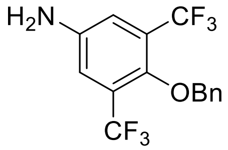

2-(benzyloxy)-5-nitro-1,3-bis(trifluoromethyl)benzene (1.3 g, 3.56 mmol) and NH_4OAc (0.82 g, 10.7 mmol) were dissolved in a mixture of 1,4-dioxane/H_2O (4:1) (1 mL, 4:1) and heated to 70 °C. Zn dust (1.16 g, 17.8 mmol) was added portion wise and stirred at 80 °C for 1 h. Upon completion, water and sat. aqueous NH_4Cl solution were added and extracted 3x with DCM. The combined organic layers were dried over sodium sulfate, evaporated and purified using Flash chromatography giving the titled compound as yellow crystals (445 mg, 37 %). ESI-MS: *m/z* 357.8 $[M+Na]^+$ (358.1 calcd.); 1H NMR (400 MHz, DMSO-d_6) δ 7.44 – 7.33 (m, 5H), 7.15 (s, 2H), 5.86 (s, 2H), 4.87 (s, 2H).

7-((4-(benzyloxy)-3,5-bis(trifluoromethyl)phenyl)amino)-1-cyclopropyl-4-methyl-1,4-dihydropyrido[3,4-b]pyrazin-3(2H)-one

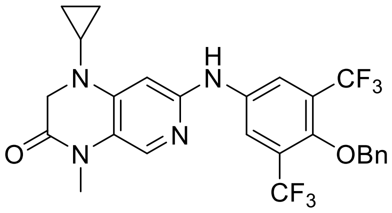

7-chloro-1-cyclopropyl-4-methyl-1,4-dihydropyrido[3,4-b]pyrazin-3(2H)-one (250 mg, 1.05 mmol), 4-(benzyloxy)-3,5-bis(trifluoromethyl)aniline (388 mg, 1.16 mmol), Xantphos Pd G4 (20 mg, 0.02 mmol) and KOtBu (236 mg, 2.1 mmol) were added to a dry flask with a stirrer in an argon atmosphere. Anhydrous 1-4dioxane (14 mL) was added. The mixture was heated at 80 °C for 4 h in an argon atmosphere. Upon completion, water was added and extracted 3x with DCM.

The combined organic layers were dried over sodium sulfate, evaporated and purified using Flash chromatography giving the titled compound as an orange oil that was used in the next step without further purification. $[M-H]^-$ =. ESI-MS: *m/z* 534.8 $[M+H]^+$ (535.2 calcd.).

1-cyclopropyl-7-((4-hydroxy-3,5-bis(trifluoromethyl)phenyl)amino)-4-methyl-1,4-dihydropyrido[3,4-b]pyrazin-3(2H)-one (46)

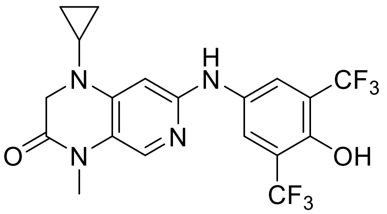

7-((4-(benzyloxy)-3,5-bis(trifluoromethyl)phenyl)amino)-1-cyclopropyl-4-methyl-1,4-dihydropyrido[3,4-b]pyrazin-3(2H)-one (58 mg, 0.11 mmol) and 0.05 mL 4 N HCl in 1,4-dioxane were added to EtOH (3 mL). The mixture was bubbled with nitrogen gas. Pd/C (6 mg) was added and the reaction mixture was bubbled with hydrogen gas for 16 h at rt. Upon completion, the mixture was filtered over Celite, evaporated and purified using Flash chromatography. The product was taken up in diethyl ether, sonicated and suction filtered yielding a white powder (22 mg, 46 %). HPLC-DAD: 254 nm: 92.7 %, 230 nm: 95.2 %; ESI-HRMS: *m/z* [M+H]+ = 447.12502 calcd.; 447.12571 found; 1H NMR (400 MHz, DMSO) δ 9.63 (s, 1H), 9.21 (s, 1H), 8.23 (s, 2H), 7.77 (s, 1H), 6.52 (s, 1H), 3.88 (s, 2H), 3.24 (s, 3H), 2.43 – 2.37 (m, 1H), 0.84 (dt, J = 6.7, 3.3 Hz, 2H), 0.65 – 0.58 (m, 2H). 13C NMR (101 MHz, DMSO-d_6) δ 163.52, 152.13, 144.94, 135.98, 131.52, 127.53, 124.81, 122.10, 121.74, 121.44, 121.15, 120.89, 118.53, 93.15, 51.77, 29.81, 27.83, 7.36. IR (ATR) [cm-1]: 3629, 2499, 3261, 3109, 1666, 1602, 1477, 1234, 1097, 827, 674.

NMR Spectra of final compounds

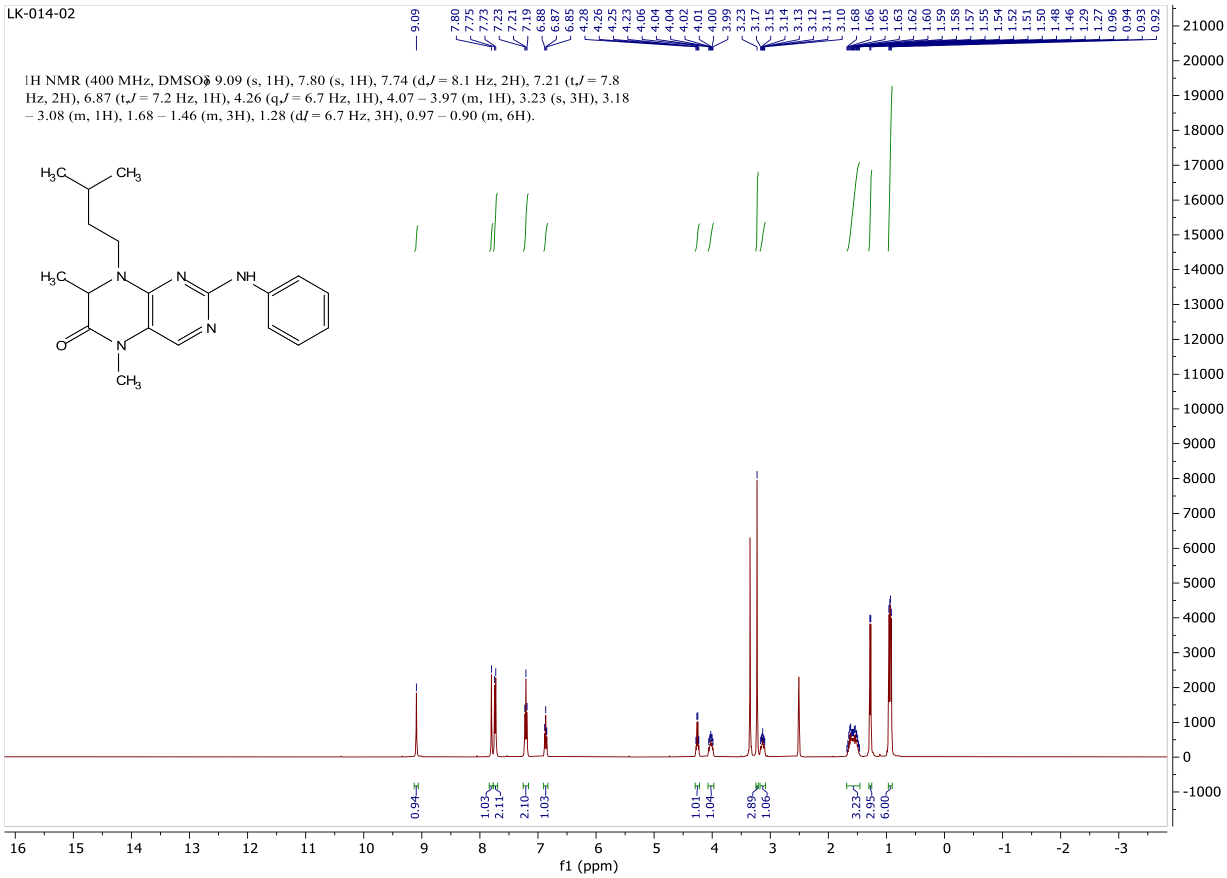

Figure S1: ^{1}H-NMR spectrum of 11 (400 MHz, DMSO-d_6).

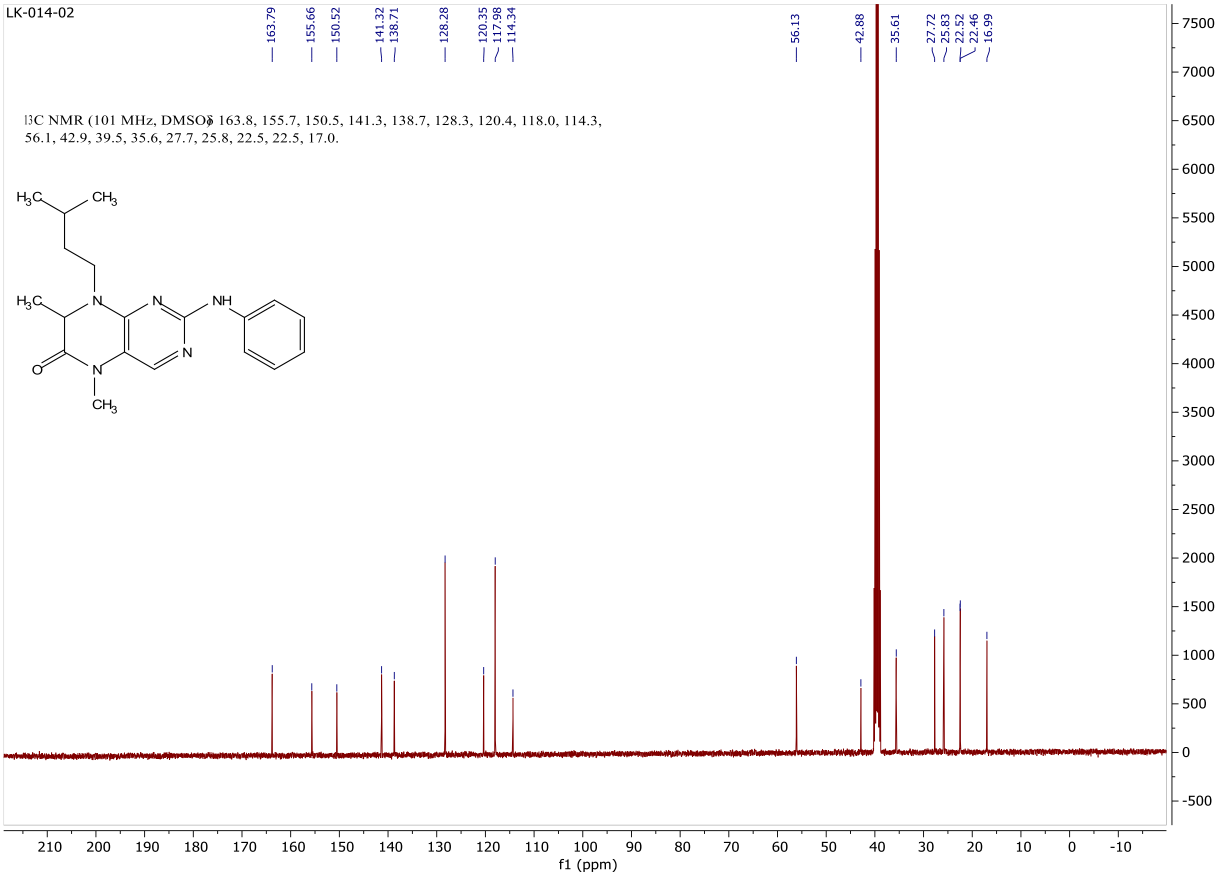

Figure S 2: ^{13}C-NMR spectrum of 11 (400 MHz, DMSO-d_6).

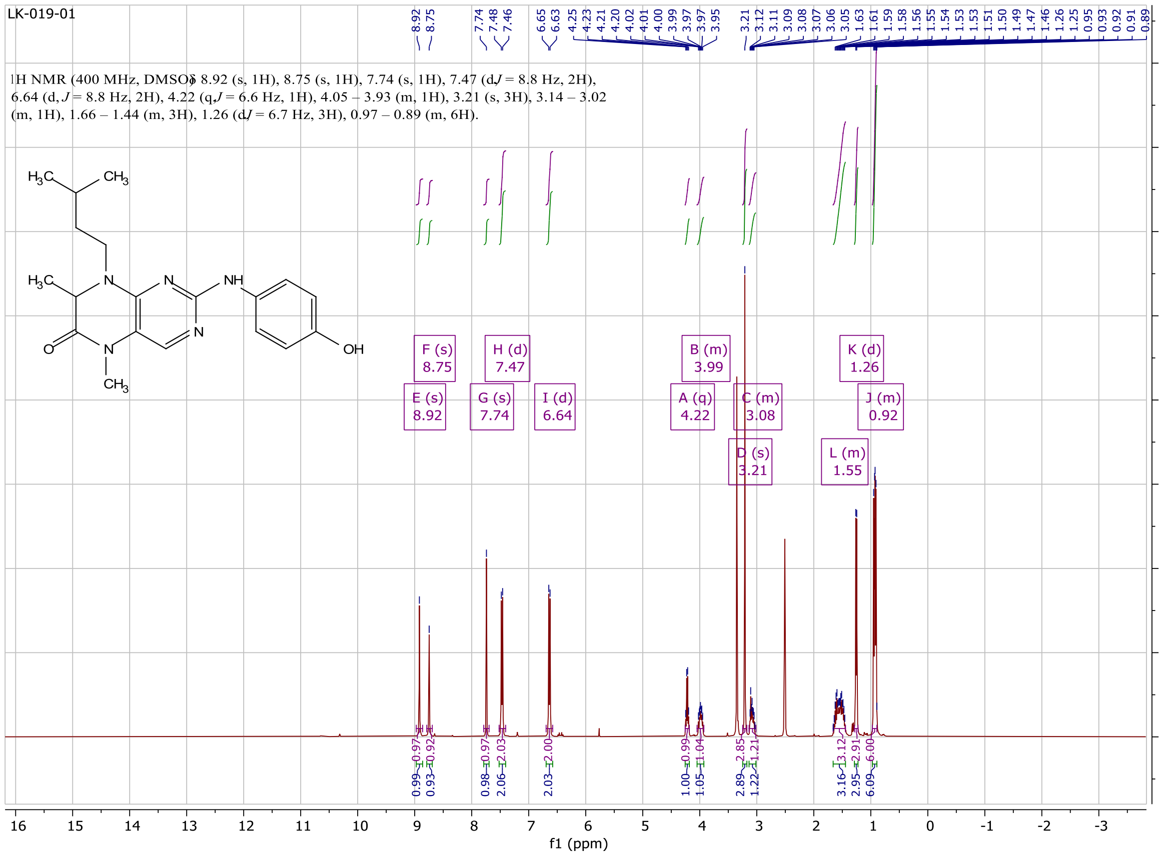

Figure S 3: ^{1}H-NMR spectrum of 12 (400 MHz, DMSO-d$_6$).

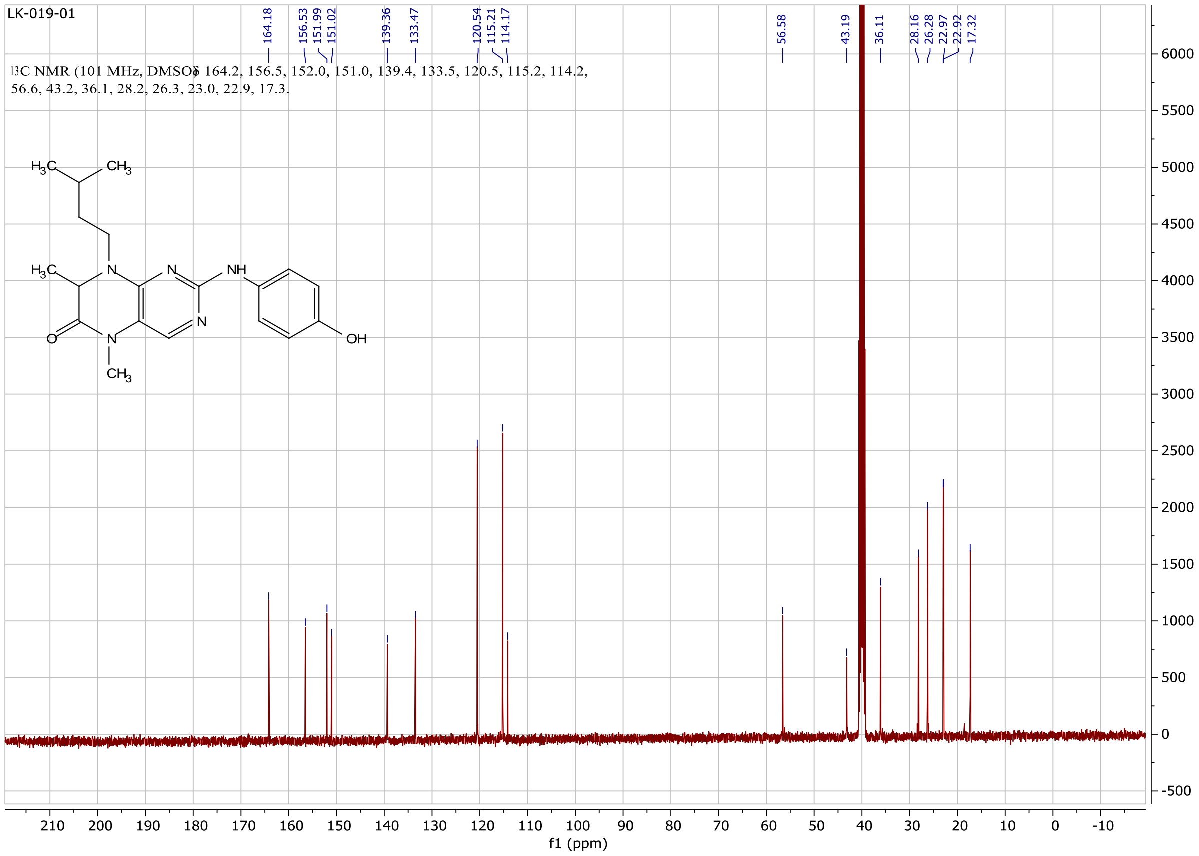

Figure S 4: ^{13}C-NMR spectrum of 12 (400 MHz, DMSO-d_6).

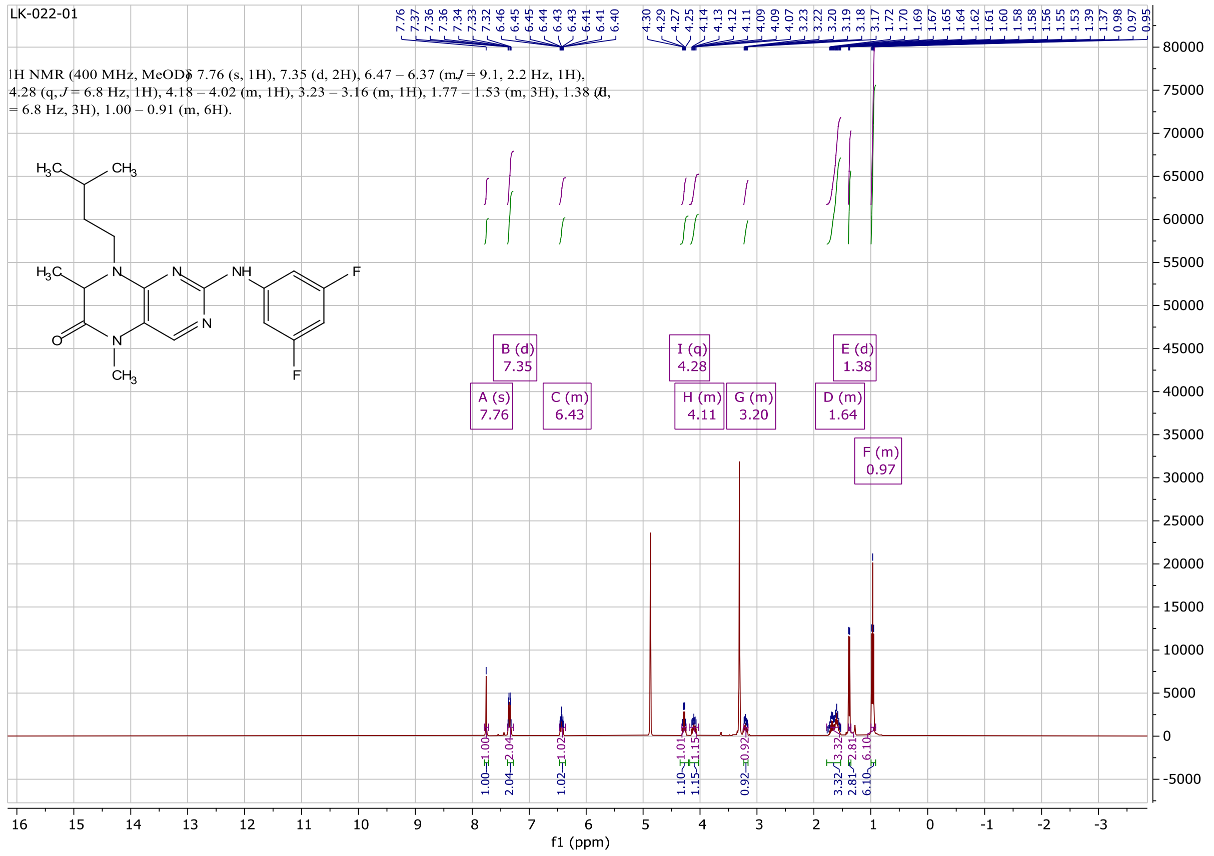

Figure S 5: ^{1}H-NMR spectrum of 13 (400 MHz, DMSO-d$_6$).

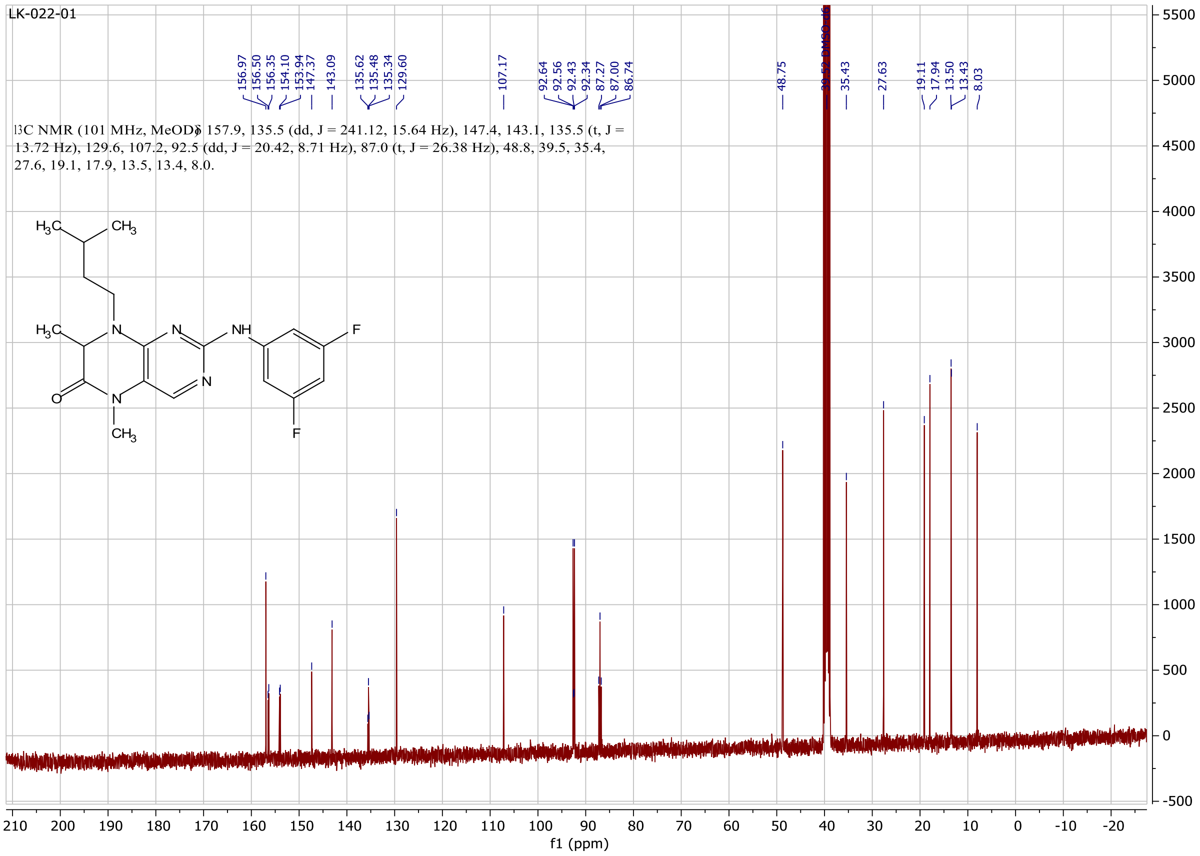

Figure S 6: ^{13}C-NMR spectrum of 13 (400 MHz, DMSO-d_6).

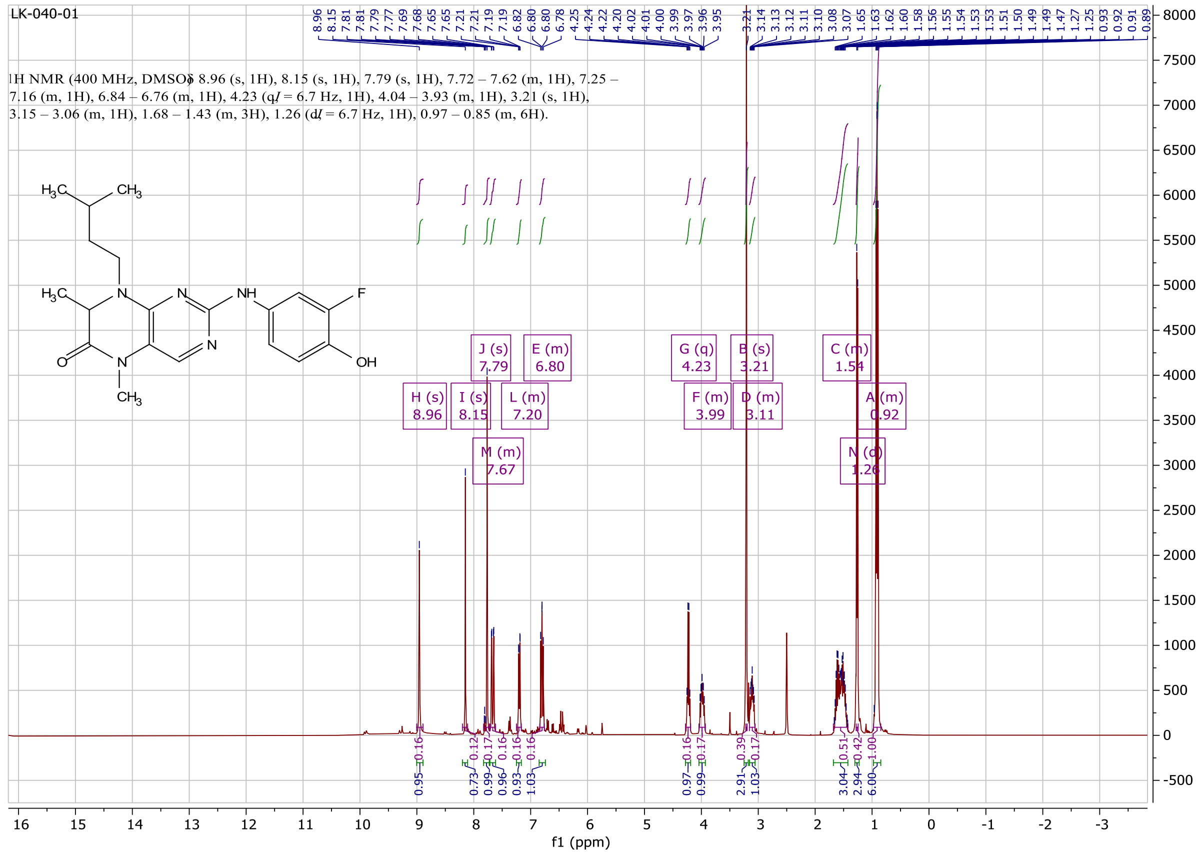

Figure S 7: ^{1}H-NMR spectrum of 14 (400 MHz, DMSO-d_6).

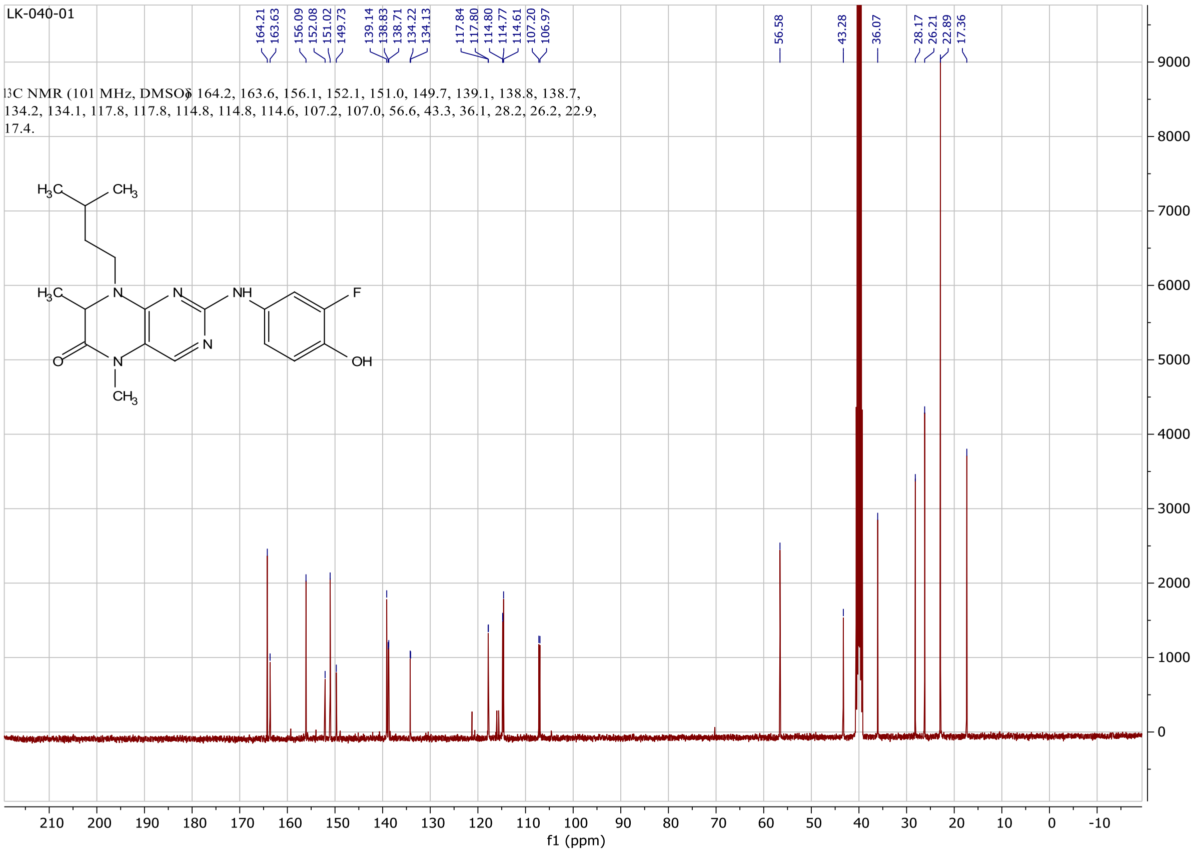

Figure S 8: ^{13}C-NMR spectrum of 14 (400 MHz, DMSO-d_6).

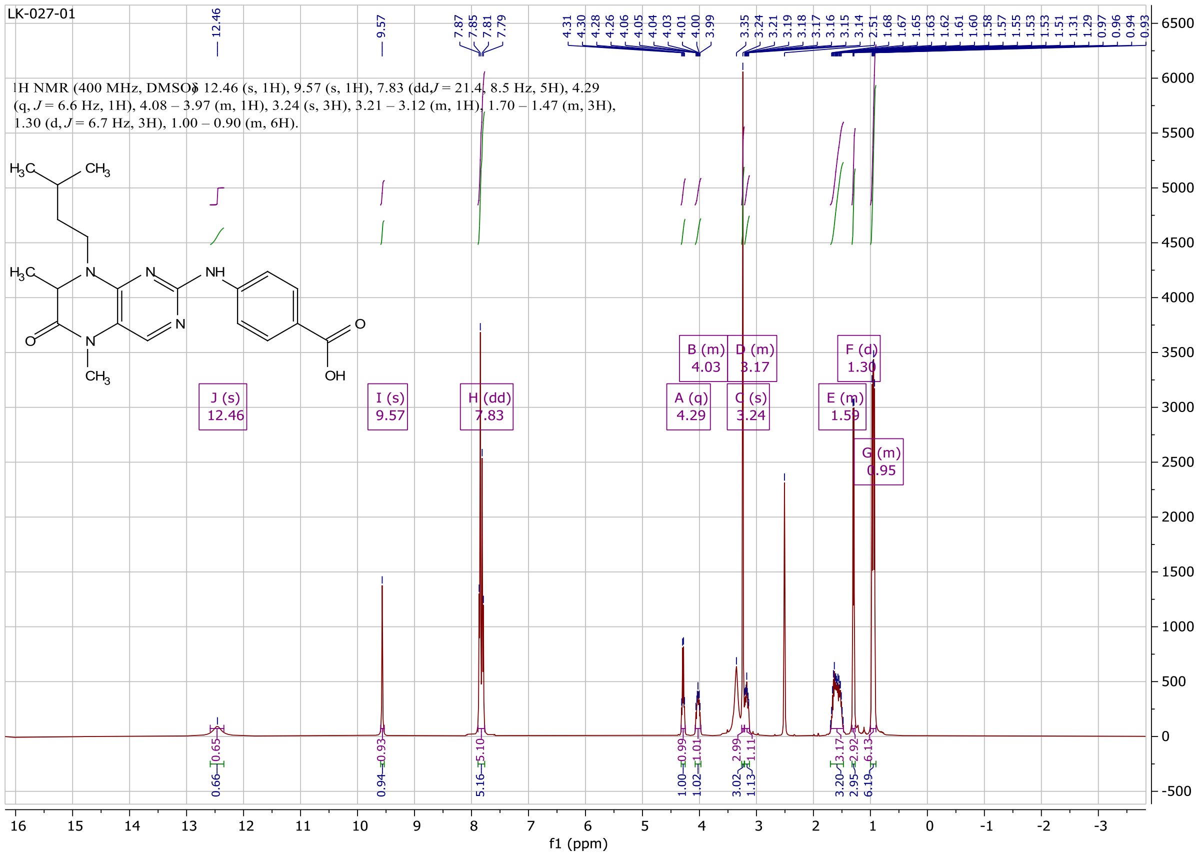

Figure S 9: ^{1}H-NMR spectrum of 15 (400 MHz, DMSO-d$_6$).

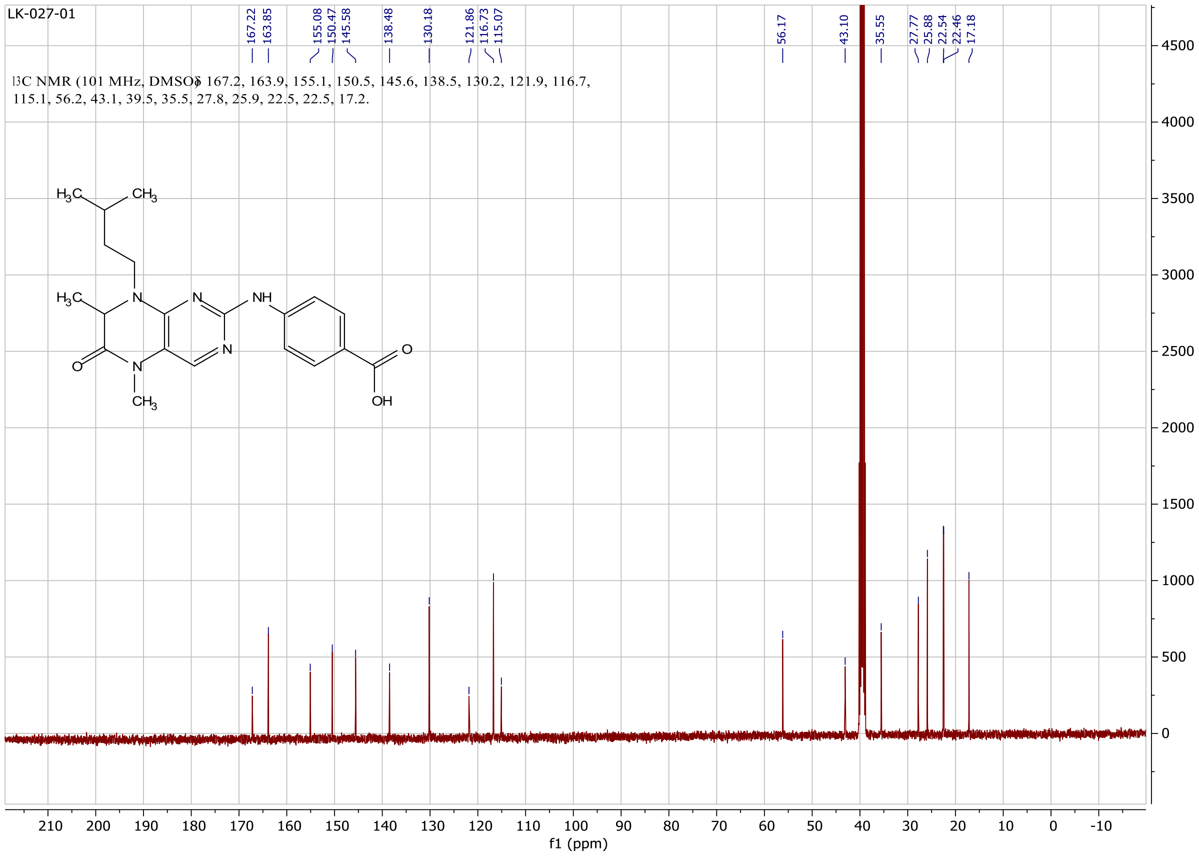

Figure S 10: ^{13}C-NMR spectrum of 15 (400 MHz, DMSO-d_6).

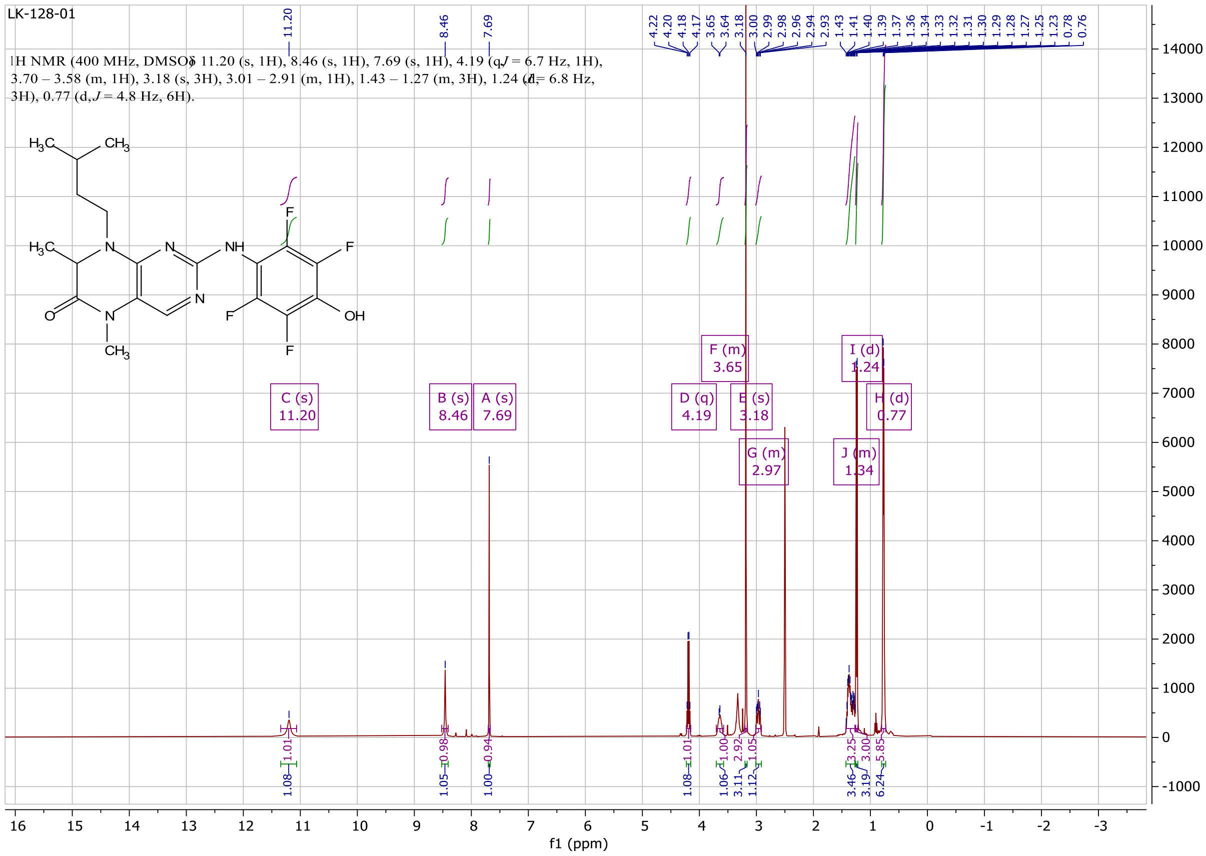

Figure S 11: ^{1}H-NMR spectrum of 16 (400 MHz, DMSO-d$_6$).

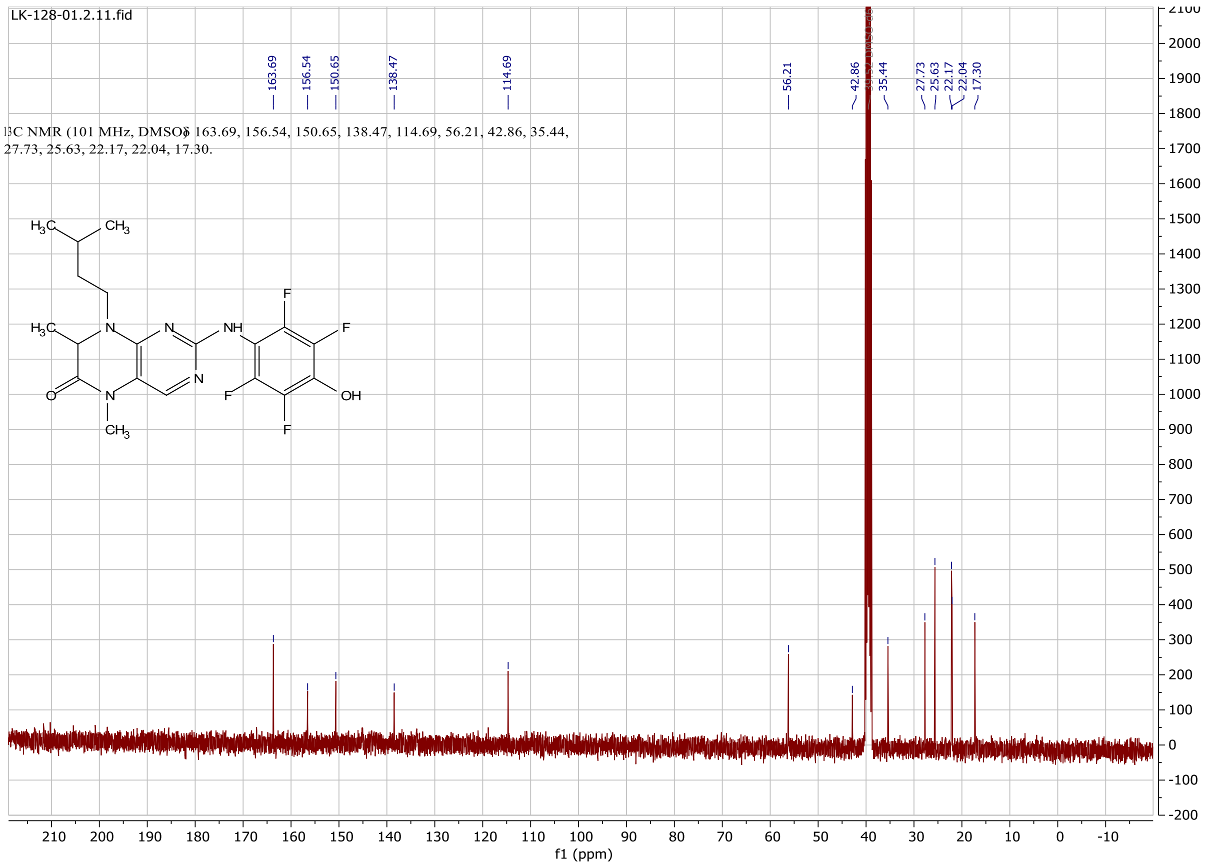

Figure S 12: ^{13}C-NMR spectrum of 16 (400 MHz, DMSO-d_6).

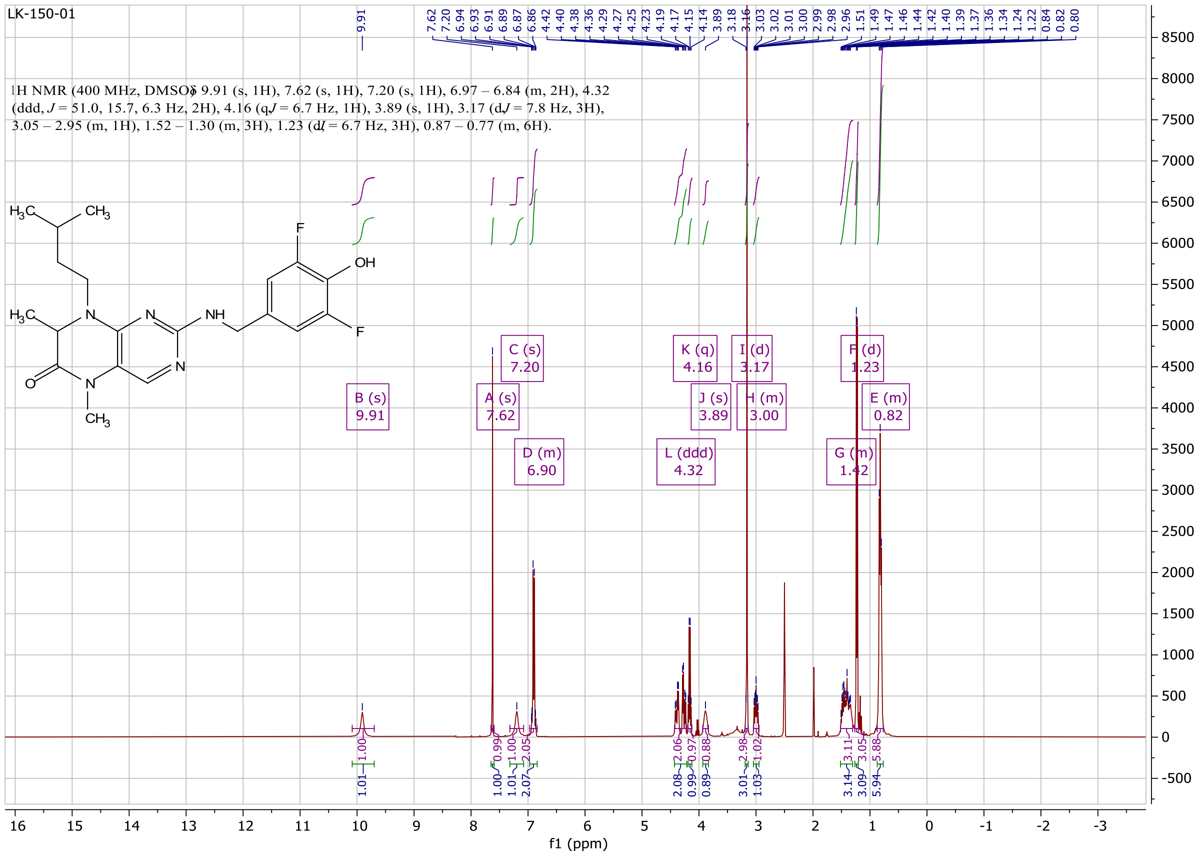

Figure S 13: ^{1}H-NMR spectrum of 17 (400 MHz, DMSO-d_6).

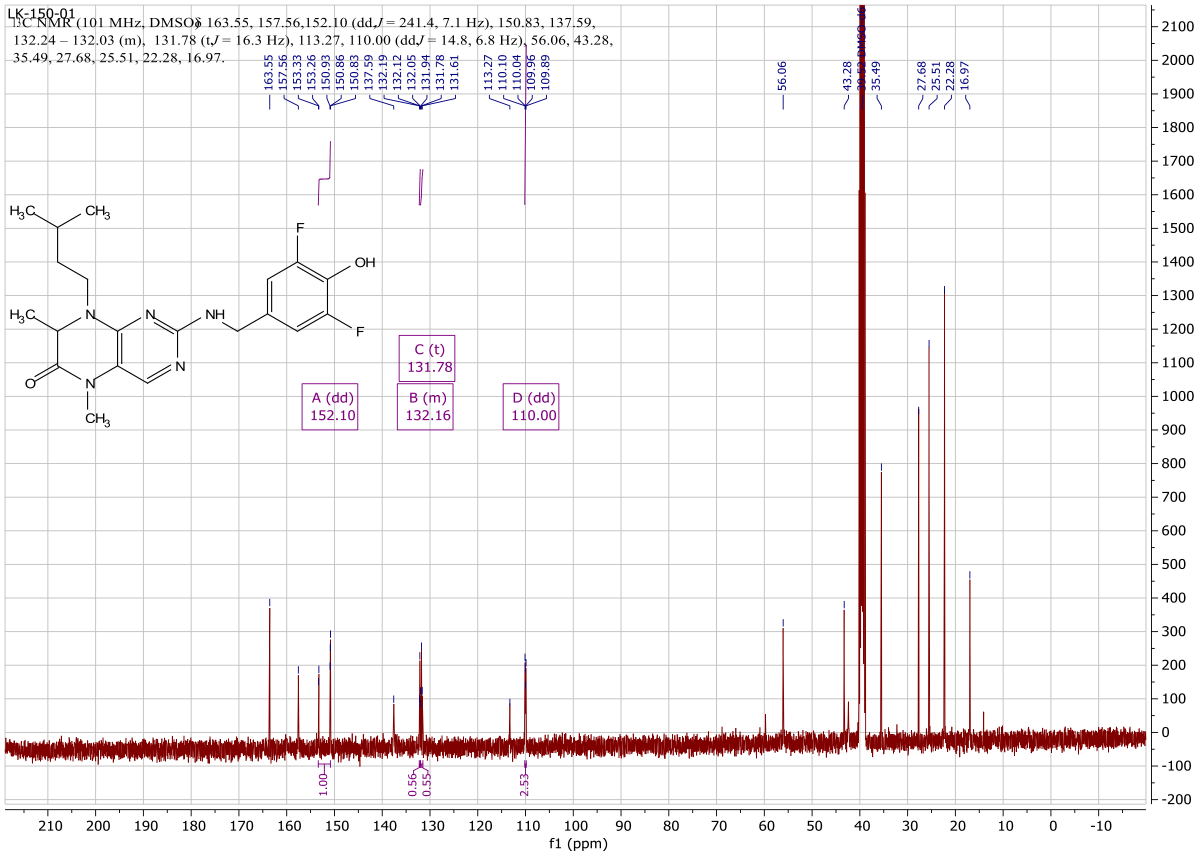

Figure S 14: ^{13}C-NMR spectrum of 17 (400 MHz, DMSO-d_6).

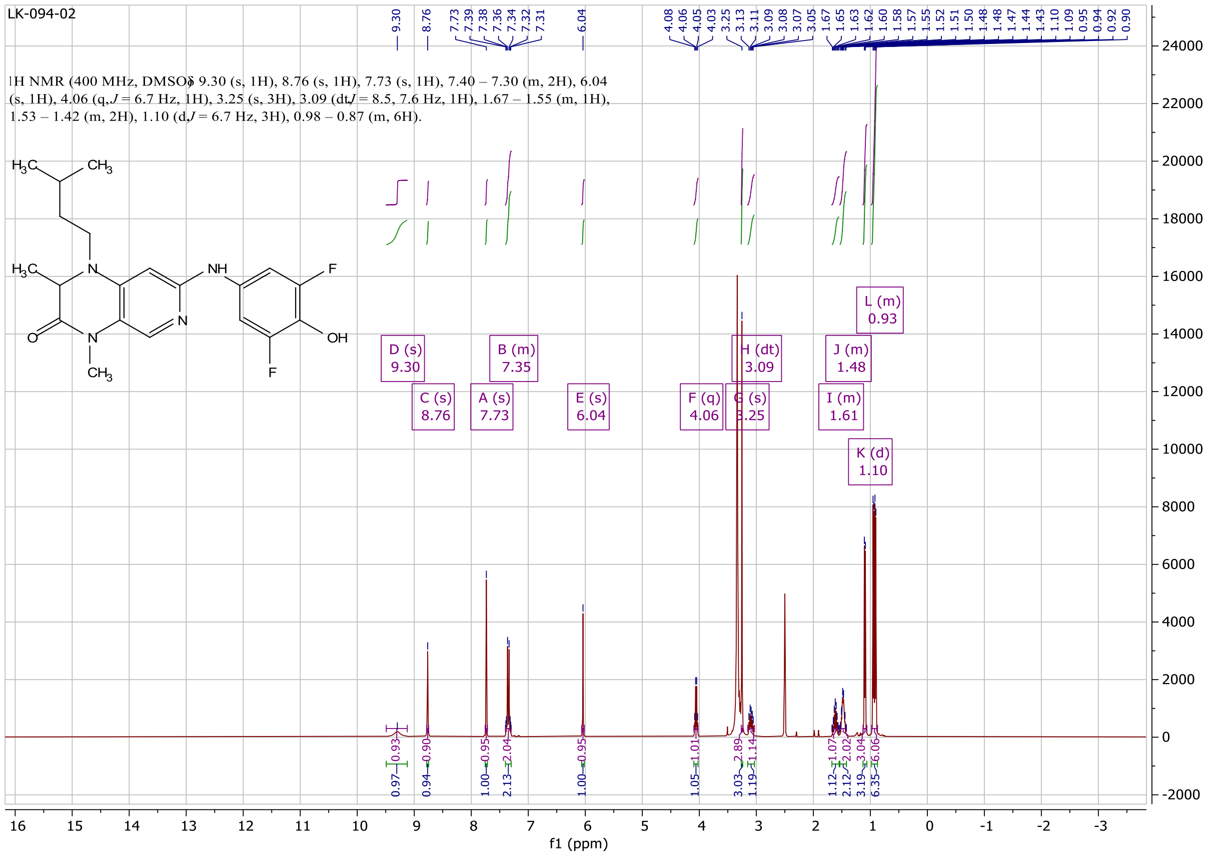

Figure S 15: ^{1}H-NMR spectrum of 18 (400 MHz, DMSO-d_6).

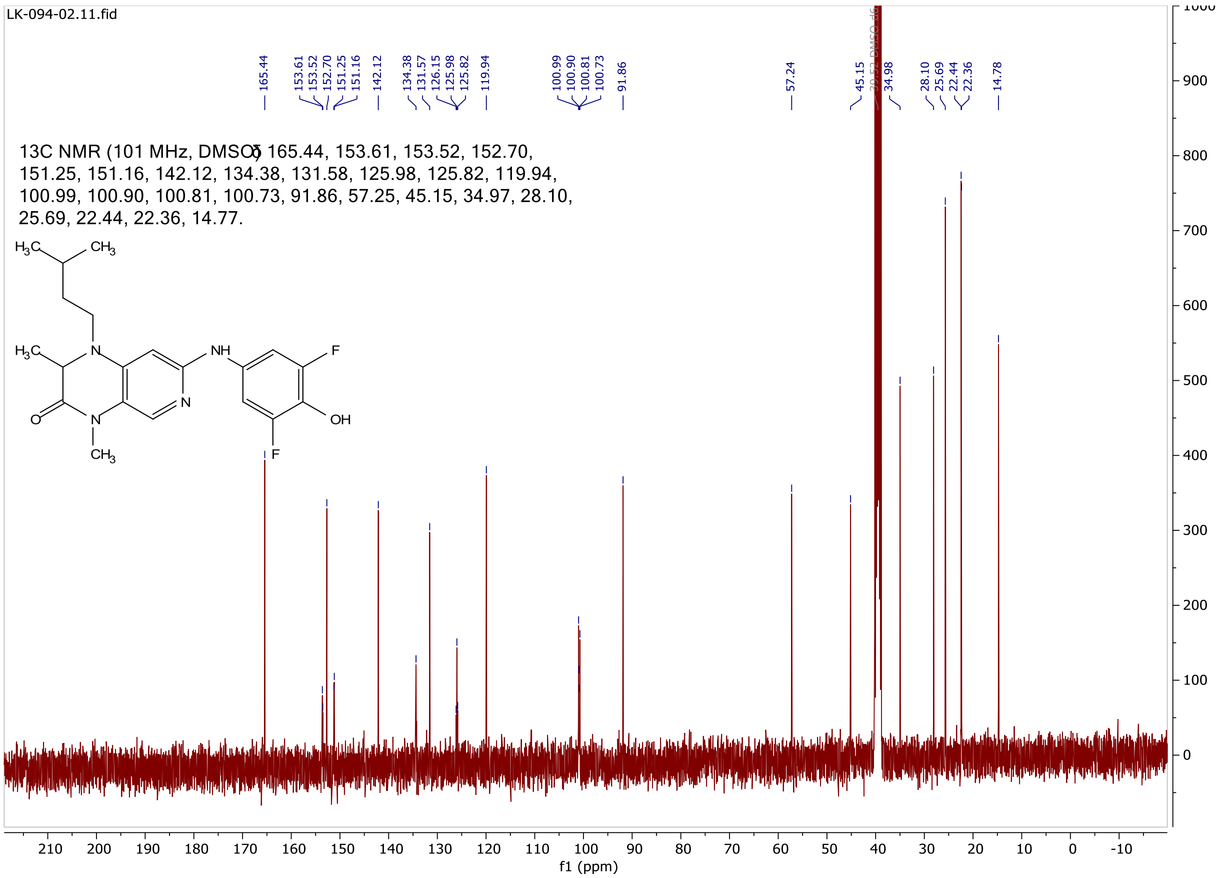

Figure S 16: ^{13}C-NMR spectrum of 18 (400 MHz, DMSO-d_6).

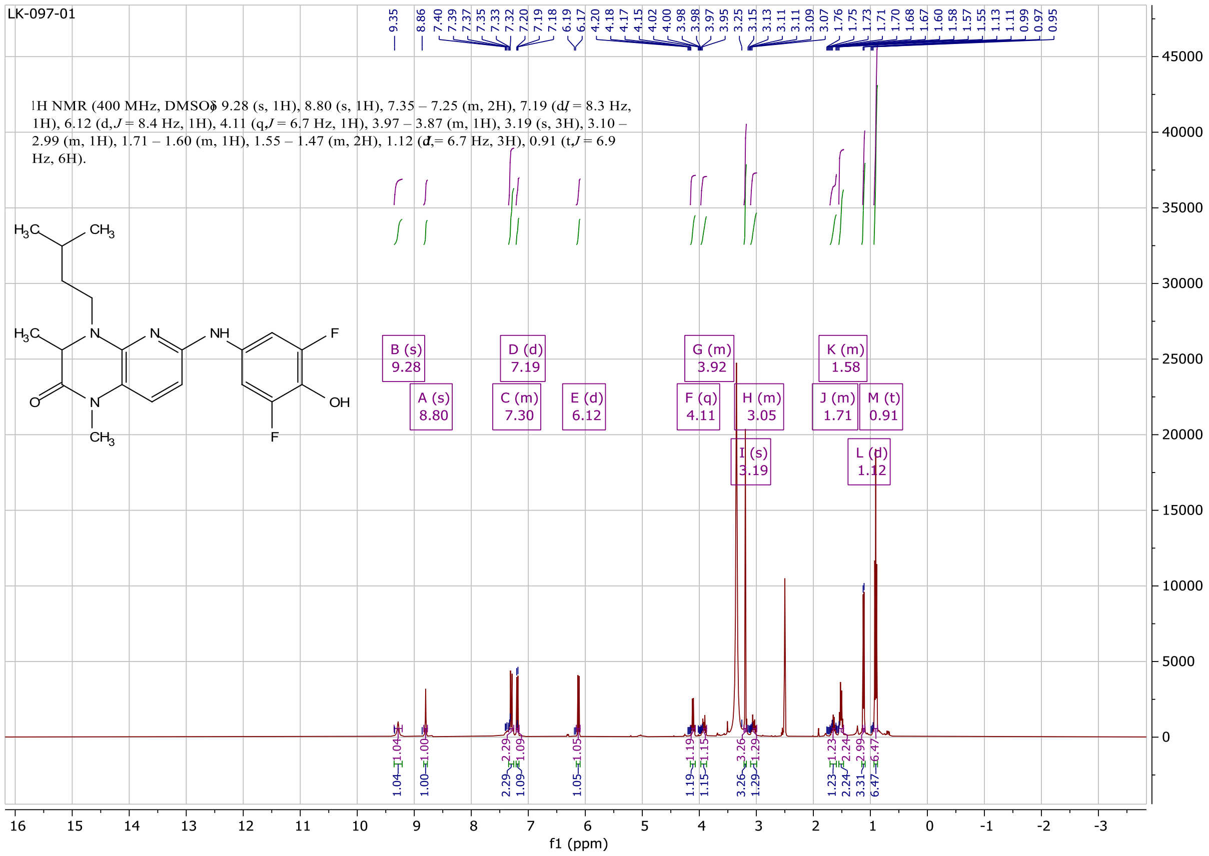

Figure S 17: ^{1}H-NMR spectrum of 19 (400 MHz, DMSO-d_6).

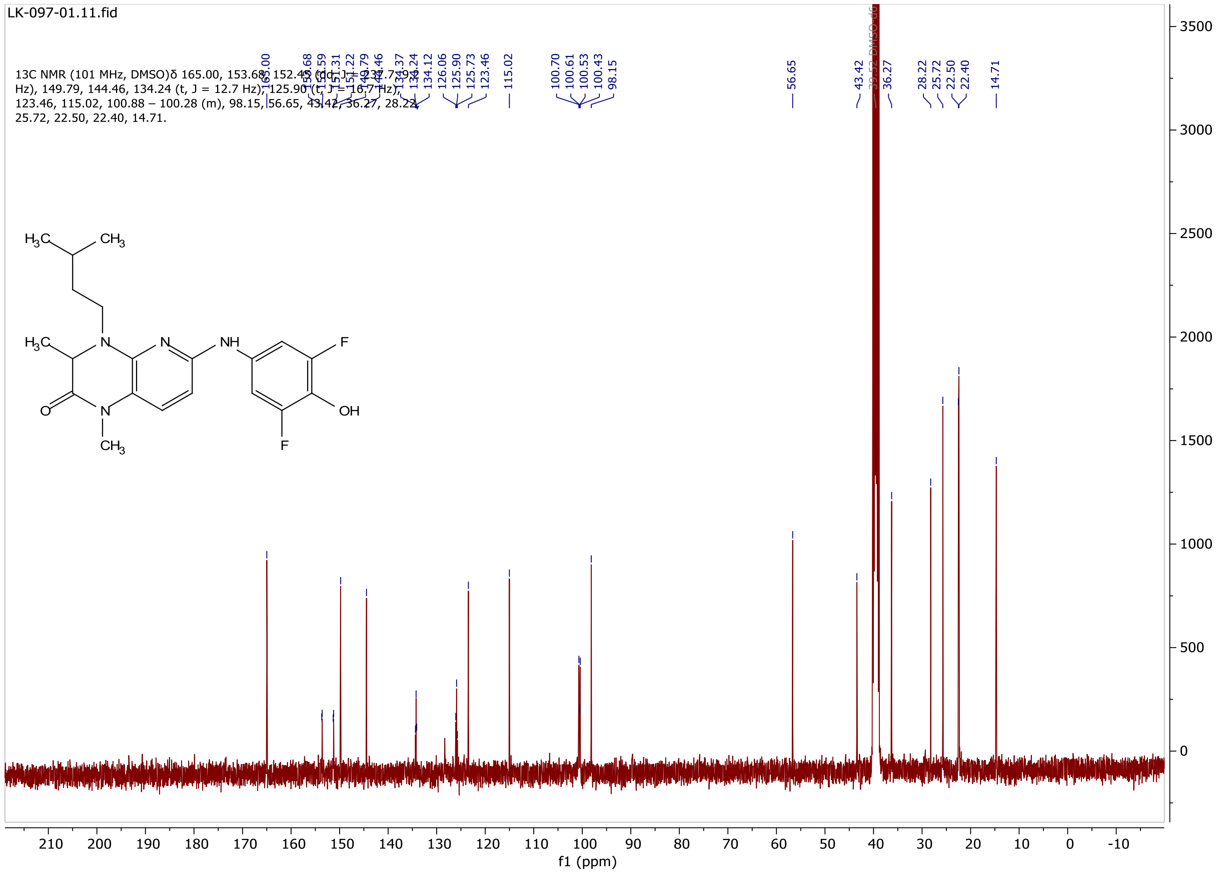

Figure S 18: ^{13}C-NMR spectrum of 19 (400 MHz, DMSO-d_6).

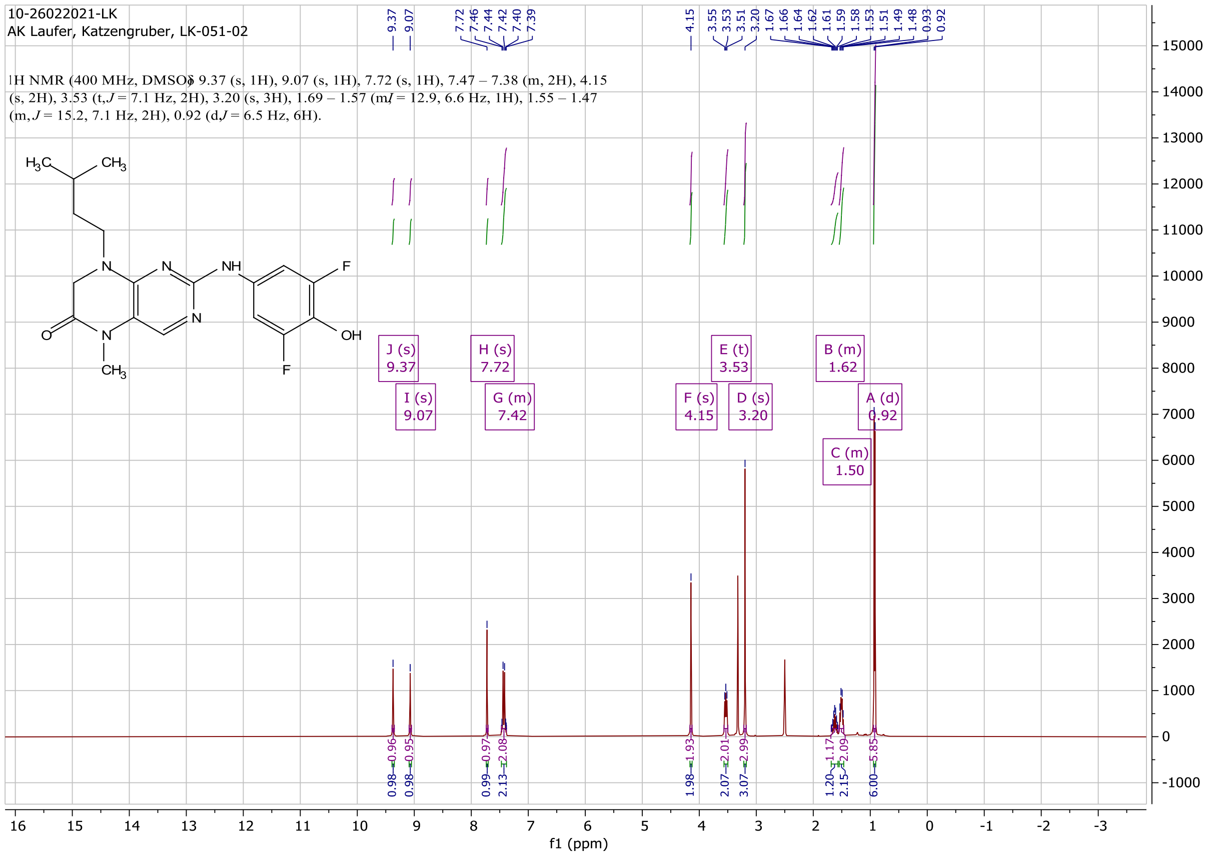

Figure S 19: ^{1}H-NMR spectrum of 20 (400 MHz, DMSO-d_6).

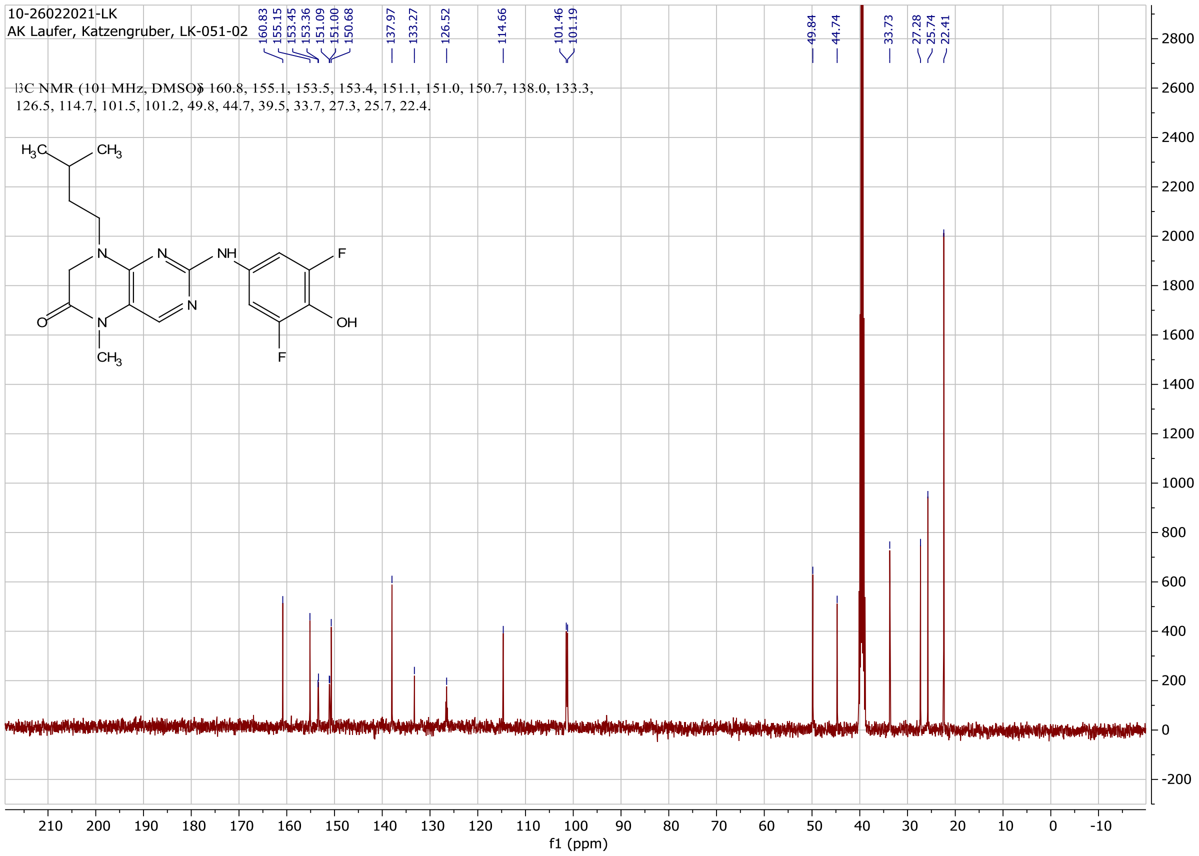

Figure S 20: ^{13}C-NMR spectrum of 20 (400 MHz, DMSO-d_6).

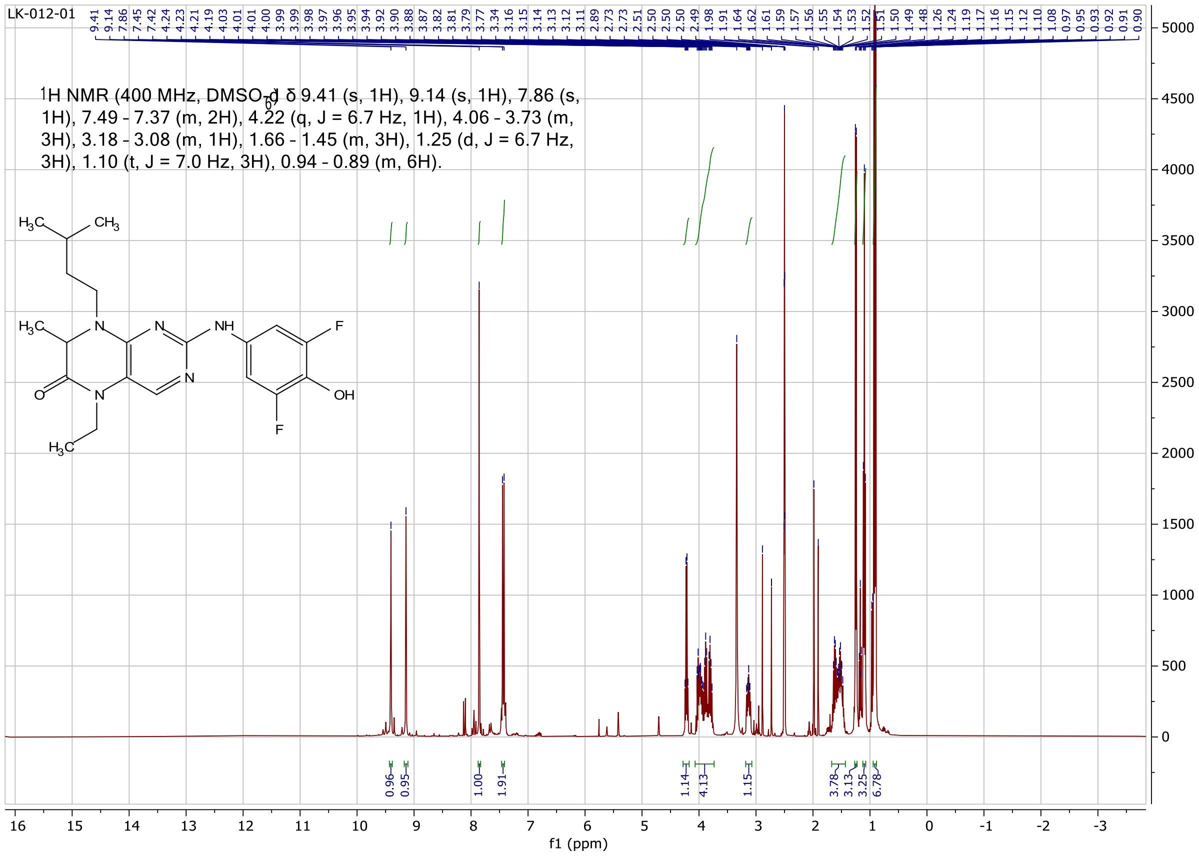

Figure S 21: ^{1}H-NMR spectrum of 21 (400 MHz, DMSO-d_6).

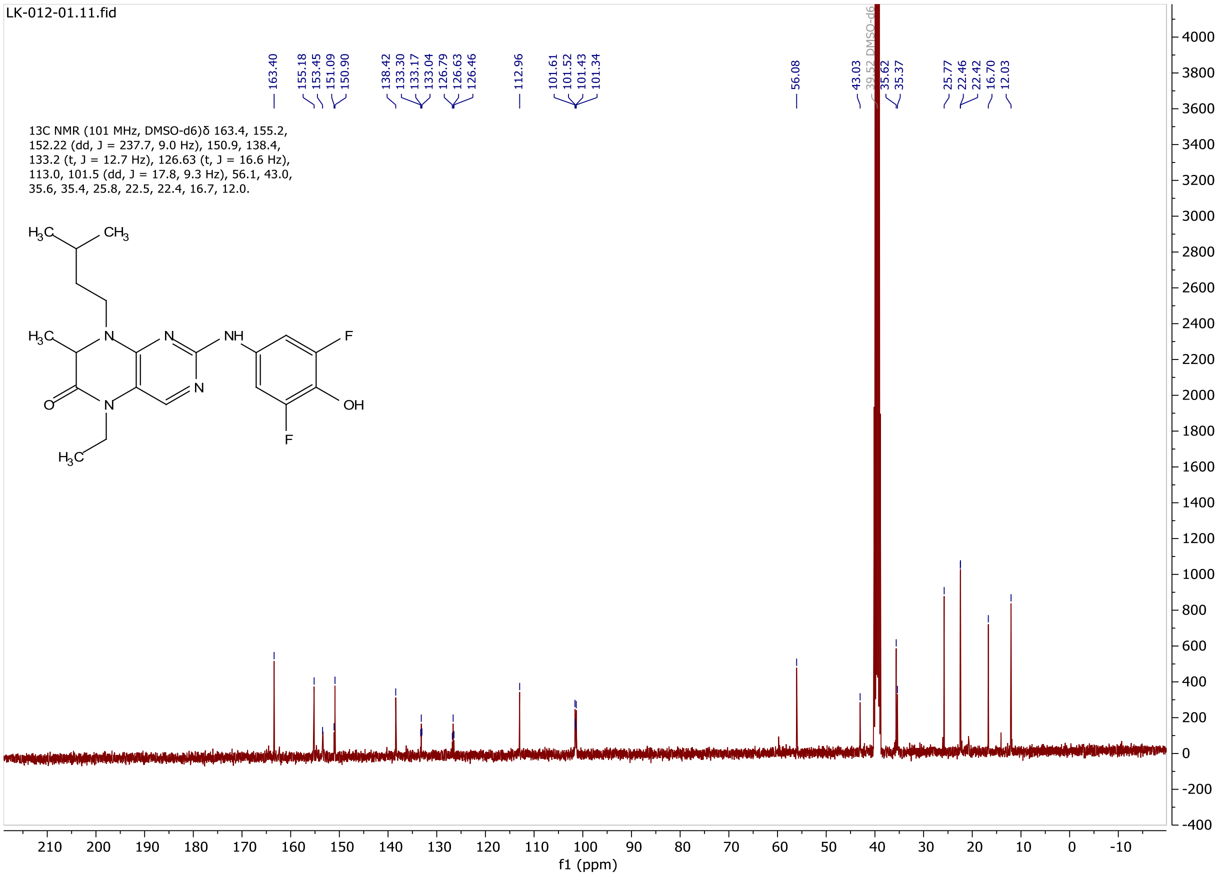

Figure S 22: ^{13}C-NMR spectrum of 21 (400 MHz, DMSO-d$_6$).

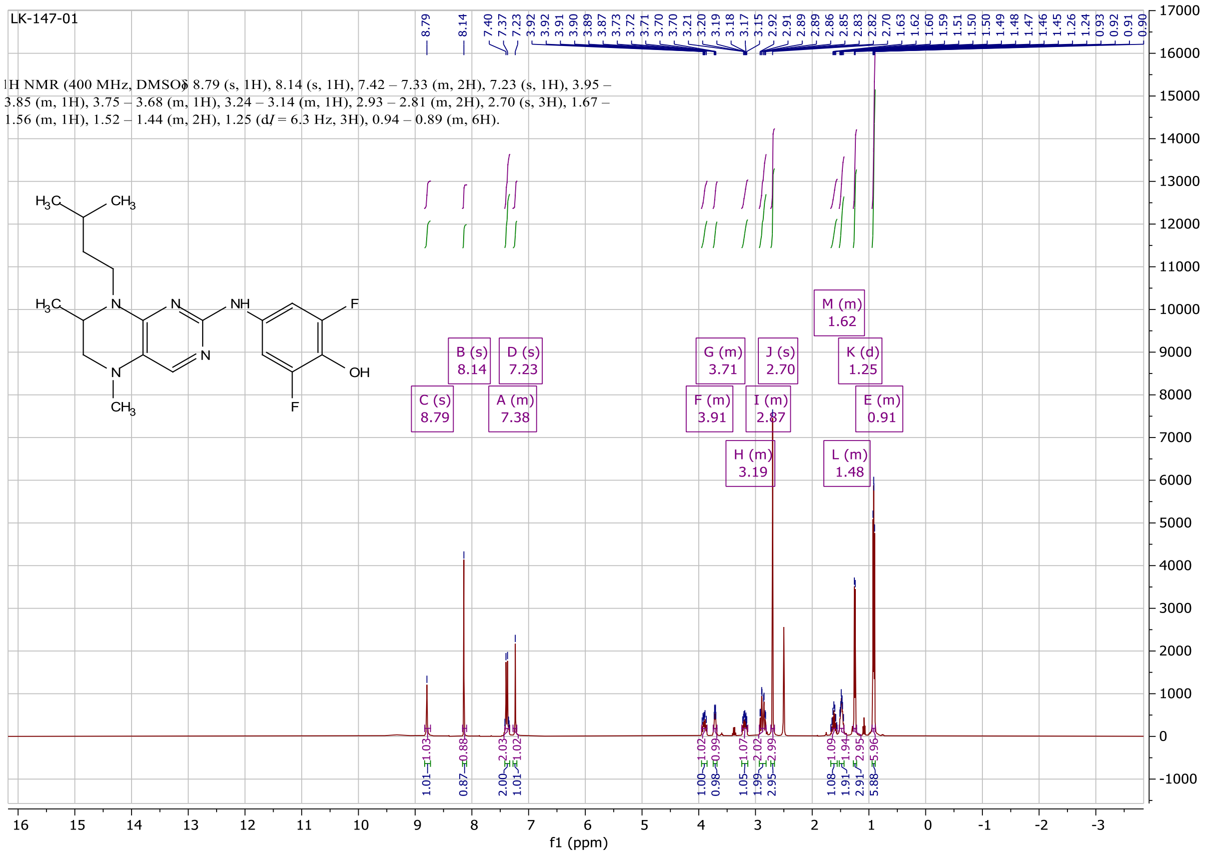

Figure S 23: ^{1}H-NMR spectrum of 22 (400 MHz, DMSO-d_6).

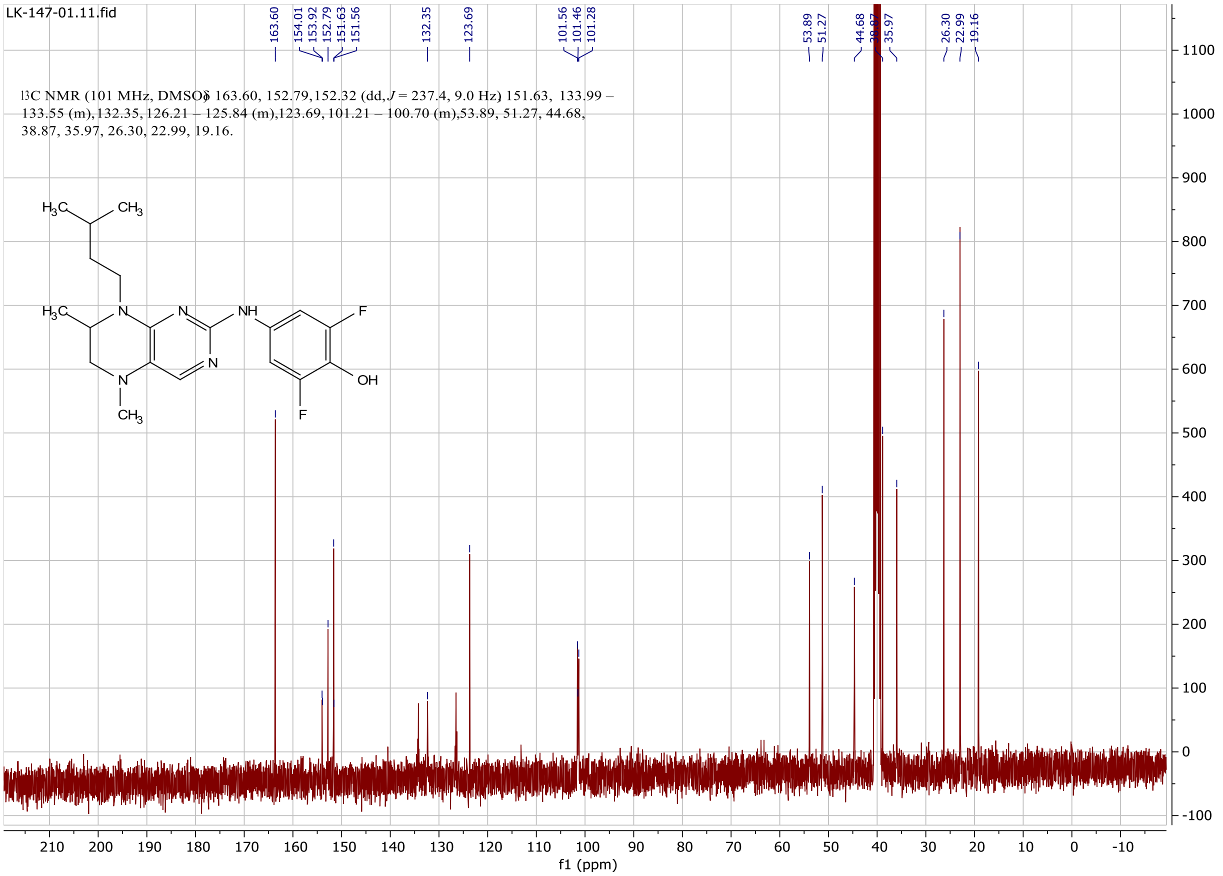

Figure S 24: ^{13}C-NMR spectrum of 22 (400 MHz, DMSO-d_6).

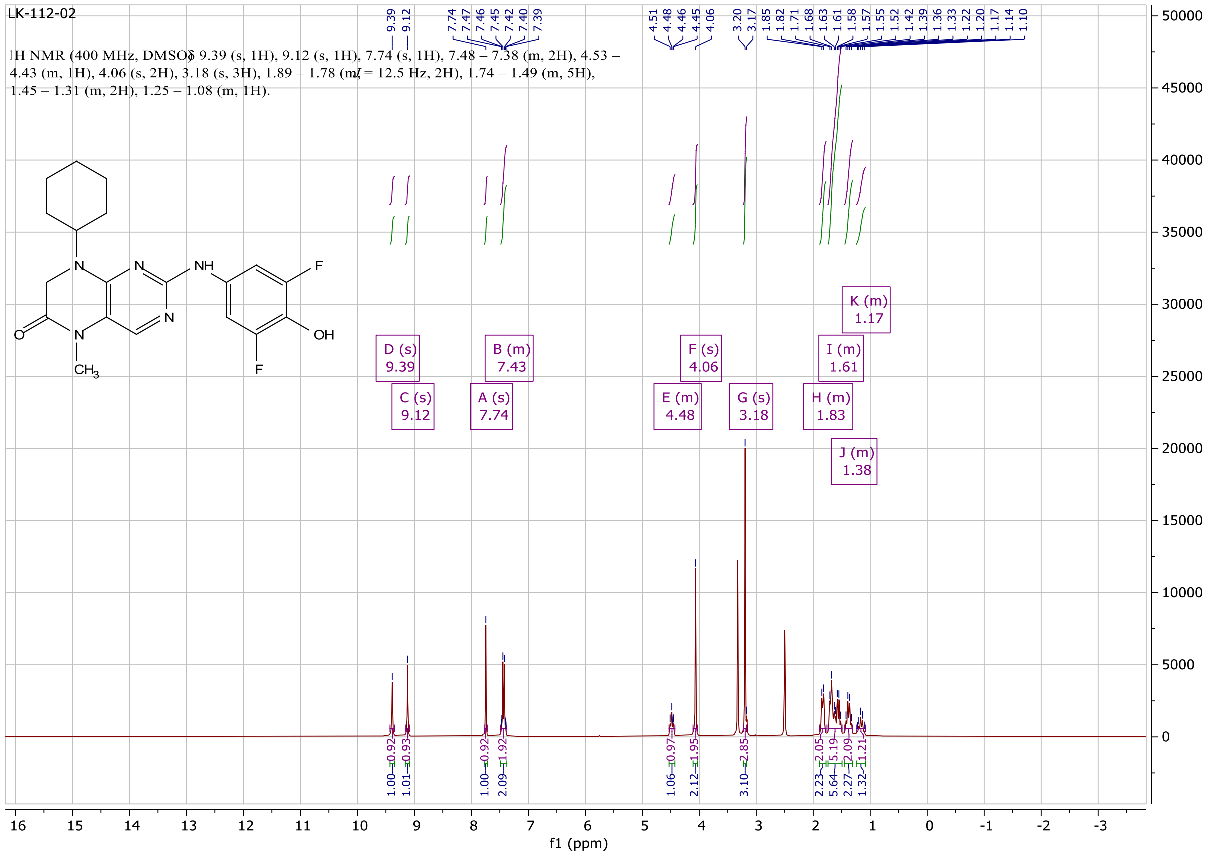

Figure S 25: ^{1}H-NMR spectrum of 23 (400 MHz, DMSO-d_6).

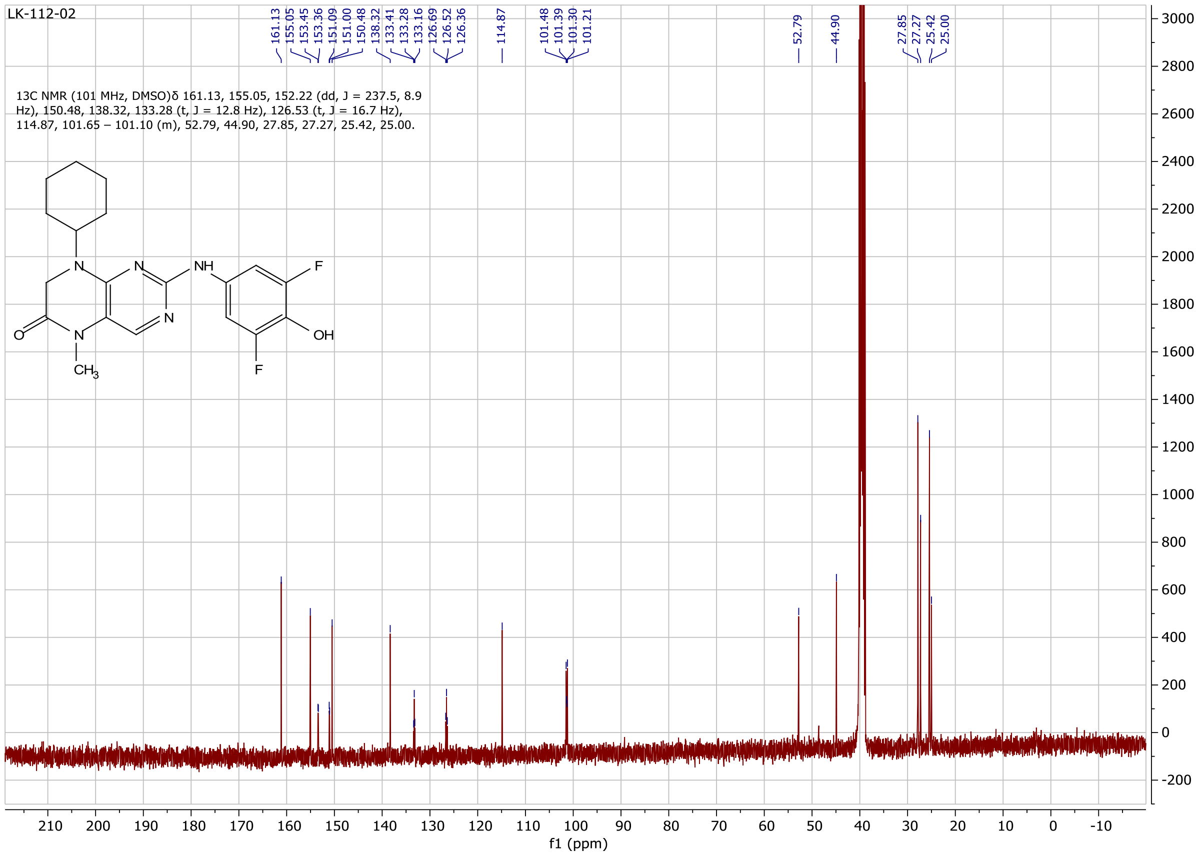

Figure S 26: ^{13}C-NMR spectrum of 23 (400 MHz, DMSO-d$_6$).

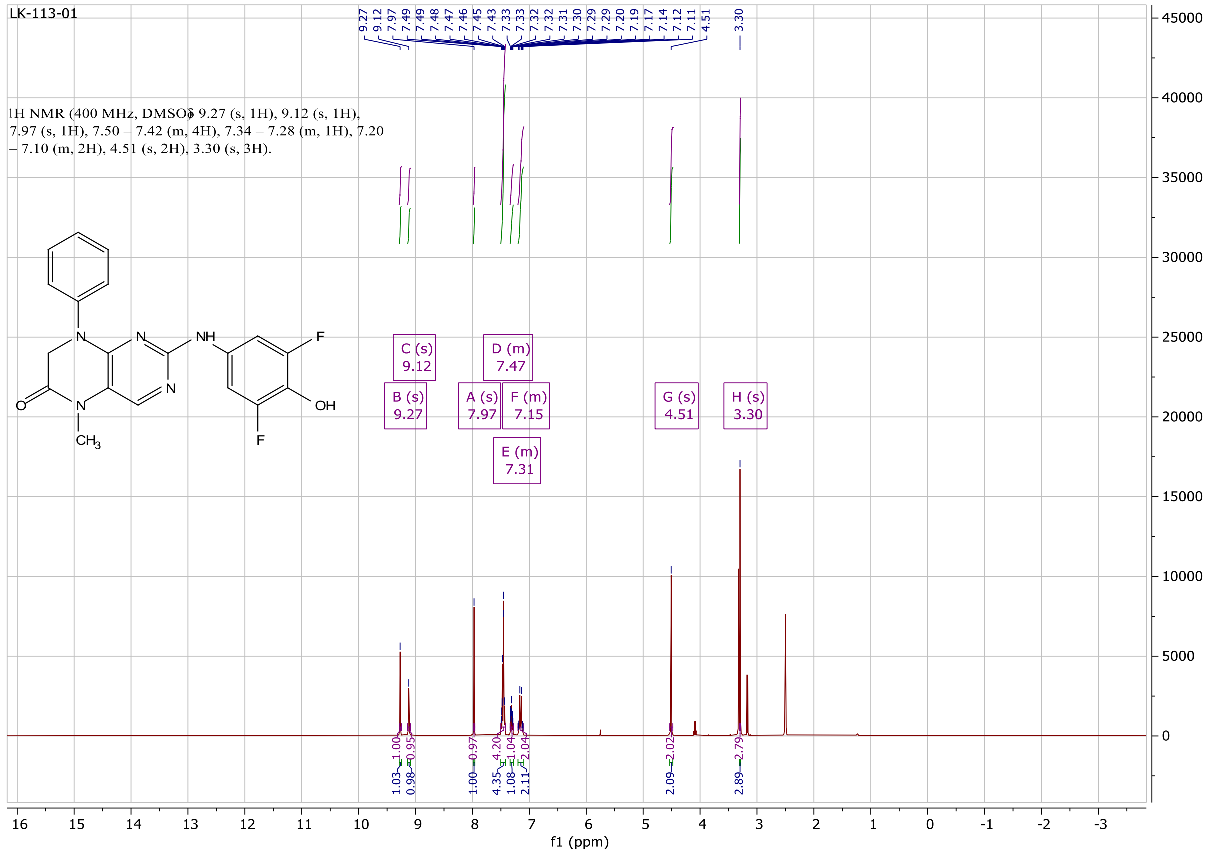

Figure S 27: ^{1}H-NMR spectrum of 24 (400 MHz, DMSO-d_6).

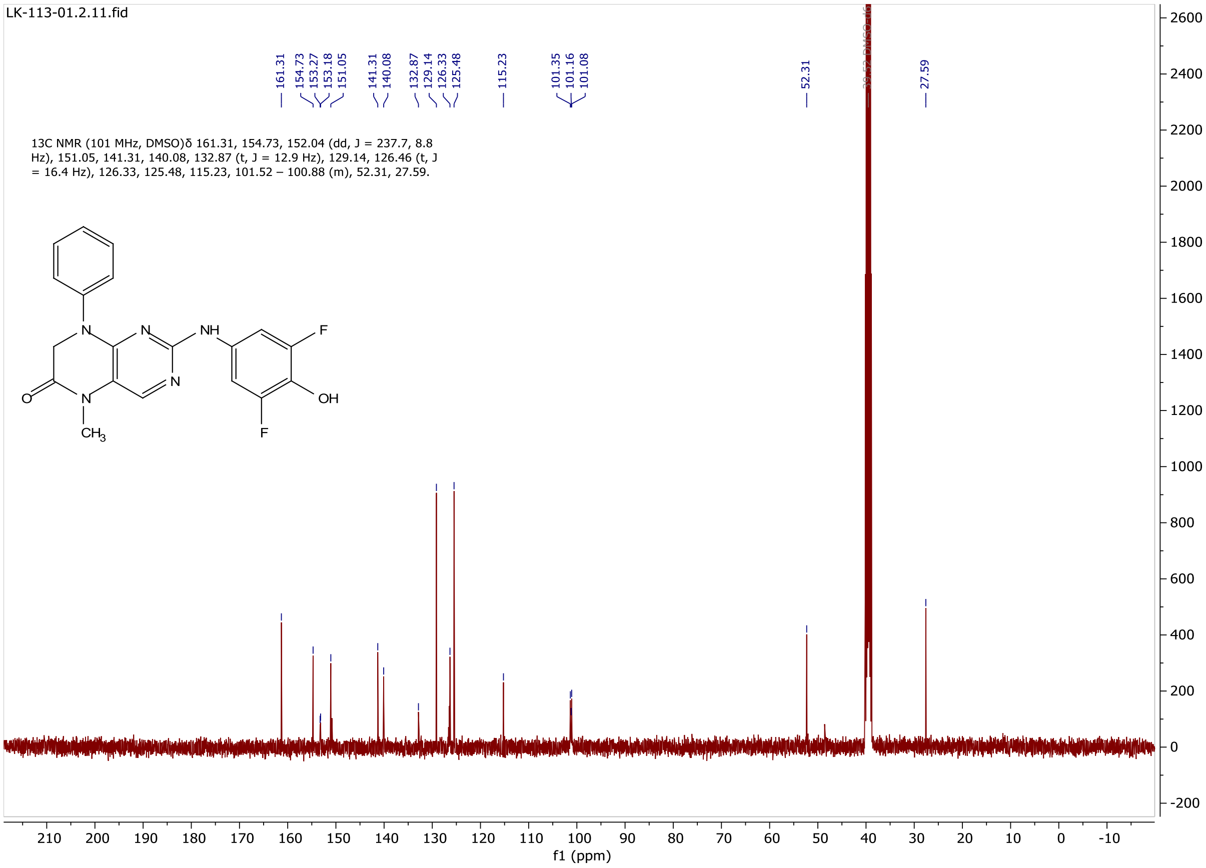

Figure S 28: ^{13}C-NMR spectrum of 24 (400 MHz, DMSO-d_6).

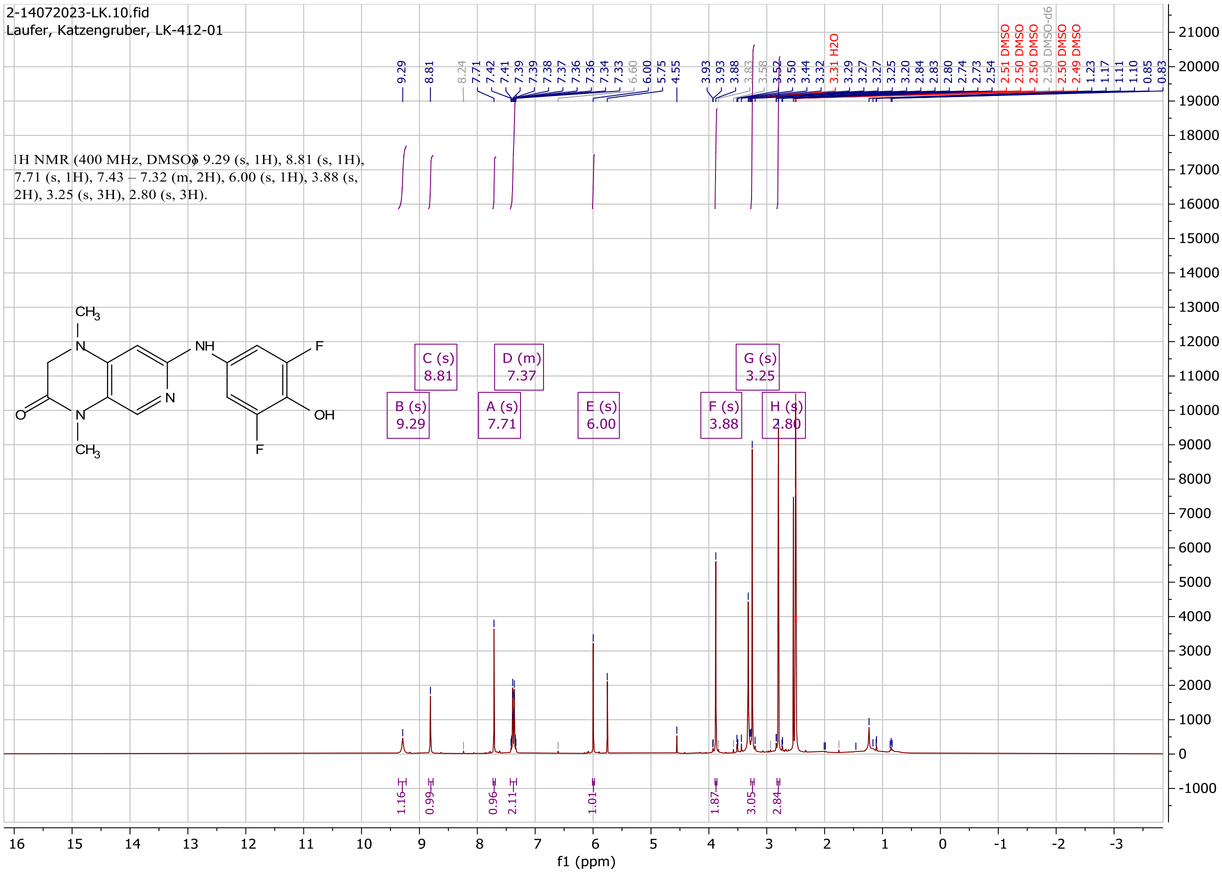

Figure S 29: ^{1}H-NMR spectrum of 25 (400 MHz, DMSO-d_6).

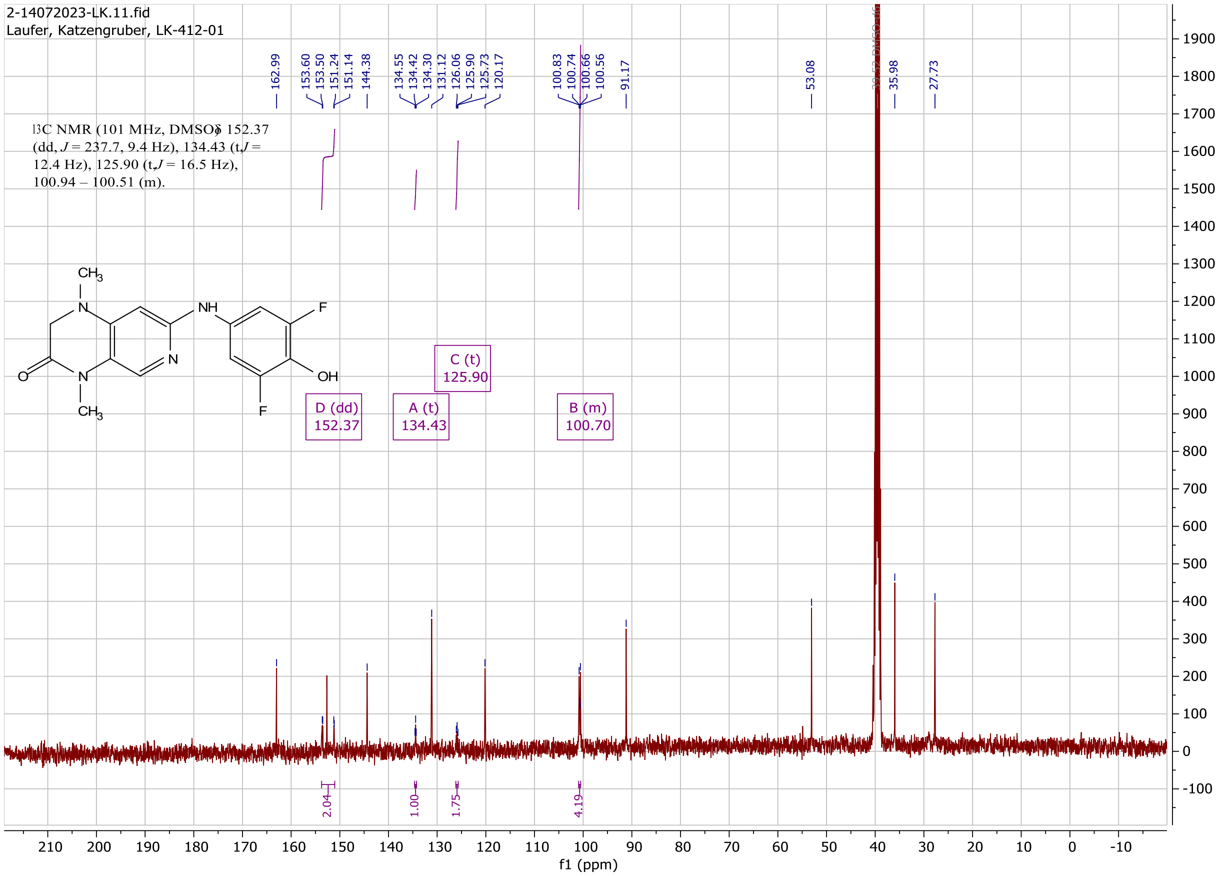

Figure S 30: ^{13}C-NMR spectrum of 25 (400 MHz, DMSO-d$_6$).

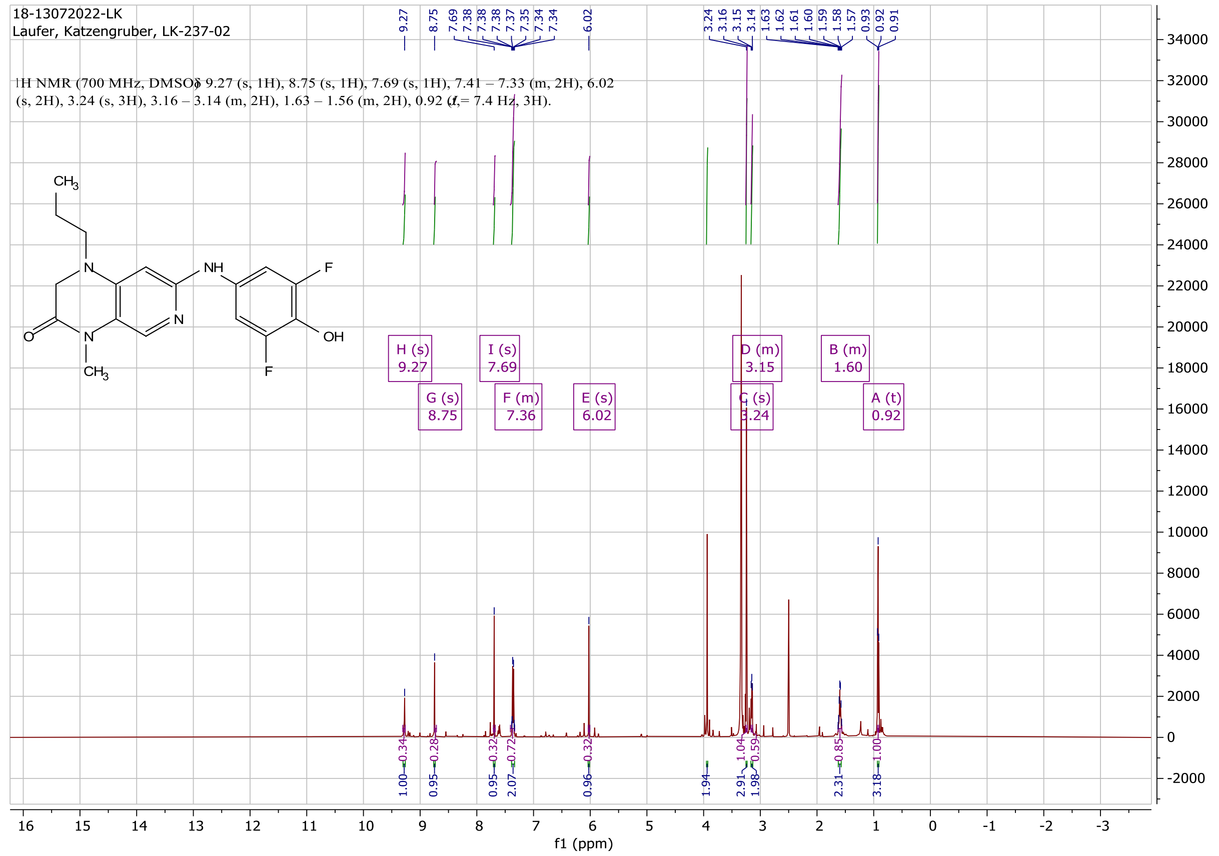

Figure S 31: ^{1}H-NMR spectrum of 26 (400 MHz, DMSO-d_6).

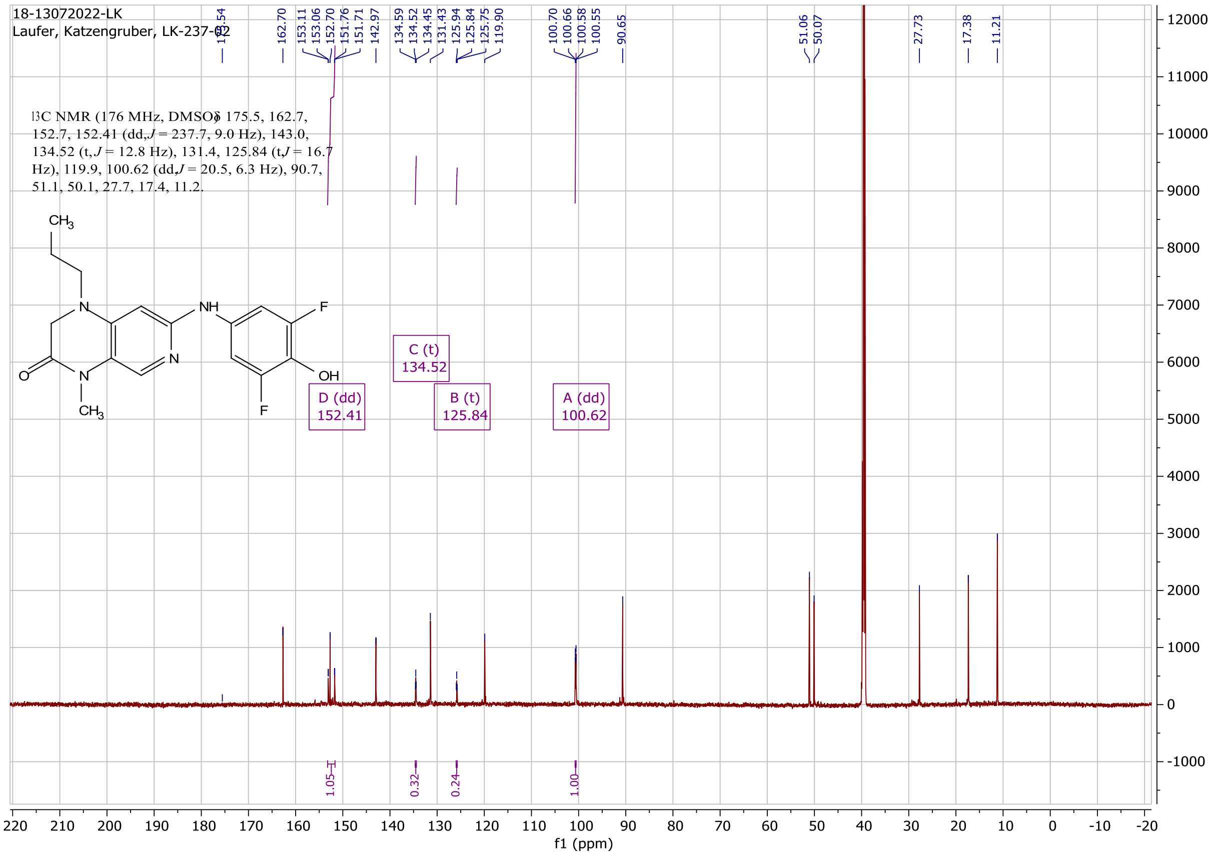

Figure S 32: ^{13}C-NMR spectrum of 26 (400 MHz, DMSO-d_6).

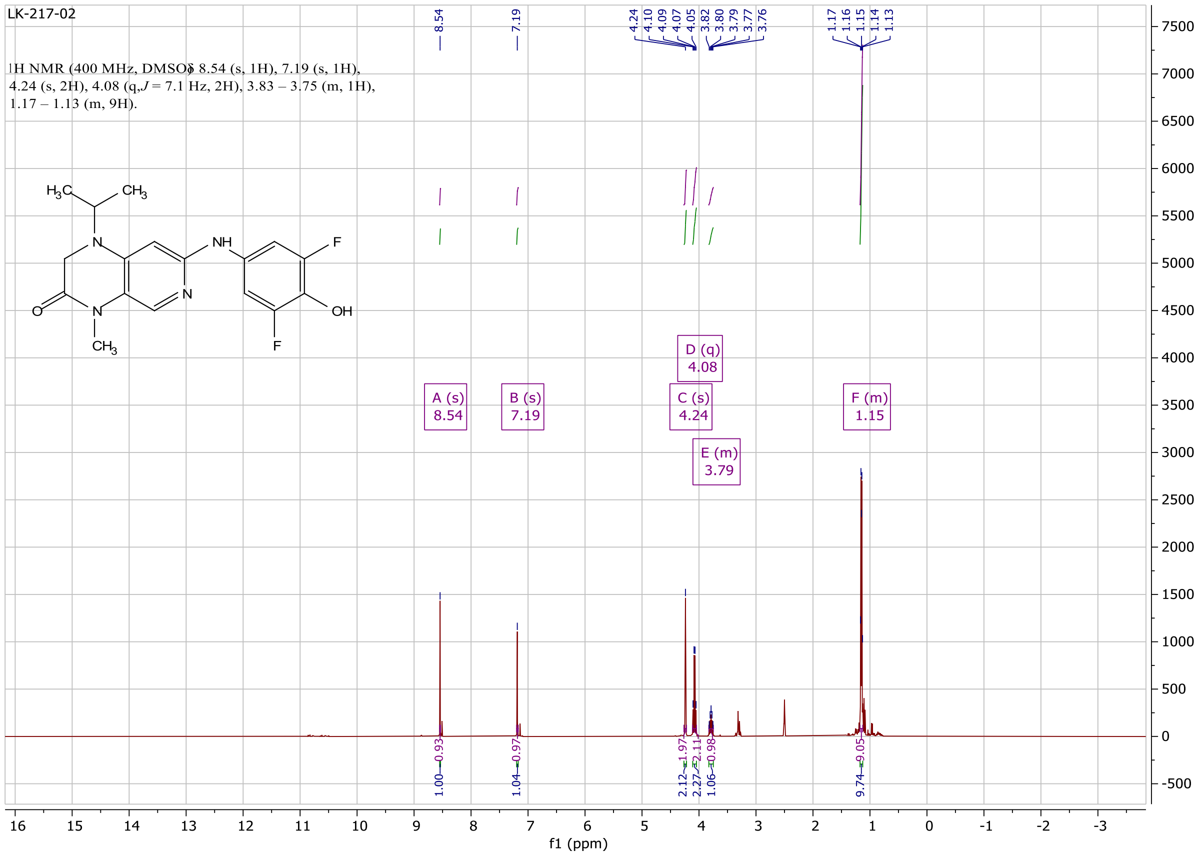

Figure S 33: ^{1}H-NMR spectrum of 27 (400 MHz, DMSO-d_6).

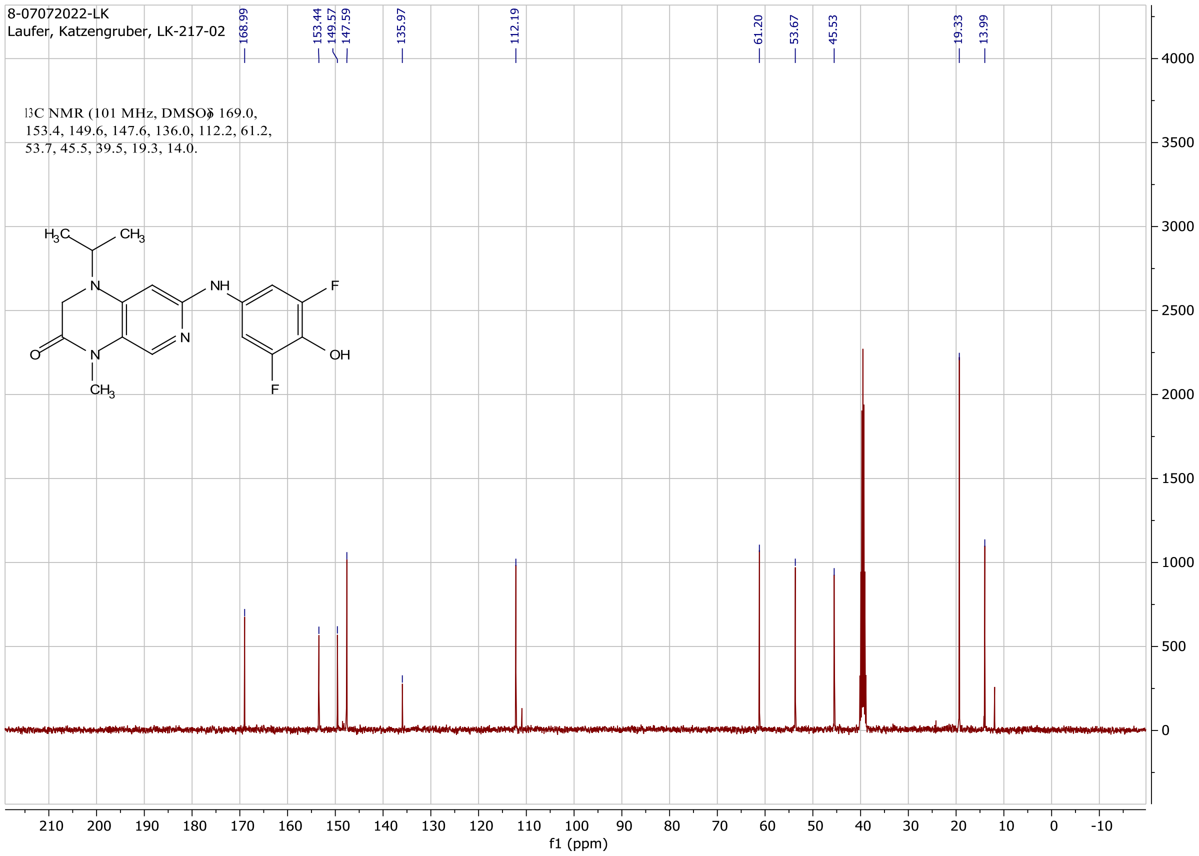

Figure S 34: ^{13}C-NMR spectrum of 27 (400 MHz, DMSO-d_6).

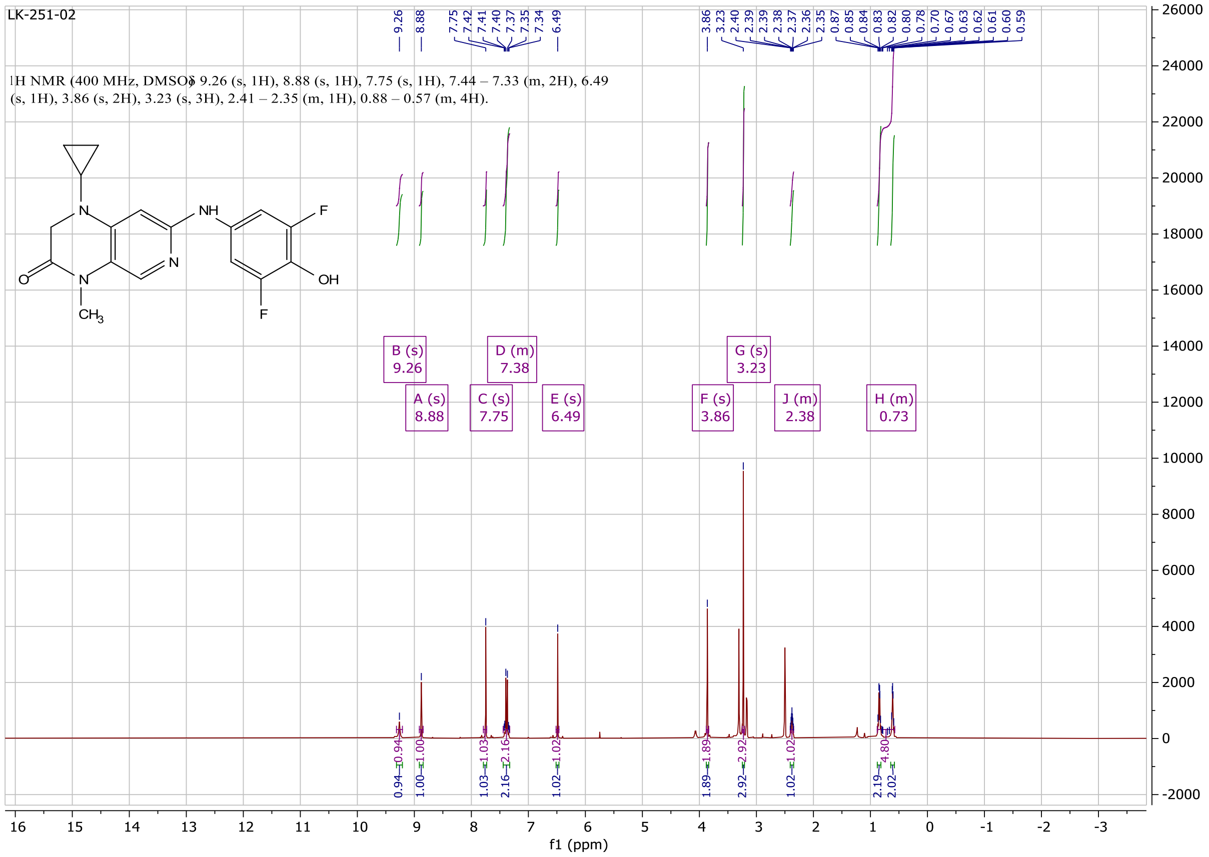

Figure S 35: ^{1}H-NMR spectrum of 28 (400 MHz, DMSO-d_6).

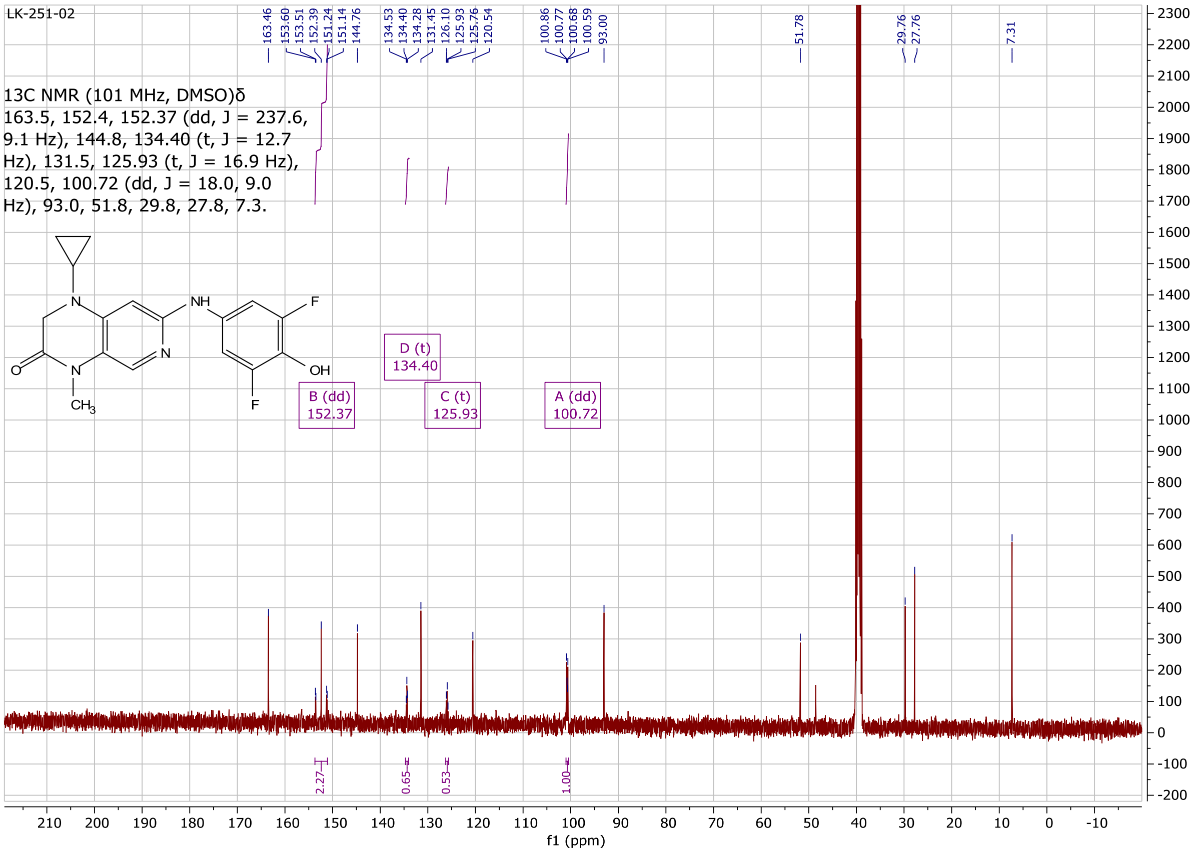

Figure S 36: ^{13}C-NMR spectrum of 28 (400 MHz, DMSO-d_6).

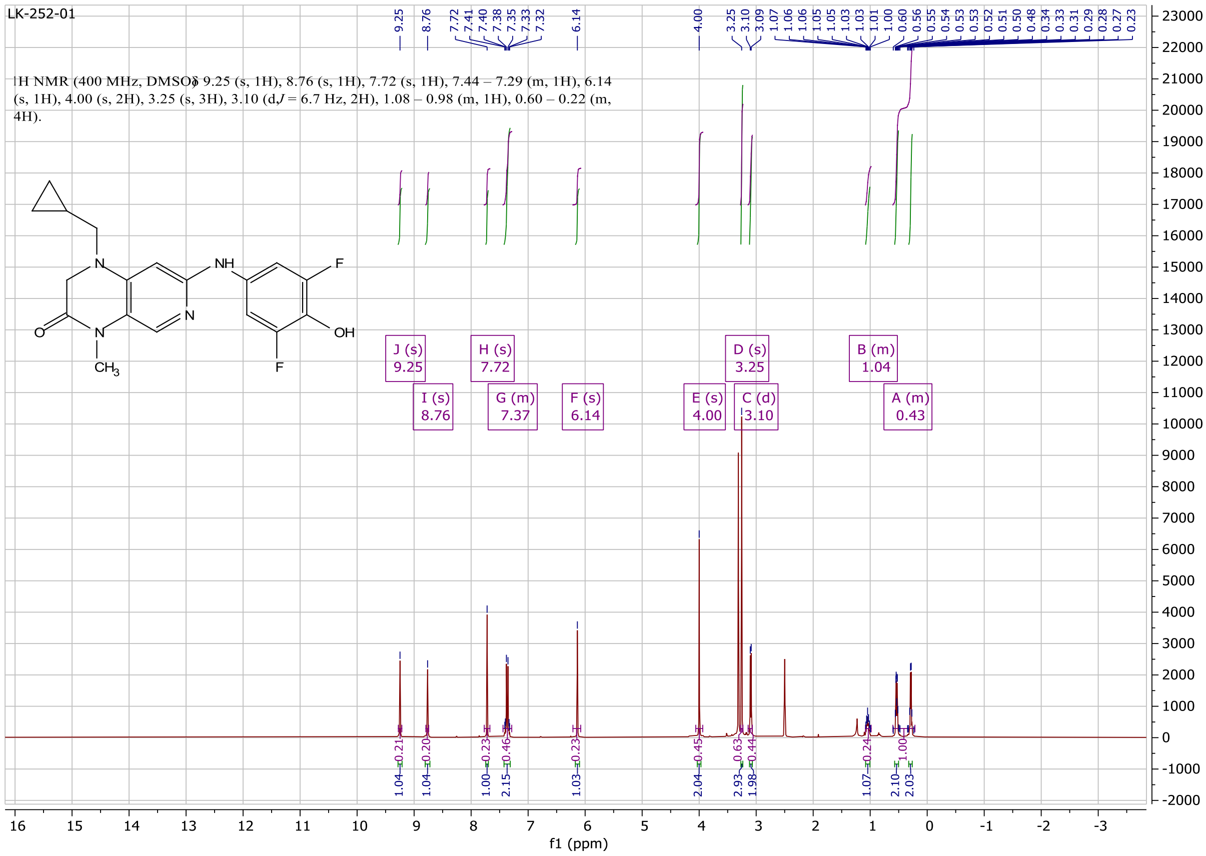

Figure S 37: ^{1}H-NMR spectrum of 29 (400 MHz, DMSO-d$_6$).

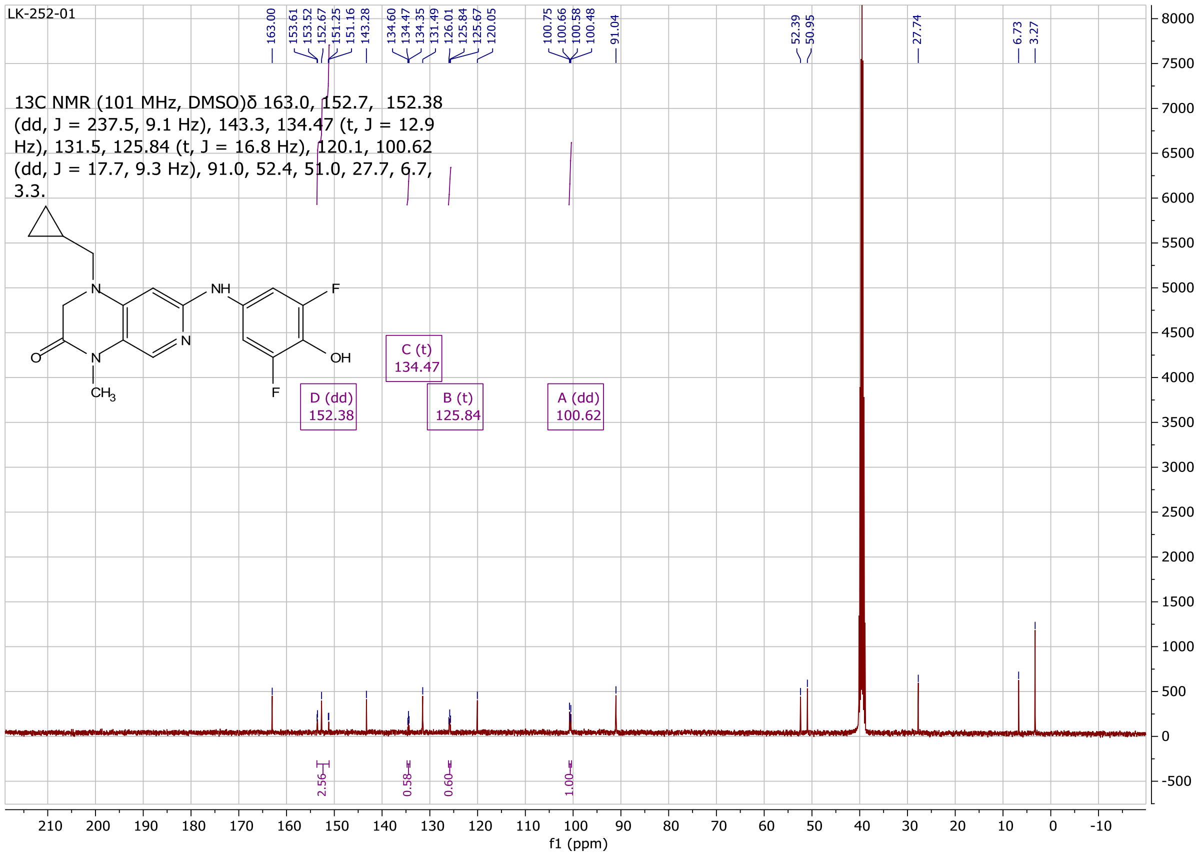

Figure S 38: ^{13}C-NMR spectrum of 29 (400 MHz, DMSO-d_6).

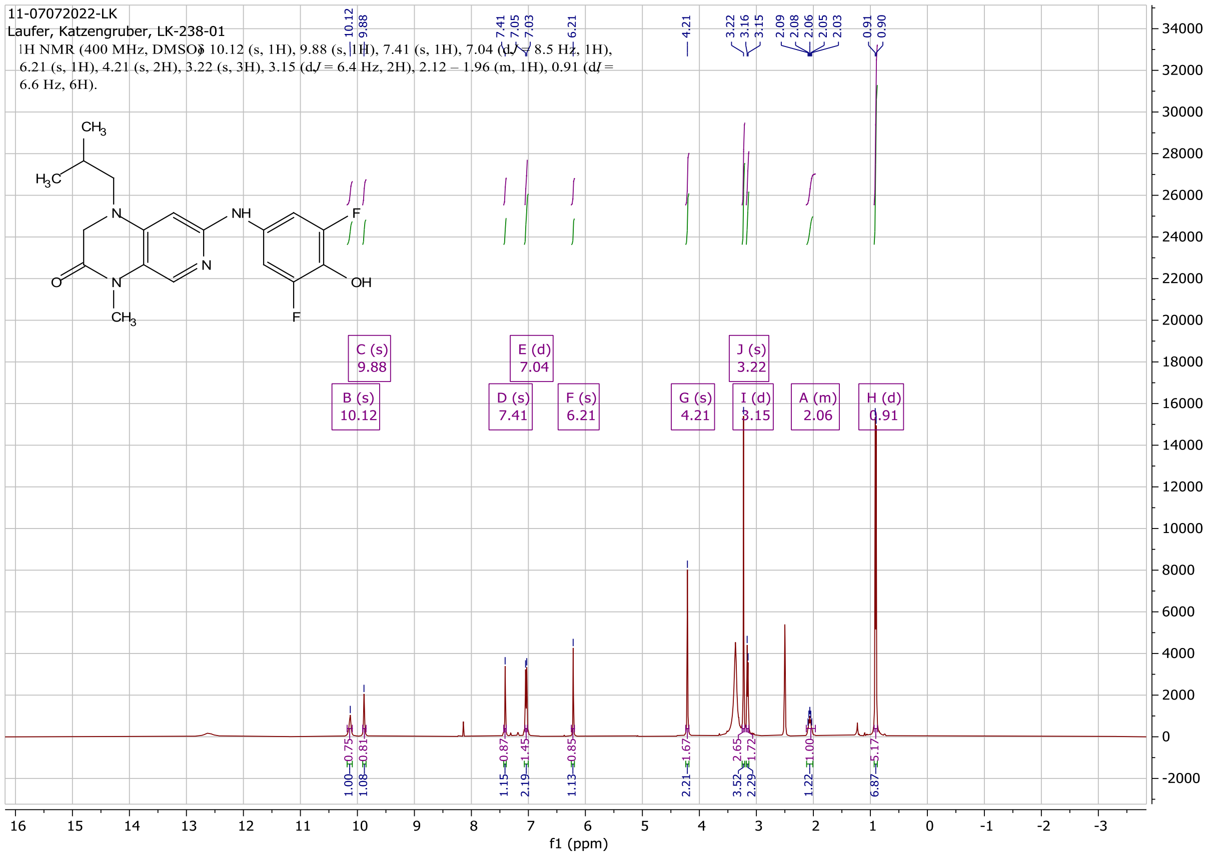

Figure S 39: ^{1}H-NMR spectrum of 30 (400 MHz, DMSO-d$_6$).

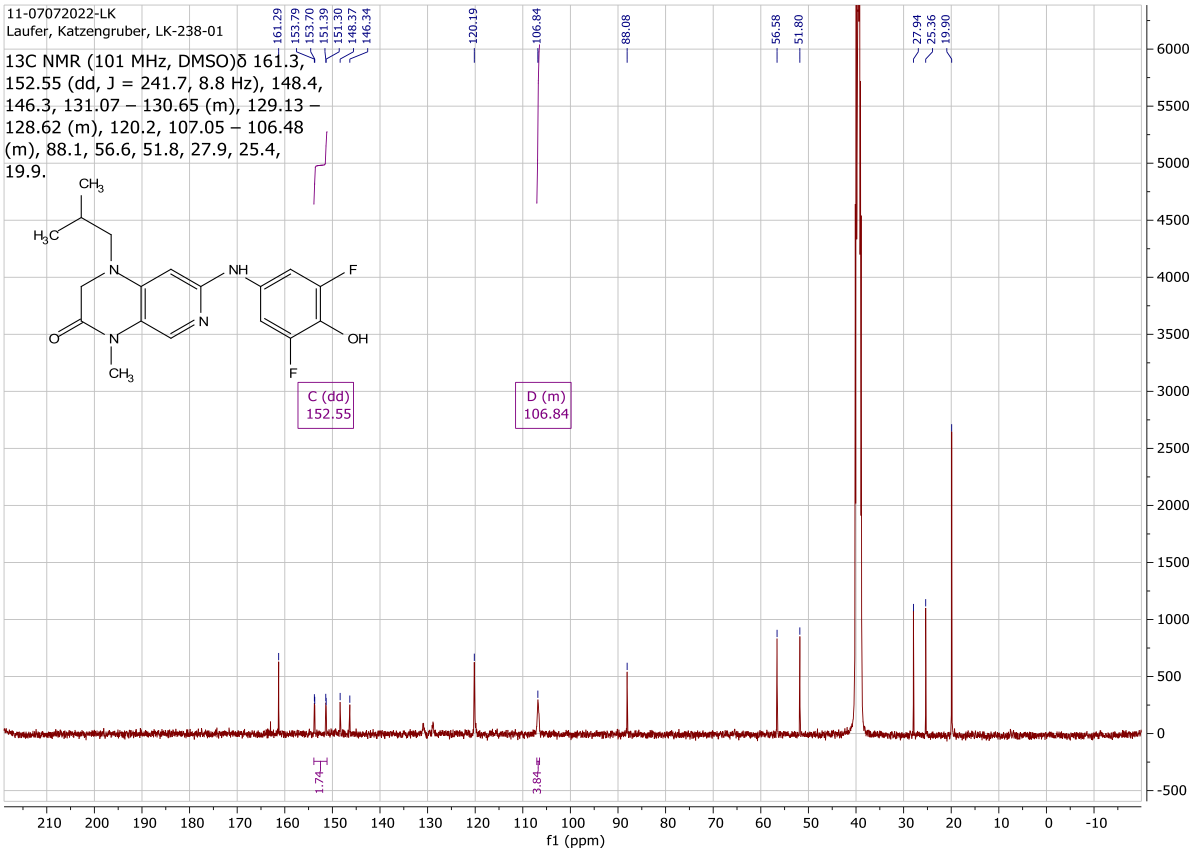

Figure S 40: ^{13}C-NMR spectrum of 30 (400 MHz, DMSO-d_6).

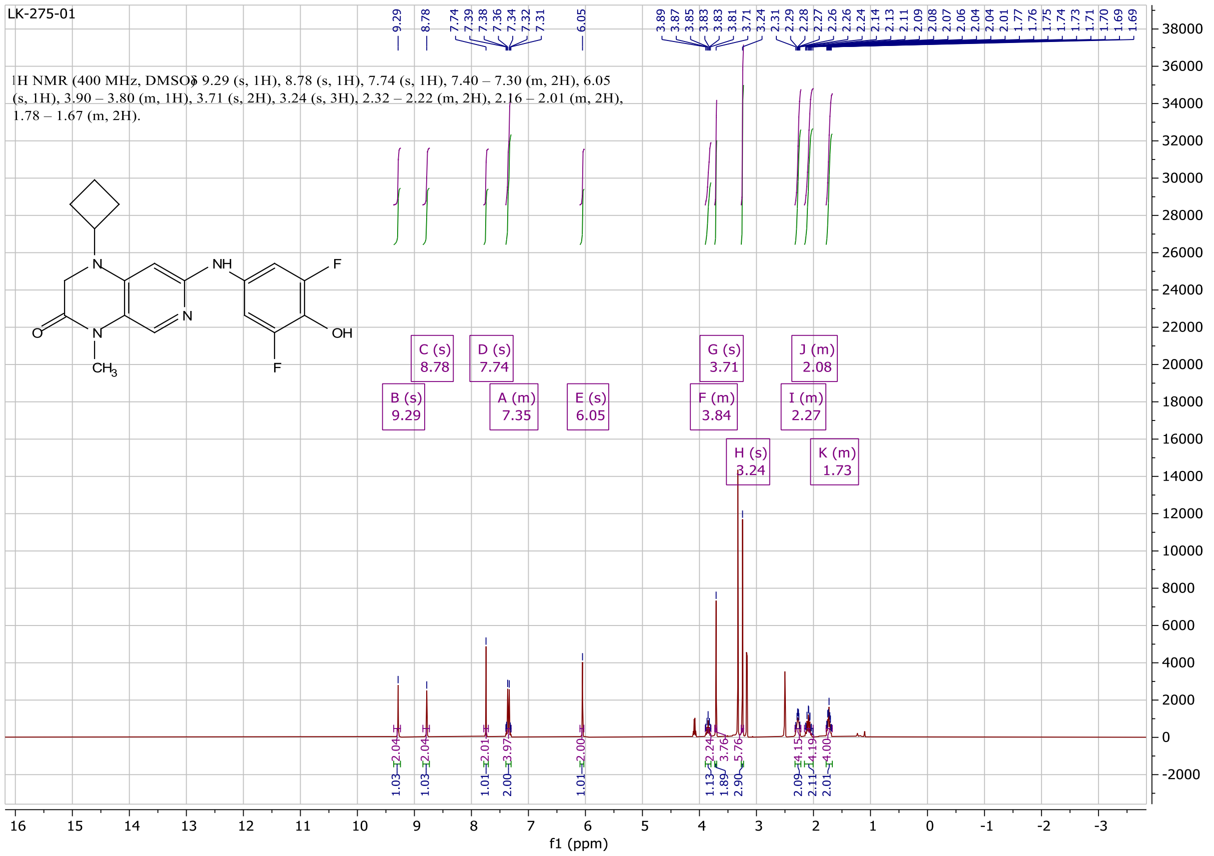

Figure S 41: ^{1}H-NMR spectrum of 31 (400 MHz, DMSO-d_6).

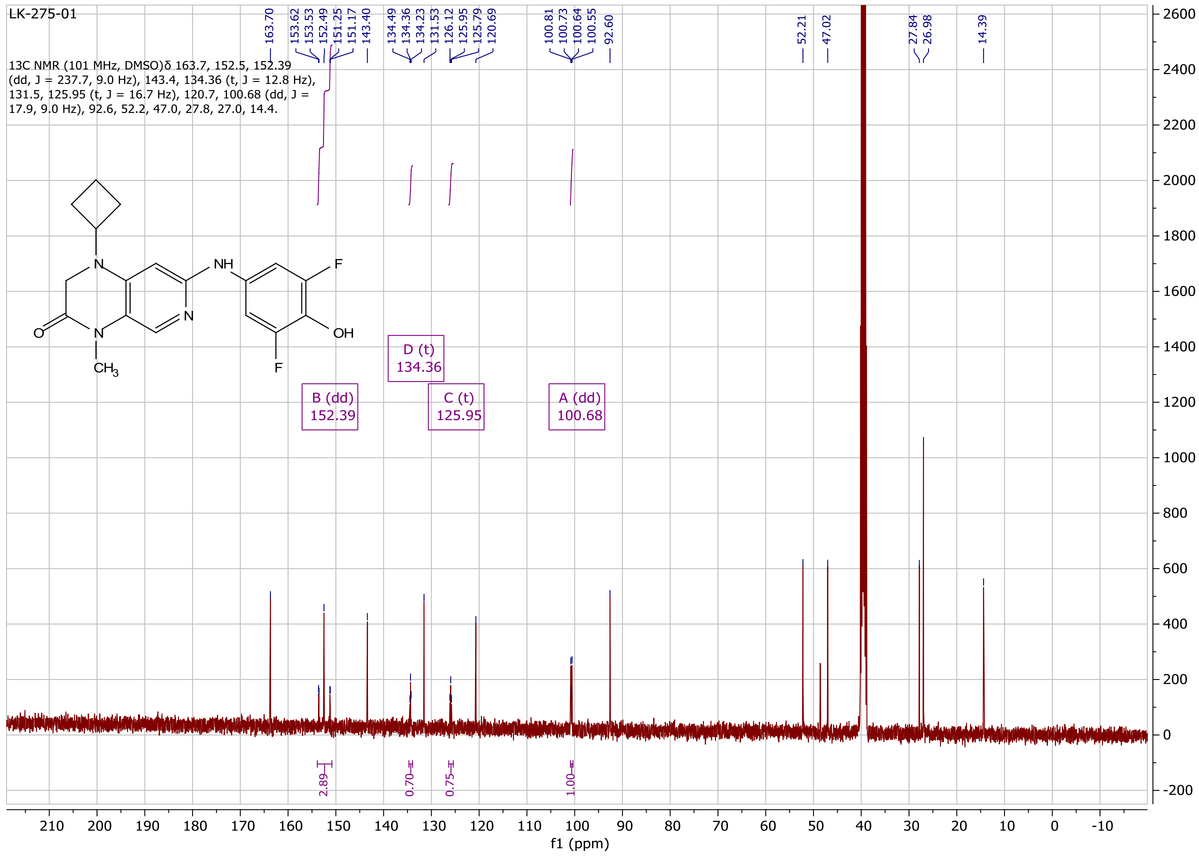

Figure S 42: ^{13}C-NMR spectrum of 31 (400 MHz, DMSO-d_6).

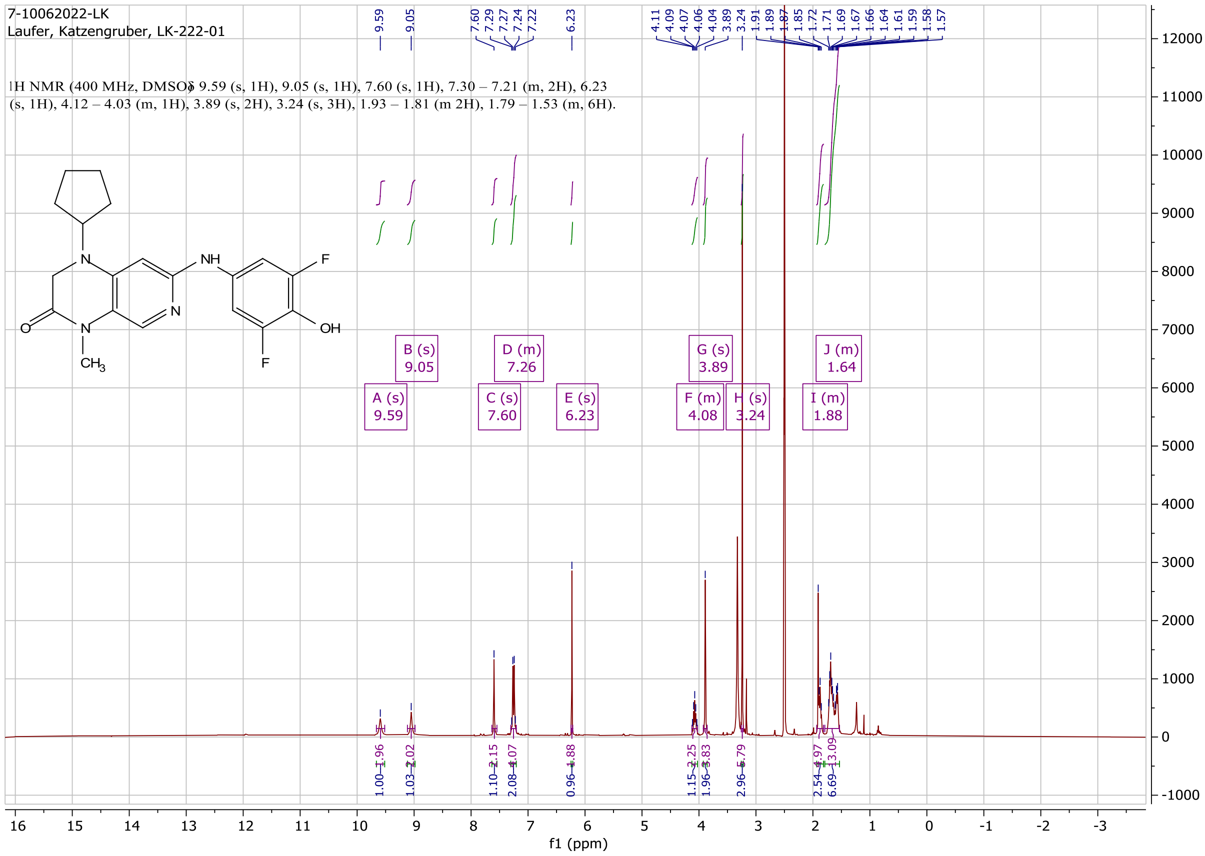

Figure S 43: ^{1}H-NMR spectrum of 32 (400 MHz, DMSO-d_6).

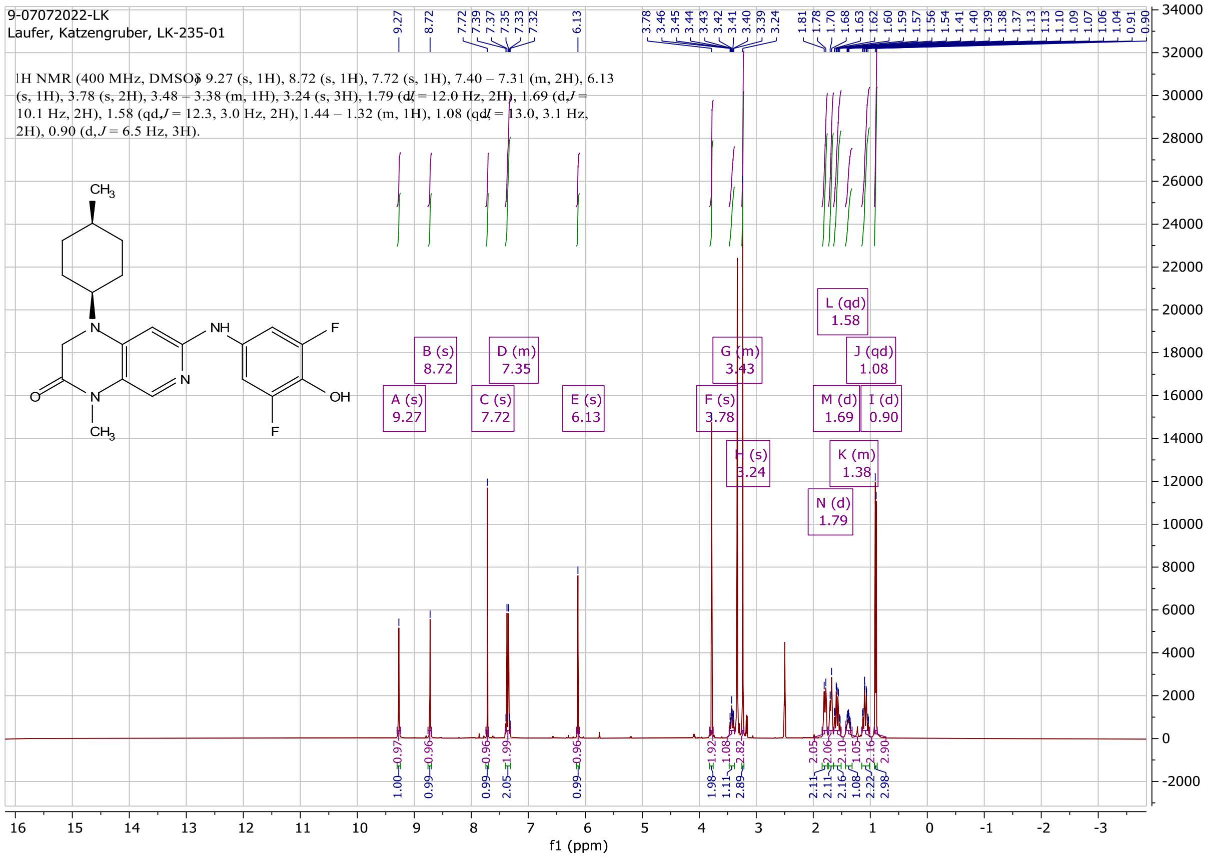

Figure S 44: ^{1}H-NMR spectrum of 33 (400 MHz, DMSO-d$_6$).

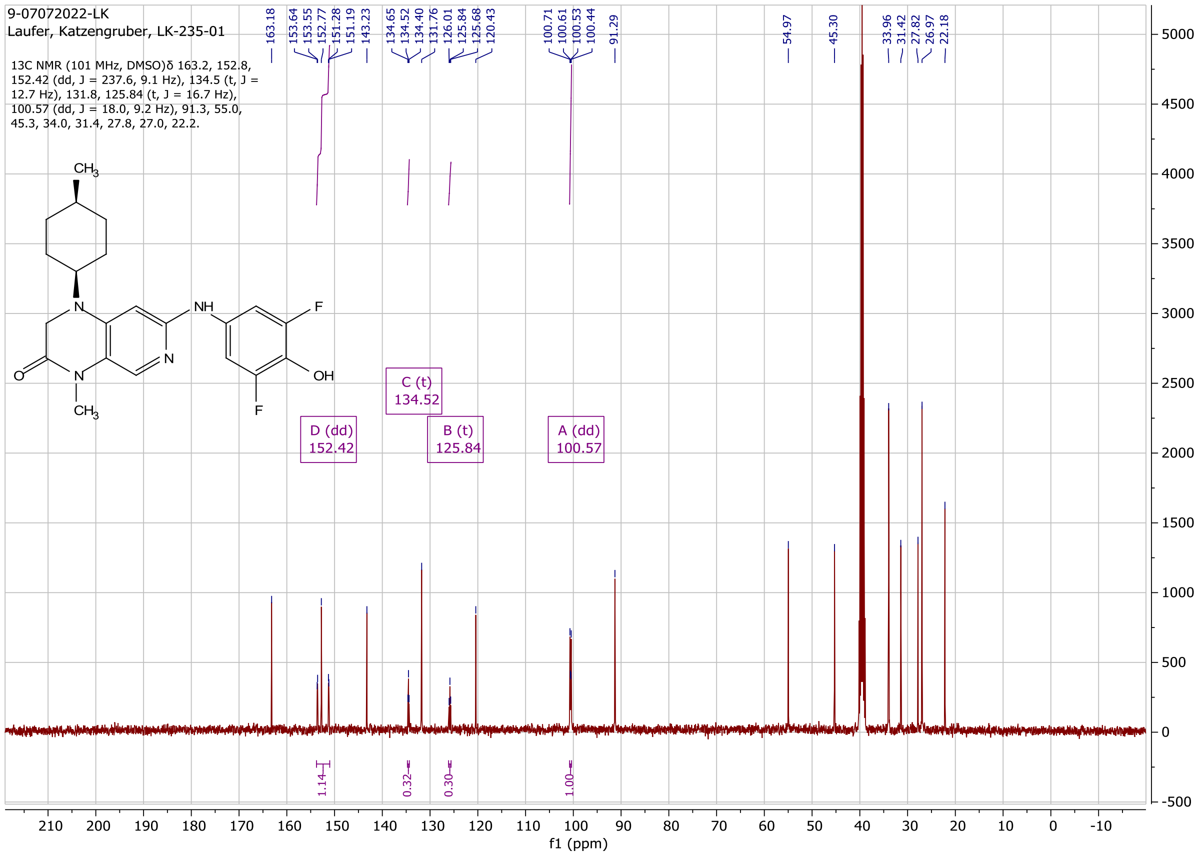

Figure S 45: ^{13}C-NMR spectrum of 33 (400 MHz, DMSO-d_6).

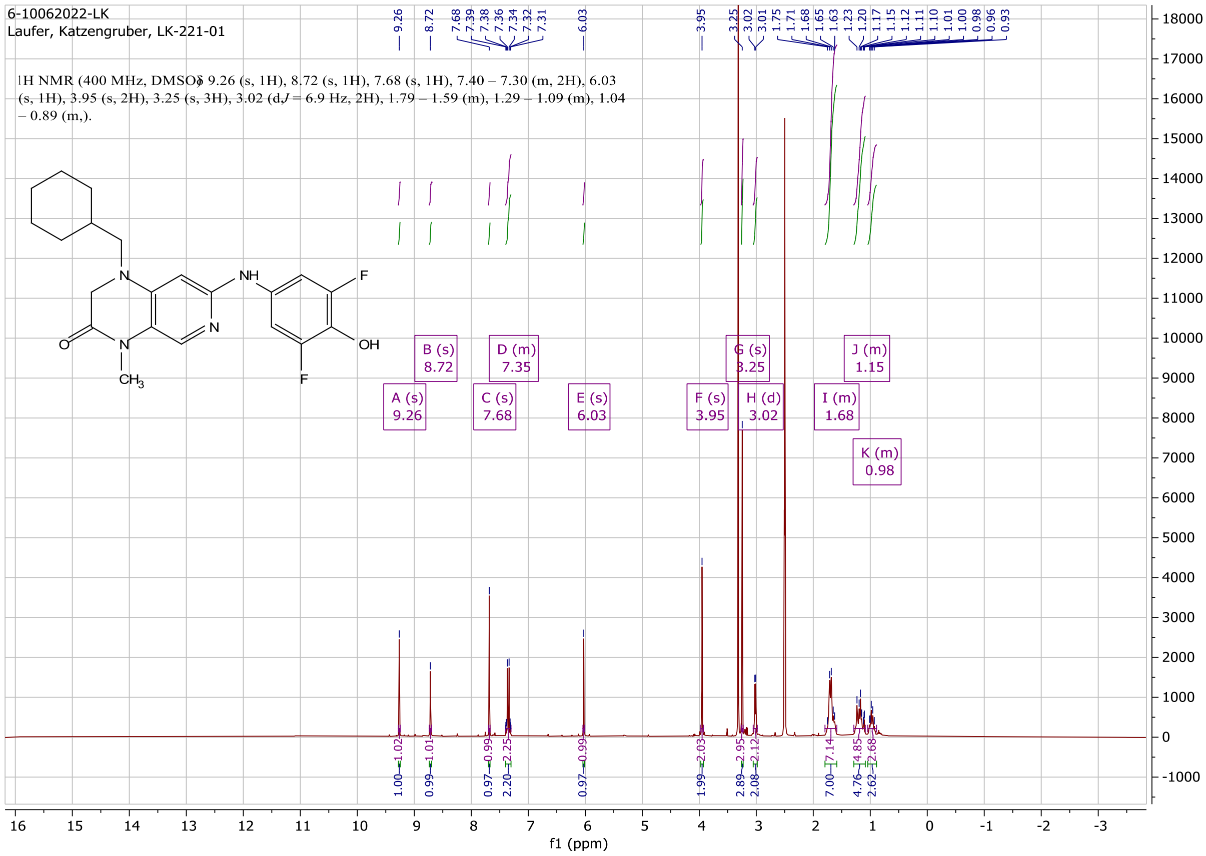

Figure S 46: ^{1}H-NMR spectrum of 34 (400 MHz, DMSO-d_6).

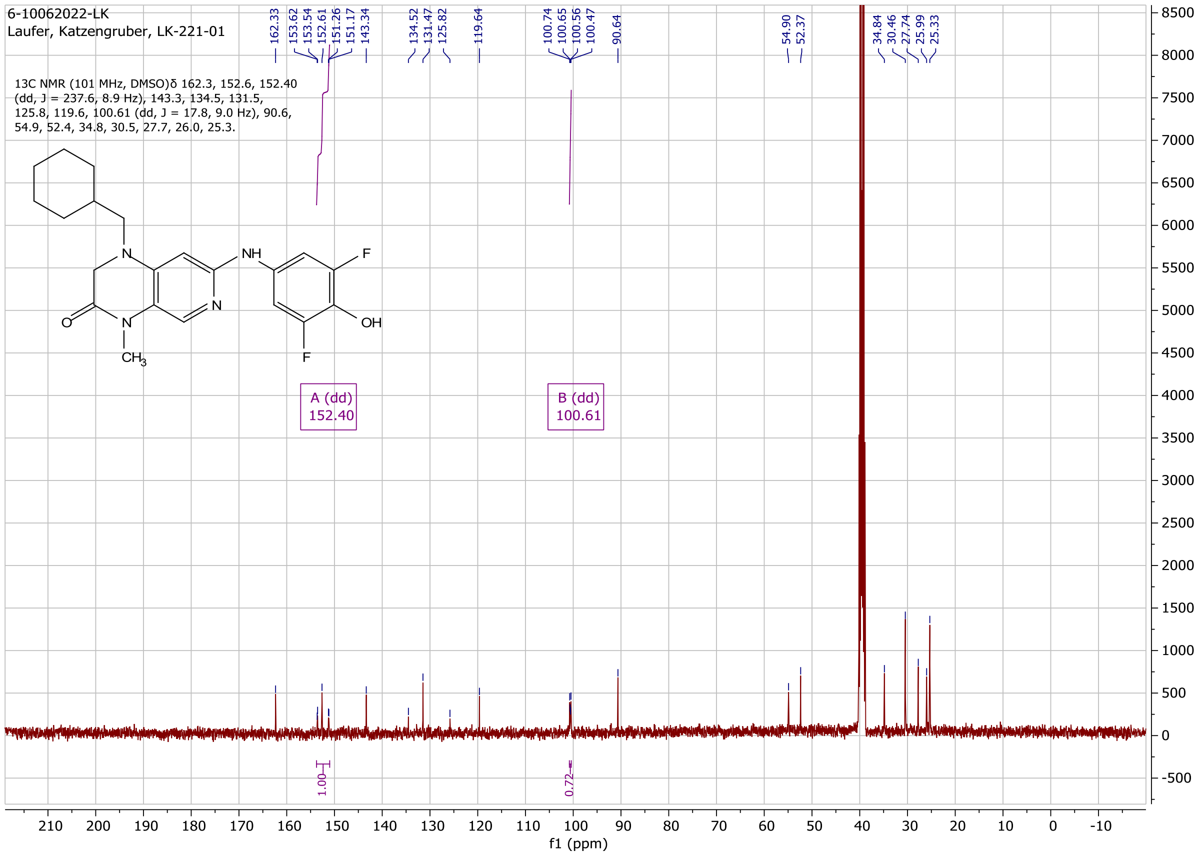

Figure S 47: ^{13}C-NMR spectrum of 34 (400 MHz, DMSO-d_6).

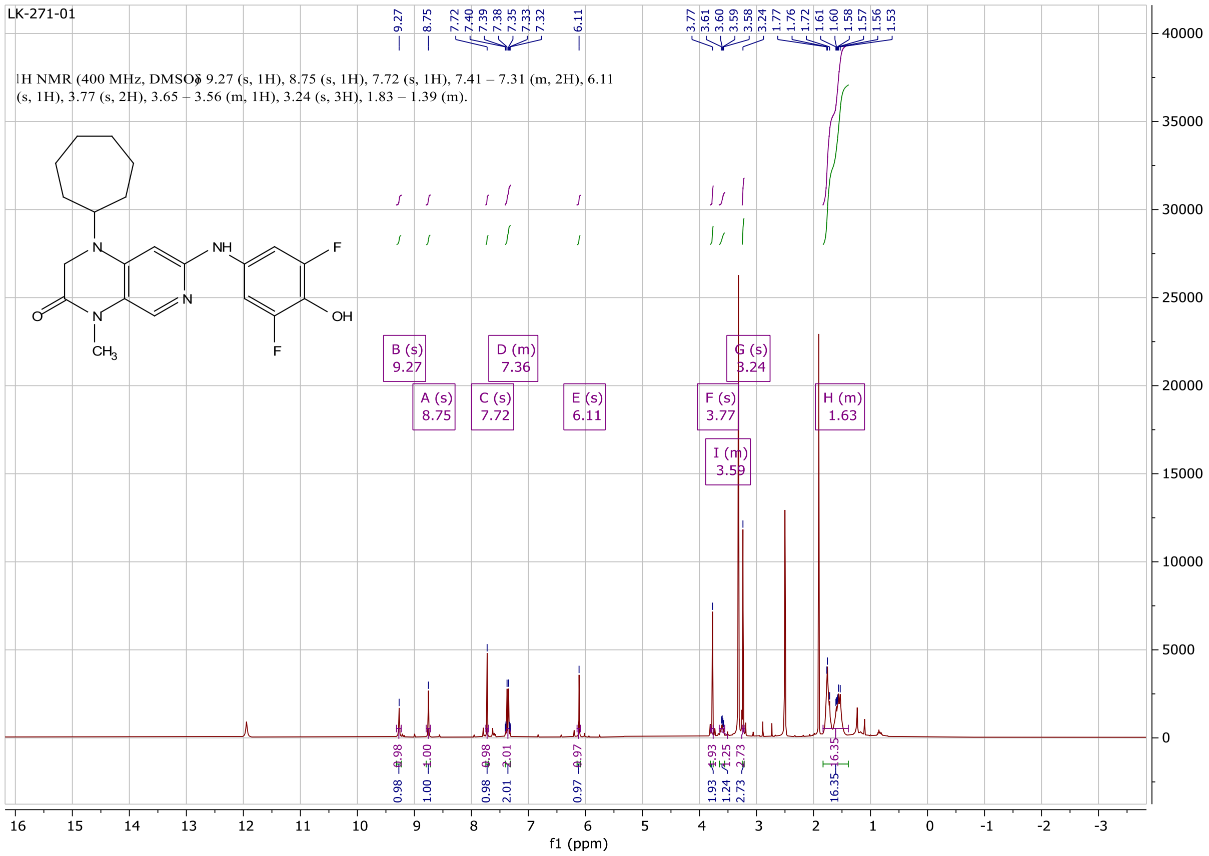

Figure S 48: ^{1}H-NMR spectrum of 35 (400 MHz, DMSO-d_6).

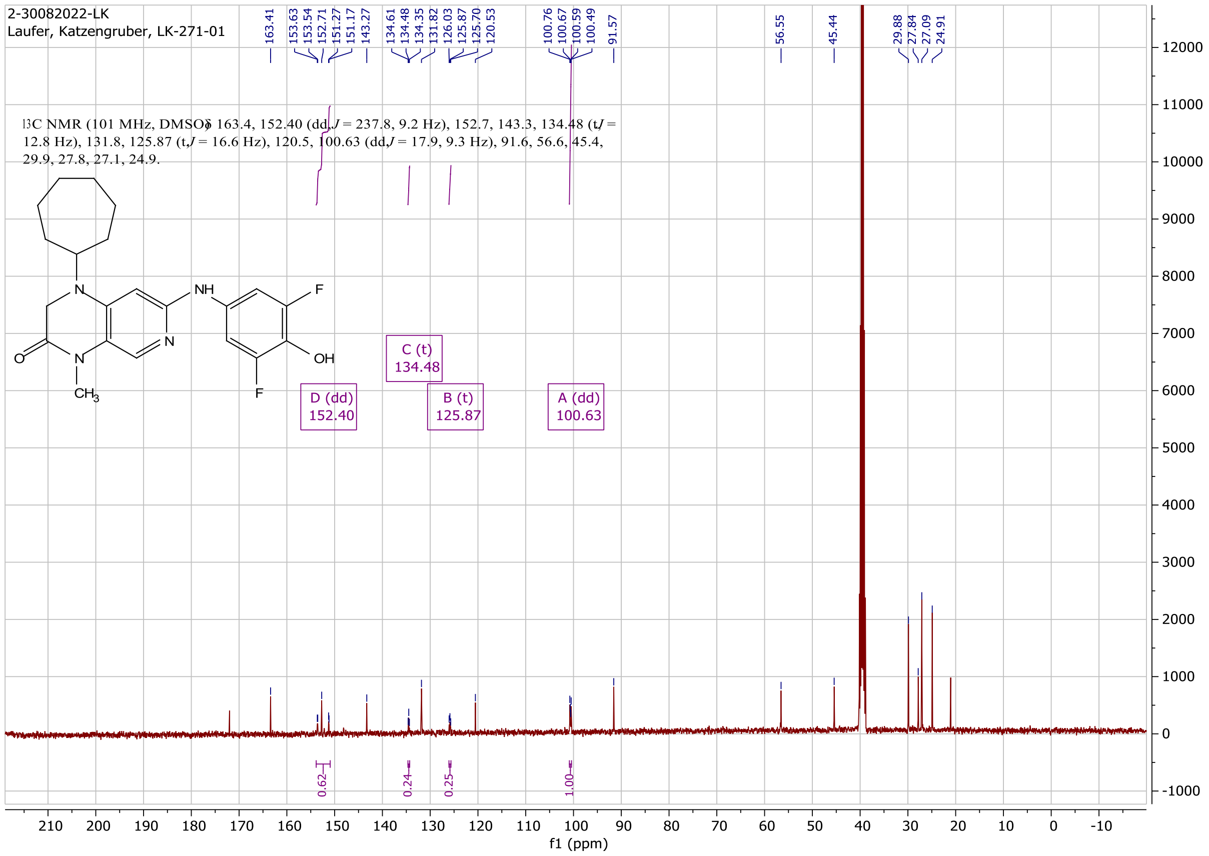

Figure S 49: ^{13}C-NMR spectrum of 35 (400 MHz, DMSO-d_6).

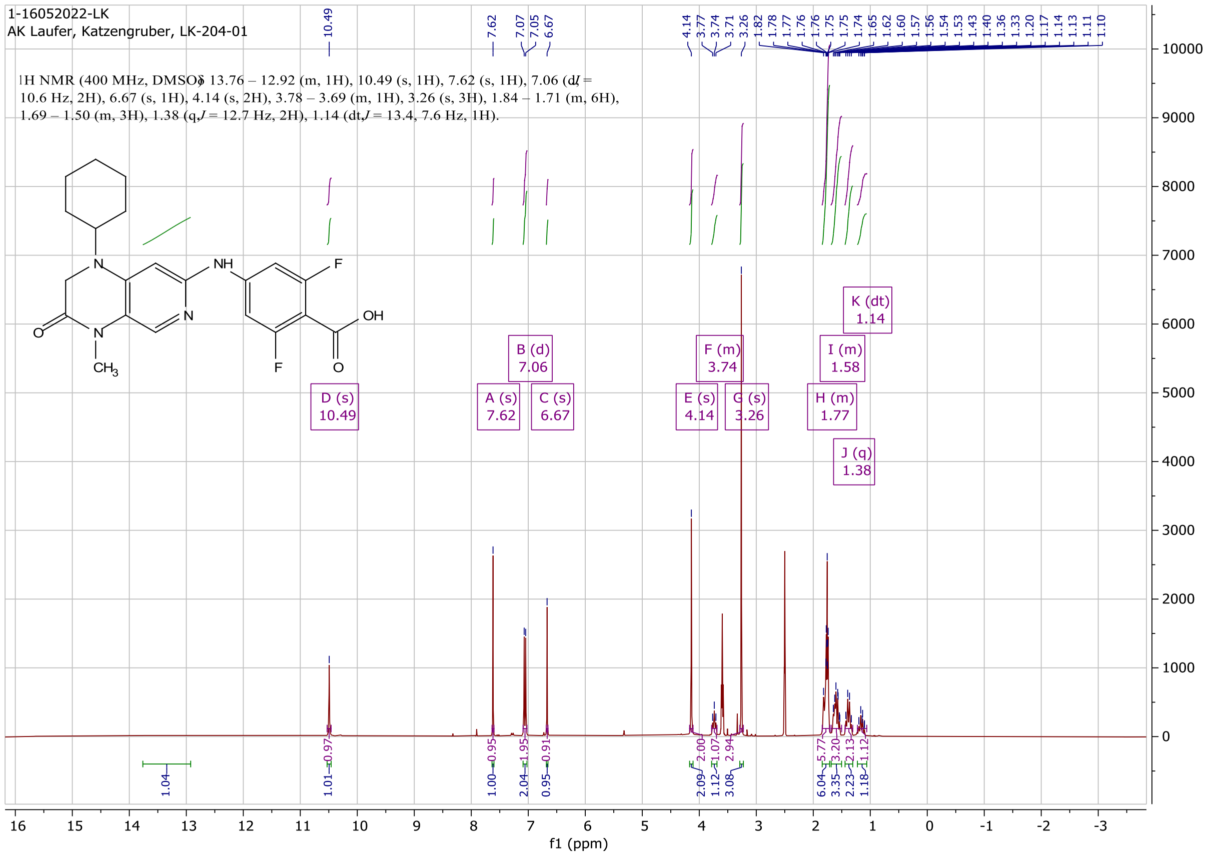

Figure S 50: ^{1}H-NMR spectrum of 36 (400 MHz, DMSO-d_6).

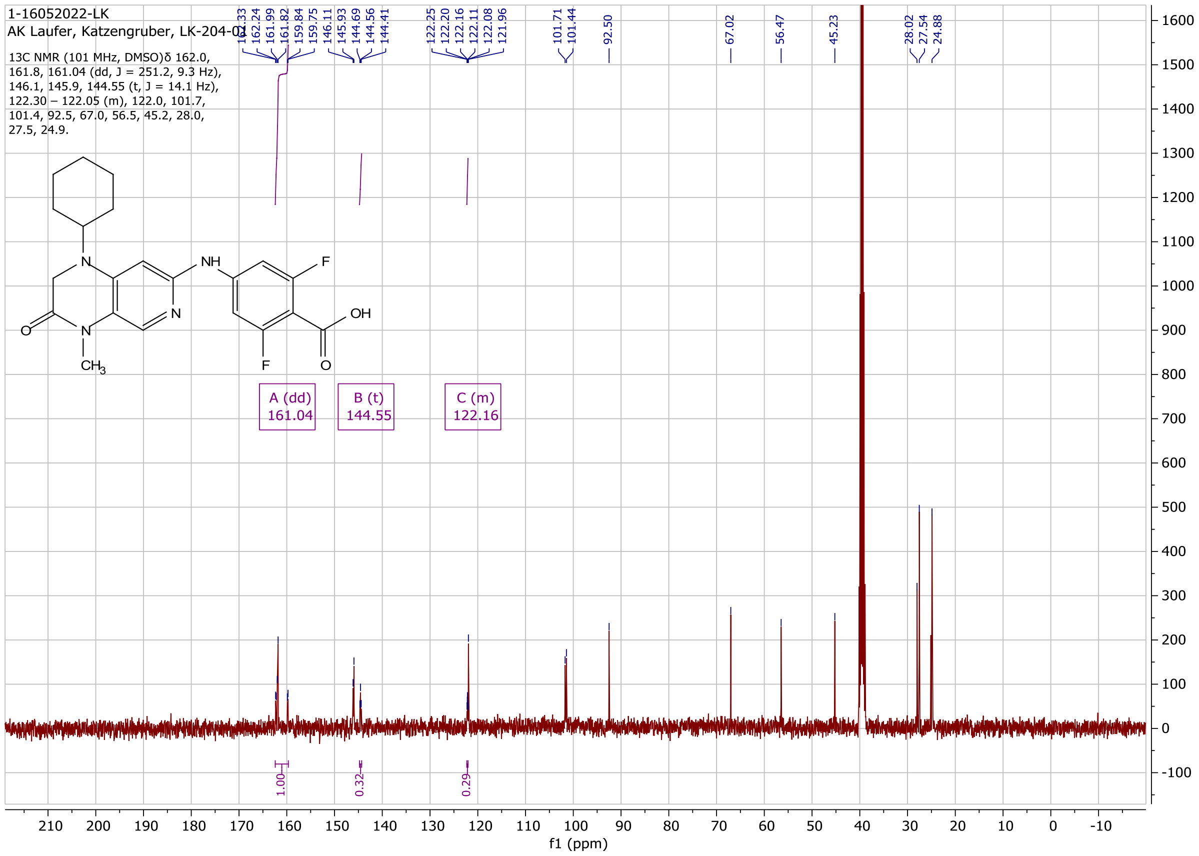

Figure S 51: ^{13}C-NMR spectrum of 36 (400 MHz, DMSO-d_6).

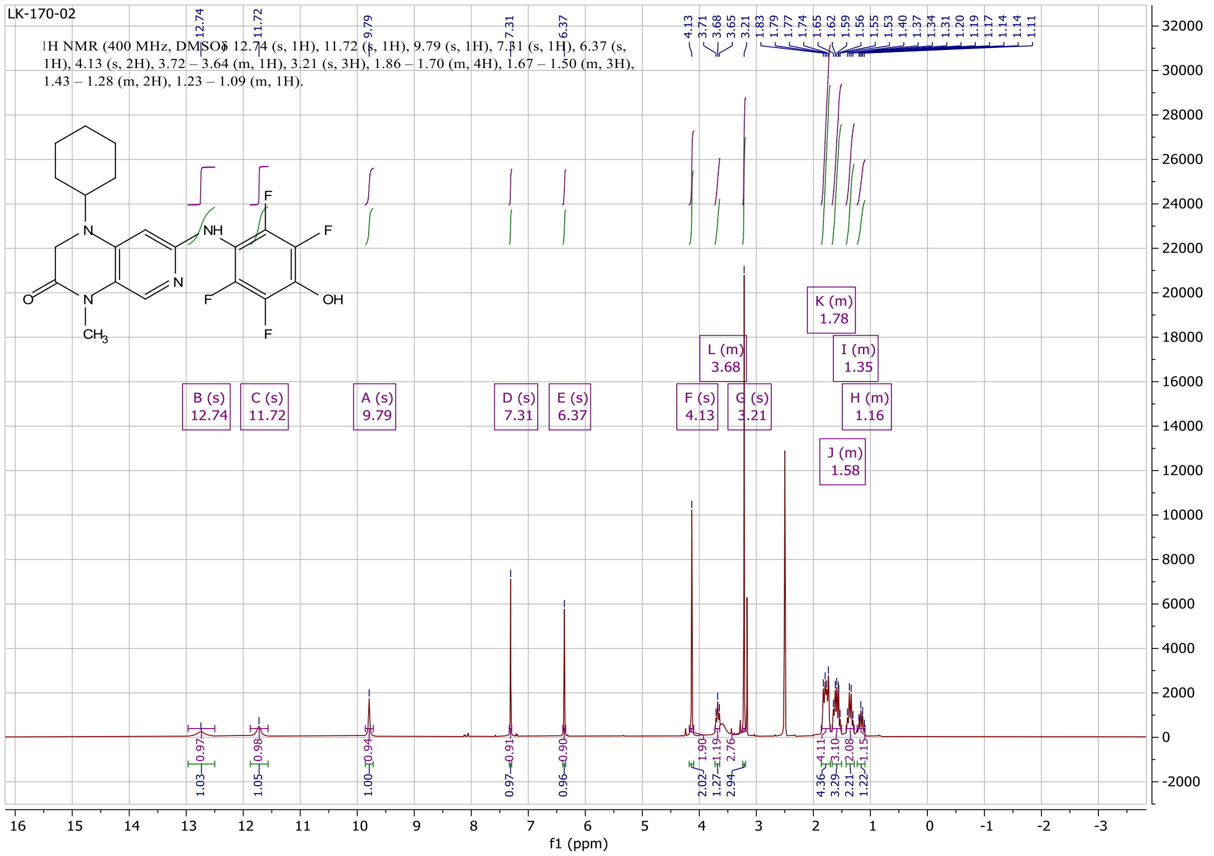

Figure S 52: ^{1}H-NMR spectrum of 37 (400 MHz, DMSO-d_6).

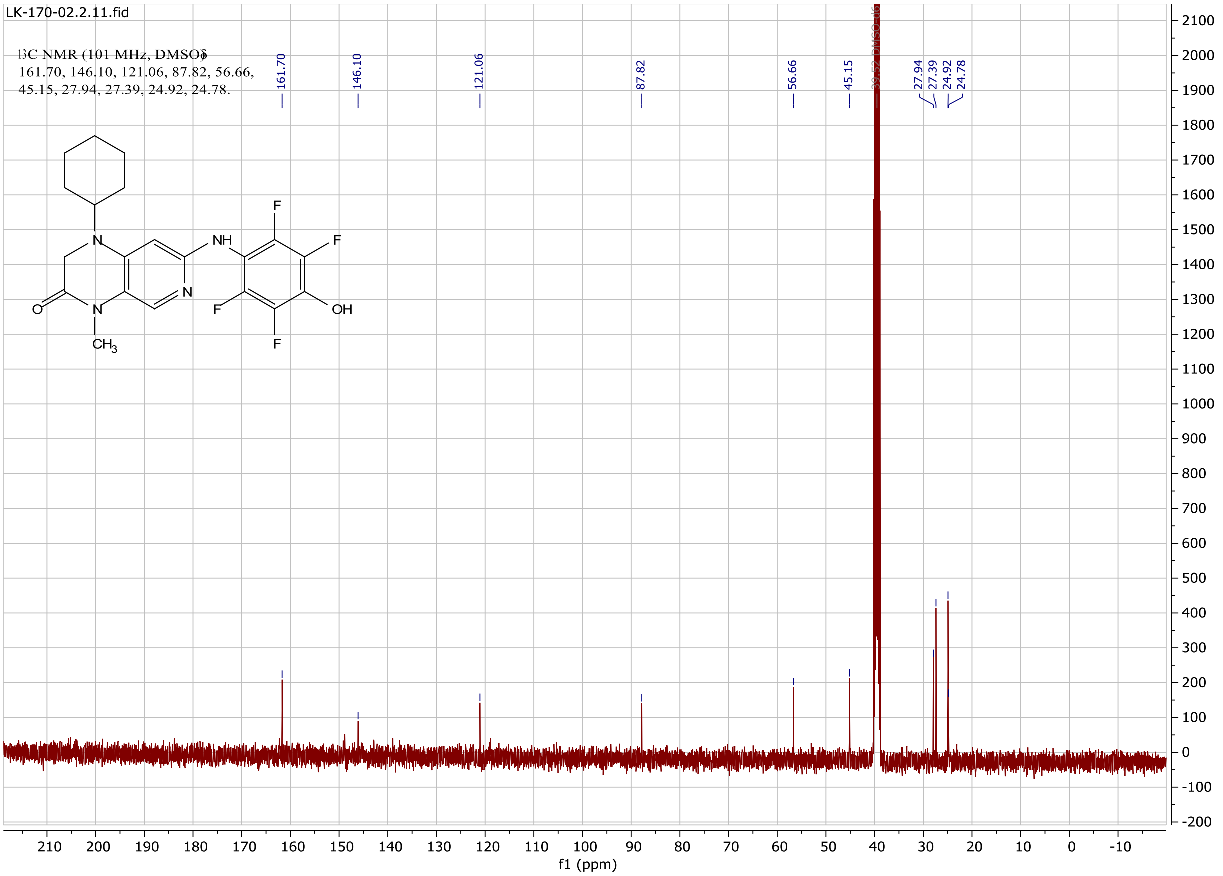

Figure S 53: ^{13}C-NMR spectrum of 37 (400 MHz, DMSO-d_6).

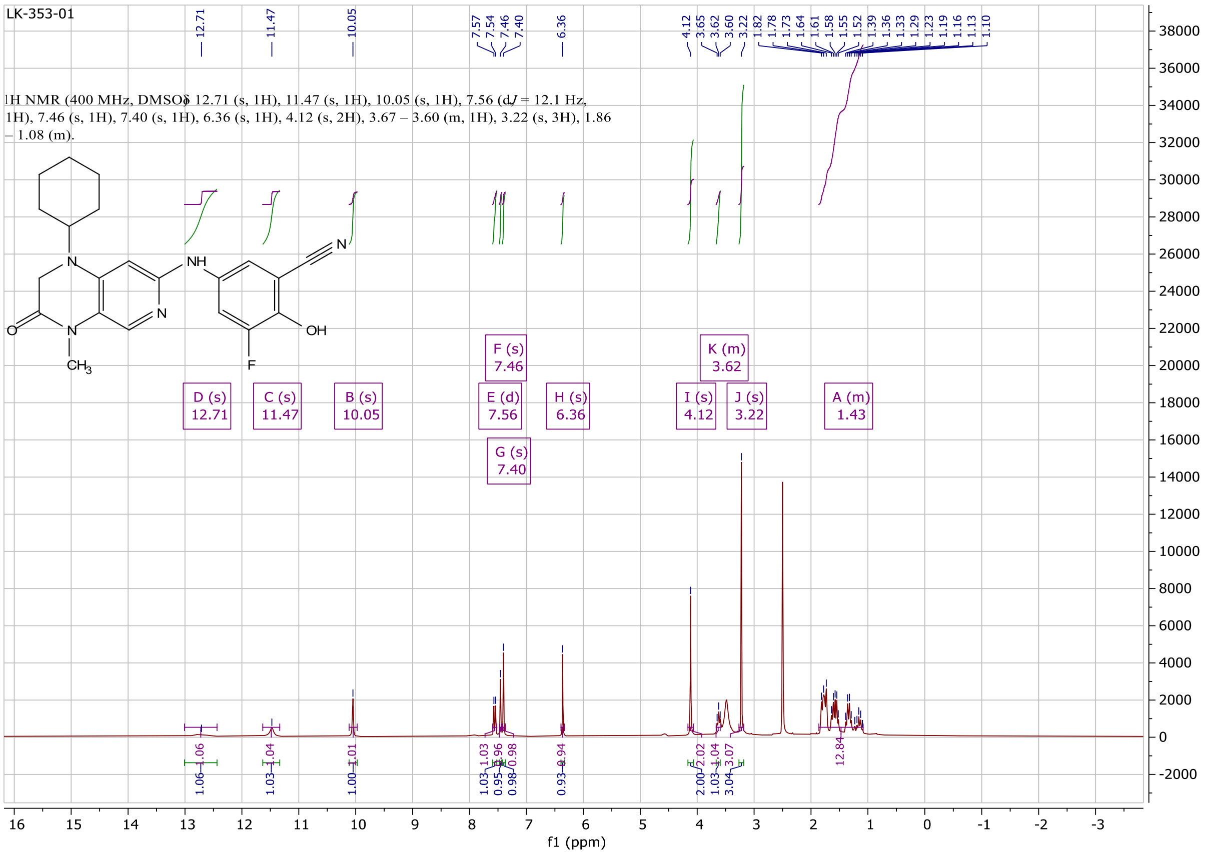

Figure S 54: ^{1}H-NMR spectrum of 38 (400 MHz, DMSO-d_6).

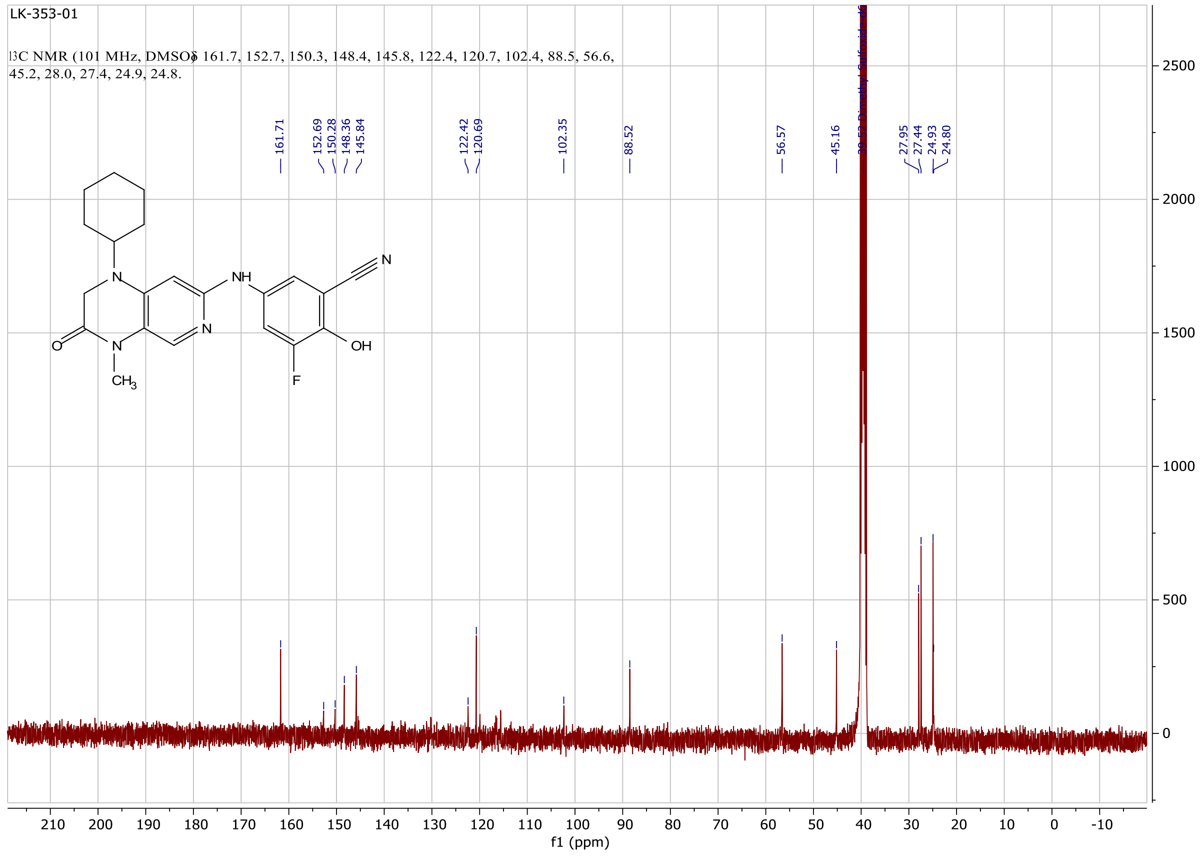

Figure S 55: ^{13}C-NMR spectrum of 38 (400 MHz, DMSO-d_6).

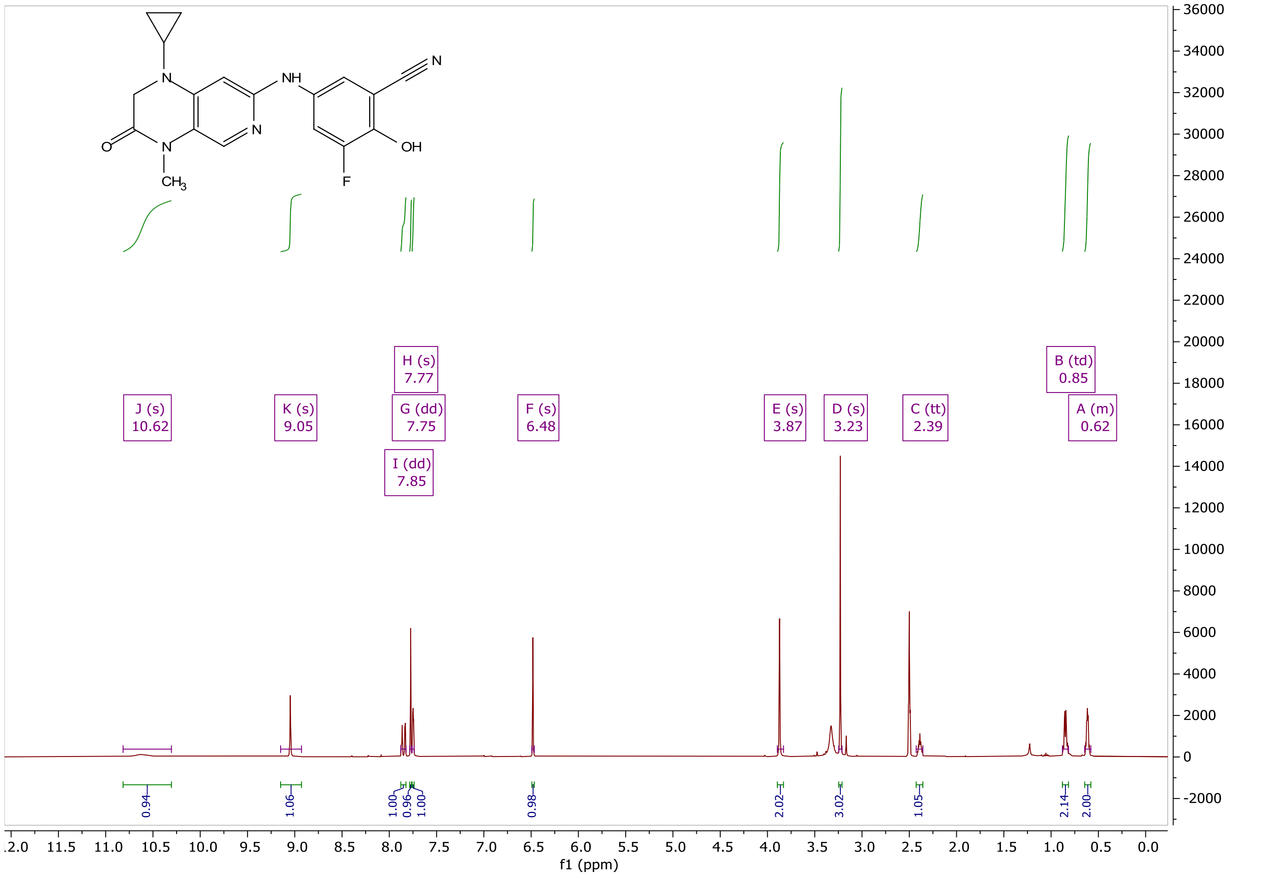

Figure S 56: ^{1}H-NMR spectrum of 39 (400 MHz, DMSO-d_6).

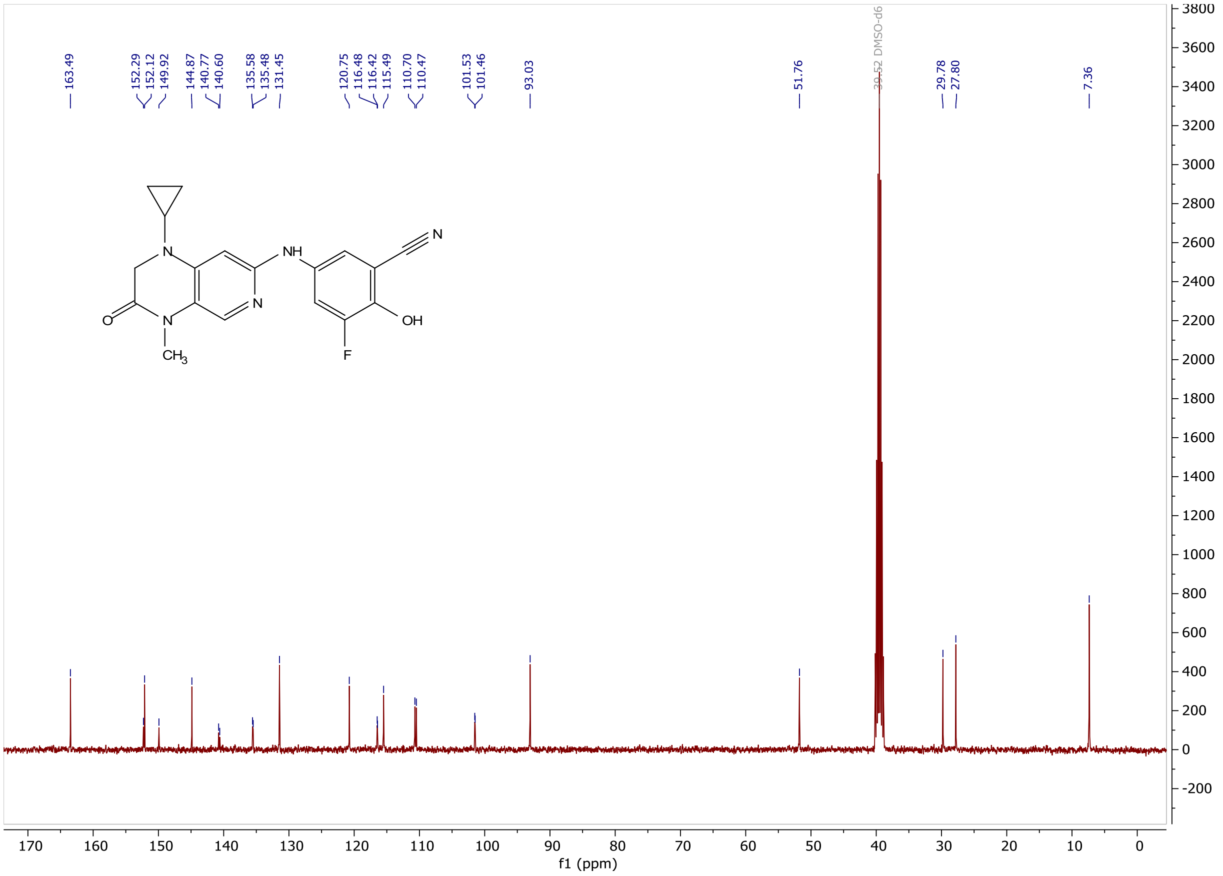

Figure S 57: ^{13}C-NMR spectrum of 39 (400 MHz, DMSO-d$_6$).

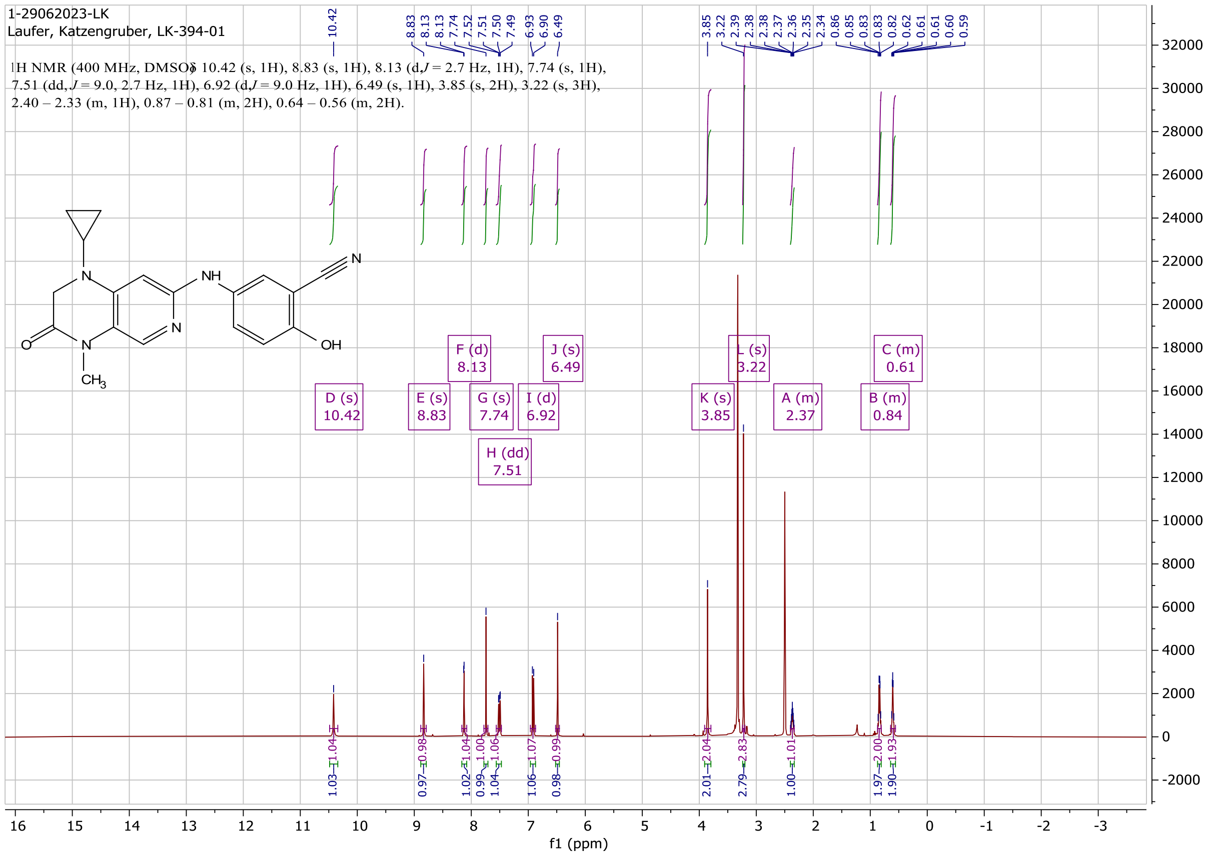

Figure S 58: ^{1}H-NMR spectrum of 40 (400 MHz, DMSO-d_6).

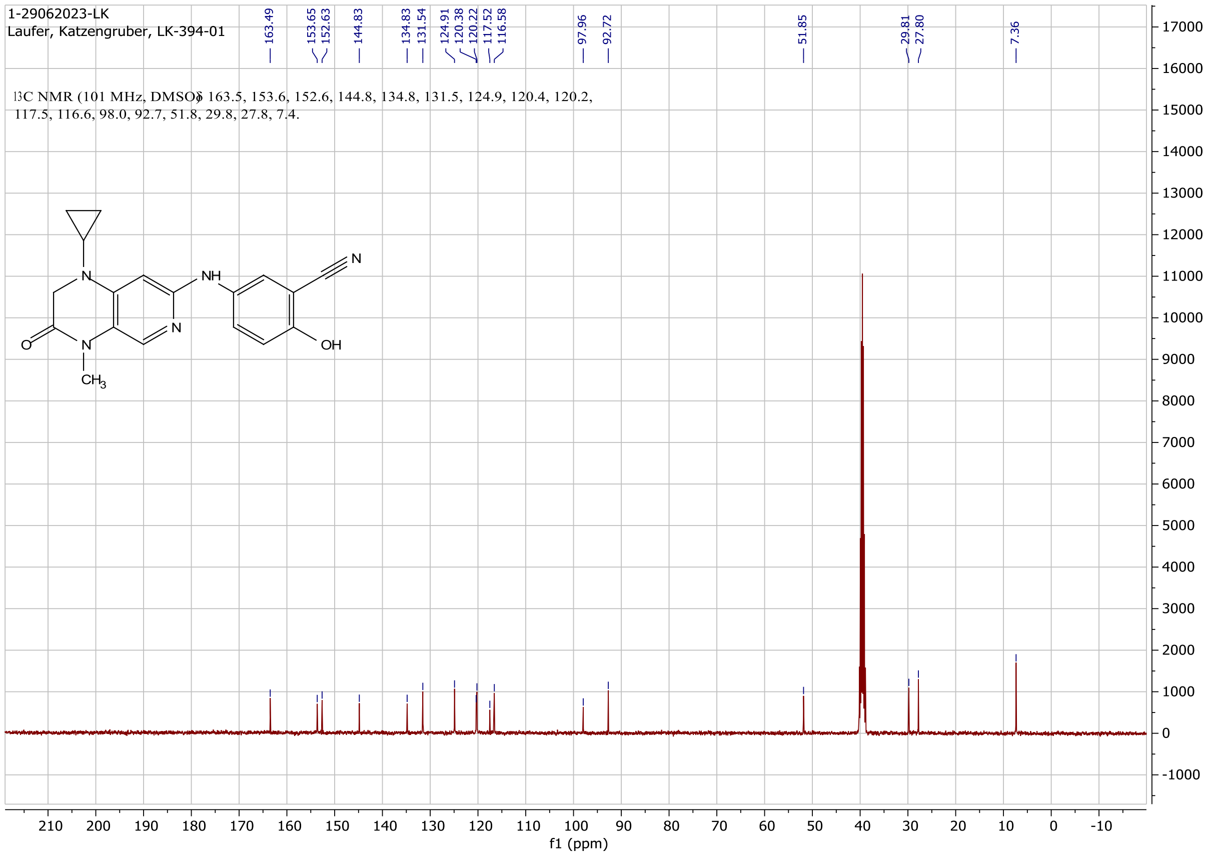

Figure S 59: ^{13}C-NMR spectrum of 40 (400 MHz, DMSO-d_6).

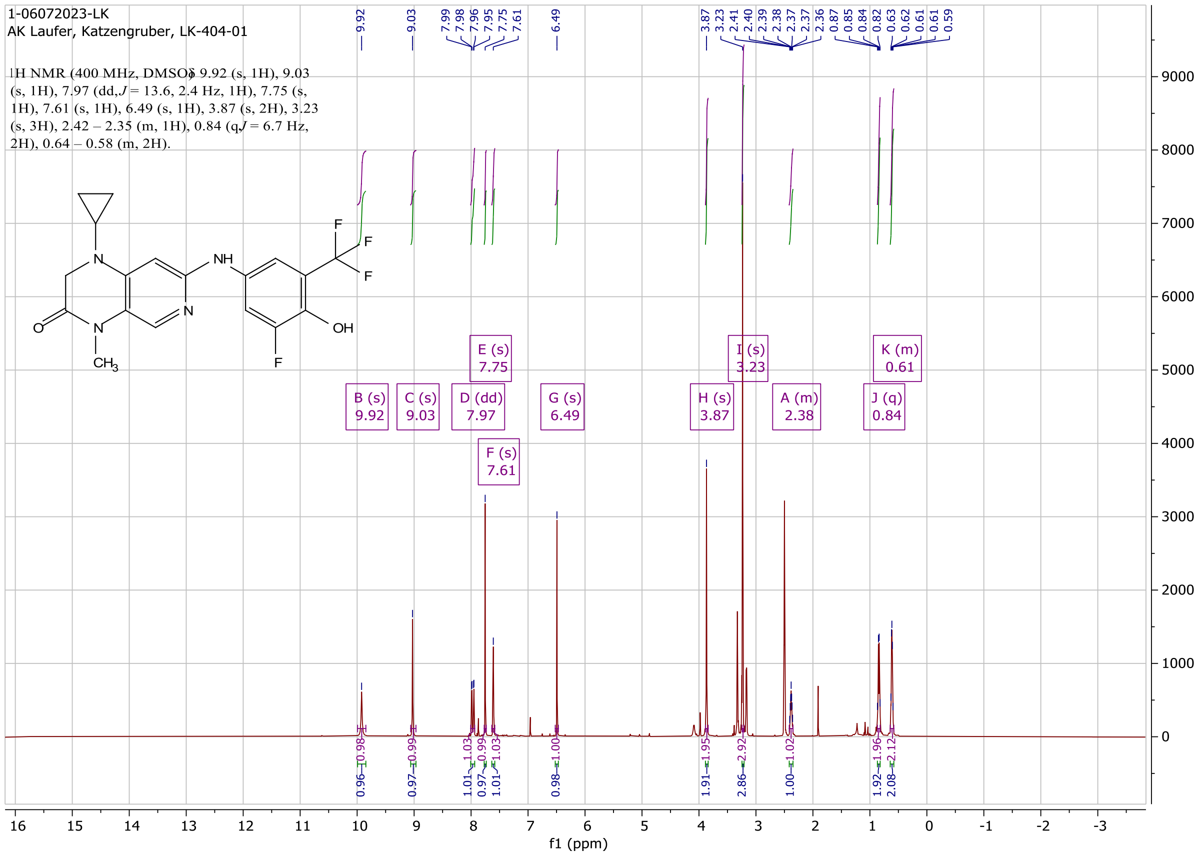

Figure S 60: ^{1}H-NMR spectrum of 41 (400 MHz, DMSO-d_6).

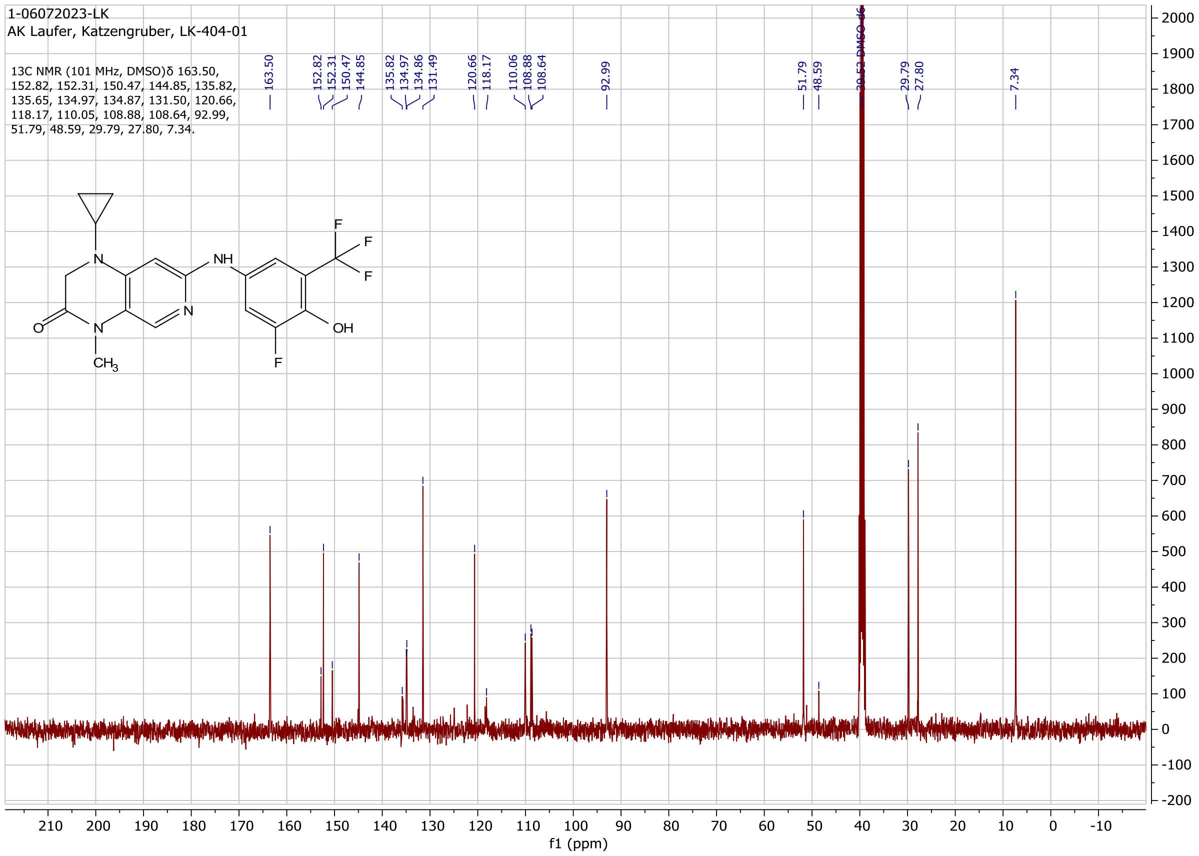

Figure S 61: ^{13}C-NMR spectrum of 41 (400 MHz, DMSO-d_6).

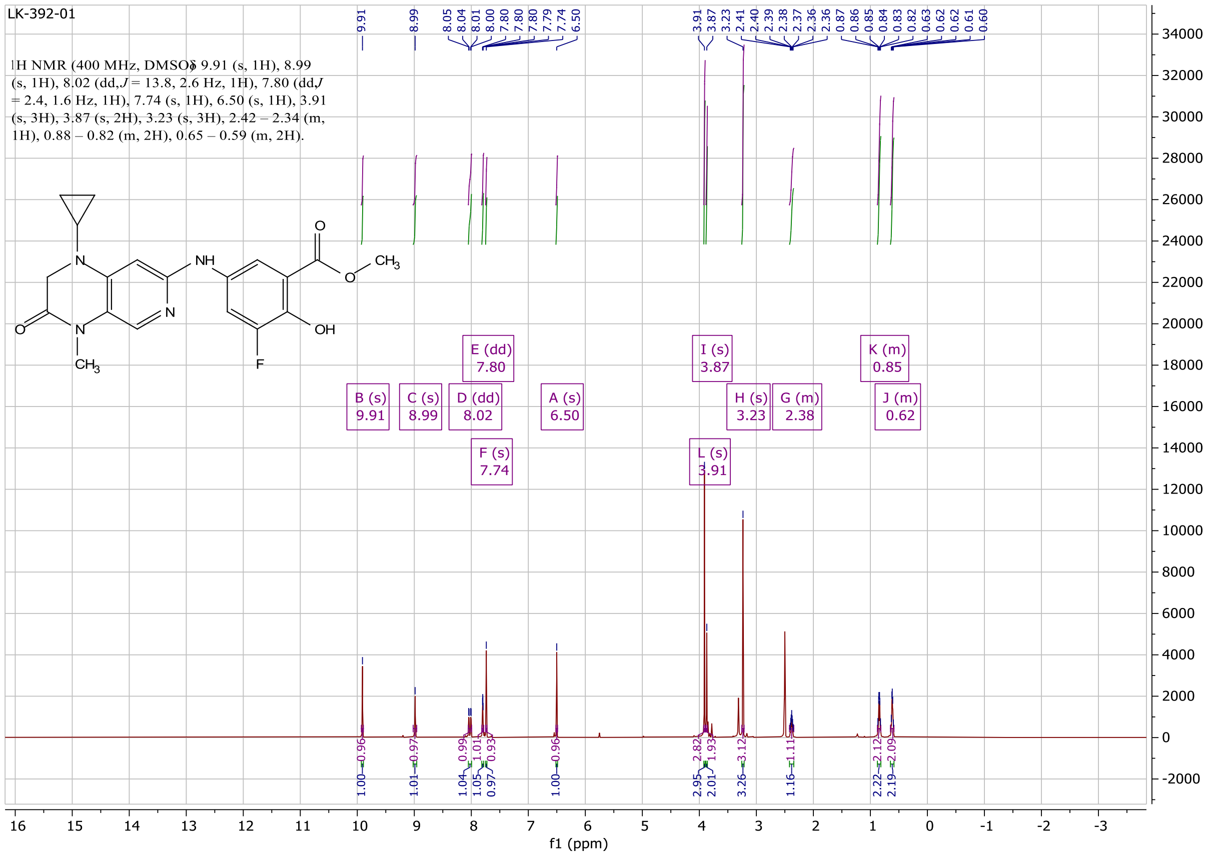

Figure S 62: ^{1}H-NMR spectrum of 42 (400 MHz, DMSO-d_6).

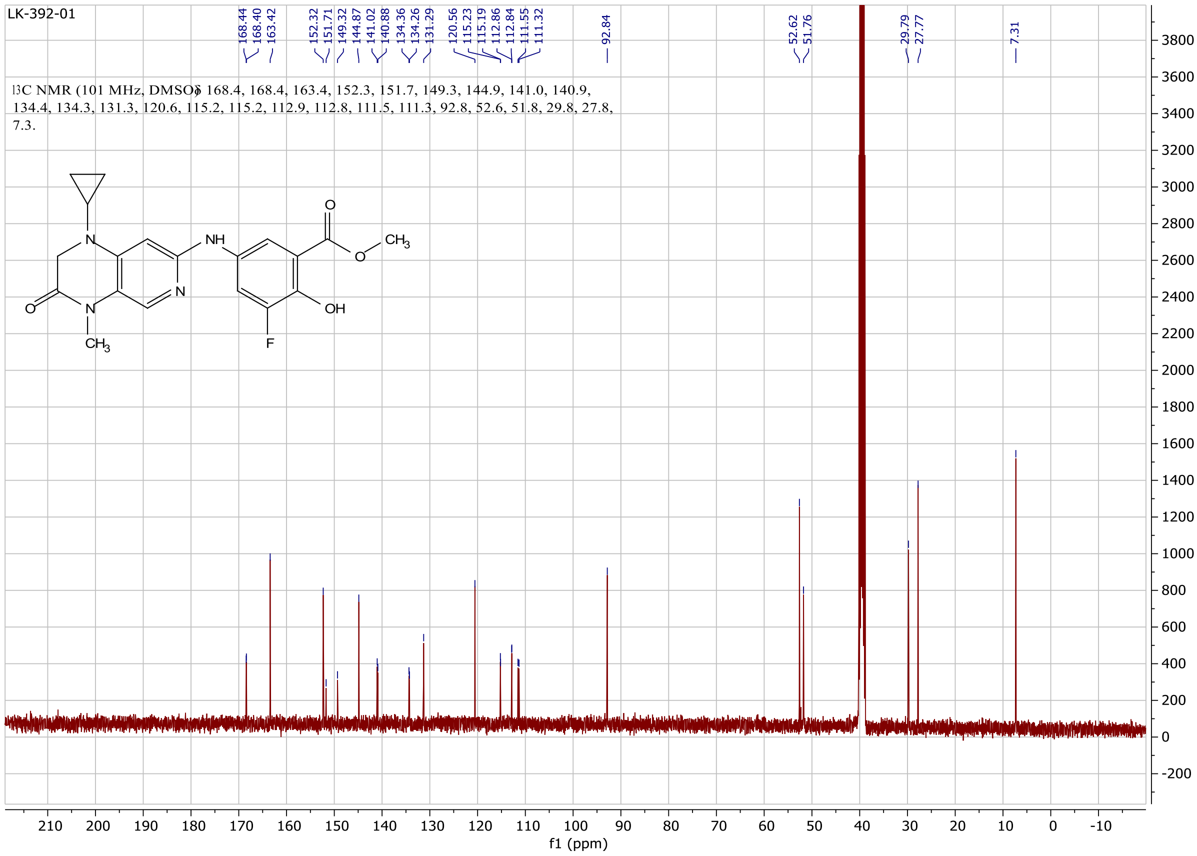

Figure S 63: ^{13}C-NMR spectrum of 42 (400 MHz, DMSO-d_6).

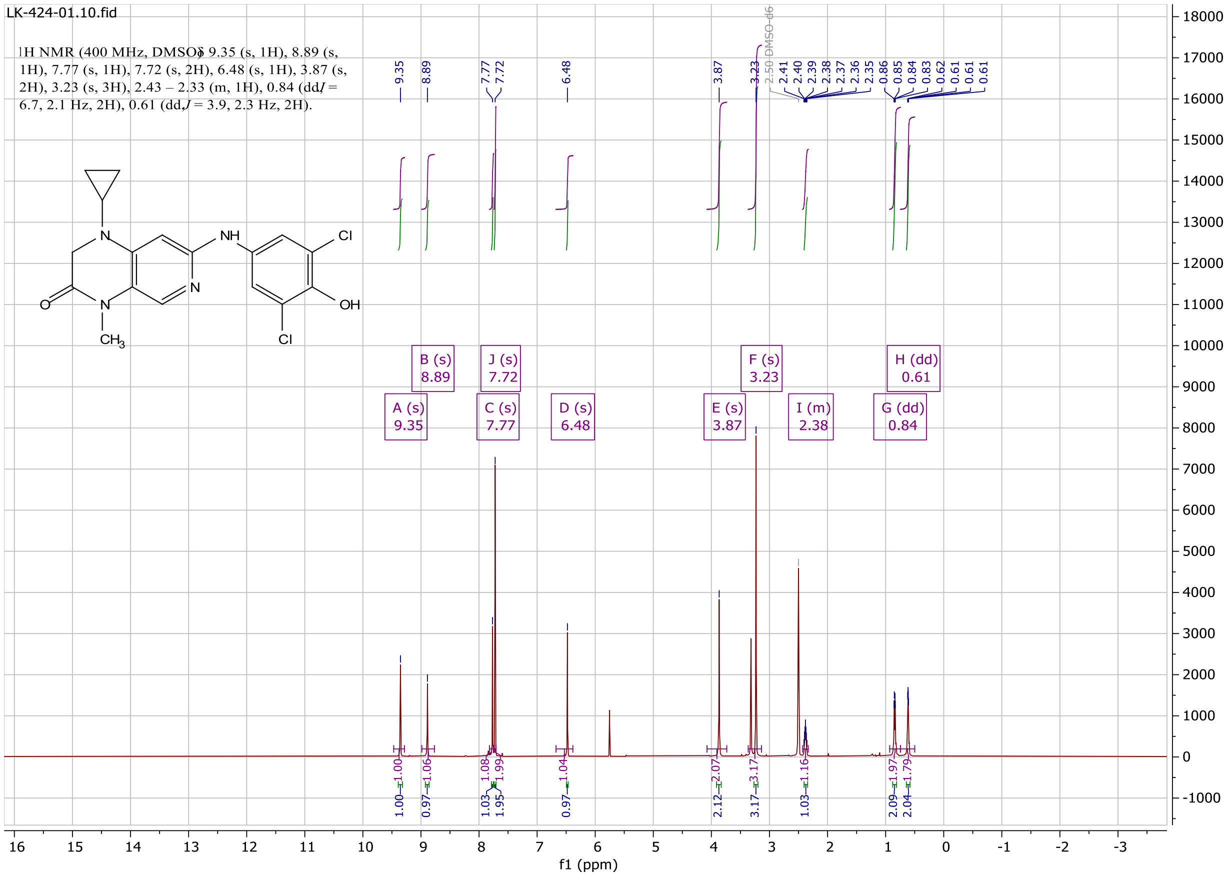

Figure S 64: ^{1}H-NMR spectrum of 43 (400 MHz, DMSO-d_6).

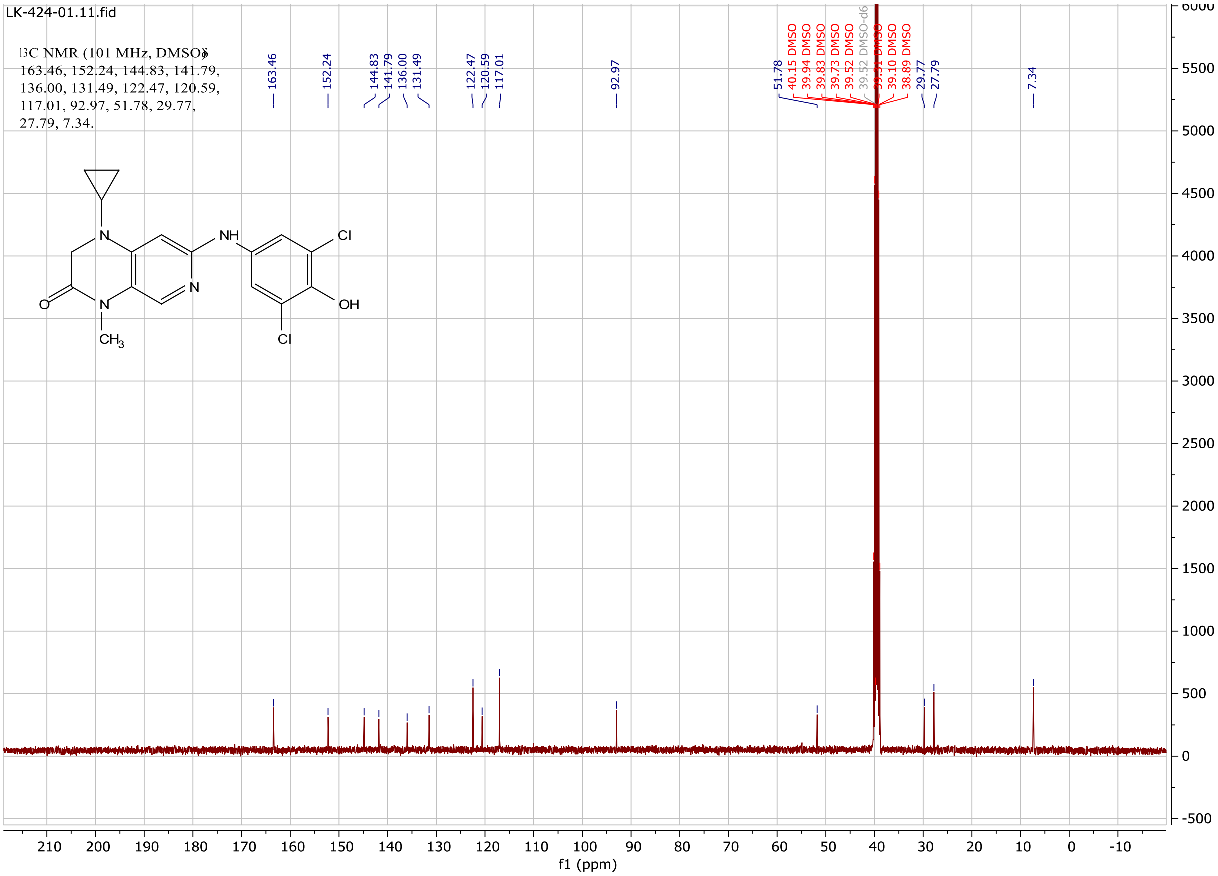

Figure S 65: ^{13}C-NMR spectrum of 43 (400 MHz, DMSO-d_6).

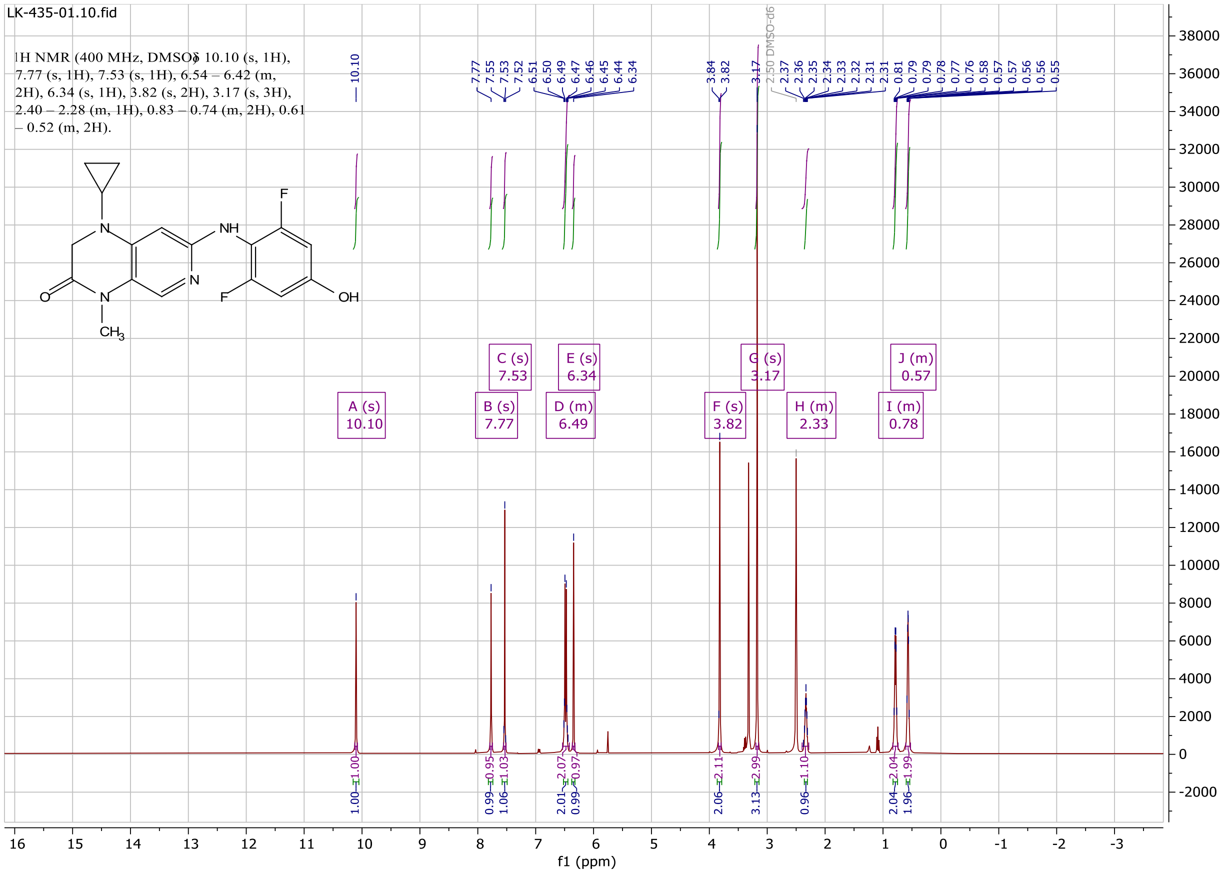

Figure S 66: ^{1}H-NMR spectrum of 44 (400 MHz, DMSO-d_6).

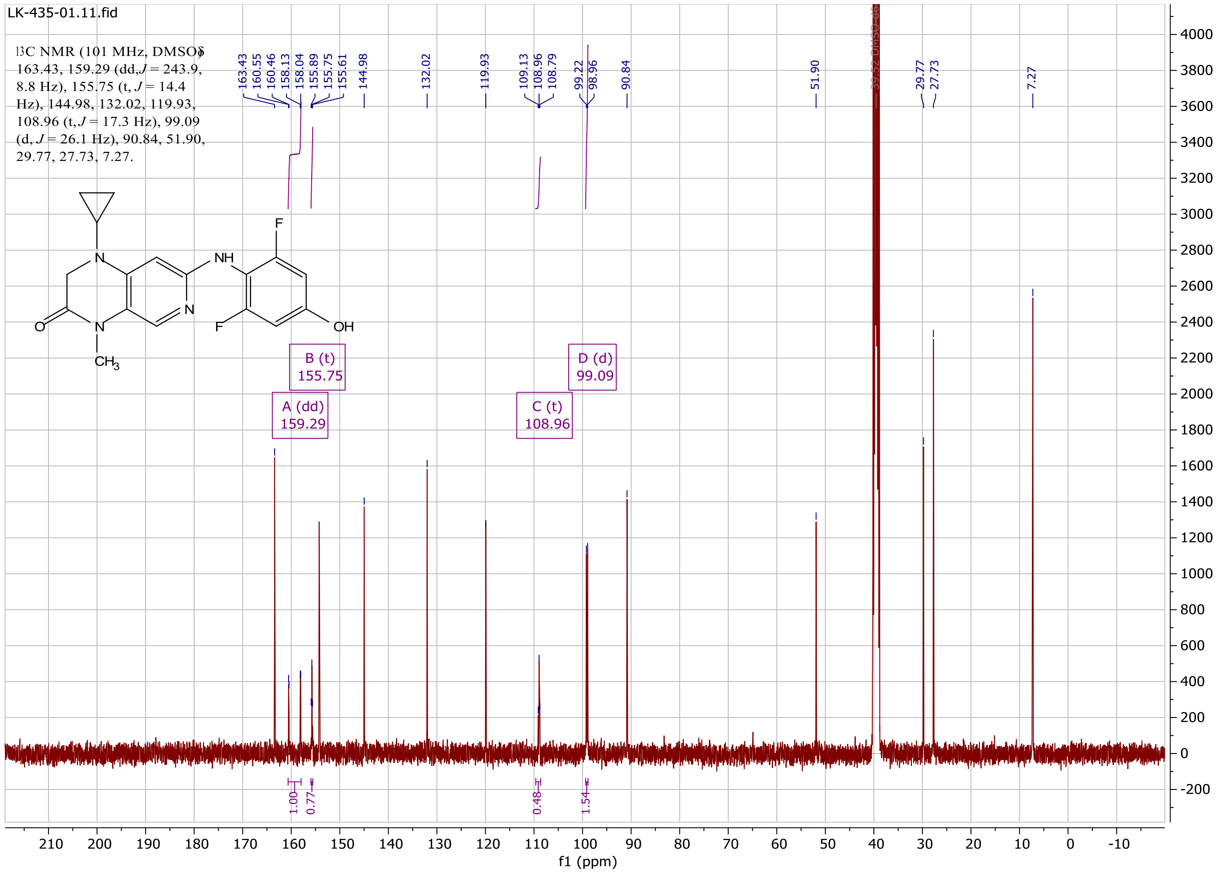

Figure S 67: ^{13}C-NMR spectrum of 44 (400 MHz, DMSO-d_6).

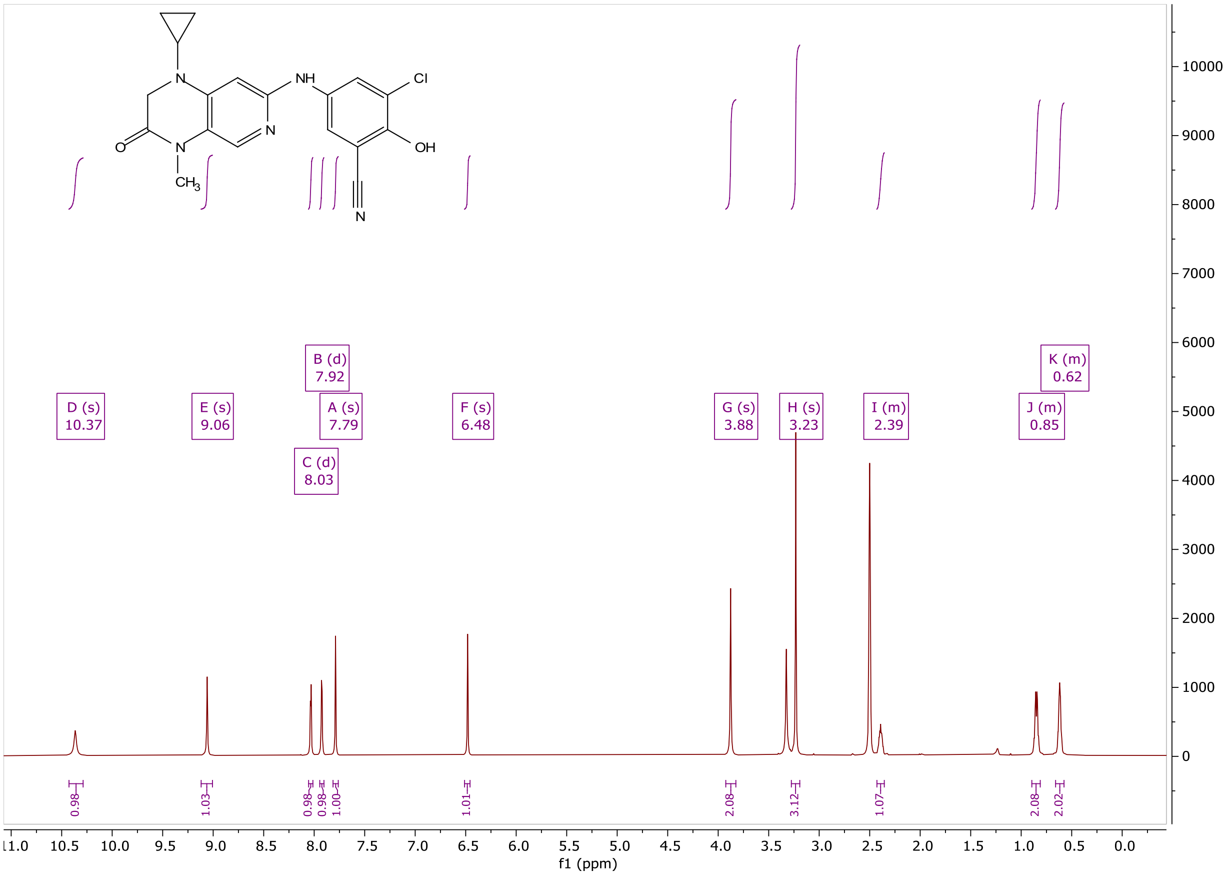

Figure S 68: ^{1}H-NMR spectrum of 45 (400 MHz, DMSO-d_6).

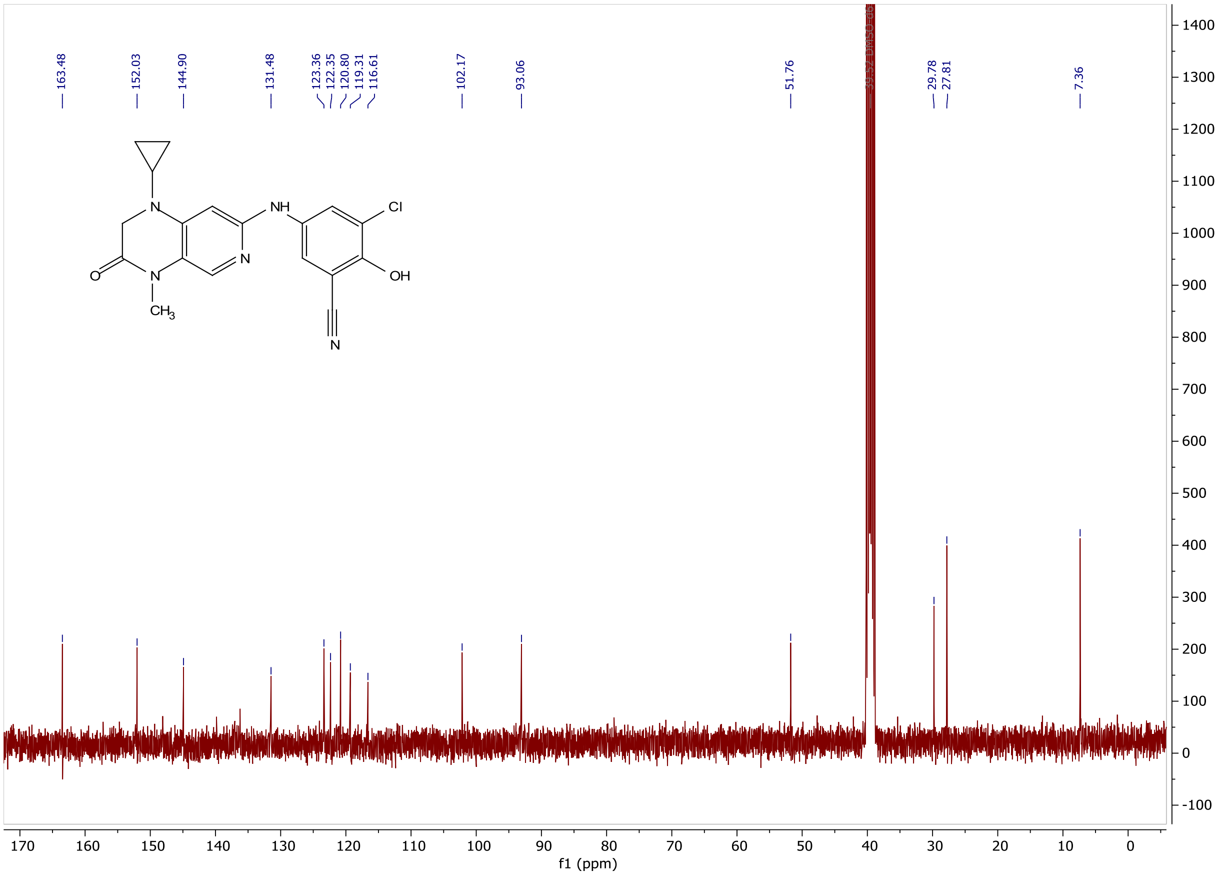

Figure S 69: ^{13}C-NMR spectrum of 45 (400 MHz, DMSO-d_6).

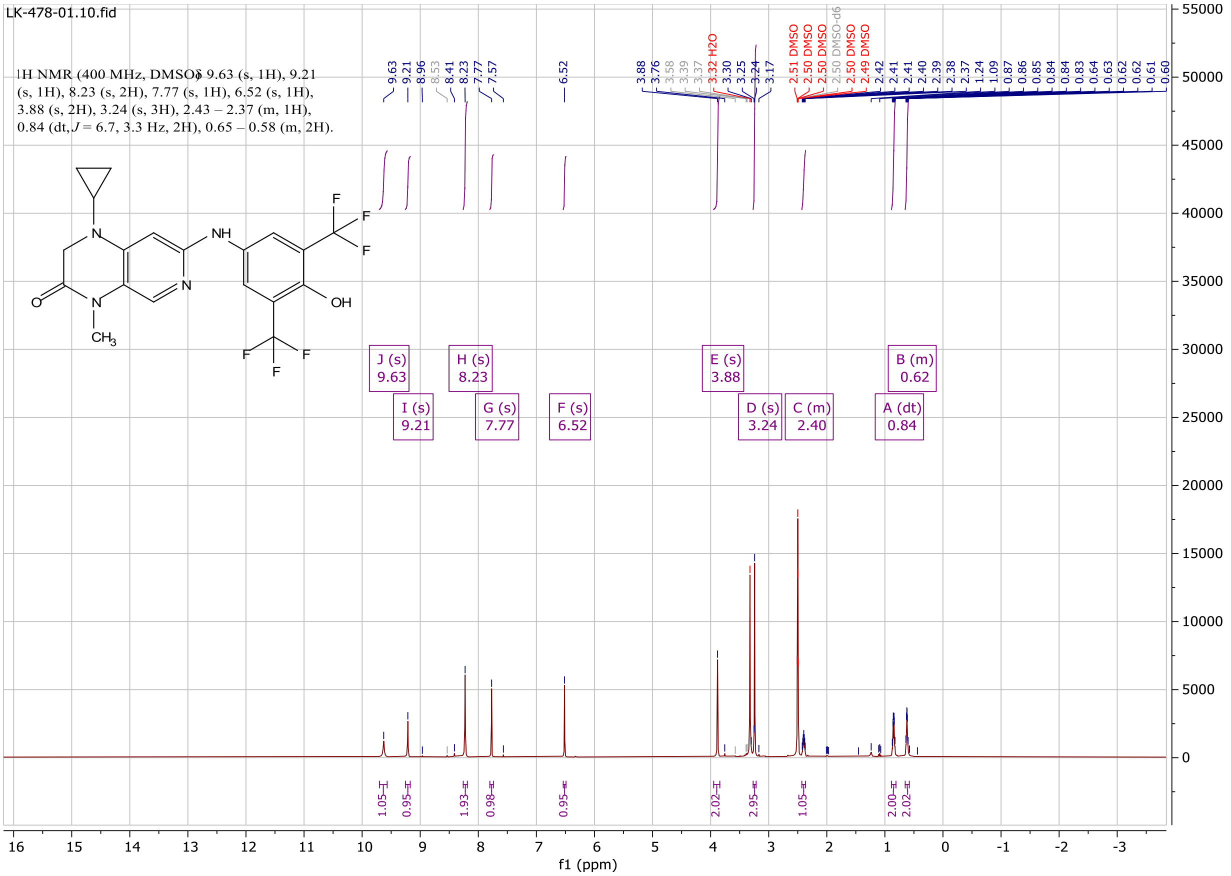

Figure S 70: ^{1}H-NMR spectrum of 46 (400 MHz, DMSO-d_6).

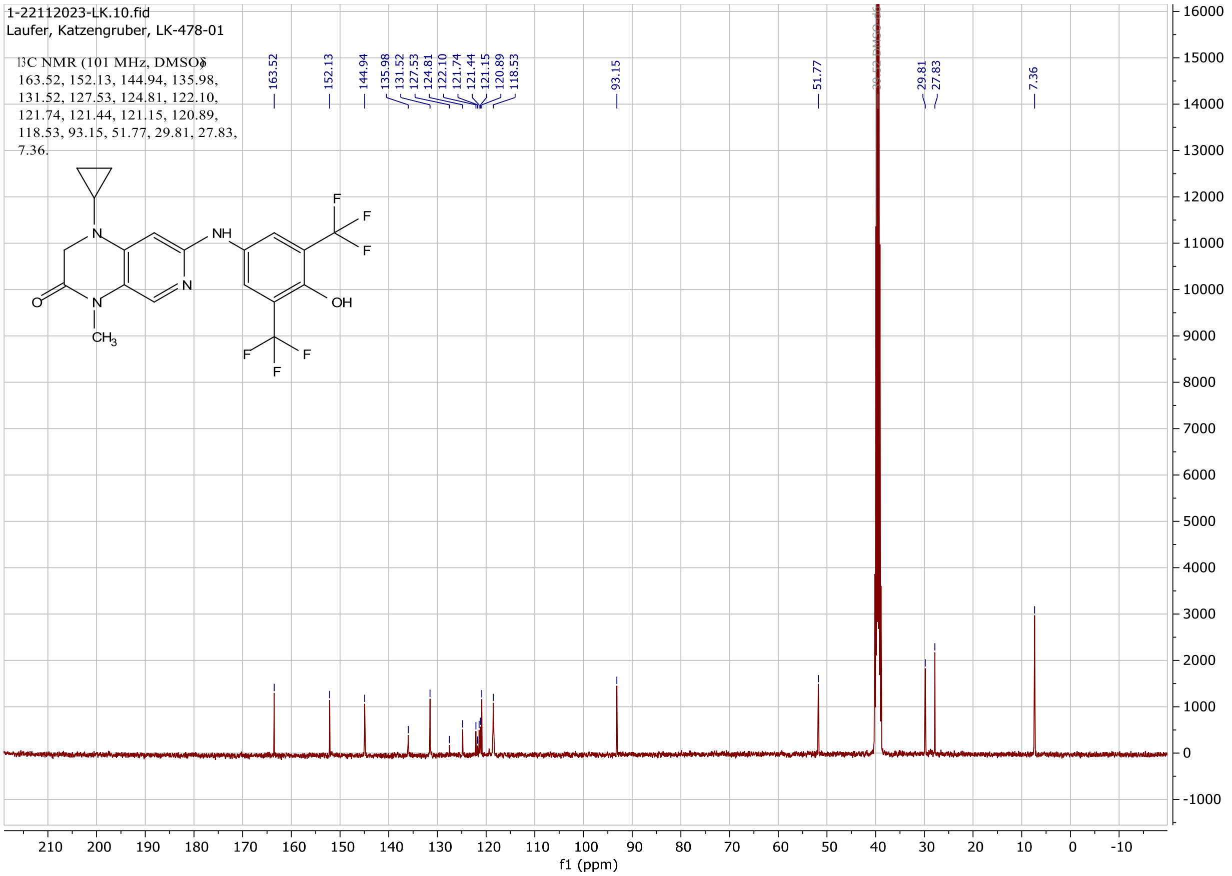

Figure S 71: ^{13}C-NMR spectrum of 46 (400 MHz, DMSO-d_6).

HPLC traces of 39 and 46

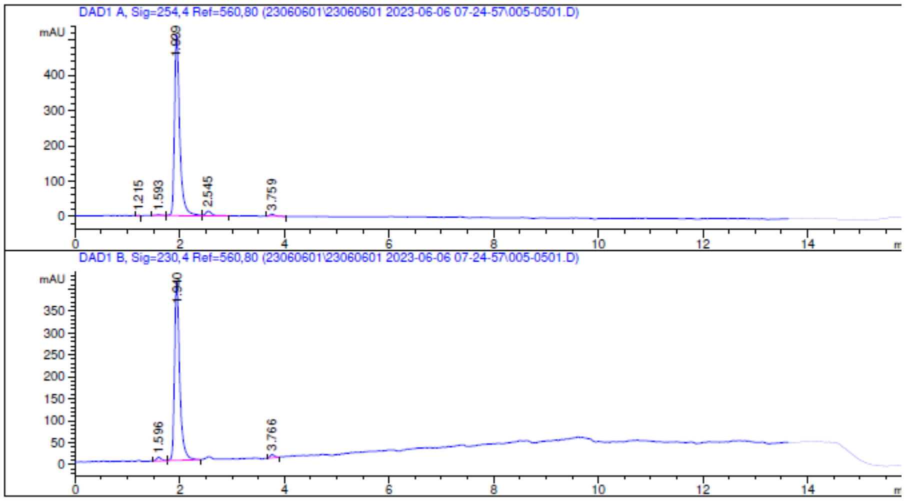

===

Area Percent Report

===

Sorted By : Signal
Multiplier: : 1.0000
Dilution: : 1.0000
Use Multiplier & Dilution Factor with ISTDs

Signal 1: DAD1 A, Sig=254,4 Ref=560,80

Peak #	RetTime [min]	Type	Width [min]	Area [mAU*s]	Height [mAU]	Area %
1	1.215	BB	0.0449	7.03417	2.34993	0.1890
2	1.593	BV	0.0998	16.55192	2.25065	0.4446
3	1.939	VV	0.1027	3545.26465	512.54541	95.2324
4	2.545	VB	0.1285	113.55551	13.18844	3.0503
5	3.759	BB	0.1023	40.34354	5.86117	1.0837

Totals : 3722.74979 536.19560

Signal 2: DAD1 B, Sig=230,4 Ref=560,80

Peak #	RetTime [min]	Type	Width [min]	Area [mAU*s]	Height [mAU]	Area %
1	1.596	BV	0.0921	59.75542	8.93202	2.0620
2	1.940	VB	0.1018	2793.56592	408.27774	96.4007
3	3.766	BB	0.0828	44.54831	7.78539	1.5373

Totals : 2897.86964 424.99515

===

*** End of Report ***

Figure S 72: HPLC traces of 39.

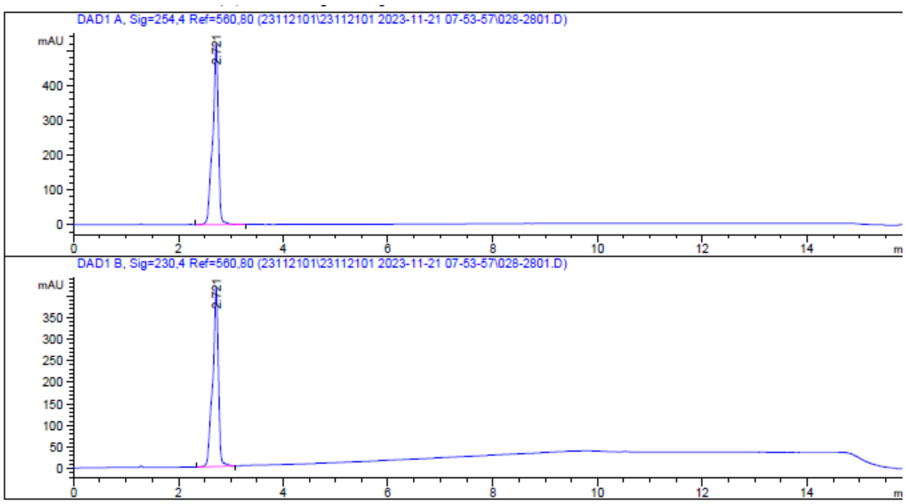


```
=====================================================================
                         Area Percent Report
=====================================================================

Sorted By             :      Signal
Multiplier:                   :      1.0000
Dilution:                     :      1.0000
Use Multiplier & Dilution Factor with ISTDs


Signal 1: DAD1 A, Sig=254,4 Ref=560,80

Peak RetTime Type  Width     Area      Height     Area
  #   [min]        [min]   [mAU*s]     [mAU]        %
----|-------|----|-------|----------|----------|--------|
   1   2.721 BB    0.1080 3931.26709  521.18616 100.0000

Totals :                  3931.26709  521.18616



Signal 2: DAD1 B, Sig=230,4 Ref=560,80

Peak RetTime Type  Width     Area      Height     Area
  #   [min]        [min]   [mAU*s]     [mAU]        %
----|-------|----|-------|----------|----------|--------|
   1   2.721 BB    0.1077 3129.08594  416.10651 100.0000

Totals :                  3129.08594  416.10651
```

Figure S 73: HPLC traces of 45.

HRMS spectra of 39 and 45

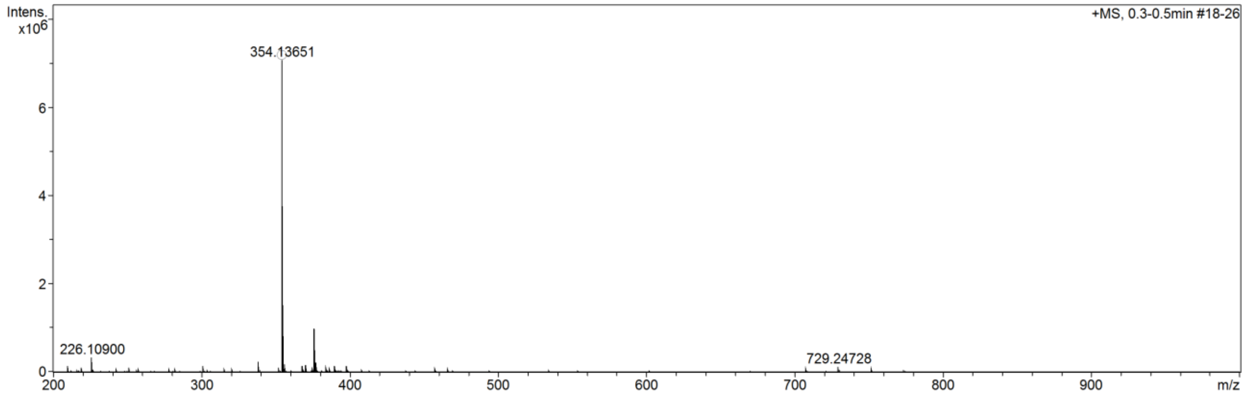

Figure S 74: ESI-HRMS spectrum of 39.

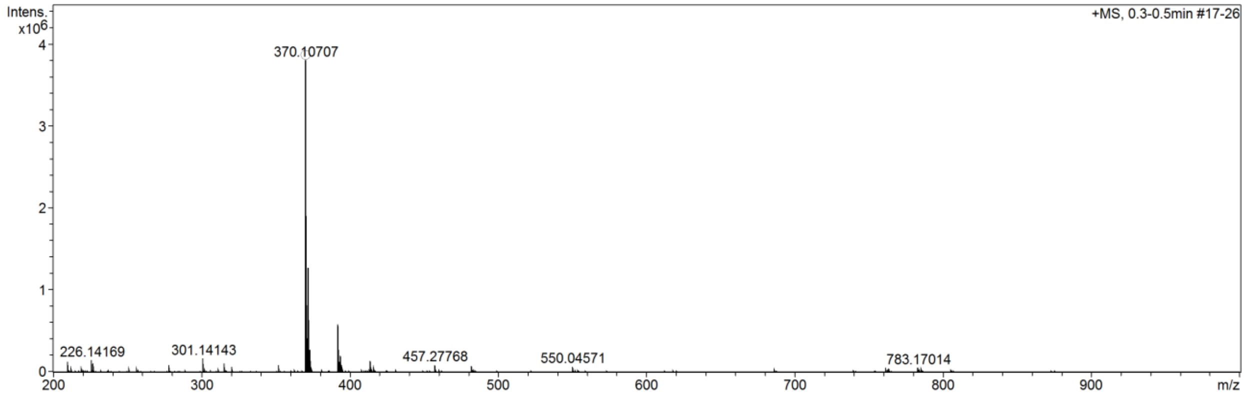

Figure S 75: ESI-HRMS spectrum of 45.

Selectivity profiling data

BI-D1870

Table S2: Profiling of **BI-D1870** at two concentrations (1 µM and 10 µM) against 340 wild-type protein kinases; singlicate measurement. Residual activities (% of control). Yellow: Residual activity < 50 %. *Classification of protein kinase families [219]. AGC: containing PKA, PKG and PKC families. CAMK: Calcium/Calmoduline-dependent protein kinases. CK1: Casein kinase 1 -like. CMGC: containing CDK, MAPK ,GSK3 and CLK families. TK: Tyrosine Kinase. TKL: Tyrosine Kinase-like. STE: Homologs of Yeast Sterile 7, Sterile 11, Sterile 20 Kinases. MKK6 SDTD, RAF1 YDYD, ARAF YDYD: Constitutively active variants.

#	Kinase Name	Kinase Family*	1.0 µM	10 µM
283	RPS6KA6	AGC	4	3
196	MINK1	STE	12	4
280	RPS6KA3	AGC	5	4
295	SNK	OTHER	5	4
279	RPS6KA2	AGC	6	5
327	TYRO3	TK	13	5
261	PLK3	OTHER	9	6
260	PLK1	OTHER	15	8
173	MAP3K11	STE	26	12
193	MELK	CAMK	23	17
317	TRKB	TK	60	18
319	TSF1	OTHER	20	19
197	MKK4	STE	23	20
74	CK1delta	CK1	27	20
218	NEK4	OTHER	39	21
177	MAP4K4	STE	39	22
30	BUB1B	OTHER	37	22
75	CK1epsilon	CK1	30	23
126	FGFR2	TK	80	25
194	MERTK	TK	40	26
208	MUSK	TK	43	27
340	ZAP70	TK	118	27
296	SRC	TK	92	30
101	EGFR	TK	125	31
109	EPHA6	TK	52	32
315	TNK1	TK	66	33
293	SLK	STE	34	34
44	CDK1/CycE1	CMGC	57	34
20	AXL	TK	88	35
312	TIE2	TK	114	36
84	CLK4	CMGC	75	36

18	AuroraB	OTHER	65	36
326	TYK2	TK	54	36
117	ERBB4	TK	96	37
113	EPHB2	TK	61	37
87	CSK	TK	105	37
3	ACK1	TK	77	37
203	MST1	STE	65	37
318	TRKC	TK	67	37
268	PYK2	TK	47	37
52	CDK2/CycA2	CMGC	56	38
278	RPS6KA1	AGC	45	38
124	FES	TK	99	38
112	EPHB1	TK	108	38
125	FGFR1	TK	89	38
53	CDK2/CycD1	CMGC	41	38
331	VEGFR3	TK	96	38
127	FGFR3	TK	100	39
270	RET	TK	74	40
54	CDK2/CycE1	CMGC	47	40
45	CDK10/CycQ	CMGC	41	40
170	LYN	TK	100	41
82	CLK2	CMGC	77	41
24	BMX	TK	75	41
123	FER	TK	103	42
217	NEK3	OTHER	60	42
169	LTK	TK	90	42
338	YES	TK	96	43
316	TRKA	TK	76	43
164	LCK	TK	88	44
12	ALK	TK	94	44
187	MATK	TK	114	44
142	HCK	TK	103	44
204	MST2	STE	68	44
221	NEK9	OTHER	48	44
128	FGFR4	TK	110	45
114	EPHB3	TK	117	46
172	MAP3K10	STE	55	46
332	VRK1	CK1	67	46
81	CLK1	CMGC	74	46
198	MKK6 SDTD	STE	62	46
238	PDK1	AGC	71	47
148	IGF1R	TK	104	47
243	PIM3	CAMK	54	47
29	BTK	TK	98	48
302	STK25	STE	57	48
105	EPHA2	TK	104	48
306	TAOK2	STE	60	49
86	CSF1R	TK	108	49

**102**	**EIF2AK2**	**OTHER**	57	50
**43**	**CDK1/CycB1**	**CMGC**	80	50
**155**	**IRAK4**	**TKL**	75	51
**168**	**LRRK2**	**TKL**	80	51
**175**	**MAP3K9**	**STE**	74	51
**115**	**EPHB4**	**TK**	107	51
**129**	**FGR**	**TK**	95	52
**214**	**NEK1**	**OTHER**	66	53
**132**	**FYN**	**TK**	110	53
**156**	**ITK**	**TK**	93	53
**122**	**FAK**	**TK**	100	54
**206**	**MST4**	**STE**	65	54
**305**	**SYK**	**TK**	95	54
**69**	**CDK9/CycK**	**CMGC**	75	55
**42**	**CDK1/CycA2**	**CMGC**	74	55
**94**	**DNAPK**	**ATYPICAL**	77	56
**241**	**PIM1**	**CAMK**	75	56
**157**	**JAK1**	**TK**	69	57
**103**	**EIF2AK3**	**OTHER**	54	58
**49**	**CDK17/p35NCK**	**CMGC**	71	58
**330**	**VEGFR2**	**TK**	92	58
**67**	**CDK7/CycH/MAT1**	**CMGC**	72	59
**329**	**VEGFR1**	**TK**	105	59
**108**	**EPHA5**	**TK**	111	60
**159**	**JAK3**	**TK**	74	61
**276**	**RON**	**TK**	112	61
**307**	**TAOK3**	**STE**	90	61
**21**	**BLK**	**TK**	79	61
**165**	**LIMK1**	**TKL**	80	62
**110**	**EPHA7**	**TK**	96	63
**297**	**SRMS**	**TK**	89	63
**190**	**MEK5**	**STE**	75	63
**286**	**SAK**	**OTHER**	107	64
**78**	**CK1gamma3**	**CK1**	66	65
**57**	**CDK3/CycC**	**CMGC**	74	65
**250**	**PKCeta**	**AGC**	96	65
**70**	**CDK9/CycT1**	**CMGC**	76	65
**58**	**CDK3/CycE1**	**CMGC**	93	66
**163**	**KIT**	**TK**	73	66
**116**	**ERBB2**	**TK**	92	66
**253**	**PKCmu**	**AGC**	78	66
**274**	**ROCK1**	**AGC**	86	66
**236**	**PDGFRalpha**	**TK**	73	66
**174**	**MAP3K7/MAP3K7IP1**	**STE**	85	67
**48**	**CDK16/CycY**	**CMGC**	79	67
**328**	**ULK2**	**OTHER**	73	67
**104**	**EPHA1**	**TK**	97	67
**131**	**FRK**	**TK**	123	67

**147**	**HRI**	**OTHER**	90	67
**324**	**TTK**	**OTHER**	86	68
**294**	**SNARK**	**CAMK**	100	68
**38**	**CAMKK2**	**OTHER**	71	68
**311**	**TGFBR2**	**TKL**	75	69
**333**	**VRK2**	**CK1**	78	69
**205**	**MST3**	**STE**	91	69
**2**	**ABL2**	**TK**	84	69
**73**	**CK1alpha1**	**CK1**	76	69
**265**	**PRKG1**	**AGC**	91	70
**237**	**PDGFRbeta**	**TK**	92	70
**64**	**CDK6/CycD1**	**CMGC**	76	70
**13**	**AMPKalpha1**	**CAMK**	82	70
**26**	**BRK**	**TK**	104	70
**141**	**GSK3beta**	**CMGC**	91	70
**251**	**PKCgamma**	**AGC**	91	70
**139**	**GSG2**	**OTHER**	76	71
**303**	**STK33**	**CAMK**	74	71
**120**	**ERK5**	**CMGC**	94	71
**215**	**NEK11**	**OTHER**	105	71
**65**	**CDK6/CycD2**	**CMGC**	84	72
**281**	**RPS6KA4**	**AGC**	87	73
**37**	**CAMKK1**	**OTHER**	96	73
**51**	**CDK19/CycC**	**CMGC**	85	73
**46**	**CDK12/CycK**	**CMGC**	80	73
**76**	**CK1gamma1**	**CK1**	83	73
**146**	**HIPK4**	**CMGC**	80	73
**59**	**CDK4/CycD1**	**CMGC**	88	73
**158**	**JAK2**	**TK**	80	73
**339**	**ZAK**	**TKL**	79	74
**244**	**PKA**	**AGC**	89	74
**121**	**ERK7**	**CMGC**	90	74
**119**	**ERK2**	**CMGC**	82	74
**63**	**CDK5/p35NCK**	**CMGC**	83	75
**195**	**MET**	**TK**	87	75
**111**	**EPHA8**	**TK**	94	75
**223**	**NLK**	**CMGC**	82	75
**210**	**MYLK2**	**CAMK**	75	75
**257**	**PKMYT1**	**OTHER**	86	75
**77**	**CK1gamma2**	**CK1**	89	76
**47**	**CDK13/CycK**	**CMGC**	94	76
**154**	**IRAK1**	**TKL**	109	76
**55**	**CDK20/CycH**	**CMGC**	86	76
**207**	**MTOR**	**ATYPICAL**	93	76
**107**	**EPHA4**	**TK**	86	76
**31**	**CAMK1D**	**CAMK**	88	77
**144**	**HIPK2**	**CMGC**	83	77
**254**	**PKCnu**	**AGC**	95	77

**41**	**CDC7/DBF4**	**OTHER**	103	77
**95**	**DYRK1A**	**CMGC**	94	77
**209**	**MYLK**	**CAMK**	113	77
**176**	**MAP4K2**	**STE**	98	78
**309**	**TEC**	**TK**	102	78
**106**	**EPHA3**	**TK**	93	78
**233**	**PAK7**	**STE**	98	78
**259**	**PKN3**	**AGC**	96	78
**180**	**MAPKAPK3**	**CAMK**	97	79
**246**	**PKCbeta1**	**AGC**	94	79
**99**	**DYRK4**	**CMGC**	84	79
**191**	**MEKK2**	**STE**	108	79
**232**	**PAK6**	**STE**	95	79
**200**	**MKNK1**	**CAMK**	88	80
**179**	**MAPKAPK2**	**CAMK**	91	80
**17**	**AuroraA**	**OTHER**	89	80
**27**	**BRSK1**	**CAMK**	110	80
**153**	**INSRR**	**TK**	95	80
**240**	**PHKG2**	**CAMK**	102	80
**130**	**FLT3**	**TK**	89	80
**287**	**SGK1**	**AGC**	89	80
**61**	**CDK4/CycD3**	**CMGC**	98	80
**149**	**IKKalpha**	**OTHER**	93	81
**80**	**CK2alpha2**	**OTHER**	81	81
**211**	**MYLK3**	**CAMK**	112	81
**275**	**ROCK2**	**AGC**	92	81
**310**	**TGFBR1**	**TKL**	82	81
**201**	**MKNK2**	**CAMK**	85	81
**245**	**PKCalpha**	**AGC**	99	81
**56**	**CDK20/CycT1**	**CMGC**	95	81
**150**	**IKKbeta**	**OTHER**	92	81
**298**	**SRPK1**	**CMGC**	82	82
**33**	**CAMK2B**	**CAMK**	124	82
**256**	**PKCzeta**	**AGC**	99	82
**11**	**AKT3**	**AGC**	90	82
**60**	**CDK4/CycD2**	**CMGC**	94	82
**35**	**CAMK2G**	**CAMK**	91	82
**255**	**PKCtheta**	**AGC**	114	82
**83**	**CLK3**	**CMGC**	111	82
**184**	**MARK3**	**CAMK**	98	82
**248**	**PKCdelta**	**AGC**	78	83
**140**	**GSK3alpha**	**CMGC**	102	83
**50**	**CDK18/CycY**	**CMGC**	92	83
**72**	**CHK2**	**CAMK**	108	83
**290**	**SIK1**	**CAMK**	99	83
**313**	**TLK1**	**AGC**	96	84
**185**	**MARK4**	**CAMK**	98	84
**212**	**NDR1**	**AGC**	95	84

**138**	**GRK7**	**AGC**	102	84
**325**	**TXK**	**TK**	94	84
**225**	**p38beta**	**CMGC**	88	84
**36**	**CAMK4**	**CAMK**	90	84
**160**	**JNK1**	**CMGC**	102	84
**216**	**NEK2**	**OTHER**	108	84
**231**	**PAK4**	**STE**	76	84
**66**	**CDK6/CycD3**	**CMGC**	87	85
**242**	**PIM2**	**CAMK**	101	85
**98**	**DYRK3**	**CMGC**	92	85
**62**	**CDK5/p25NCK**	**CMGC**	85	85
**284**	**S6K**	**AGC**	100	85
**334**	**WEE1**	**OTHER**	92	85
**308**	**TBK1**	**OTHER**	91	85
**247**	**PKCbeta2**	**AGC**	101	86
**68**	**CDK8/CycC**	**CMGC**	94	86
**40**	**CDC42BPB**	**AGC**	95	86
**28**	**BRSK2**	**CAMK**	104	86
**266**	**PRKG2**	**AGC**	105	86
**222**	**NIK**	**STE**	101	86
**4**	**ACVR1**	**TKL**	91	86
**304**	**STK39**	**STE**	91	87
**162**	**JNK3**	**CMGC**	92	87
**273**	**RIPK5**	**TKL**	100	87
**264**	**PRKD2**	**CAMK**	89	87
**202**	**MLK4**	**TKL**	110	87
**300**	**STK17A**	**CAMK**	92	87
**7**	**ACVR2B**	**TKL**	94	87
**34**	**CAMK2D**	**CAMK**	94	87
**301**	**STK23**	**CAMK**	102	88
**97**	**DYRK2**	**CMGC**	102	88
**235**	**PBK**	**OTHER**	91	88
**15**	**ARK5**	**CAMK**	90	88
**92**	**DDR2**	**TK**	105	88
**263**	**PRK2**	**AGC**	95	88
**91**	**DCAMKL2**	**CAMK**	96	88
**171**	**MAP3K1**	**STE**	106	88
**277**	**ROS**	**TK**	91	88
**145**	**HIPK3**	**CMGC**	105	88
**6**	**ACVR2A**	**TKL**	97	88
**299**	**SRPK2**	**CMGC**	108	88
**183**	**MARK2**	**CAMK**	99	89
**291**	**SIK2**	**CAMK**	94	89
**271**	**RIPK2**	**TKL**	96	89
**143**	**HIPK1**	**CMGC**	102	89
**71**	**CHK1**	**CAMK**	96	89
**288**	**SGK2**	**AGC**	103	89
**285**	**S6Kbeta**	**AGC**	90	89

**189**	**MEK2**	**STE**	94	89
**262**	**PRK1**	**AGC**	93	89
**292**	**SIK3**	**CAMK**	94	90
**151**	**IKKepsilon**	**OTHER**	93	90
**239**	**PHKG1**	**CAMK**	92	90
**32**	**CAMK2A**	**CAMK**	98	90
**167**	**LKB1/MO25a/STRADa**	**CAMK**	98	90
**226**	**p38delta**	**CMGC**	107	91
**219**	**NEK6**	**OTHER**	102	91
**9**	**AKT1**	**AGC**	101	91
**152**	**INSR**	**TK**	90	91
**118**	**ERK1**	**CMGC**	121	92
**188**	**MEK1**	**STE**	140	92
**227**	**p38gamma**	**CMGC**	89	92
**192**	**MEKK3**	**STE**	98	93
**336**	**WNK2**	**OTHER**	68	93
**320**	**TSK2**	**CAMK**	130	93
**230**	**PAK3**	**STE**	95	94
**282**	**RPS6KA5**	**AGC**	103	94
**93**	**DMPK**	**AGC**	107	94
**90**	**DAPK3**	**CAMK**	102	94
**186**	**MASTL**	**AGC**	98	94
**213**	**NDR2**	**AGC**	97	95
**267**	**PRKX**	**AGC**	113	95
**229**	**PAK2**	**STE**	95	95
**1**	**ABL1**	**TK**	110	95
**161**	**JNK2**	**CMGC**	94	95
**314**	**TLK2**	**AGC**	118	96
**79**	**CK2alpha1**	**OTHER**	102	96
**322**	**TTBK1**	**CK1**	128	96
**199**	**MKK7**	**STE**	92	96
**96**	**DYRK1B**	**CMGC**	111	96
**258**	**PKMzeta**	**AGC**	98	96
**178**	**MAP4K5**	**STE**	103	96
**181**	**MAPKAPK5**	**CAMK**	115	97
**182**	**MARK1**	**CAMK**	107	97
**234**	**PASK**	**CAMK**	104	98
**335**	**WNK1**	**OTHER**	95	98
**16**	**ASK1**	**STE**	103	98
**337**	**WNK3**	**OTHER**	108	98
**8**	**ACVRL1**	**TKL**	101	98
**249**	**PKCepsilon**	**AGC**	90	100
**289**	**SGK3**	**AGC**	102	100
**166**	**LIMK2**	**TKL**	110	100
**39**	**CDC42BPA**	**AGC**	119	100
**272**	**RIPK4**	**TKL**	105	100
**14**	**ARAF YDYD**	**TKL**	106	101
**10**	**AKT2**	**AGC**	107	102

**25**	**BRAF**	**TKL**	88	102
**23**	**BMPR1B**	**TKL**	98	102
**134**	**GRK3**	**AGC**	112	103
**85**	**COT**	**STE**	105	103
**220**	**NEK7**	**OTHER**	116	103
**224**	**p38alpha**	**CMGC**	107	103
**252**	**PKCiota**	**AGC**	100	103
**19**	**AuroraC**	**OTHER**	102	104
**100**	**EEF2K**	**ATYPICAL**	111	105
**137**	**GRK6**	**AGC**	121	105
**269**	**RAF1 YDYD**	**TKL**	89	107
**5**	**ACVR1B**	**TKL**	109	109
**22**	**BMPR1A**	**TKL**	113	109
**323**	**TTBK2**	**CK1**	105	109
**228**	**PAK1**	**STE**	91	110
**89**	**DAPK2**	**CAMK**	134	110
**136**	**GRK5**	**AGC**	119	112
**133**	**GRK2**	**AGC**	100	112
**321**	**TSSK1**	**CAMK**	128	114
**135**	**GRK4**	**AGC**	125	117
**88**	**DAPK1**	**CAMK**	130	121
**Selectivity Score (< 50 % residual activity):**			**0.076**	**0.235**

Compound 39

Table S3: Profiling of compound **39** at two concentrations (1 µM and 10 µM) against 350 wild-type protein kinases; singlicate measurement. Residual activities (% of control). Yellow: Residual activity < 50 %. *Classification of protein kinase families [219]: AGC: containing PKA, PKG and PKC families. CAMK: Calcium/Calmoduline-dependent protein kinases. CK1: Casein kinase 1 -like. CMGC: containing CDK, MAPK ,GSK3 and CLK families. TK: Tyrosine Kinase. TKL: Tyrosine Kinase-like. STE: Homologs of Yeast Sterile 7, Sterile 11, Sterile 20 Kinases. MKK6 SDTD, RAF1 YDYD, ARAF YDYD: Constitutively active variants.

#	**Kinase Name**	**Kinase Family***	**1.0 µM**	**10 µM**
**205**	**MKK4**	**STE**	1	-4
**231**	**NLK**	**CMGC**	31	8
**302**	**SNK**	**OTHER**	43	9
**290**	**RPS6KA6**	**AGC**	61	15
**335**	**TYRO3**	**TK**	65	18
**286**	**RPS6KA2**	**AGC**	68	19
**287**	**RPS6KA3**	**AGC**	71	19

**268**	**PLK3**	**OTHER**	67	19
**245**	**PDGFRbeta**	**TK**	70	23
**251**	**PIM3**	**CAMK**	74	24
**125**	**ERK2**	**CMGC**	74	24
**115**	**EPHA6**	**TK**	77	27
**79**	**CK1delta**	**CK1**	80	28
**201**	**MERTK**	**TK**	65	30
**174**	**LRRK2**	**TKL**	83	31
**99**	**DNAPK**	**ATYPICAL**	74	31
**211**	**MST1**	**STE**	76	33
**310**	**STK25**	**STE**	84	34
**212**	**MST2**	**STE**	70	35
**80**	**CK1epsilon**	**CK1**	82	35
**200**	**MELK**	**CAMK**	91	38
**108**	**EIF2AK3**	**OTHER**	99	40
**136**	**FLT3**	**TK**	80	40
**124**	**ERK1**	**CMGC**	92	41
**179**	**MAP3K11**	**STE**	83	42
**107**	**EIF2AK2**	**OTHER**	83	43
**332**	**TTK**	**OTHER**	79	43
**267**	**PLK1**	**OTHER**	89	45
**76**	**CHK2**	**CAMK**	80	46
**83**	**CK1gamma3**	**CK1**	98	46
**184**	**MAP4K4**	**STE**	84	46
**132**	**FGFR2**	**TK**	84	47
**119**	**EPHB2**	**TK**	83	48
**216**	**MUSK**	**TK**	93	49
**146**	**GSK3alpha**	**CMGC**	98	50
**246**	**PDK1**	**AGC**	91	50
**168**	**JNK3**	**CMGC**	90	50
**203**	**MINK1**	**STE**	89	50
**20**	**AXL**	**TK**	89	50
**16**	**ASK1**	**STE**	85	51
**30**	**BUB1B**	**OTHER**	90	52
**18**	**AuroraB**	**OTHER**	100	52
**311**	**STK33**	**CAMK**	89	53
**214**	**MST4**	**STE**	90	53
**67**	**CDK5/p35NCK**	**CMGC**	94	54
**39**	**CDC42BPA**	**AGC**	99	55
**285**	**RPS6KA1**	**AGC**	97	55
**58**	**CDK2/CycE1**	**CMGC**	91	56
**56**	**CDK2/CycA2**	**CMGC**	98	57
**178**	**MAP3K10**	**STE**	107	58
**249**	**PIM1**	**CAMK**	91	58

**334**	**TYK2**	**TK**	96	59
**324**	**TRKA**	**TK**	85	61
**327**	**TSF1**	**OTHER**	97	61
**206**	**MKK6 SDTD**	**STE**	91	61
**167**	**JNK2**	**CMGC**	96	61
**78**	**CK1alpha1**	**CK1**	98	61
**204**	**MKK3**	**STE**	85	62
**202**	**MET**	**TK**	88	63
**342**	**VRK1**	**CK1**	93	63
**300**	**SLK**	**STE**	92	63
**244**	**PDGFRalpha**	**TK**	96	64
**21**	**BLK**	**TK**	92	64
**152**	**HIPK4**	**CMGC**	92	65
**288**	**RPS6KA4**	**AGC**	95	65
**301**	**SNARK**	**CAMK**	107	66
**277**	**RET**	**TK**	106	67
**66**	**CDK5/p25NCK**	**CMGC**	92	68
**185**	**MAP4K5**	**STE**	108	68
**166**	**JNK1**	**CMGC**	100	68
**111**	**EPHA2**	**TK**	95	69
**182**	**MAP4K1**	**STE**	102	69
**226**	**NEK4**	**OTHER**	94	70
**180**	**MAP3K7/MAP3K7IP1**	**STE**	95	70
**292**	**S6Kbeta**	**AGC**	94	70
**17**	**AuroraA**	**OTHER**	94	70
**89**	**CLK4**	**CMGC**	99	71
**12**	**ALK**	**TK**	95	71
**133**	**FGFR3**	**TK**	94	71
**98**	**DMPK**	**AGC**	92	72
**13**	**AMPKalpha1**	**CAMK**	92	72
**326**	**TRKC**	**TK**	102	72
**336**	**ULK1**	**OTHER**	94	73
**225**	**NEK3**	**OTHER**	73	73
**57**	**CDK2/CycD1**	**CMGC**	91	73
**82**	**CK1gamma2**	**CK1**	98	73
**93**	**DAPK1**	**CAMK**	95	73
**314**	**TAOK2**	**STE**	107	73
**154**	**IGF1R**	**TK**	98	74
**241**	**PAK7**	**STE**	106	74
**337**	**ULK2**	**OTHER**	99	74
**118**	**EPHB1**	**TK**	109	75
**213**	**MST3**	**STE**	98	75
**181**	**MAP3K9**	**STE**	95	75
**101**	**DYRK1B**	**CMGC**	104	75

**85**	**CK2alpha2**	**OTHER**	100	75
**86**	**CLK1**	**CMGC**	98	75
**325**	**TRKB**	**TK**	125	75
**94**	**DAPK2**	**CAMK**	92	75
**116**	**EPHA7**	**TK**	102	75
**131**	**FGFR1**	**TK**	100	76
**343**	**VRK2**	**CK1**	110	76
**229**	**NEK9**	**OTHER**	94	76
**157**	**IKKepsilon**	**OTHER**	97	76
**294**	**SGK1**	**AGC**	98	77
**271**	**PRKD2**	**CAMK**	88	77
**50**	**CDK15/CycA2**	**CMGC**	112	77
**59**	**CDK20/CycH**	**CMGC**	98	77
**262**	**PKCnu**	**AGC**	106	77
**275**	**PYK2**	**TK**	99	77
**280**	**RIPK5**	**TKL**	87	77
**51**	**CDK15/CycB1**	**CMGC**	97	77
**239**	**PAK4**	**STE**	97	78
**87**	**CLK2**	**CMGC**	103	78
**220**	**NDR1**	**AGC**	88	79
**81**	**CK1gamma1**	**CK1**	105	79
**44**	**CDK1/CycE1**	**CMGC**	94	79
**147**	**GSK3beta**	**CMGC**	100	79
**145**	**GSG2**	**OTHER**	99	80
**170**	**LCK**	**TK**	106	80
**299**	**SIK3**	**CAMK**	96	80
**127**	**ERK7**	**CMGC**	98	80
**281**	**ROCK1**	**AGC**	101	80
**165**	**JAK3**	**TK**	106	80
**340**	**VEGFR2**	**TK**	97	80
**84**	**CK2alpha1**	**OTHER**	98	80
**344**	**WEE1**	**OTHER**	96	80
**276**	**RAF1 YDYD**	**TKL**	85	80
**263**	**PKCtheta**	**AGC**	96	81
**196**	**MEK2**	**STE**	104	81
**161**	**IRAK4**	**TKL**	96	81
**261**	**PKCmu**	**AGC**	96	82
**312**	**STK39**	**STE**	94	82
**71**	**CDK7/CycH/MAT1**	**CMGC**	96	82
**97**	**DDR2**	**TK**	98	82
**227**	**NEK6**	**OTHER**	92	82
**217**	**MYLK**	**CAMK**	101	82
**92**	**CSK**	**TK**	95	83
**323**	**TNK1**	**TK**	98	83

**183**	**MAP4K2**	**STE**	101	83
**45**	**CDK10/CycQ**	**CMGC**	95	83
**175**	**LTK**	**TK**	99	83
**215**	**MTOR**	**ATYPICAL**	104	83
**72**	**CDK8/CycC**	**CMGC**	102	83
**207**	**MKK7**	**STE**	95	83
**53**	**CDK17/p35NCK**	**CMGC**	103	84
**329**	**TSSK1**	**CAMK**	97	84
**308**	**STK17B**	**CAMK**	94	84
**102**	**DYRK2**	**CMGC**	82	84
**232**	**p38alpha**	**CMGC**	93	84
**282**	**ROCK2**	**AGC**	95	84
**19**	**AuroraC**	**OTHER**	106	84
**198**	**MEKK2**	**STE**	113	85
**105**	**EEF2K**	**ATYPICAL**	84	85
**103**	**DYRK3**	**CMGC**	94	85
**60**	**CDK20/CycT1**	**CMGC**	100	85
**209**	**MKNK2**	**CAMK**	91	85
**320**	**TIE2**	**TK**	110	85
**91**	**CSF1R**	**TK**	101	85
**128**	**FAK**	**TK**	109	86
**289**	**RPS6KA5**	**AGC**	96	86
**298**	**SIK2**	**CAMK**	90	86
**14**	**ARAF YDYD**	**TKL**	94	86
**233**	**p38beta**	**CMGC**	92	86
**33**	**CAMK2B**	**CAMK**	107	86
**219**	**MYLK3**	**CAMK**	100	86
**2**	**ABL2**	**TK**	96	86
**62**	**CDK3/CycE1**	**CMGC**	103	86
**100**	**DYRK1A**	**CMGC**	87	86
**236**	**PAK1**	**STE**	94	86
**293**	**SAK**	**OTHER**	103	87
**70**	**CDK6/CycD3**	**CMGC**	88	87
**135**	**FGR**	**TK**	99	87
**224**	**NEK2**	**OTHER**	110	88
**11**	**AKT3**	**AGC**	89	88
**113**	**EPHA4**	**TK**	101	88
**341**	**VEGFR3**	**TK**	110	88
**1**	**ABL1**	**TK**	94	88
**159**	**INSRR**	**TK**	88	88
**265**	**PKMzeta**	**AGC**	120	88
**138**	**FYN**	**TK**	94	88
**218**	**MYLK2**	**CAMK**	88	88
**29**	**BTK**	**TK**	100	88

**40**	**CDC42BPB**	**AGC**	103	88
**266**	**PKN3**	**AGC**	103	88
**258**	**PKCeta**	**AGC**	102	88
**272**	**PRKG1**	**AGC**	89	88
**64**	**CDK4/CycD2**	**CMGC**	102	88
**155**	**IKKalpha**	**OTHER**	106	89
**264**	**PKCzeta**	**AGC**	99	89
**126**	**ERK5**	**CMGC**	97	89
**112**	**EPHA3**	**TK**	94	89
**42**	**CDK1/CycA2**	**CMGC**	100	89
**15**	**ARK5**	**CAMK**	97	89
**160**	**IRAK1**	**TKL**	103	89
**346**	**WNK2**	**OTHER**	96	89
**75**	**CHK1**	**CAMK**	88	89
**230**	**NIK**	**STE**	96	89
**164**	**JAK2**	**TK**	97	89
**256**	**PKCdelta**	**AGC**	98	89
**210**	**MLK4**	**TKL**	108	90
**243**	**PBK**	**OTHER**	104	90
**106**	**EGFR**	**TK**	106	90
**173**	**LKB1/MO25a/STRADa**	**CAMK**	104	90
**43**	**CDK1/CycB1**	**CMGC**	85	90
**316**	**TBK1**	**OTHER**	115	90
**193**	**MASTL**	**AGC**	99	90
**172**	**LIMK2**	**TKL**	87	90
**176**	**LYN**	**TK**	111	90
**31**	**CAMK1D**	**CAMK**	115	90
**28**	**BRSK2**	**CAMK**	99	90
**34**	**CAMK2D**	**CAMK**	95	90
**319**	**TGFBR2**	**TKL**	95	90
**134**	**FGFR4**	**TK**	100	91
**37**	**CAMKK1**	**OTHER**	95	91
**129**	**FER**	**TK**	109	91
**25**	**BRAF**	**TKL**	97	91
**23**	**BMPR1B**	**TKL**	104	91
**156**	**IKKbeta**	**OTHER**	98	91
**248**	**PHKG2**	**CAMK**	103	91
**90**	**COT**	**STE**	93	91
**309**	**STK23**	**CAMK**	97	91
**24**	**BMX**	**TK**	92	91
**252**	**PKA**	**AGC**	91	91
**307**	**STK17A**	**CAMK**	100	91
**104**	**DYRK4**	**CMGC**	102	92
**186**	**MAPKAPK2**	**CAMK**	104	92

**121**	**EPHB4**	**TK**	107	92
**284**	**ROS**	**TK**	97	92
**333**	**TXK**	**TK**	91	92
**306**	**SRPK2**	**CMGC**	96	92
**237**	**PAK2**	**STE**	100	92
**61**	**CDK3/CycC**	**CMGC**	89	92
**348**	**YES**	**TK**	110	92
**63**	**CDK4/CycD1**	**CMGC**	97	92
**187**	**MAPKAPK3**	**CAMK**	102	92
**273**	**PRKG2**	**AGC**	97	92
**330**	**TTBK1**	**CK1**	112	92
**95**	**DAPK3**	**CAMK**	113	92
**296**	**SGK3**	**AGC**	100	93
**55**	**CDK19/CycC**	**CMGC**	107	93
**313**	**SYK**	**TK**	100	93
**315**	**TAOK3**	**STE**	99	93
**110**	**EPHA1**	**TK**	103	93
**123**	**ERBB4**	**TK**	111	93
**74**	**CDK9/CycT1**	**CMGC**	97	93
**130**	**FES**	**TK**	106	93
**177**	**MAP3K1**	**STE**	97	93
**122**	**ERBB2**	**TK**	105	93
**162**	**ITK**	**TK**	95	94
**240**	**PAK6**	**STE**	105	94
**257**	**PKCepsilon**	**AGC**	103	94
**270**	**PRK2**	**AGC**	103	94
**142**	**GRK5**	**AGC**	110	94
**149**	**HIPK1**	**CMGC**	99	94
**234**	**p38delta**	**CMGC**	99	94
**238**	**PAK3**	**STE**	94	94
**46**	**CDK11B/CycK**	**CMGC**	103	94
**36**	**CAMK4**	**CAMK**	95	94
**52**	**CDK16/CycY**	**CMGC**	93	95
**321**	**TLK1**	**AGC**	107	95
**339**	**VEGFR1**	**TK**	96	95
**305**	**SRPK1**	**CMGC**	99	95
**88**	**CLK3**	**CMGC**	108	95
**322**	**TLK2**	**AGC**	105	95
**54**	**CDK18/CycY**	**CMGC**	98	95
**349**	**ZAK**	**TKL**	99	95
**278**	**RIPK2**	**TKL**	103	95
**38**	**CAMKK2**	**OTHER**	104	95
**153**	**HRI**	**OTHER**	102	95
**254**	**PKCbeta1**	**AGC**	102	95

**317**	**TEC**	**TK**	99	95
**318**	**TGFBR1**	**TKL**	102	96
**221**	**NDR2**	**AGC**	109	96
**6**	**ACVR2A**	**TKL**	112	96
**283**	**RON**	**TK**	107	96
**169**	**KIT**	**TK**	108	96
**120**	**EPHB3**	**TK**	106	96
**297**	**SIK1**	**CAMK**	103	96
**65**	**CDK4/CycD3**	**CMGC**	105	96
**148**	**HCK**	**TK**	104	96
**117**	**EPHA8**	**TK**	95	96
**171**	**LIMK1**	**TKL**	114	96
**109**	**EIF2AK4**	**OTHER**	91	96
**222**	**NEK1**	**OTHER**	94	97
**194**	**MATK**	**TK**	116	97
**338**	**ULK3**	**OTHER**	101	97
**260**	**PKCiota**	**AGC**	92	97
**151**	**HIPK3**	**CMGC**	97	97
**41**	**CDC7/DBF4**	**OTHER**	97	97
**347**	**WNK3**	**OTHER**	104	97
**69**	**CDK6/CycD2**	**CMGC**	106	98
**27**	**BRSK1**	**CAMK**	99	98
**114**	**EPHA5**	**TK**	122	98
**5**	**ACVR1B**	**TKL**	95	98
**304**	**SRMS**	**TK**	96	98
**47**	**CDK12/CycK**	**CMGC**	92	98
**22**	**BMPR1A**	**TKL**	102	99
**345**	**WNK1**	**OTHER**	89	99
**250**	**PIM2**	**CAMK**	104	99
**274**	**PRKX**	**AGC**	107	99
**48**	**CDK13/CycK**	**CMGC**	102	99
**3**	**ACK1**	**TK**	109	99
**150**	**HIPK2**	**CMGC**	92	100
**163**	**JAK1**	**TK**	91	100
**269**	**PRK1**	**AGC**	120	100
**8**	**ACVRL1**	**TKL**	103	100
**255**	**PKCbeta2**	**AGC**	104	101
**253**	**PKCalpha**	**AGC**	105	101
**192**	**MARK4**	**CAMK**	100	101
**208**	**MKNK1**	**CAMK**	113	101
**191**	**MARK3**	**CAMK**	102	101
**295**	**SGK2**	**AGC**	102	101
**190**	**MARK2**	**CAMK**	102	101
**279**	**RIPK4**	**TKL**	107	101

**291**	**S6K**	**AGC**	100	101
**32**	**CAMK2A**	**CAMK**	107	101
**9**	**AKT1**	**AGC**	97	101
**189**	**MARK1**	**CAMK**	118	102
**259**	**PKCgamma**	**AGC**	106	102
**49**	**CDK14/CycY**	**CMGC**	94	102
**73**	**CDK9/CycK**	**CMGC**	102	102
**4**	**ACVR1**	**TKL**	94	102
**331**	**TTBK2**	**CK1**	99	102
**158**	**INSR**	**TK**	95	103
**197**	**MEK5**	**STE**	101	103
**68**	**CDK6/CycD1**	**CMGC**	105	103
**247**	**PHKG1**	**CAMK**	94	103
**77**	**CIT 1-450**	**AGC**	116	104
**139**	**GRK2**	**AGC**	98	105
**223**	**NEK11**	**OTHER**	112	105
**235**	**p38gamma**	**CMGC**	106	105
**26**	**BRK**	**TK**	96	105
**303**	**SRC**	**TK**	112	106
**328**	**TSK2**	**CAMK**	100	107
**350**	**ZAP70**	**TK**	128	107
**10**	**AKT2**	**AGC**	101	108
**144**	**GRK7**	**AGC**	111	108
**199**	**MEKK3**	**STE**	111	108
**140**	**GRK3**	**AGC**	105	109
**228**	**NEK7**	**OTHER**	116	109
**7**	**ACVR2B**	**TKL**	133	109
**35**	**CAMK2G**	**CAMK**	111	110
**242**	**PASK**	**CAMK**	119	111
**137**	**FRK**	**TK**	109	112
**195**	**MEK1**	**STE**	119	114
**143**	**GRK6**	**AGC**	132	114
**141**	**GRK4**	**AGC**	140	115
**188**	**MAPKAPK5**	**CAMK**	110	118
**96**	**DCAMKL2**	**CAMK**	105	121
**Selectivity Score (< 50 % residual activity):**			**0.009**	**0.109**

Thermal Shift Assay data of BI-D1870, 39, and 45

Replicate 1

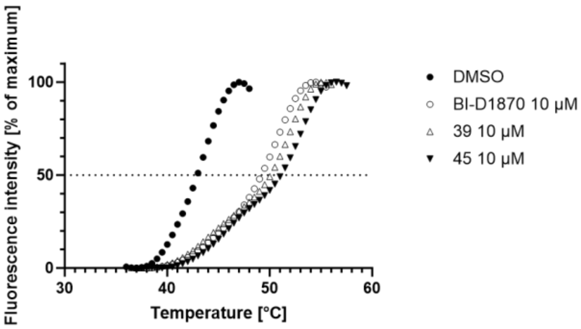

ΔT_m (BI-D1870) = 6°C
ΔT_m (39) = 7°C
ΔT_m (45) = 8°C

Replicate 2

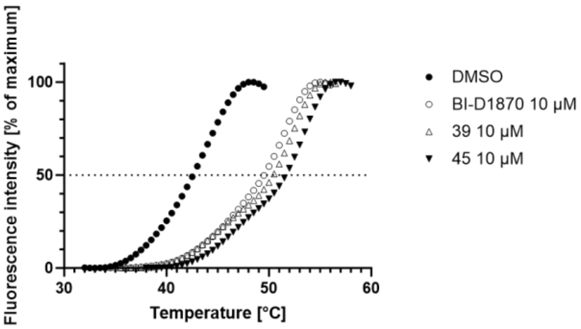

ΔT_m (BI-D1870) = 7.5°C
ΔT_m (LN6836) = 8.5°C
ΔT_m (LN7049) = 9.5°C

Replicate 3

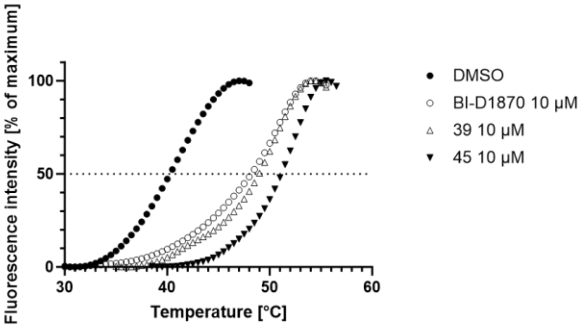

ΔT_m (BI-D1870) = 7.5°C
ΔT_m (LN6836) = 8.5°C
ΔT_m (LN7049) = 10.5°C

Figure S 76: Results of thermal stabilization of 1 µg activated MKK4 with DMSO (negative control), 10 µM BI-D1870, 39 and 45 (n = 3). The images were generated using GraphPad Prism 10.1.1.

NCI-2030 cellular toxicity data of BI-D1870, 39, and 45

Replicate 1

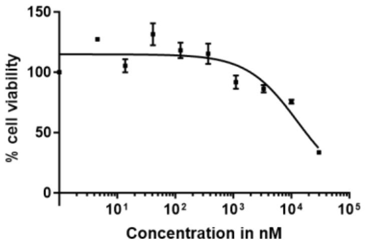

IC$_{50}$ (BI-D1870): 12.64 μM

Replicate 2

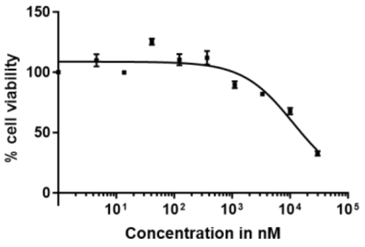

IC$_{50}$ (BI-D1870): 11.33 μM

Replicate 3

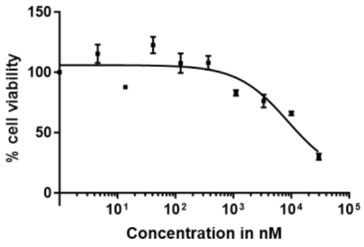

IC$_{50}$ (BI-D1870): 8.75 μM

Replicate 1

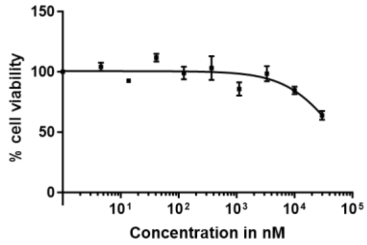

IC$_{50}$ (39): n.d.

Replicate 2

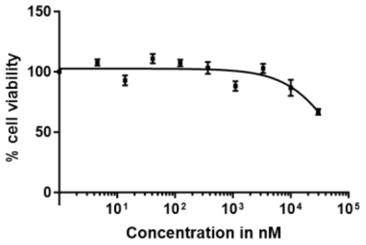

IC$_{50}$ (39): n.d.

Replicate 3

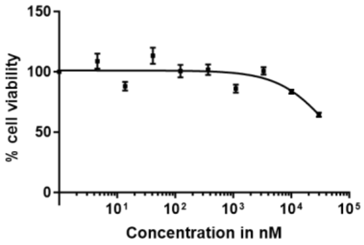

IC$_{50}$ (39): n.d.

Replicate 1

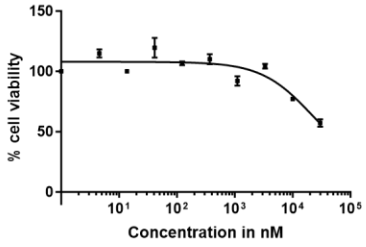

IC$_{50}$ (45): n.d.

Replicate 2

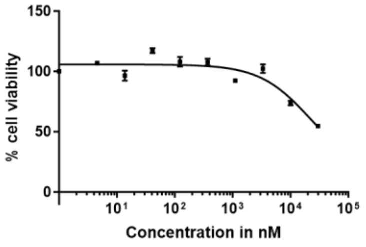

IC$_{50}$ (45): n.d.

Replicate 3

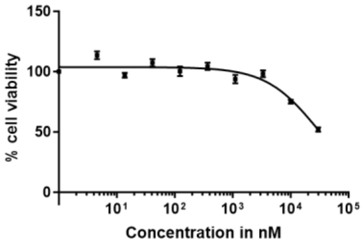

IC$_{50}$ (45): n.d.

Figure S 77: Cytotoxicity assay. BI-D1870, 39 and 45 were evaluated on NCI-H2030 NSCLC cells at concentrations ranging from 45.7 nM – 30 μM (n = 3). Cell viability was determined by XTT assay. The images were generated using GraphPad Prism 10.1.1. n.d. not determined (above limit).
